# Lucigenin: a strongly oxidizing dicationic photocatalyst for the direct azolation of arenes

**DOI:** 10.1039/d6sc02111e

**Published:** 2026-05-06

**Authors:** Alexandra Matei, Baptiste Roure, Xiaobing Chen, Sebastian B. Beil, Ben L. Feringa

**Affiliations:** a Stratingh Institute for Chemistry, University of Groningen 9747AG Groningen The Netherlands b.l.feringa@rug.nl

## Abstract

The direct oxidative C–H amination of arenes has become an attractive method to bypass the classical multistep and sequential synthesis of arylamines. Most current approaches rely on photocatalysis, using strong photooxidants to generate an arene radical cation *in situ*, which can then be trapped by a nitrogen nucleophile. In this regard, the development of new photocatalysts capable of accessing oxidation potentials greater than +2.5 V has been a thriving field of research in recent years. Here, we report the use of commercially available Lucigenin—widely used as a fluorescent probe in biology—to achieve this transformation. This strategy features short reaction times, a wide azole scope, and avoids the use of additives or fluorinated solvents, a common limitation of many previous methodologies. Preliminary mechanistic studies are also described, suggesting that the initial formation of an azole radical cation could be involved.

## Abstract 2

Lucigenin, a widely used fluorescent probe in biology, was used for the first time in photocatalysis to achieve the direct C–H azolation of arenes.

## Graphical abstract

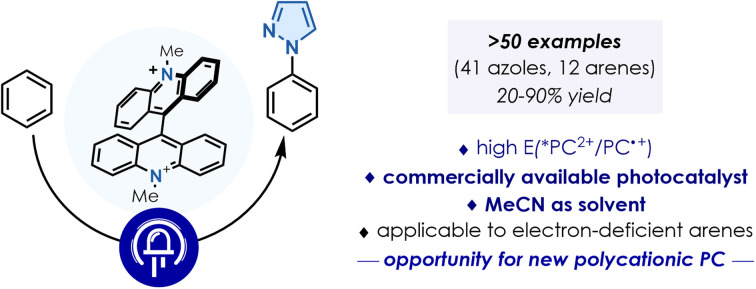

## Introduction

Over the last 20 years, photoredox catalysis—particularly metallophotoredox^1,2^ and organophotoredox^3,4^—has emerged as one of the most powerful methodologies to access high energy intermediates (mainly radicals) by harnessing the highly oxidizing or reducing nature of the excited states of various photocatalysts. In this regard, a variety of studies have been published on topics ranging from cross-coupling to C–H activation^5^ or even transformations involving radical intermediates.^6,7^ The tunability of the photocatalyst structure—particularly in the case of organophotoredox catalysis—has enabled organic chemists to obtain diverse physicochemical properties, spanning from high excited-state life times, high quantum yields,^4^ strong excited state oxidation and reduction potentials,^8–10^ hydrogen-atom transfer (HAT) properties^11^ to red-shifted absorption of the applied light.^12–14^

**Fig. 1:**
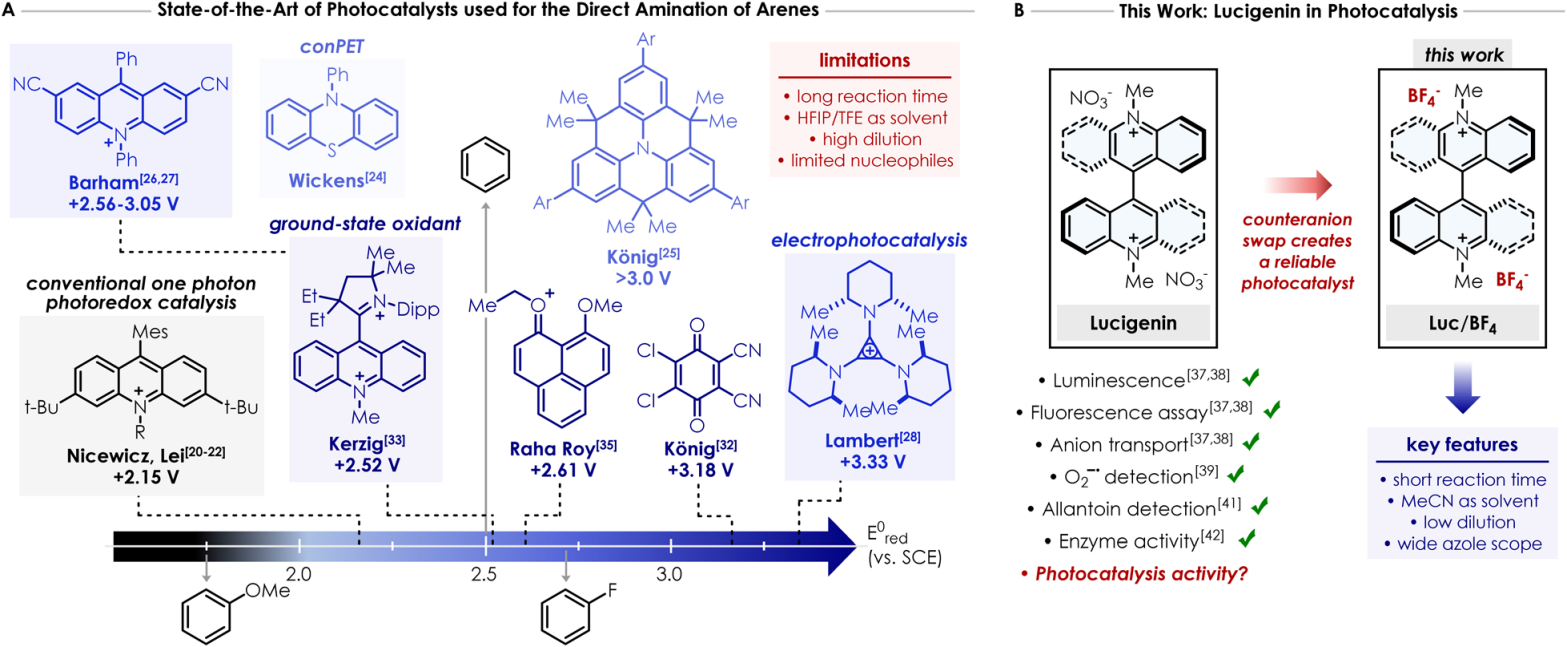
(A) State-of-the-art of photocatalysts used for the direct amination of arenes in the literature with various methods to access high oxidation potential (*i.e.* conPET, ground-state oxidant or electrophotocatalysis). conPET = consecutive Photoinduced Electron Transfer process. TFE = 2,2,2-trifluoroethanol. HFIP = 1,1,1,3,3,3-hexafluoro-2-propanol. SCE = Standard Calomel Electrode. Ar = 2,4-difluorobenzene. (B) This work: counterion swap transforms Lucigenin into a reliable and strongly oxidizing photocatalyst for the direct azolation of arenes.

A prototypical example is the direct C–H coupling between nitrogen nucleophiles—such as amines, amides or azoles—and arenes, including benzene derivatives. The direct introduction of nitrogen based functionalities onto aromatic rings is crucial, considering the broad applicability of these compounds, particularly in the pharmaceutical industry. Such transformation actually bypasses classical methods such as the widely applied Buchwald–Hartwig coupling^15–17^ or nucleophilic aromatic substitutions (S_N_Ar),^18,19^ which rely on the sequential, multistep functionalisation of arene derivatives to introduce the N-moiety and, additionally require a transition metal catalyst. In 2015, Nicewicz and co-workers reported a pioneering study showing that a combination of an acridinium-based photocatalyst with oxygen and catalytic amounts of 2,2,6,6-tetramethyl-piperidine-1-oxyl (TEMPO) enables the oxidative amination of electron-rich arenes (grey box in Fig. 1A).^20–22^ However, this transformation is restricted to highly electron-rich arenes like anisole or mesitylene and it could not be applied to simple benzene or more electron-deficient derivatives—such as fluorobenzene or chlorobenzene—since their oxidation potentials are beyond the reach of this class of photocatalysts.^23^ Therefore, the development of an organic photocatalyst that provides (a) a broader redox window capable of driving transformations previously out of reach (*i.e. E* ≥ +2.5 V *vs.* SCE), (b) enhanced functional group compatibility, and (c) fast and straightforward synthesis, would require fundamentally distinct concepts and photocatalysts design. Wickens and colleagues described the direct C–H azolation of arene using *N*-phenylphenothiazine (PTH) as the photocatalyst and oxygen as the sole oxidant (light blue box in Fig. 1A).^24,25^ Two consecutive electron-transfer events—*i.e.* a conPET mechanism—converted a conventional photoreductant into a highly potent photooxidant capable of promoting this transformation. While this protocol was efficient for benzene and methylated arenes, more electron-deficient arenes such as chlorobenzene, formed the azolated product in low yield. Both Barham^26,27^ and Lambert^28^ independently reported electrophotocatalytic approaches that generate the true potent superoxidant—a radical cation—*in situ via* electrochemical oxidation, followed by photoexcitation (blue box in Fig. 1A). In a conPET mechanism, the overall driving force results from the combination of one electron and one photon. In principle, this strategy could access oxidation potential even exceeding +3.0 V *vs.* SCE. Despite the attractiveness of this tactic, photoelectrochemistry^29–31^ requires tailored electrochemical setups and involves a more intricate conceptual framework than Nicewicz's arene amination, which operates *via* a purely photochemical process. Other contributions to this field employ ground-state oxidants as photocatalysts.^32–36^ König and co-workers described the use of 2,3-dichloro-5,6-dicyano-1,4-benzoquinone (DDQ)—which can act as a strong organic photooxidant under irradiation—for the functionalisation of arenes with various nucleophiles including amides, carbamates, sulfonamides, and pyrazoles (Fig. 1A).^32^ While the protocol was efficient for amides in general, the reactivity of azoles remained largely restricted to electron-rich arenes. In recent years, several groups have developed a variety of ground-state oxidant structures (mostly based on mono- or dicationic compounds) that could act as potential strong photooxidants upon photoexcitation. Notably, Kerzig and Hansmann reported a dicationic acridinium/carbene hybrid photocatalyst based on the Fukuzumi catalyst (dark blue box in Fig. 1A),^33^ Choudhury *et al.* used a N-fused nitrenium structure,^34^ while Roy and colleagues employed a phenalenyl-type structure as the photocatalyst (Fig. 1A).^35^

Albeit elegant and effective, all described strategies required, (a) the use of DDQ, which narrows the applicability, and the use of *tert*-butyl nitrite as an external terminal co-oxidant;^32^ (b) the utilisation of an additional Lewis acid like LiClO_4_ to preclude the deactivation of PTH photocatalyst due to the generation of inhibitory reactive oxygen species;^24,33^ (c) long reaction times; and (d) the use of HFIP or TFE as main or exclusive solvents in most of the cases, despite their cost and rising concerns regarding PFAS compounds in general. Therefore, the main goal of this work is to design an easily accessible and commercially available cationic photocatalyst capable of addressing some of the challenges aforementioned, particularly avoiding the use of fluorinated solvents and shortening the reaction time. Herein, we present the use of Lucigenin—a widely used probe in biology for anion transport^37–39^ and its chemiluminescence properties^37,38,40–42^—for the direct C–H azolation of arenes (Fig. 1B).

## Results and discussion

Our group recently reported that a “mixed” thioxanthene-acridinium (TX-Acr) switch possesses redox properties that lie between those of its parent symmetric analogs (*i.e.* bisthioxanthylidene BTX (ref. 43 and 44) and Lucigenin Luc^2+^).^45^ During this study, we were curious to see if the interesting redox properties of the Lucigenin hybrids could be applied in the case of photoredox catalysis and more specifically for the oxidative C–H amination of arenes. While Tx-Acr and BTX showed very poor photocatalytic activity, Luc/ClO_4_ (where the counteranion of commercially available Lucigenin is simply swapped from nitrate to perchlorate for better solubility in organic solvents, see SI Section 2)^45^ gave a promising result when pyrazole was reacted with a large excess of benzene (220 equivalents), forming the corresponding product in 53% yield (see SI Section 6).

Based on this encouraging result, we started our optimisation efforts by evaluating similar dicationic species, as well as different lucigenin salts bearing a variety of counterions for the model reaction between benzene (1a) and 1*H*-pyrazole (2a). Subjecting commercially available lucigenin nitrate salt under the standard reaction conditions, showed very little conversion into desired product (Table 1 and entry 2). Additionally, modifying the photocatalyst structure by changing the methyl substituent to a phenyl substituent, also did not facilitate the transformation, for neither the nitrate salt (see SI Section 6.1) nor the BF_4_^−^ salt (Table 1 and entry 3). In our evaluation of different lucigenin salts, BF_4_^−^ was determined to be the optimal counterion, while counterions such as PF_6_^−^ and BAr^F^_4_^−^ performed slightly worse (Table 1 and entry 4). Investigation of the catalyst loading demonstrated that even 1 mol% of Luc/BF_4_ is sufficient to achieve yields as high as 54% (Table 1 and entry 5), while 10 mol% does not enable further conversion of starting material into the desired product (Table 1 and entry 6). Light irradiation was required for the desired transformation to occur (see SI Section 13.1), and the wavelength as well as the light intensity were both important for the performance of the reaction. Consistent with literature reports, the system can be irradiated at different wavelengths, however, a higher performance was observed at 450 nm and at 100% light intensity compared to 390 nm (Table 1 and entry 7). Reducing the light intensity to 50% required an increase in reaction time, thus we proceeded with 450 nm, 100% light intensity and 2 h irradiation as optimal conditions. To confirm if the oxygen atmosphere was required, the transformation was conducted under inert conditions, and as expected a very low conversion to the desired product was observed (Table 1 and entry 9). Sparging the solution with O_2_ for longer periods of time up to 30 min, did not improve the conversion into product 2b (Table 1 and entry 10). In previous reports, a large excess of benzene was usually required to facilitate the conversion, thus we also investigated if this was the case for our photocatalyst. Surprisingly, a large excess was demonstrated to inhibit the transformation (Table 1 and entry 11), suggesting that solubility could be a key element of the reaction performance. Reducing the equivalents of benzene also decreases the conversion into the desired product (Table 1 and entry 12) limiting us to 110 : 1 benzene:pyrazole as the optimal ratio (see SI Section 6.5). Concentration proved crucial for the performance of the transformation, with 0.3 M in MeCN as the optimal concentration. Both lower (Table 1 and entry 13) and higher (Table 1 and entry 14) concentrations interfered with the conversion into product 2b, while small variations of 0.05 M already showed a decrease in yield and running the reaction neat only afforded traces of desired product (see SI Section 6.6). Considering the limitation regarding solvent choices in the literature, we were highly motivated to find greener, non-fluorinated solvent alternatives. While the common solvent mixture of TFE : HFIP performed worse than MeCN (Table 1 and entry 15), we observed that conducting the reaction in HFIP alone achieved slightly higher yields of product 2b (Table 1 and entry 16). However, considering current concerns with fluorinated solvents, and no other solvents performing better in the transformation (see SI Section 6.7) we opted for MeCN as our solvent choice. Overall, under the optimised reaction conditions the desired product is obtained in 69% isolated yield (Table 1 and entry 1).

**Table 1:** Reaction optimisation^a^

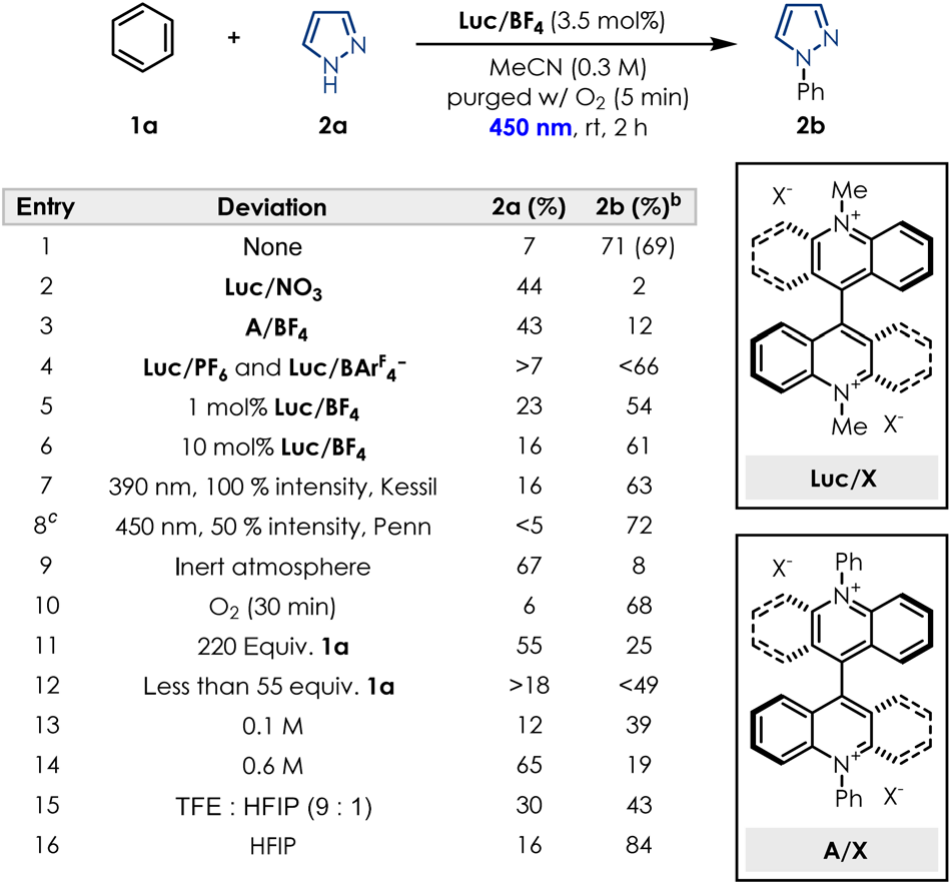

a Reactions were performed using 0.2 mmol of pyrazole SM, benzene (2 mL, 110 equiv.) Luc/BF4 (3.5 mol%) and MeCN (0.3 mL) and irradiated for 2 h in a Penn PhD Photoreactor M2 equipped with 450 nm wavelength at 100% intensity unless otherwise stated. Yield in parentheses refers to the isolated product.

b Yields determined by ^1^H NMR using 1,3-dinitrobenzene as internal standard.

c Increased reaction time to 4 h.

**Scheme 1:**
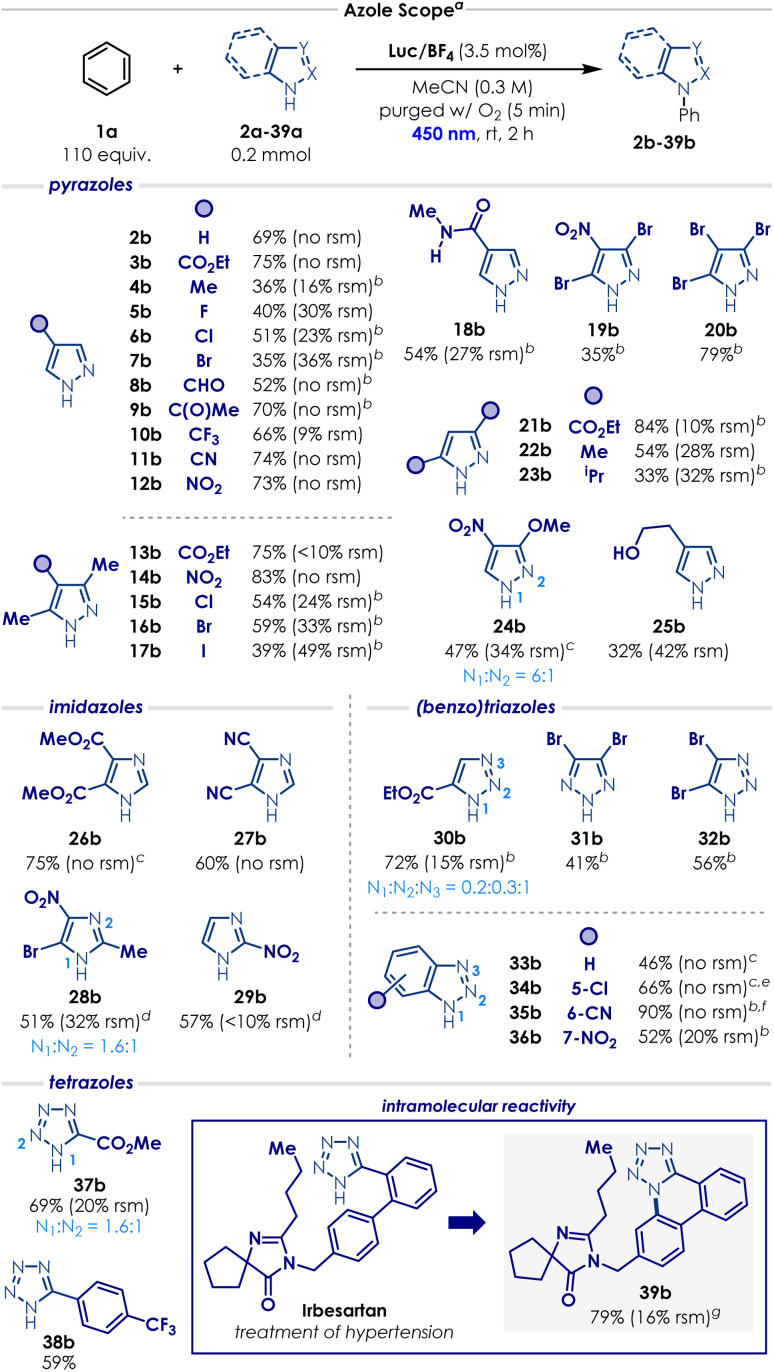
Scope of azoles. ^*a*^ Isolated yields are reported. Reactions were performed using 0.2 mmol of heterocycle, arene (2 mL), Luc/BF_4_ (3.5 mol%), and MeCN (*c* = 0.3 M, 0.65 mL) under irradiation for 2 h in a Penn photoreactor M2 with a 450 nm light source. ^*b*^ The reaction was irradiated for 4 h. ^*c*^ HFIP was used as solvent. ^*d*^ The reaction was irradiated for 6 h, using 7 mol% of Luc/BF_4_ and *c* = 0.2 M. ^*e*^ Regioisomers were obtained, *r.r.* is N_1_ : N_3_ = 0.9 : 1 ^*f*^ Regioisomers were obtained, *r.r.* is N_1_ : N_2_ : N_3_ = 0.54 : 0.16 : 0.3. ^*g*^ The reaction was run without PhH and using MeOH as the solvent (*c* = 0.1 M, 2 mL). Detailed reaction conditions (solvent, concentration, photocatalyst loading, reaction time and photoreactor parameters) for each azole example are provided in the SI Section 8. rsm = recovered starting material.

With the optimal conditions in our hands, the scope of the process was evaluated, starting with the azole substrates (Scheme 1). Overall, the transformation proved compatible with a wide variety of functional groups and azole derivatives. Pyrazoles bearing strongly electron-withdrawing substituents such as –CO_2_Et, –C(O)Me, –CF_3_ and –CN at the C4 position underwent productive reactions with benzene, delivering the corresponding *N*-phenyl pyrazole products (3b, 9b–11b) in good yields (66% to 75%). More sensitive electron-poor groups at the C4 position—such as carbaldehyde 8a (susceptible to oxidation); nitro 12a (prone to photoexcitation); or amide 18a— were also well-tolerated, affording the desired products (8b, 12b and 18b) in remarkable 52%, 73% and 54% yield, respectively. Other weakly electron-donating substituents at the C4 position such as -Me, –F, -Cl, -Br (4a–7a) displayed a quite distinct reactivity trend. In these cases, a significant amount of decomposition was observed, and the standard reaction time resulted in poor conversion. Gratifyingly, extending the reaction time to 4 h enabled the formation of the corresponding *N*-phenyl pyrazole products (4b–7b) in moderate but synthetically useful yields (35 to 51%). More sensitive electron-rich groups, like a pyrazole with a free hydroxyethyl group 25b, also provided the corresponding product, albeit in a relatively low yield (32%, 42% rsm). We then evaluated a range of di- and trisubstituted pyrazoles (13a–17a, 19a–24a). A similar reactivity trend emerged: ester- and nitro-substituted derivatives (13a–14a, 21a) afforded the desired product in excellent yields (75 to 84%), whereas halide- and alkyl-substituted pyrazoles (15a–17a, 22a–23a) required longer reactions times and furnished the corresponding *N*-phenyl pyrazoles in low to moderate yields (33 to 59%). Pyrazole 24a reacted quite poorly in MeCN—probably due to its poor solubility—but switching the solvent to HFIP enabled the formation of the desired product 24b in 47% yield. Trisubstituted pyrazole 19a and 20a are frequently used in industry as entry points for the modular synthesis of drug leads and their derivatives during structure-activity relationship campaigns (SAR).^46–48^ We were pleased to see that, under our optimised conditions, the corresponding products 19b and 20b were obtained in 35% and 79% yield, respectively.

The scope evaluation was continued with other azoles (Scheme 1). Even though imidazoles generally performed moderately in our case, this motif still represents one of the major limitations of the previous reports on the oxidation C–H amination of arenes.^20,28,32,33^ By simply changing the solvent from MeCN to HFIP—primarily due to solubility issues, electron-deficient imidazole 26a gave the corresponding *N*-phenyl imidazole 26b in high yield (75%). Imidazole 27a reacted efficiently under the standard conditions, affording product 27b in 60% yield. For imidazoles 28a and 29a, slow reactivity and solubility issues required slight adjustments to the reaction parameters: increasing the photocatalyst loading, decreasing the concentration and increasing the reaction time. With these modifications, the corresponding *N*-phenyl imidazole 28b and 29b were delivered in 51% and 57%, respectively. Triazoles and benzotriazoles also engaged in the C–H amination of arenes (Scheme 1). Triazoles 30a, 31a and 32a furnished the corresponding *N*-phenyl triazoles 30b, 31b and 32b in moderate to good yields (41–72%; as a mixture of regioisomers for 30b). Substituting the solvent from MeCN to HFIP to address solubility issues enabled benzotriazole 33a to deliver the desired product in 46% yield. Introducing a chloro, cyano or nitro substituent onto the benzotriazole core did not significantly impact the reactivity: benzotriazoles 34a, 35a and 36a provided the *N*-phenyl products in moderate to excellent yields (52–90%; as a mixture of regioisomers for 34b and 35b). Finally, tetrazoles 37a and 38a produced the corresponding *N*-phenyl compounds in good yields (59–69%; as a mixture of regioisomers for 37b). We also sought to evaluate the transformation on a more complex substrate and we chose Irbesartan (39a), a tetrazole-containing drug used for the treatment of hypertension (Scheme 1). Under the standard conditions, the drug was completely insoluble and no reaction occurred. Interestingly, when MeOH was introduced as a cosolvent, a new unexpected intramolecular cyclisation product 39b started to form. By removing benzene and slightly decreasing the reaction concentration, we managed to obtain the annulated tetrazole 39b in excellent yield (79%).

**Scheme 2:**
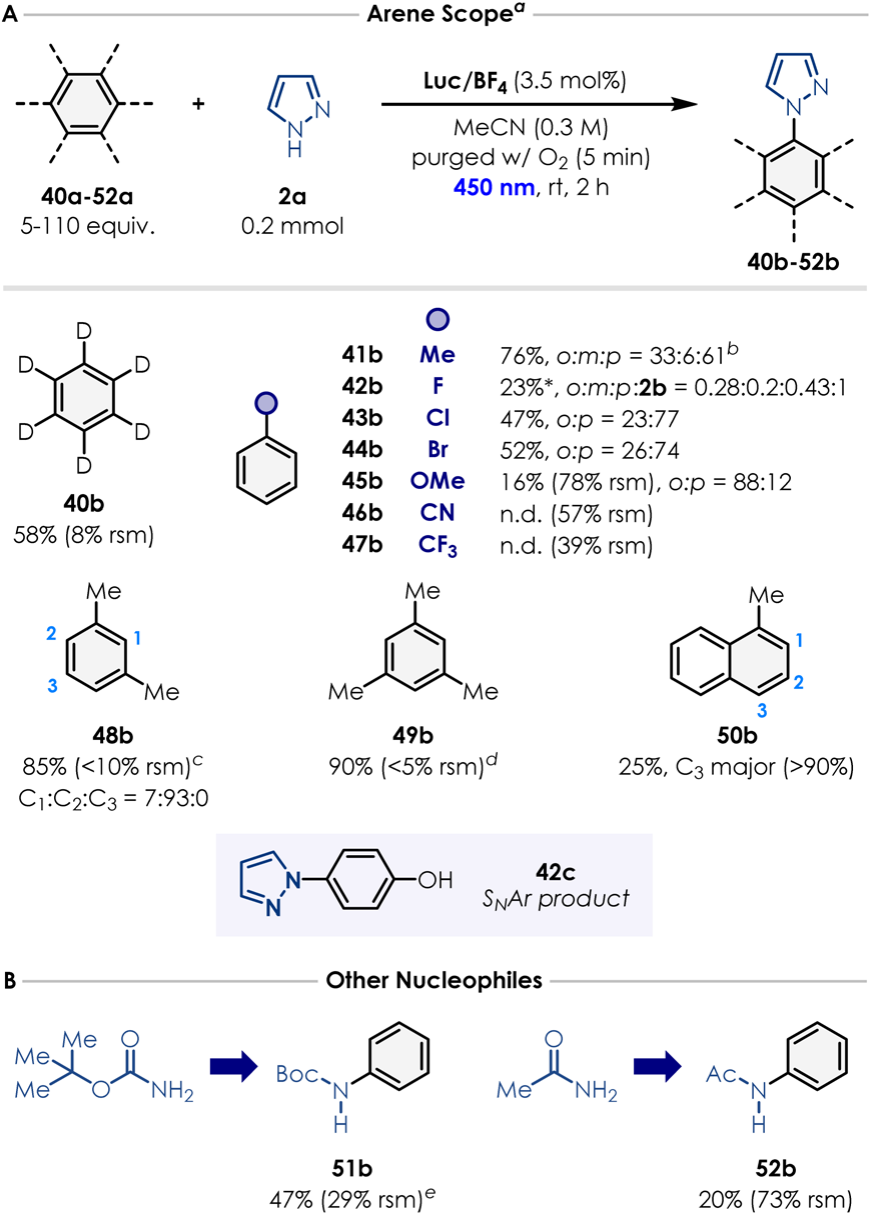
(A) Scope of arenes. ^*a*^ Isolated yields are reported. Reactions were performed using 0.2 mmol of pyrazole, arene (5–110 equiv.), Luc/BF_4_ (3.5 mol%), and MeCN (*c* = 0.3 M, 0.65 mL) under irradiation in a Penn Photoreactor M2 with a 450 nm light source. ^*b*^ 94 equiv. of the arene were used. ^*c*^ 80 equiv. of the arene were used. ^*d*^ 5 equiv. of the arene were used. *A S_N_Ar product was also isolated in 25% yield. (B) Other nucleophiles. ^*e*^ Standard reaction conditions but irradiating the reaction for 6 h. Detailed reaction conditions (solvent, concentration, photocatalyst loading, reaction time and photoreactor parameters) for each arene example are provided in the SI Section 8.

**Fig. 2:**
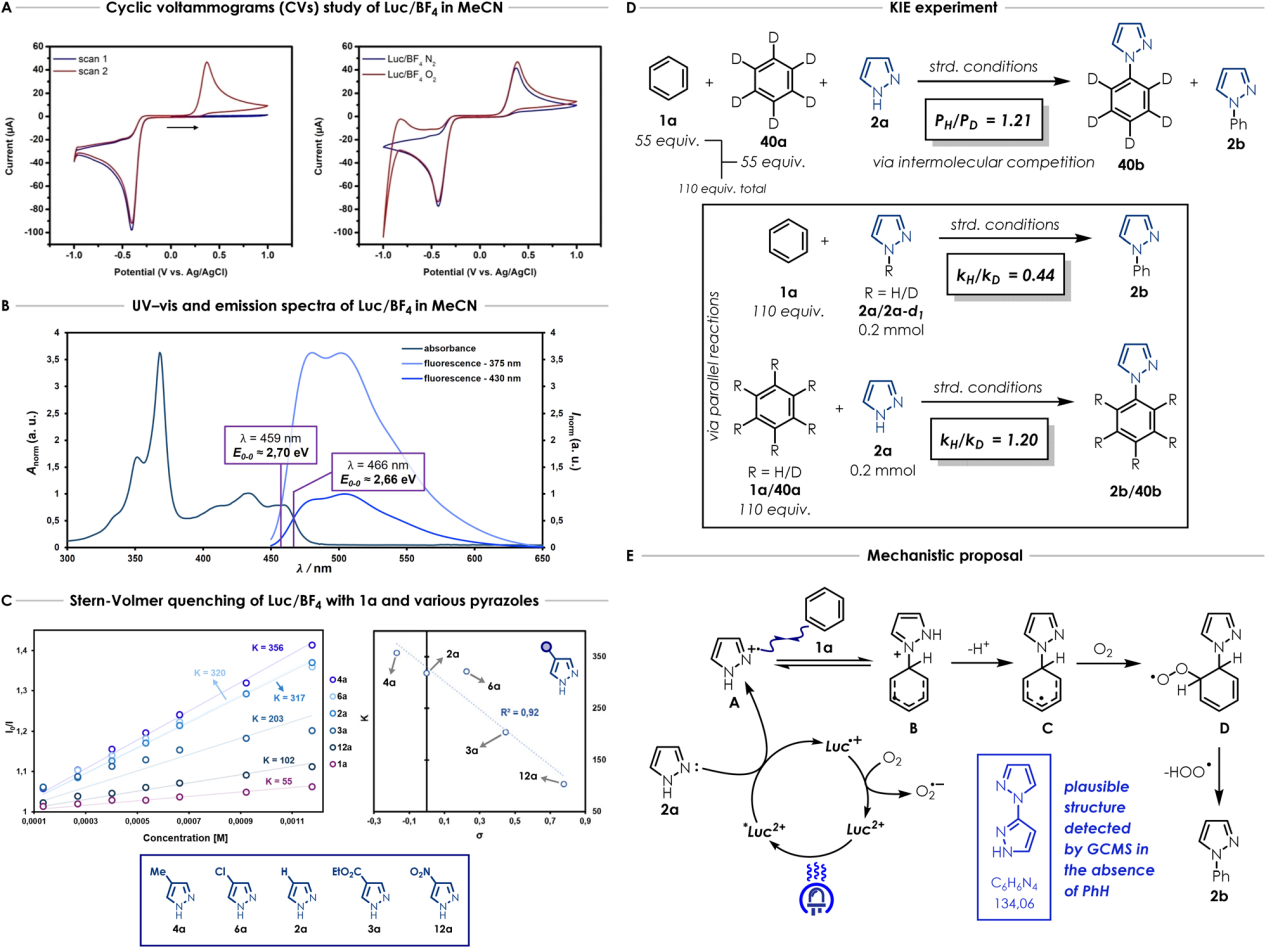
(A) Cyclic voltammograms of Luc/BF_4_ in MeCN showing the impact of scans and atmosphere. A scan rate of 100 mV s^−1^ was used for all CV experiments. (B) Calibrated UV-vis absorption spectra and normalised emission spectra of Luc/BF_4_ in MeCN for 375 nm and 430 nm excitation maxima respectively, along with *E*_00_ determination. (C) Stern–Volmer quenching plots of Luc/BF_4_ in MeCN with various amount of 1a or different pyrazoles (2a, 3a, 4a, 6a, 12a) and plot of the Stern–Volmer quenching constants *K vs.* the corresponding Hammett *para*-substituent constants *σ*. *K* is defined as the slope of the Stern–Volmer plot, see SI Section 12.1 for more details (D) KIE experiments. Standard conditions = 0.2 mmol of pyrazole, arene (2 mL), Luc/BF_4_ (3.5 mol%), and MeCN (*c* = 0.3 M, 0.65 mL) under irradiation in a Penn Photoreactor M2 with a 450 nm light source. Kinetic constants for the KIE study were calculated by the initial rate method, see the SI Section 13.3 for more details. *P*_H_ corresponds to the yield of 2b and *P*_D_ corresponds to the yield of 40b. (E) Mechanistic proposal for the direct azolation of arenes.

Finally, we evaluated the arene substrate scope (Scheme 2). Toluene, *m*-xylene and mesitylene afforded the corresponding *N*-arylated pyrazoles (41b, 48b–49b) in excellent yields (76–90%; as a mixture of regioisomers for 41b and 48b). In the case of mesitylene, 5 equivalents of the arene were sufficient to carry out the reaction. Notably, benzene-*d*_*6*_ performed relatively similarly to benzene, providing the deuterated *N*-phenyl-*d*_5_ product 40b in good yield (58%). As discussed, accessing oxidation potentials greater than +2.5 V *vs.* SCE enables to use electron-poor arenes that were unreactive under many previously reported conditions from the literature.^20,22,24^ We were pleased to see that chlorobenzene and bromobenzene afforded the desired products 43b (*o*3 : *p* = 23 : 77) and 44b (*o*3 : *p* = 26 : 74) in yields comparable to the literature (47% and 52% respectively).^33–35^ Remarkably, even fluorobenzene could be employed as an arene partner, giving the desired product 42b in 20% yield as a mixture of isomers, along with the defluorinated product 2b (*o*3 : *m*3 : *p* : 2b = 0.28 : 0.2 : 0.43 : 1). Product 42c was also isolated in 25% yield, which is supposed to form *via* S_N_Ar onto the *para* product obtained with fluorobenzene. Anisole (45a) also coupled successfully with pyrazole, albeit with low efficiency (16% yield after 2 h). Benzonitrile (46a) and trifluorotoluene (47a) did not engage in the desired reactivity, possibly because of the strong polarity mismatch existing between these highly electron-poor arenes and the azole radical cation forming over the course of the reaction (Fig. 2F). Finally, 1-methylnaphthalene furnished the corresponding pyrazole product 50b as a mixture of isomers in low yield (25% with C3 as major regioisomer). We also explored nucleophiles beyond azoles. Preliminary results showed that BocNH_2_ (51a) and acetamide (52a) successfully reacted with benzene, forming the corresponding product in 47% and 20% yield, respectively.

Remarkably, this methodology performs similarly well compared to approaches reported in the literature (see SI Section 14.2 for more details). Therefore, it may serve as a viable alternative to cross-coupling reactions typically employed in previous studies (*e.g.* Chan-Lam or Ullmann coupling) which require a metal catalyst, prefunctionalised arene substrates (*e.g.* iodobenzene or phenylboronic acid) or even forcing conditions in some cases.

### Mechanistic investigations

In order to gain mechanistic insights about the photocatalyst and its behaviour in the model reaction, the redox properties of Luc/BF_4_ were investigated by cyclic voltammetry (CV). We first evaluated the importance of the scanning direction, to gain insights into the photocatalyst species observed. We expected that the photocatalyst would need to be reduced to Luc^·+^ first and then oxidized to Luc^2+^. This is confirmed by first scanning in the anodic direction and observing an absence of an oxidation wave. Once the first sweep is complete, and the reduction wave is observed, the second scan displays the corresponding oxidation wave (Fig. 2A). Consistent with this interpretation, initiating the experiment in the cathodic direction results in a full redox couple already in the first scan (see SI Section 10.1). Additionally, Luc/BF_4_ exhibited stability over 20 cyclic voltammetry cycles, while the commercial Luc/NO_3_ displayed large decreases in current over 20 cycles, also confirming the importance of the counterion (see SI Section 10.2 for more details).

For quantitative analysis purposes, the second scan of the Luc/BF_4_ cyclic voltammogram was therefore used to extract the anodic and cathodic peak potentials values (respectively *E*_pa_ and *E*_pc_) of +0.38 V and −0.43 V *vs.* Ag/AgCl in the ground state. Considering the requirement for an oxygen atmosphere (Table 1 and entry 9), CVs of Luc/BF_4_ were measured both in the absence and presence of oxygen. Under inert atmosphere, Luc/BF_4_ displays substantial redox hysteresis (Fig. 2A). The large separation between the anodic and cathodic peak potentials indicates that this is not a simple reversible one-electron transfer, but instead reflects electron transfer coupled to significant structural reorganisation, consistent with related lucigenin-derived overcrowded alkene systems.^45^

To evaluate the photooxidant strength of Luc/BF_4_, we recorded its UV-Vis absorption and emission spectra in MeCN (Fig. 2B), giving the excited-state energy as *E*_O,O_ = 2.70 eV, consistent with the values reported for comparably strong dicationic photocatalysts.^27,28,32–35^ Using this value, we estimated an excited-state reduction potential of 
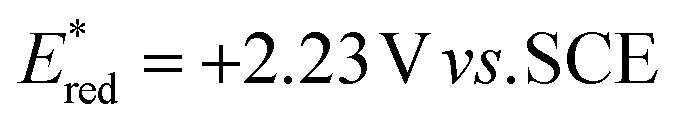
 (see SI Section 11 for detailed calculations). While this calculation provides an initial approximation, it underestimates the oxidizing strength of our photocatalyst. Indeed, based on our azole scope, we determined that substrates with oxidation potentials beyond +2.23 V *vs.* SCE such as 3a (+2.50 V *vs.* SCE) and 6a (+2.41 V *vs.* SCE) (see SI Section 10.4 for CV measurements) can react efficiently in the presence of our photocatalyst Luc/BF_4_, suggesting a higher value for the excited-state reduction potential than the approximation reported above. Because the redox event involves two electrons, a more detailed analysis is necessary and this is part of ongoing mechanistic research in our laboratory. Therefore, the calculated value should be considered as an estimate rather than definitive photooxidant strength of Luc/BF_4_.

Several Stern–Volmer quenching studies were conducted to determine how substituents on the pyrazole impact the quenching rate *vs.* benzene (Fig. 2C). We first investigated the luminescence quenching efficiency of the different substrates in the model reaction. Stern–Volmer quenching studies in MeCN and MeCN:benzene mixture, show a considerably higher quenching rate for pyrazole (2a) (*K* = 317 and *K* = 247), suggesting a faster reductive quenching of Lucigenin compared to benzene (1a) (*K* = 55) (Fig. 2C, SI Section 12.1).

We also conducted Stern–Volmer quenching studies comparing the quenching rates of substituted pyrazoles. Among various electron-rich and -poor functional groups, we observed a trend for the different pyrazole derivatives (Fig. 2C). Strong electron-withdrawing substitutions such as nitro 12a (*K* = 102) and ethoxycarbonyl 3a (*K* = 203) lead to lower quenching rates which is consistent with their higher oxidation potentials (see SI Section 12 for more details). Substituents which are weakly electron-withdrawing such as chloro 6a (*K* = 320) or electron-donating such as methyl 4a (*K* = 356), display higher quenching constants which are closer to the unsubstituted pyrazole (2a). While we observe substituent-dependent trends among pyrazole derivatives, when compared to benzene, this remains the slowest quencher in the series (mauve plot in Fig. 2C), reinforcing the conclusion that both unsubstituted and substituted azoles may be preferentially oxidized by the excited state *Luc^2+^. In Fig. 2C the correlation between the quenching rate constants obtained for pyrazoles 2a (H), 3a (CO_2_Et), 4a (Me), 6a (Cl) and 12a (NO_2_) and the Hammett substituent constants is displayed.^49^ Overall, a trend between the electron-richness of the pyrazole (linked to the inductive and resonance effect *via* the Hammett substituent constants) and the quenching rate constant of Luc/BF_4_ appears. However, additional experiments are required to fully understand how this trend is linked to the reactivity observed for this transformation.

We also performed an extensive kinetic isotope effect (KIE) study (Fig. 2D).^50,51^ Intermolecular KIE competition experiment study (Fig. 2D).^50,51^ Intermolecular KIE competition experiment reveals a small, perhaps secondary, kinetic isotope effect when the model reaction is carried out with benzene (1a) and benzene-*d*_*6*_ (40a) together (*P*_H_/*P*_D_ = 1.21, Fig. 2D). Remarkably, when two parallel reactions were conducted with pyrazole (2a) and pyrazole-*d*_*1*_ (2a-*d*_6_), a strong inverse kinetic isotope effect was observed (*k*_H_/*k*_D_ = 0.44, Fig. 2D). Finally, benzene (1a) and benzene-*d*_*6*_ (40a) were used in two parallel reactions and the results showed in this case as well a small, possibly secondary, kinetic isotope effect (*k*_H_/*k*_D_ = 1.20, Fig. 2D), corroborating the result observed for the intermolecular KIE competition experiment. Overall, the KIE study indicated that the C–H bond cleavage on the arene partner was not involved in the rate-determining step and that the N–H/N–D bond from the pyrazole plays a significant role during the transformation.

A control experiment performed in the absence of benzene showed partial decomposition of the pyrazole, and GC-MS analysis enabled to detect a peak with a mass that could correspond to the structure of a bipyrazole derivative. At the moment, the exact structure of this intermediate is not known; however, the fragments obtained by GC-MS suggest that 1′*H*-1,3′-bipyrazole (shown in the blue box in Fig. 2F) is a likely candidate (see SI Section 13.4 for more details). This observation suggests a potential PCET-type (proton-coupled electron transfer) mechanism involving the formation of a pyrazole radical cation intermediate—which could dimerize into 1′*H*-1,3′-bipyrazole in the absence of benzene (see SI Section 13.4 for more details). When the reaction was carried out with varying amounts of TEMPO, almost complete recovery of the starting material was observed, and only traces to low amounts of product were detected by ^1^H NMR depending on the TEMPO loading. This result is consistent with the intermediacy of radical species in the reaction pathway (see SI Section 13.4).

Considering all experiments reported in this work and the results from prior studies on this reaction class with strongly oxidizing photocatalytic systems, a plausible mechanism can be outlined as shown in Fig. 2E. First, photoexcitation of Luc/BF_4_ would create a strong photooxidant *in situ*
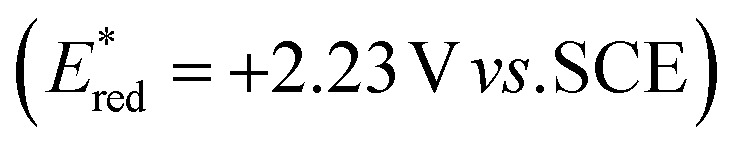
 that could in theory oxidize either pyrazole (2a) or benzene (1a). According to the fluorescence quenching studies and across most of the azoles studied here, we propose that pyrazole (2a) may be getting oxidized faster and therefore, radical cation intermediate A would be generated *via* a one-electron oxidation. This highly electrophilic radical can then add onto benzene to form the radical cation intermediate B that can transform into the radical intermediate C after deprotonation. The delocalized radical on the phenyl ring can then be trapped by oxygen and subsequently get oxidized to the expected product 2b after elimination of hydroperoxyl radical (ultimately forming hydrogen peroxide, see SI Section 13.5 for more details). Alternatively, the superoxide anion (O_2_˙^−^) forming during the photocatalyst regeneration with oxygen can also directly abstract one hydrogen from the radical intermediate C to deliver the expected product along with hydrogen peroxide (see SI Section 13.5 for more details).^33,35^

Interestingly, the involvement of an azole radical cation in the proposed mechanism differs from what is generally reported in the literature (*i.e.* benzene oxidation).^20,21,28,32,33^ Only two reports suggested that the oxidation of the pyrazole could take place instead but HFIP was utilised as the solvent in both cases.^34,35^ To our knowledge, the involvement of a azole radical cation intermediate in MeCN has never been reported previously. Given the excess of benzene used in this reaction, the formation of the benzene radical cation cannot be ruled out completely at this time and ongoing mechanistic research in our laboratory is conducted to decipher which species is actually forming over the course of the reaction (see SI Section 13.6 for more details).

## Conclusions

In summary, this work repurposes Lucigenin—a widely used chemical probe in biology—as a strongly oxidizing photocatalyst based on a bisacridinium structure. This dication is readily accessible *via* anion exchange of commercially available Lucigenin. We subsequently employed this photooxidant in the direct oxidative C–N coupling of arenes with various azoles. After extensive optimisation, this methodology was applicable to a broad substrate scope—featuring a wide range of functional groups and even electron-deficient arenes that conventional acridinium photocatalysts fail to engage, clearly highlighting the critical role of polycationic photocatalyst species. Our approach features the use of relatively high concentration and short reaction times, and it also avoids the use of HFIP and TFE, solvents required in most previously reported strategies. Preliminary mechanistic investigations, including Stern–Volmer quenching, fluorescence and UV-vis spectroscopy, control experiments, cyclic voltammetry study, and KIE experiments, suggest an azole radical cation could be involved in the transformation and showcase the strength of Luc/BF_4_ as a versatile photooxidant. Notably, since this method is performing comparably to cross-coupling approaches reported in the literature, it could serve as a viable alternative for the straightforward synthesis of *N*-arylated azoles. Considering the modularity of the synthesis of bisacridiniums, we envision this work opens an avenue for further research towards the design of novel, highly potent polycationic photooxidants for direct photocatalytic transformations.

## Author contributions

A. M. and B. R. contributed equally. A. M., B. R. and B. L. F. conceived the project. A. M. and B. R. synthesised the compounds and carried out all experimental studies and characterisations. A. M. carried out the CV study. B. R. carried out the UV-vis study and the KIE experiments. A. M. and B. R. performed all the Stern–Volmer quenching and fluorescence experiments. X. C. synthesised photocatalysts Luc/BF_4_, Luc/ClO_4_, Luc/PF_6_, A/ClO_4_, A/BF_4_. All authors contributed to the writing and proofreading of the manuscript. B. L. F. and S. B. B. guided and supervised the research.

## Conflicts of interest

The authors declare no conflicts of interest.

## Supplementary Material

SC-OLF-D6SC02111E-s001

## Data Availability

The authors confirm that the data supporting the findings of this study are available within the article and its supplementary information (SI). Supplementary information: synthetic procedures, experimental details, reaction setup details, SI figures and characterisation of compounds. See DOI: https://doi.org/10.1039/d6sc02111e.
