# Supplementary material for: Lucigenin: a strongly oxidizing dicationic photocatalyst for the direct azolation of arenes
Source: Chem Sci. 2026 May 6;17(23):11436–44. doi: 10.1039/d6sc02111e (PMC13148268; doi:10.1039/d6sc02111e)
Supplement: SC-017-D6SC02111E-s001 [file SC-017-D6SC02111E-s001.pdf]

Supplementary Information (SI) for Chemical Science.
This journal is © The Royal Society of Chemistry 2026

Supplementary Information

# Lucigenin: A Strongly Oxidizing Dicationic Photocatalyst for the Direct Azolation of Arenes

Alexandra Matei,[a†] Baptiste Roure[a†], Xiaobing Chen,[a] Sebastian B. Beil[a,b] and Ben L. Feringa*[a]

*[a] Stratingh Institute for Chemistry, University of Groningen, 9747AG Groningen, the Netherlands;*

*[b] Current address: Max-Planck-Institute for Chemical Energy Conversion, Department for Electrosynthesis, Stiftstraße 34–36, 45470 Mülheim an der Ruhr, Germany;*

*† These authors contributed equally.*

* b.l.feringa@rug.nl

# Table of content


**1 General Experimental Details .... 4**

1.1 Optical Measurements .... 5

1.2 Photoreactors .... 5

**2 General Procedures .... 7**

**3 Synthesis of the photocatalysts .... 10**

**4 Structure of Photocatalysts .... 16**

**5 Structure of Starting Materials .... 17**

**6 Reaction Optimisation .... 19**

6.1 Influence of photocatalyst nature and counterion .... 20

6.2 Influence of photocatalyst loading .... 21

6.3 Influence of irradiation wavelength and intensity .... 22

6.4 Influence of atmosphere .... 24

6.5 Influence of benzene loading .... 25

6.6 Influence of concentration .... 26

6.7 Influence of solvent .... 27

6.8 Influence of additives (including bases) .... 28

**7 Pictures of Reaction Set-up .... 29**

**8 Scope .... 32**

8.1 Additional Substrate Scope Examples .... 64

**9 UV-Vis absorption studies .... 67**

9.1 UV-Vis spectra and Beer-Lambert law .... 67

9.2 Solvent effect on the UV-Vis absorption of Luc/$BF_4$ .... 69

9.3 EDA between benzene and Luc/$BF_4$ or pyrazole and Luc/$BF_4$ .... 70

9.4 EDA between benzene and pyrazole .... 73

9.5 UV-Vis spectra of Luc/$BF_4$ before and after irradiation .... 74

**10 Cyclic Voltammetry (CV) Experiments .... 76**

10.1 Cyclic voltammograms of Luc/$BF_4$ in MeCN .... 76

10.2 Cyclic voltammograms of Luc/$NO_3$ in MeCN .... 79

10.3 Cyclic voltammograms of Luc/$BF_4$ in HFIP .... 81

10.4 Cyclic voltammograms of different azoles and arenes in MeCN ..... 82

**11 Fluorescence, emission spectra and determination of $Ered*$ ..... 83**

**12 Initial fluorescence quenching experiments and Stern-Volmer studies ..... 85**

12.1 Stern-Volmer quenching in MeCN ..... 86

**13 Mechanistic Study ..... 96**

13.1 Light on & off experiment ..... 96

13.2 Reaction profile ..... 97

13.3 Kinetic Isotope Effect experiments (KIE) ..... 101

13.4 Control experiments ..... 107

13.5 $H_2O_2$ Detection Experiment ..... 112

13.6 Mechanistic proposal ..... 113

**14 Comparison with the literature ..... 116**

14.1 Comparison with other methodologies utilising photooxidants ..... 116

14.2 Comparison with cross-coupling methods (Chan-Lam/Ullmann) ..... 118

**15 NMR Spectra ..... 121**

**16 References ..... 193**

# 1 General Experimental Details

All required fine chemicals were purchased from TCI Chemicals, Merck, Fisher Scientific, Fluorochem Ltd and BLD Pharm. All of them were used directly without purification unless stated otherwise. $TBAPF_6$ was of electrochemical grade and obtained from Sigma Aldrich. Lucigenin was obtained as a nitrate salt from TCI chemicals or Fluorochem Ltd. All air and moisture sensitive reactions were carried out under an argon atmosphere using standard Schlenk manifold technique. All solvents were bought from Acros as 99.8% purity. $^{1}H$ and $^{13}C$ Nuclear Magnetic Resonance (NMR) spectra were acquired at various field strengths as indicated and were referenced to $CDCl_3$ (7.26 and 77.16 ppm for $^{1}H$ and $^{13}C$ respectively). $^{1}H$ NMR coupling constants are reported in Hertz and refer to apparent multiplicities and not true coupling constants. Data are reported as follows: chemical shift, integration, multiplicity (s = singlet, br s = broad singlet, d = doublet, t = triplet, q = quartet, p = quintet, sx = sextet, hp = heptet, m = multiplet, dd = doublet of doublets, etc.), proton assignment (determined by 2D NMR experiments: NOESY, HSQC and HMBC) where possible. $^{19}F$ NMR spectra were recorded using $CDCl_3$ as solvent and reported unreferenced. High-resolution mass spectra were obtained using an AEI-MS-902 mass spectrometer (EI+) or a LTQ Orbitrap XL (ESI+, ESI-, APCI+). Spectra were obtained using electron impact ionization (EI) and chemical ionization (CI) techniques, or positive electrospray (ES). GC-MS was carried out on a Shimadzu GC-2010 gas chromatograph with the mass detector GCMS QP2010. The used column is a quartz capillary column (30 m x 250 μm x 0.25 μm) HP-5 supplied by Agilent using hydrogen as the carrier gas. Analytical TLC: aluminum backed plates pre-coated (0.25 mm) with Merck Silica Gel 60 F254. Compounds were visualised by exposure to UV-light or by dipping the plates in permanganate ($KMnO_4$) stain followed by heating. Flash column chromatography was performed using Merck Silica Gel 60 (40–63 μm). All mixed solvent eluents are reported as v/v solutions.

### 1.1 Optical Measurements

UV/Vis absorption spectra were recorded on an Agilent Cary 8454 spectrophotometer or a JASCO V-770 in a 1 cm quartz cuvette. Fluorescence spectra were obtained on a JASCO FP-6200 or FS5 v2 Spectrofluorometer from Edinburgh Instruments. Fluorescence spectra were recorded using 5 nm excitation and 2 nm emission slit widths. More details can be found in the dedicated part of the Supplementary Information.

For Stern-Volmer quenching, a 1 cm High Precision Cell made of quartz from Hellma Analytics (117.100F-QS 10x10 mm, macro fluorescence sealable cuvette with an open cap with Silicone rubber seal, https://shop.hellmausa.com/products/117-100f-qs-10x10-mm?variant=45549332955389).

### 1.2 Photoreactors

All reactions except the ones carried out with Kessil lamps were conducted in 8 mL screw neck vials N15 with a screw cap fitted with a Silicone/PTFE septum from CBN Labsuppliers B.V. (article number: 702096; Vial N15-8, SC, c, 16.6x61, flat 8 mL and article number: 702180; SCB N15-H, blk, Sil w/PTFE bg, 45°).

All reactions except the ones carried out with Kessil lamps were conducted in Penn PhD Photoreactor M2 (https://www.sigmaaldrich.com/NL/en/product/sial/z744035) equipped with the corresponding wavelength light source. The parameters used were:

- 800 rpm stirring rate
- 6800 rpm fan rate
- 100% LEDs intensity (or the intensity stated in the experimental procedure)

A maximum of three reactions could be run in parallel inside a single photoreactor. Irradiating more than three at the same time led to decrease in yields. A 3D printed holder was used when more than one reaction was carried out in the photoreactor (see the part related to Pictures of Reaction Set-up).

All reactions with Kessil lamps were conducted in a 3D printed batch UFO photoreactor as the one described by the Noel group.[1] The 3D printing was carried out with the same materials as in the original article.

## 2 General Procedures

### General Procedure for the Azolation of benzene – GP1

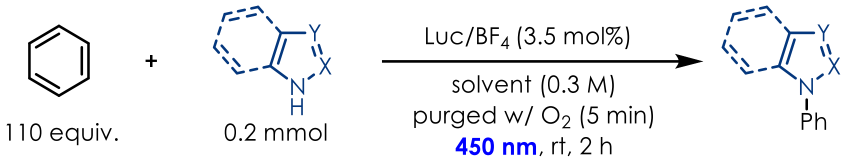


A dry 8 mL screw neck vial N15 equipped with a stirring bar was charged with the corresponding azole (0.2 mmol, 1.0 equiv.) if solid and **Luc/BF4** (3.5 mol%, 7 µmol, 3.9 mg). Under air, the corresponding dry solvent (0.65 mL, $c$ = 0.3 M) and dry benzene (2 mL, 22 mmol, 110 equiv.) were added. The vial was capped with a screw cap fitted with a Silicone/PTFE septum. The resulting solution was sparged with oxygen for 5 min (either with a 1 atm balloon or directly from an oxygen tank fitted with a pressure regulator). If liquid, the corresponding azole was added at this time (0.2 mmol, 1.0 equiv.). The vial was placed in the Penn PhD Photoreactor M2 equipped with a 450 nm light source. The parameters used for the irradiation are described in **part 1.2**. The mixture was stirred under irradiation for 2 h in most cases. Upon completion, the solvent was evaporated, and the residue was directly purified by column chromatography on silica gel to give the desired product. *Note: reactions can also be run with standard HPLC grade solvents without any decrease in yield*

**Table S1.** Main solvent variations used during the permutation reactions.

| GP | Solvent |
|---|---|
| **GP1a** | MeCN (0.3 M) |
| **GP1b** | HFIP (0.3 M) |

### General Procedure for the Azolation of Arenes – GP2

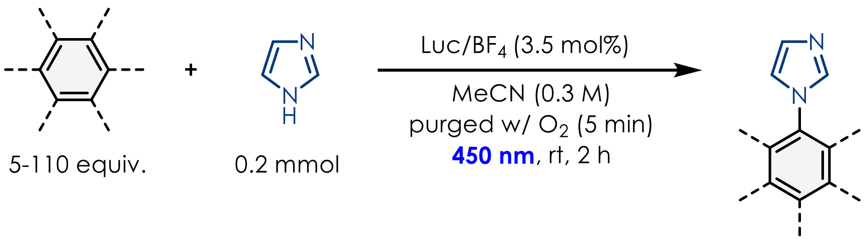


A dry 8 mL screw neck vial N15 equipped with a stirring bar was charged with pyrazole (13.6 mg, 0.2 mmol, 1.0 equiv.) and **Luc/BF4** (3.5 mol%, 7 µmol, 3.9 mg). Under air, dry

MeCN (0.65 mL, $c$ = 0.3 M) and the corresponding arene (5-110 equiv.) were added. The vial was capped with a screw cap fitted with a Silicone/PTFE septum. The resulting solution was sparged with oxygen for 5 min (either with a 1 atm balloon or directly from an oxygen tank fitted with a pressure regulator). The vial was placed in the Penn PhD Photoreactor M2 equipped with a 450 nm light source. The parameters used for the irradiation are described in **part 1.2**. The mixture was stirred under irradiation for 2 h in most cases. Upon completion, the solvent was evaporated, and the residue was directly purified by column chromatography on silica gel to give the desired product.

**General Procedure for the Azolation of benzene under "forcing" conditions – GP3**

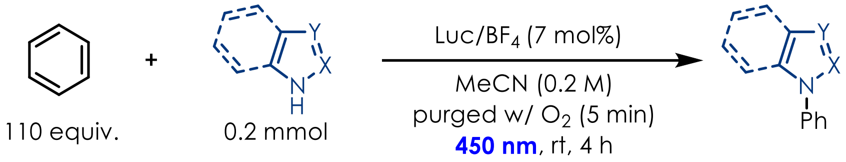


A dry 8 mL screw neck vial N15 equipped with a stirring bar was charged with the corresponding azole (0.2 mmol, 1.0 equiv.) if solid and **Luc/BF₄** (7 mol%, 14 µmol, 7.8 mg). Under air, dry MeCN (1 mL, $c$ = 0.2 M) and dry benzene (2 mL, 22 mmol, 110 equiv.) were added. The vial was capped with a screw cap fitted with a Silicone/PTFE septum. The resulting solution was sparged with oxygen for 5 min (either with a 1 atm balloon or directly from an oxygen tank fitted with a pressure regulator). If liquid, the corresponding azole was added at this time (0.2 mmol, 1.0 equiv.). The vial was placed in the Penn PhD Photoreactor M2 equipped with a 450 nm light source. The parameters used for the irradiation are described in **part 1.2**. The mixture was stirred under irradiation for 6 h in most cases. Upon completion, the solvent was evaporated, and the residue was directly purified by column chromatography on silica gel to give the desired product.

**General Procedure for the preparation of photocatalysts via salt exchange (anion metathesis) – GP4**

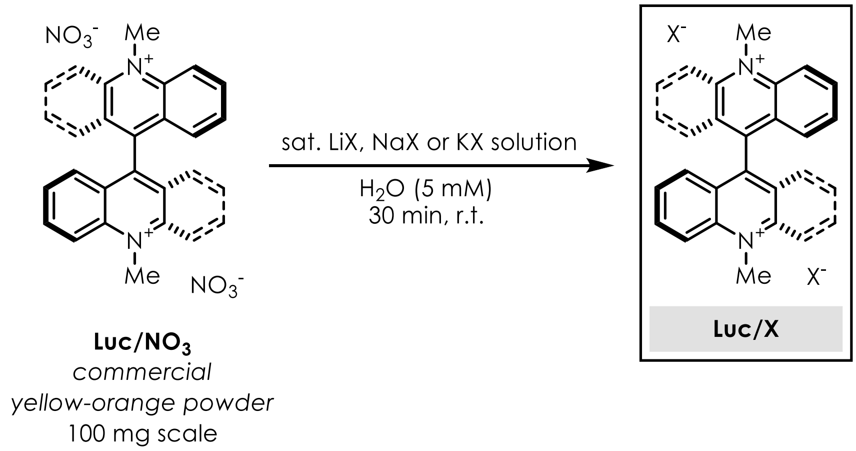


A dry round bottom flask equipped with a stirring bar was charged with Lucigenin (as nitrate salt, 0.2 mmol to 0.8 mmol, 1 equiv.). The solid was dissolved in distilled $H_2O$ ($c$ = 5-10 mM) and the flask was sonicated to ensure the full dissolution of Lucigenin. To this a saturated aqueous solution of the corresponding salt was added dropwise until no more precipitation was observed (*it corresponds to around 5 mL of the saturated aqueous solution*). The resulting solution was stirred 30 min at room temperature before collecting the precipitate on a fritted funnel and washing it with water (x 2), $Et_2O$ (x 2) and finally pentane. After drying under vacuum overnight, the corresponding product was obtained as a solid and it was stored under air in a screw neck brown vial.

# 3 Synthesis of the photocatalysts

## 10,10'-Dimethyl-[9,9'-biacridine]-10,10'-diium diperchlorate (Luc/$ClO_4$)

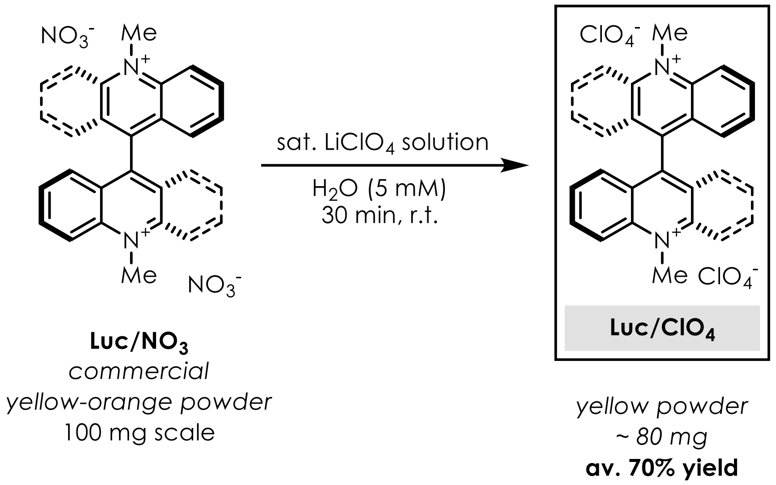


Following **GP4**, **Luc/$NO_3$** (100 mg, 0.2 mmol, 1.0 equiv.) and a saturated aqueous solution of lithium perchlorate gave **Luc/$ClO_4$** (70% average isolated yield, 80 mg) as a yellow powder.

**$^1$H NMR (400 MHz, DMSO-*d*$_6$)**: δ 9.05 (d, *J* = 9.4 Hz, 4H), 8.48 (t, *J* = 8.0 Hz, 4H), 7.76 (dd, *J* = 8.7, 6.6 Hz, 4H), 7.63 (d, *J* = 8.8 Hz, 4H), 5.12 (s, 6H).

**$^{13}$C NMR (151 MHz, DMSO-*d*$_6$):** δ 151.05, 141.71, 138.92, 128.89, 128.70, 126.52, 119.65, 40.06.

Data in accordance with the literature.[2]

## 10,10'-Dimethyl-[9,9'-biacridine]-10,10'-diium ditetrafluoroborate (Luc/$BF_4$)

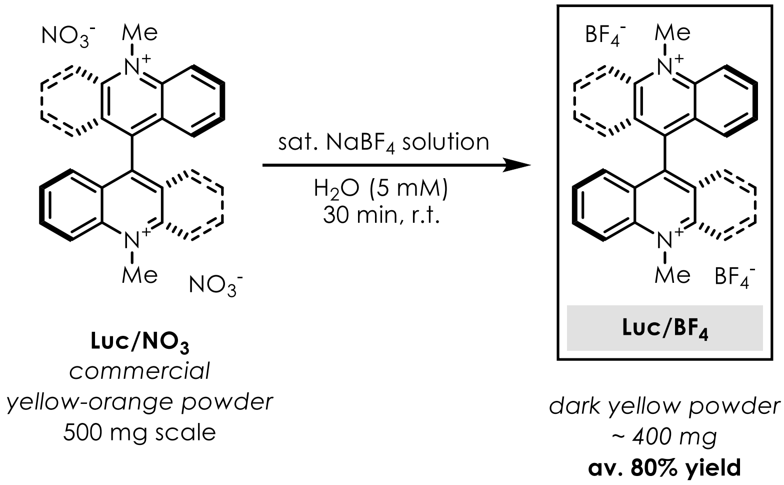

Following **GP4**, **Luc/NO3** (400 mg, 0.8 mmol, 1.0 equiv.) and a saturated aqueous solution of sodium tetrafluoroborate gave **Luc/BF4** (80% average isolated yield, 400 mg) as a dark yellow powder.

**$^1$H NMR (600 MHz, DMSO-*d6*):** δ 9.05 (d, *J* = 9.4 Hz, 4H), 8.53 – 8.43 (m, 4H), 7.81 – 7.72 (m, 4H), 7.63 (d, *J* = 8.7 Hz, 4H), 5.11 (s, 6H).

**$^{13}$C NMR (151 MHz, DMSO-*d6*):** δ 151.55, 142.21, 139.43, 129.41, 129.20, 127.03, 120.15, 40.06.

**$^{19}$F NMR (565 MHz, DMSO-*d6*):** δ -148.33 (s).

**$^{11}$B NMR (193 MHz, DMSO-*d6*):** δ -1.31

**10,10'-Dimethyl-[9,9'-biacridine]-10,10'-diium ditrifluoromethanesulfonate (Luc/OTf)**

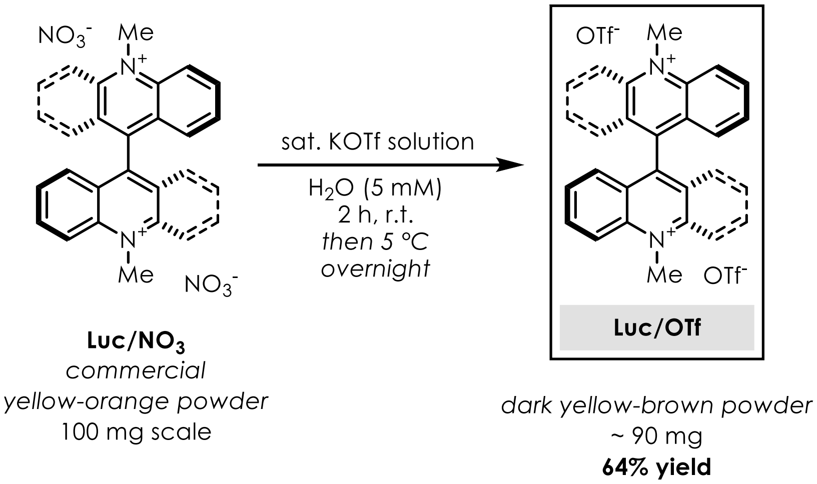


A dry round bottom flask equipped with a stirring bar was charged with **Luc/NO3** (100 mg, 0.2 mmol, 1.0 equiv.). The solid was dissolved in distilled $H_2O$ (30 mL, *c* = 7 mM) and the flask was sonicated to ensure the full dissolution of Lucigenin. To this an aqueous solution of potassium triflate (1 mmol in 2-3 mL of $H_2O$, 5 equiv.) was added dropwise. The resulting solution was stirred 2 h at room temperature and then it was cooled down to 5 °C overnight to force the precipitation. The precipitate was collected on a fritted funnel and washed with water (x 2), $Et_2O$ (x 2) and finally pentane. After drying under vacuum overnight, **Luc/OTf** was obtained as a dark yellow-brown powder (64% average isolated yield, 90 mg) and it was stored under air in a screw neck brown vial.

**$^1$H NMR (600 MHz, DMSO-*d$_6$*):** δ 9.05 (d, *J* = 9.3 Hz, 5H), 8.48 (s, 4H), 7.76 (t, *J* = 7.6 Hz, 5H), 7.63 (s, 4H), 5.11 (s, 6H).

**$^{19}$F NMR (565 MHz, DMSO-*d$_6$*):** δ -77.75 (s).

*<u>Note</u>: The peaks were too broad in $^{13}$C NMR to give a satisfactory NMR spectrum.*

### 10,10'-Dimethyl-[9,9'-biacridine]-10,10'-diium dihexafluorophosphate (Luc/$PF_6$)

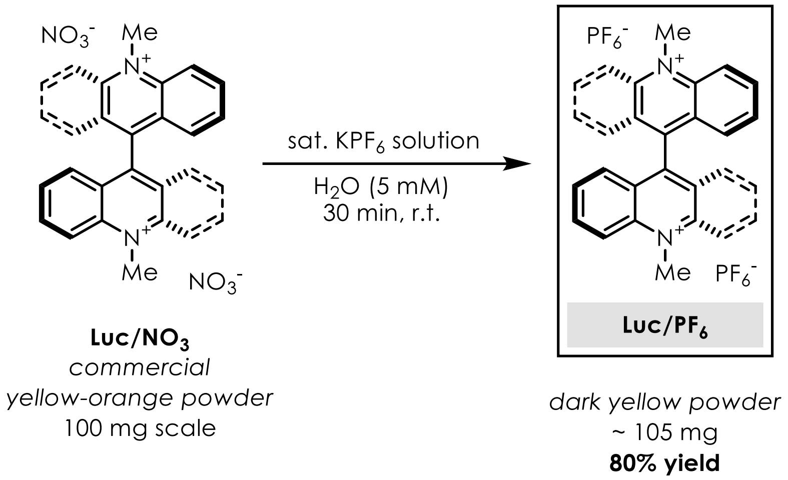


Following **GP4**, **Luc/$NO_3$** (100 mg, 0.2 mmol, 1.0 equiv.) and a saturated aqueous solution of potassium hexafluorophosphate gave **Luc/$PF_6$** (80% average isolated yield, 105 mg) as a dark yellow powder.

**$^1$H NMR (600 MHz, DMSO-*d$_6$*)** δ 9.06 (br s, 4H), 8.48 (br s, 4H), 7.76 (br s, 4H), 7.63 (br s, 4H), 5.11 (br s, 6H).

**$^{19}$F NMR (565 MHz, DMSO-*d$_6$*)** δ -70.18 (d, *J* = 710.7 Hz).

*<u>Note</u>: The peaks were too broad in $^{13}$C NMR to give a satisfactory NMR spectrum.*

**10,10'-Dimethyl-[9,9'-biacridine]-10,10'-diium bis[tetrakis(3,5-bis(trifluoromethyl)phenyl)borate)] (Luc/BAr$^F$$_4$)**

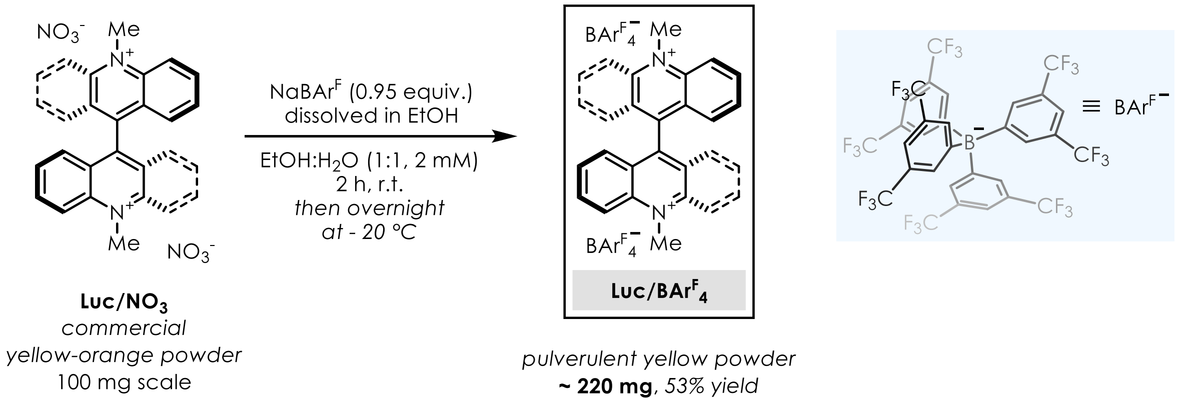


A dry round bottom flask equipped with a stirring bar was charged with **Luc/NO$_3$** (100 mg, 0.2 mmol, 1.0 equiv.). The solid was dissolved in distilled $H_2O$ (30 mL, *c* = 7 mM) and the flask was sonicated to ensure the full dissolution of Lucigenin. To this an ethanolic solution of tetrakis(3,5-bis(trifluoromethyl)phenyl)borate (0.19 mmol in the minimal amount of EtOH, 0.95 equiv.) was added dropwise. If necessary, more EtOH was added. The resulting solution was stirred vigorously for 2 h at room temperature and then it was cooled down to -20 °C overnight to force the precipitation. The precipitate was collected on a fritted funnel and washed with water (x 2), $Et_2O$ (x 2) and finally pentane. After drying under vacuum overnight, **Luc/BAr$^F$$_4$** was obtained as a pulverulent yellow powder (53% isolated yield, 220 mg) and it was stored under air in a screw neck brown vial.

**$^1$H NMR (600 MHz, DMSO-*d$_6$*):** δ 9.05 (br s, 4H), 8.49 (br s, 4H), 7.77 (br s, 4H), 7.72 (s, 8H), 7.62 (br s, 16H), 7.62 (br s, 4H), 5.12 (s, 6H).

**$^{13}$C NMR (151 MHz, DMSO-*d$_6$*):** δ 160.94 (q, $J_{C\text{-}B}$ = 49.9 Hz), 151.03, 141.74, 138.98, 134.05, 128.96, 128.73, 128.50 (q, $J_{C\text{-}F}$ = 32.6 Hz), 126.55, 124.02 (q, $J_{C\text{-}F}$ = 272.6 Hz), 119.70, 117.75, 40.06.

**$^{19}$F NMR (565 MHz, DMSO-*d$_6$*):** δ -61.62 (s).

**$^{11}$B NMR (193 MHz, DMSO-*d$_6$*):** δ -6.83.

**10,10'-Diphenyl-[9,9'-biacridine]-10,10'-diium dinitrate(A/$NO_3$)**

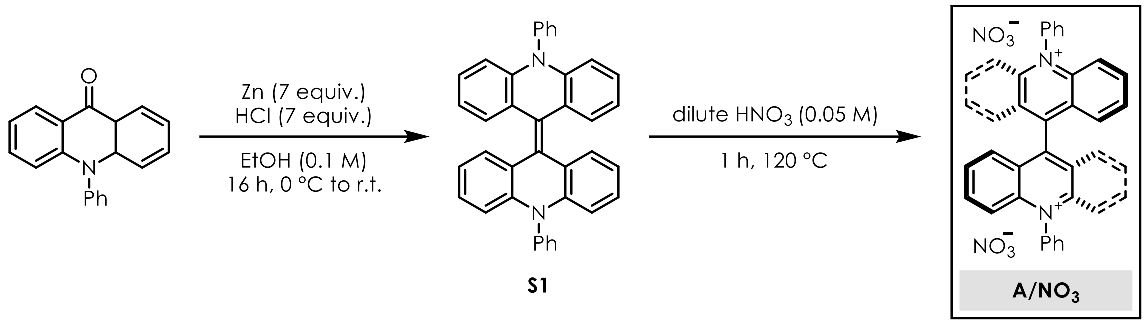


*orange-brown crystals*
**116 mg**, *15% overall yield*

**Step 1**: A dry round bottom flask equipped with a stirring bar was charged with *N*-phenylacridone (1.36 g, 5 mmol, 1.0 equiv.) and zinc powder (2.29 g, 35 mmol, 7 equiv.). The solid was dissolved in absolute EtOH (20 mL) and the resulting mixture was cooled down to 0°C with an ice-water bath. At 0 °C, a saturated ethanolic solution of hydrogen chloride (30 mL, $c$ = 1.25 M, 7 equiv.) was added dropwise. The cooling bath was then removed and the reaction mixture was stirred until most of the zinc was consumed. The reaction was terminated by pouring the mixture into water. The crude product was filtered off and rinsed well with water. Recrystallization from toluene gave the desired intermediate **S1** as a solid (33% isolated yield, 830 mg) and it was used directly in the next step.

**Step 2**: A dry round bottom flask equipped with a stirring bar was charged with **S1** (200 mg, 0.392 mmol, 1.0 equiv.). Dilute $HNO_3$ (50 mL, 2N) was added and the resulting mixture was heated for 1 h at 120 °C until most of the brownish solid was dissolved. The heating bath was then removed and undissolved by-products were filtered off. After cooling down to room temperature, orange to orange-brownish crystals appeared in the filtrate. The crystals were filtered off and dry under vacuum overnight, **A/$NO_3$** was obtained as orange to orange-brownish crystals (46% isolated yield, 116 mg) and it was stored under air in a screw neck brown vial.

**$^1$H NMR (600 MHz, DMSO-*d*$_6$):** δ 8.29 (br s, 4H), 8.15 – 7.85 (m, 15H), 7.84 – 7.66 (m, 7H).

**$^{13}$C NMR (151 MHz, DMSO-*d*$_6$)**: δ 152.78, 142.64, 139.54, 137.64, 132.39, 131.73, 129.57, 129.14, 128.11, 127.02, 120.60.

**10,10'-Diphenyl-[9,9'-biacridine]-10,10'-diium ditetrafluoroborate (A/$BF_4$)**

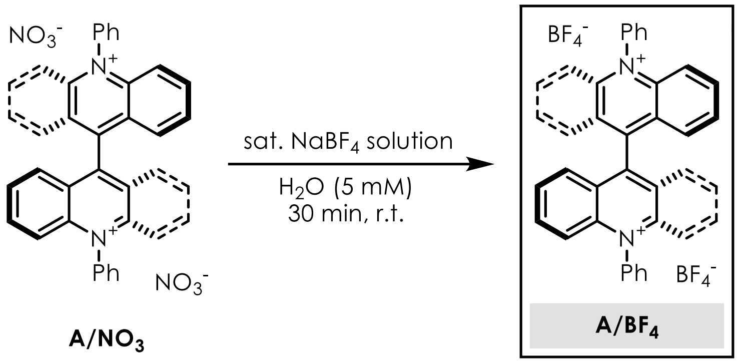


*dark yellow powder*
**av. 80% yield**

Following **GP4**, **A/$NO_3$** (100 mg, 0.16 mmol, 1.0 equiv.) and a saturated aqueous solution of sodium tetrafluoroborate gave **A/$BF_4$** (80% average isolated yield, 86 mg) as a dark yellow powder.

**$^1$H NMR (600 MHz, DMSO-*d*$_6$):** δ 8.29 (br s, 4H), 8.15 – 7.85 (m, 15H), 7.84 – 7.66 (m, 7H).

**$^{13}$C NMR (151 MHz, DMSO-*d*$_6$)**: δ 152.78, 142.64, 139.54, 137.64, 132.39, 131.73, 129.57, 129.14, 128.11, 127.02, 120.60.

**$^{19}$F NMR (565 MHz, DMSO-*d*$_6$):** δ -148.33 (s).

**$^{11}$B NMR (193 MHz, DMSO-*d*$_6$):** δ -1.31

# 4 Structure of Photocatalysts

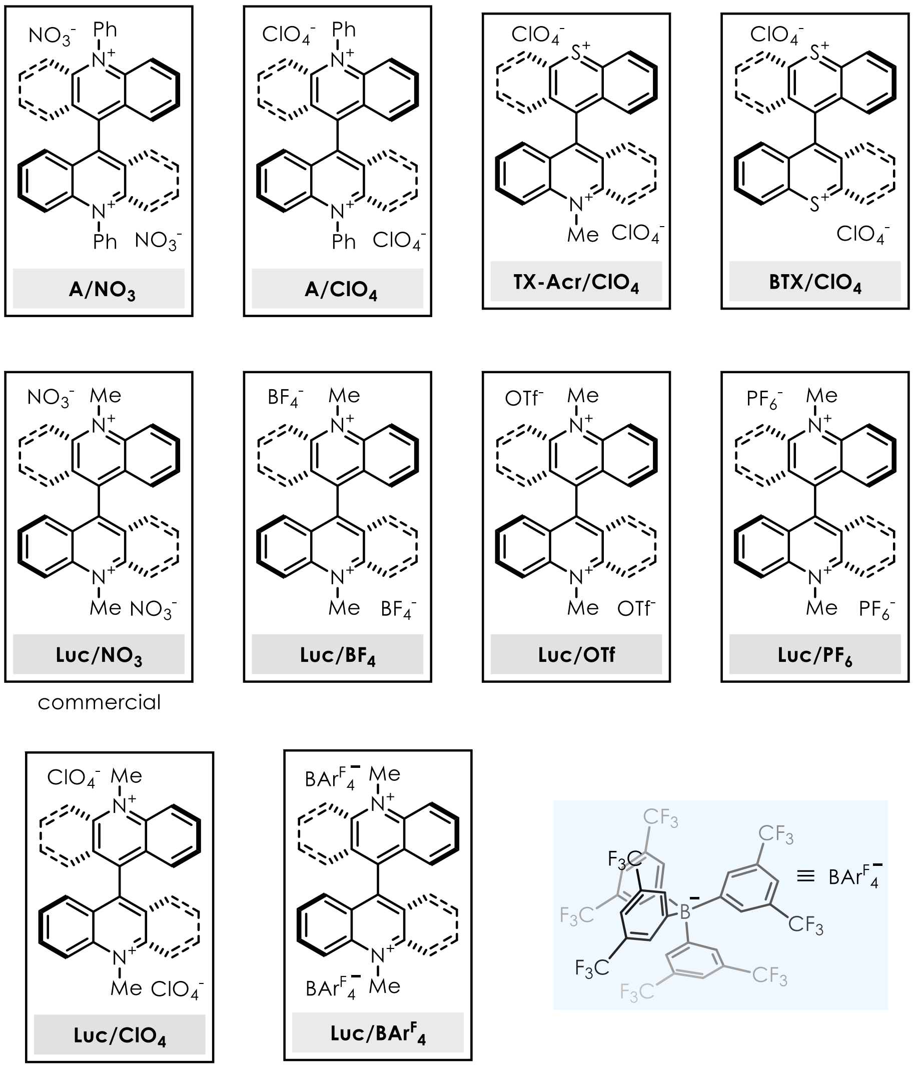

# 5 Structure of Starting Materials

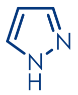

**2a**
commercial

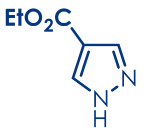

**3a**
commercial

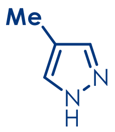

**4a**
commercial

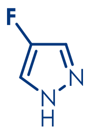

**5a**
commercial

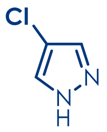

**6a**
commercial

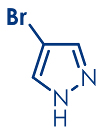

**7a**
commercial

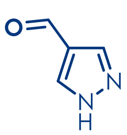

**8a**
commercial

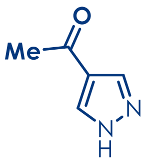

**9a**
commercial

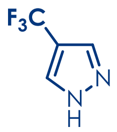

**10a**
commercial

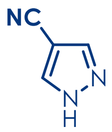

**11a**
commercial

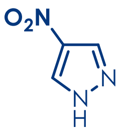

**12a**
commercial

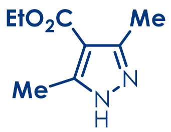

**13a**
commercial

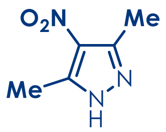

**14a**
commercial

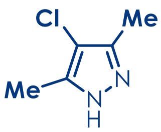

**15a**
commercial

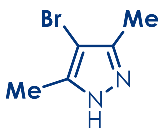

**16a**
commercial

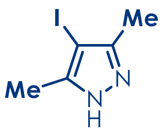

**17a**
commercial

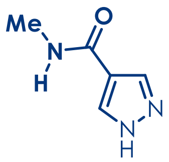

**18a**
commercial

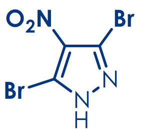

**19a**
commercial

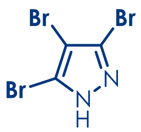

**20a**
commercial

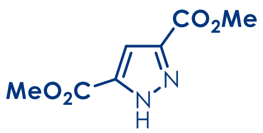

**21a**
commercial

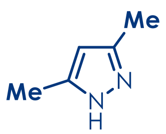

**22a**
commercial

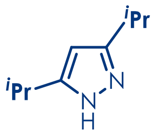

**23a**
commercial

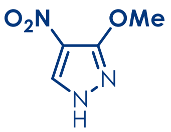

**24a**
commercial

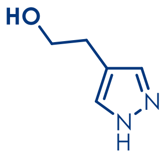

**25a**
commercial

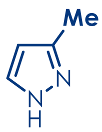

**S1a**
commercial

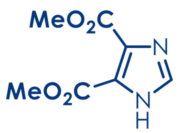

**26a**
commercial

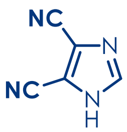

**27a**
commercial

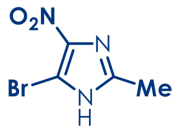

**28a**
commercial

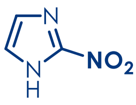

**29a**
commercial

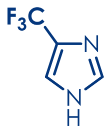

**S2a**
commercial

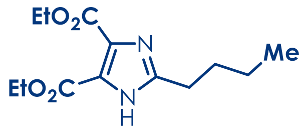

**S3a**
commercial

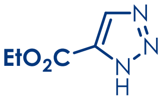


**30a**
commercial

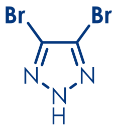


**31a**
commercial

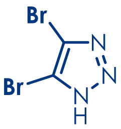


**32a**
commercial

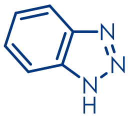


**33a**
commercial

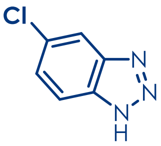


**34a**
commercial

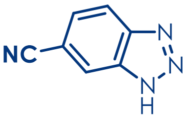


**35a**
commercial

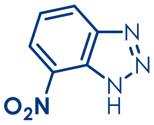


**36a**
commercial

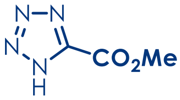


**37a**
commercial

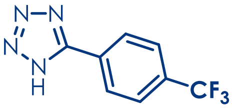


**38a**
commercial

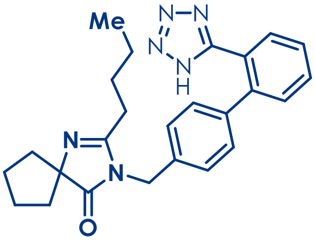


**39a**
commercial

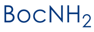


**51a**
commercial

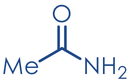


**52a**
commercial

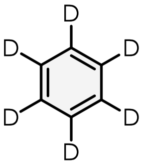


**40a**
commercial

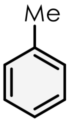


**41a**
commercial

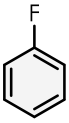


**42a**
commercial

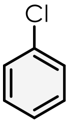


**43a**
commercial

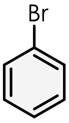


**44a**
commercial

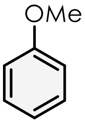


**45a**
commercial

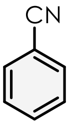


**46a**
commercial

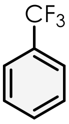


**47a**
commercial

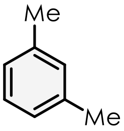


**48a**
commercial

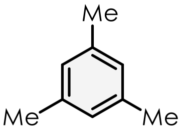


**49a**
commercial

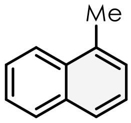


**50a**
commercial

# 6 Reaction Optimisation

## General Procedure for the Reaction Optimisation – GP5

A dry 8 mL screw neck vial N15 equipped with a stirring bar was charged with 1H-pyrazole (0.2 mmol, 1.0 equiv., 13.6 mg) and **Luc/BF$_4$** (3.5 mol%, 7 µmol, 3.9 mg). Under air, then dry acetonitrile (0.65 mL, $c$ = 0.3 M) and dry benzene (2 mL, 22 mmol, 110 equiv.) were added. The vial was capped with a screw cap fitted with a Silicone/PTFE septum. The resulting solution was sparged with oxygen for 5 min (either with a 1 atm balloon or directly from an oxygen tank fitted with a pressure regulator). The vial was placed in the Penn PhD Photoreactor M2 equipped with a 450 nm light source. The parameters used for the irradiation are described in **part 1.2**. The mixture was stirred under irradiation for 2 h unless otherwise noted. Upon completion, 0.5 mL of 1,3-dinitrobenzene stock solution in ethyl acetate (0.4 M) is added to the crude mixture, which is then diluted with MeOH to solubilise mixture. Then an aliquot of the mixture is concentrated under vacuum, the residue is diluted with MeOH-$d_4$ and analysed by $^1$H NMR spectroscopy to determine the NMR yield.

## 6.1 Influence of photocatalyst nature and counterion

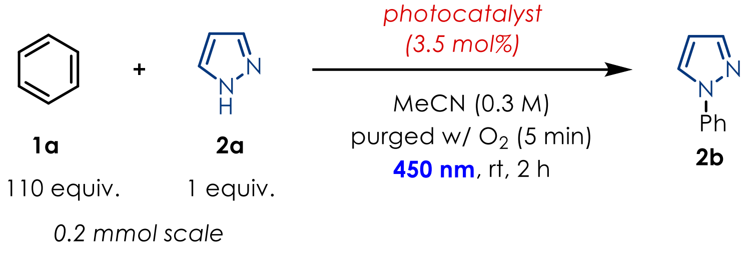


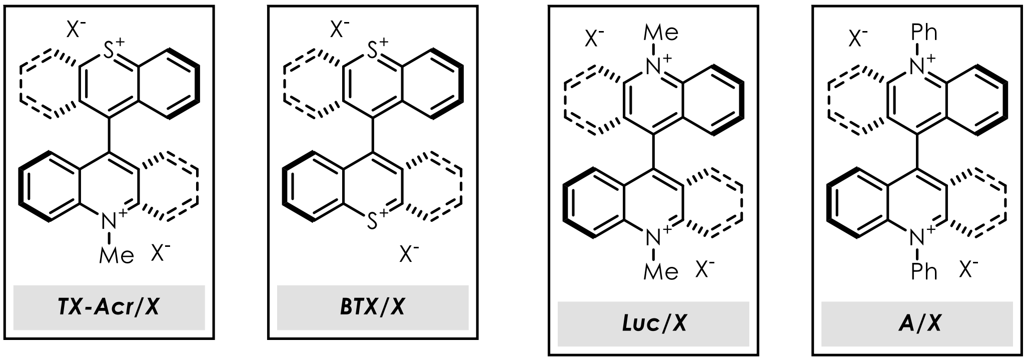


All the reaction parameters were optimised following **GP5** using **2a** (13.6 mg, 0.2 mmol, 1.0 equiv.) and 3.5 mol% of photocatalyst (PC).

**Table S2.** Screening of photocatalysts and different counter ions.

| entry | PC | 2a (%) | 2b (%) |
|---|---|---|---|
| **1** | **TX-Acr/$ClO_4$** | 29 | 10 |
| **2** | **BTX/$ClO_4$** | 57 | n.d. |
| **3** | **Luc/$ClO_4$** | 31 | 29 |
| **4** | **Luc/$NO_3$** | 44 | 2 |
| **5** | **Luc/$BF_4$** | 7/8/5 | **71/71/66** |
| **6** | **Luc/$PF_6$** | 17 | 58 |
| **7** | **Luc/OTf** | 7 | 66 |
| **8** | **Luc/$BAr^F_4$** | - | 11 |
| **9** | **A/$NO_3$** | 71 | n.d. |
| **10** | **A/$BF_4$** | 43 | 12 |

## 6.2 Influence of photocatalyst loading

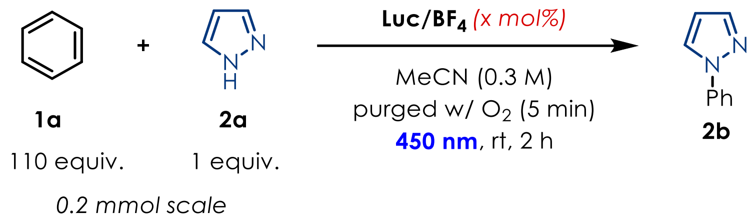


All the reaction parameters were optimised following **GP5** using **2a** (13.6 mg, 0.2 mmol, 1.0 equiv.) and **Luc/BF$_4$** as photocatalyst.

**Table S3.** Screening of photocatalyst loading.

| entry | PC loading (mol%) | 2a (%) | 2b (%) |
|---|---|---|---|
| **1** | 1 | 23 | 54 |
| **2** | 2 | 9 | 64 |
| **3** | 3 | 11 | 67 |
| **4** | 3.5 | 9 | **68** |
| **5** | 5 | 8 | 65 |
| **6** | 10 | 16 | 61 |

## 6.3 Influence of irradiation wavelength and intensity

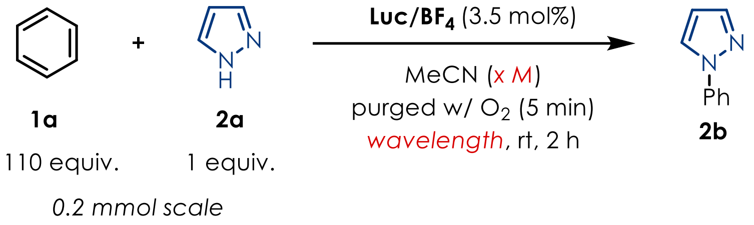


All the reaction parameters were optimised following **GP5** using **2a** (13.6 mg, 0.2 mmol, 1.0 equiv.) and 3.5 mol% of **Luc/BF$_4$** as photocatalyst.

**Table S4.** Screening of irradiation wavelength and intensity in Penn PhD Photoreactor M2.

| entry | concentration (M) | λ (nm) | intensity (%) | time (h) | 2a (%) | 2b (%) |
|---|---|---|---|---|---|---|
| **1** | 0.1 | 450 | 100 | 2 | 11 | 45 |
| **2** | | 420 | 100 | 2 | 13 | 39 |
| **3** | | 395 | 100 | 2 | 12 | 49 |
| **4** | | 365 | 100 | 2 | 22 | 30 |
| **5** | 0.4 | 450 | 25 | 2 | 44 | 34 |
| **6** | | | 35 | 2 | 39 | 37 |
| **7** | | | 50 | 2 | 26 | 58 |
| **8** | | | 60 | 2 | 24 | 58 |
| **9** | | | 75 | 2 | 13 | 63 |
| **10** | | | 100 | 2 | 21 | 65 |
| **11** | 0.3 | 450 | 50 | 4 | <5 | **72** |
| **12** | | | 100 | 2 | 7 | **71** |

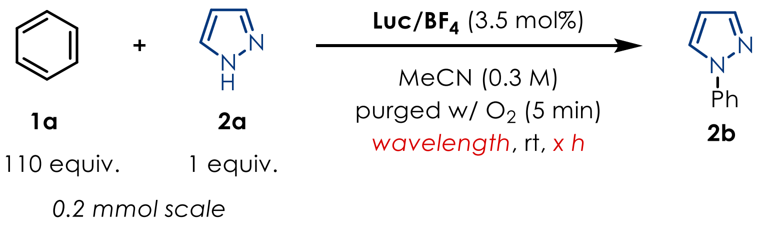


**Table S5.** Screening of irradiation wavelength, intensity and time in Kessil setup at 0.3 M in MeCN.

| entry | wavelength (nm) | intensity (%) | time (h) | 2a (%) | 2b (%) |
|---|---|---|---|---|---|
| **1** | 440 | 100 | 4 | <5 | 61 |
| **2** | | 50 | 8 | <5 | 67 |
| **3** | 427 | 100 | 4 | 10 | 64 |
| **5** | 390 | 100 | 2 | 16 | 63 |
| **6** | | 100 | 3 | 11 | 63 |
| **7** | | 50 | 4 | 23 | 63 |
| **8** | | 50 | 8 | 10 | 70 |
| **9** | | 50 | 12 | 8 | **75** |
| **10** | | 50 | 16 | 4 | 70 |
| **11** | 370 | 100 | 2 | 16 | 63 |

## 6.4 Influence of atmosphere

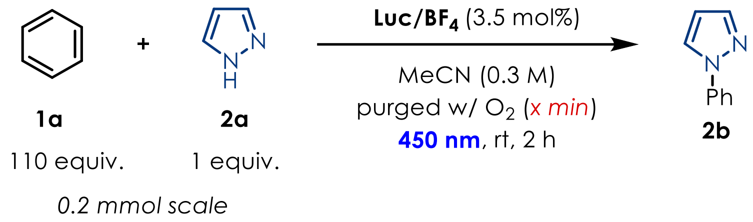


All the reaction parameters were optimised following **GP5** using **2a** (13.6 mg, 0.2 mmol, 1.0 equiv.) and **Luc/BF₄** as photocatalyst, while varying the oxidant purging times.

**Table S6.** Screening of oxidant purging times.

| entry | time (min) | 2a (%) | 2b (%) |
|---|---|---|---|
| **1** | - | 67 | 8 |
| **2** | 2 | 6 | 70 |
| **3** | 5 | 5 | 64 |
| **4** | 10 | 9 | 68 |
| **5** | 20 | 6 | 69 |
| **6** | 30 | 6 | 68 |

## 6.5 Influence of benzene loading

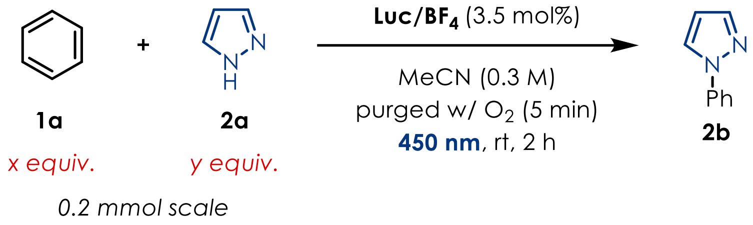


All the reaction parameters were optimised following **GP5** using **2a** (13.6 mg, 0.2 mmol, 1.0 equiv.) and **Luc/BF$_4$** as photocatalyst, while varying the ratios between reagents.

**Table S7.** Screening of equivalent ratios between reagents. [a]Solvents were purged with $O_2$ before adding to vial.

| entry | 1a:2a | 2a (%) | 2b (%) |
|---|---|---|---|
| **1** | 220:1 | 55 | 25 |
| **2** | 110:1 | 9 | **68** |
| **3** | 55:1 | 20 | 49 |
| **4**[a] | 27.5:1 | 19 | 37 |
| **5**[a] | 13.75:1 | 18 | 38 |
| **6**[a] | 5:1 | 28 | 29 |
| **7**[a] | 2:1 | 33 | 16 |
| **8**[a] | 1:1 | 51 | 9 |
| **9**[a] | 1:2 | 71 | 7 |
| **10**[a] | 1:5 | 84 | <5 |

### 6.6 Influence of concentration

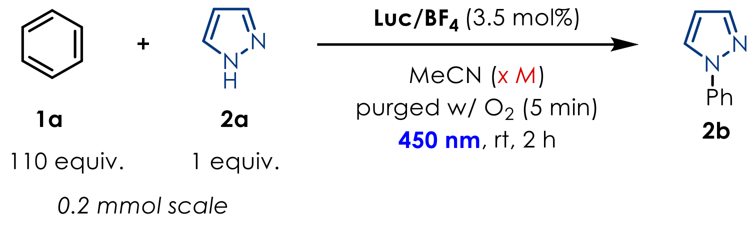


All the reaction parameters were optimised following **GP5** using **2a** (13.6 mg, 0.2 mmol, 1.0 equiv.) and **Luc/BF₄** as photocatalyst in MeCN, while varying concentration.

**Table S8.** Screening of concentration in MeCN as solvent.

| entry | concentration (M) | 2a (%) | 2b (%) |
|---|---|---|---|
| **1** | 0.05 | 18 | 25 |
| **2** | 0.1 | 12 | 39 |
| **3** | 0.2 | 10 | 45 |
| **4** | 0.25 | <5 | 66 |
| **5** | 0.3 | 7/8/5 | **71/71/66** |
| **6** | 0.35 | 14 | 63 |
| **7** | 0.4 | 8 | 60 |
| **8** | 0.5 | 42 | 42 |
| **9** | 0.6 | 65 | 19 |
| **10** | *neat* | 73 | <5 |

## 6.7 Influence of solvent

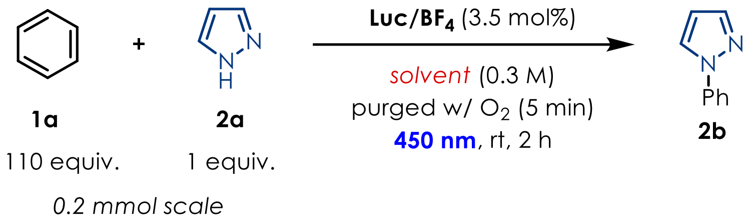


All the reaction parameters were optimised following **GP5** using **2a** (13.6 mg, 0.2 mmol, 1.0 equiv.) and **Luc/BF₄** as photocatalyst, while varying solvent at 0.3 M concentration.

**Table S9.** Screening of solvents using the optimal 0.3 M concentration.

| entry | solvent | 2a (%) | 2b (%) |
|---|---|---|---|
| **1** | THF | 75 | n.d. |
| **2** | dioxane | 83 | n.d. |
| **3** | acetone | 75 | 3 |
| **4** | EtOH | 41 | 1 |
| **5** | EtOAc | 58 | n.d. |
| **6** | MeOH | 35 | 8 |
| **7** | *i*-PrOH | 84 | n.d. |
| **8** | DMSO | 100 | n.d. |
| **9** | DCE | 68 | n.d. |
| **10** | DMF | 91 | n.d. |
| **11** | $CH_2Cl_2$ | 80 | n.d. |
| **12** | EtOH:MeCN (1:9) | 10 | 57 |
| **13** | DMSO:MeCN (1:9) | 50 | 30 |
| **14** | acetone:MeCN (1:9) | 5 | 68 |
| **15** | $H_2O$:MeCN (1:9) | 25 | 39 |
| **16** | TFE:HFIP (1:9) | 30 | 43 |
| **17** | TFE | 27 | 56 |
| **18** | HFIP | 17 | **84** |
| **19** | MeCN | 7/8/5 | **71/71/66** |

## 6.8 Influence of additives (including bases)

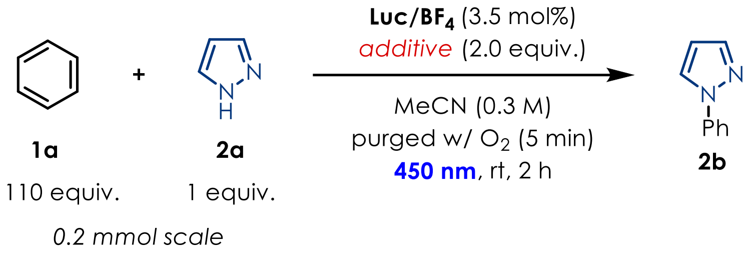


All the reaction parameters were optimised following **GP5** using **2a** (13.6 mg, 0.2 mmol, 1.0 equiv.) and **Luc/BF$_4$** as photocatalyst in MeCN, while varying the additives used.

**Table S10.** Screening of additives.

| entry | additive | 2a (%) | 2b (%) |
|---|---|---|---|
| **1** | none | 9 | **68** |
| **2** | DBU | 70 | n.d. |
| **3** | BTMG | 68 | n.d. |
| **4** | pyridine | 57 | <5 |
| **5** | 2,6-lutidine | 64 | <5 |
| **6** | $Cs_2CO_3$ | 90 | n.d. |
| **7** | $K_3PO_4$ | 18 | n.d. |
| **8** | $K_2HPO_4$ | 43 | 31 |
| **9** | 2,2,6,6-TMP | 67 | n.d. |
| **10** | 2,6-dimethylaniline | 50 | 17 |
| **11** | $NaHCO_3$ | 28 | 44 |
| **12** | NaOAc | 62 | 6 |
| **13** | $ZnCl_2$ | 79 | 8 |

DBU = 1,8-Diazabicyclo(5.4.0)undec-7-ene; BTMG = 2-*tert*-Butyl-1,1,3,3-tetramethylguanidine

2,2,6,6-TMP = 2,2,6,6-tetramethylpiperidine

# 7 Pictures of Reaction Set-up

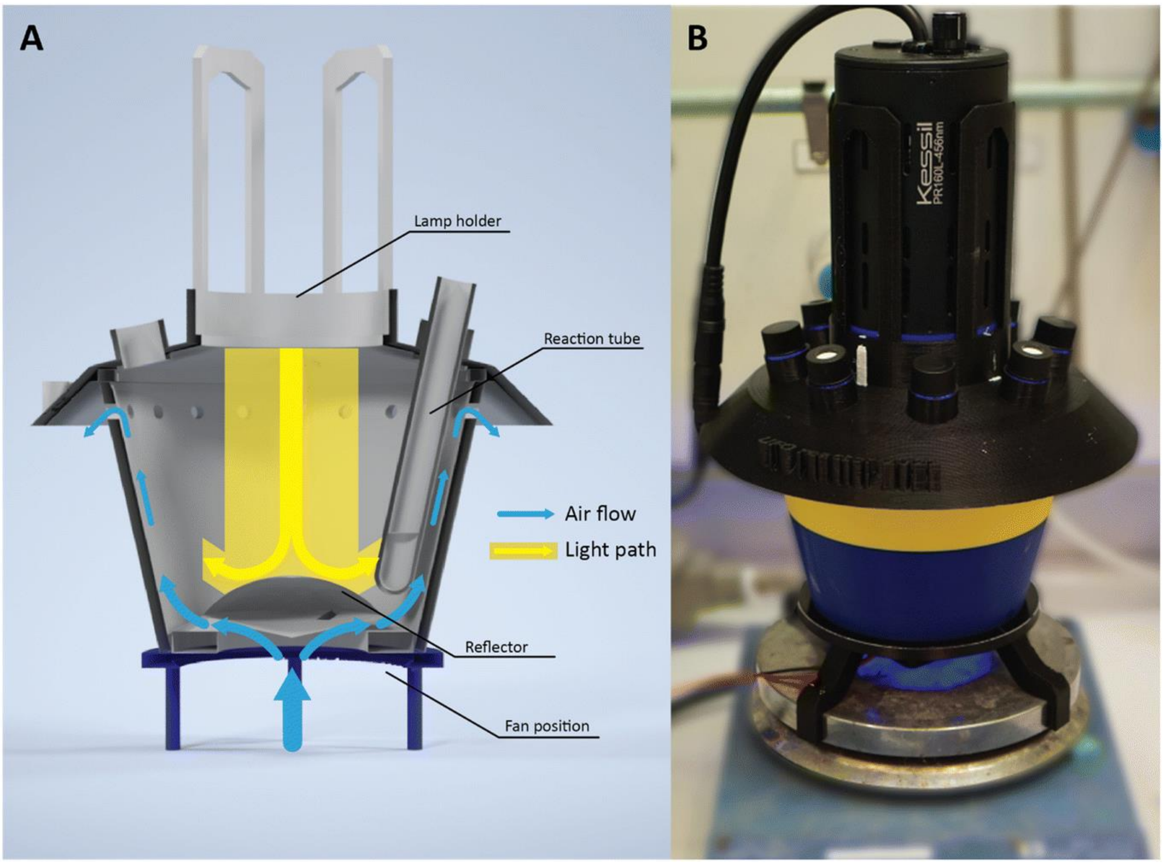


**Figure S1.** A) Sliced schematic design of the UFO batch reactor system with air flow and light path directions indicated. B) Assembled 8-vial UFO reactor being operated, mounted on a custom clamp support to fit IKA magnetic stirring plates and a Kessil lamp on top. Adapted from *React. Chem. Eng.*, **2024**, *9*, 2218-2225 (ref. 1).

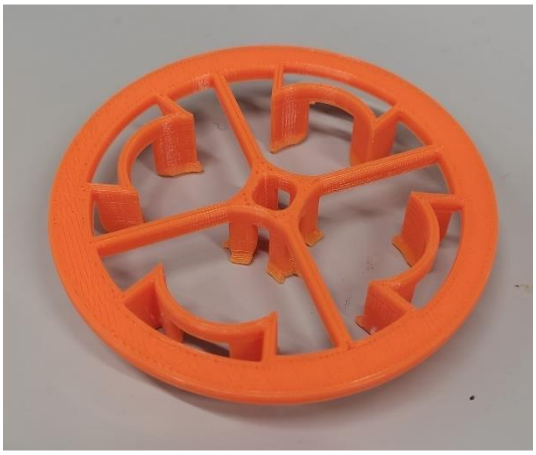

**Figure S2.** 3D-printed holder for the Penn photoreactor M2 fitting 4 vials maximum.

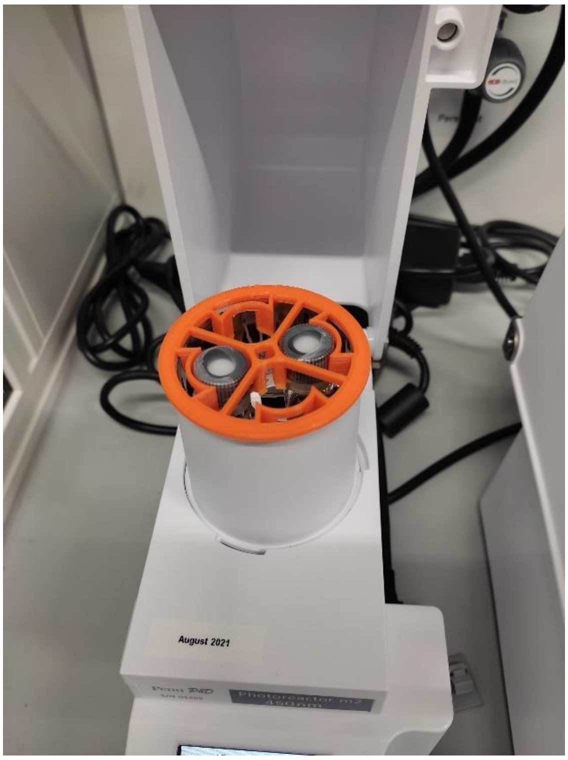


**Figure S3.** Picture of the reaction setup for two reaction vials in the Penn photoreactor M2 equipped with a 450 nm light source.

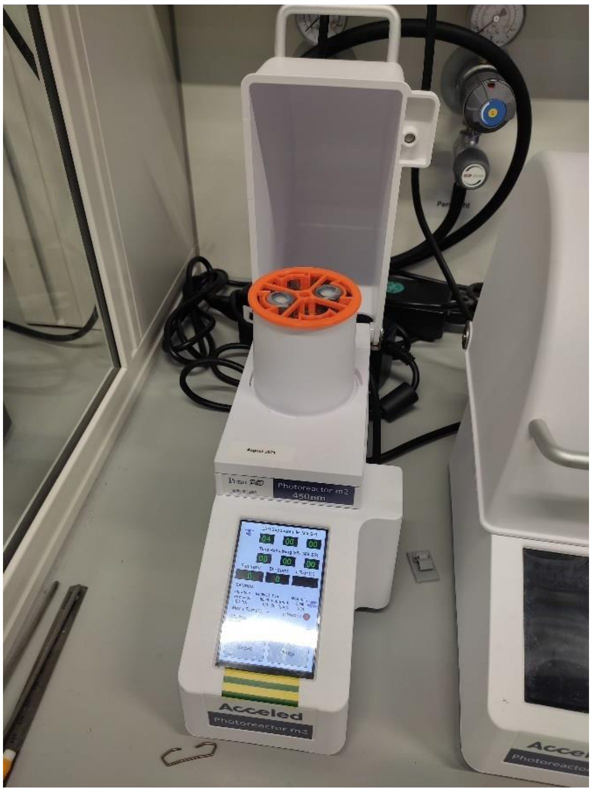


**Figure S4** Additional picture of the reaction setup for two reaction vials in the Penn photoreactor M2 equipped with a 450 nm light source.

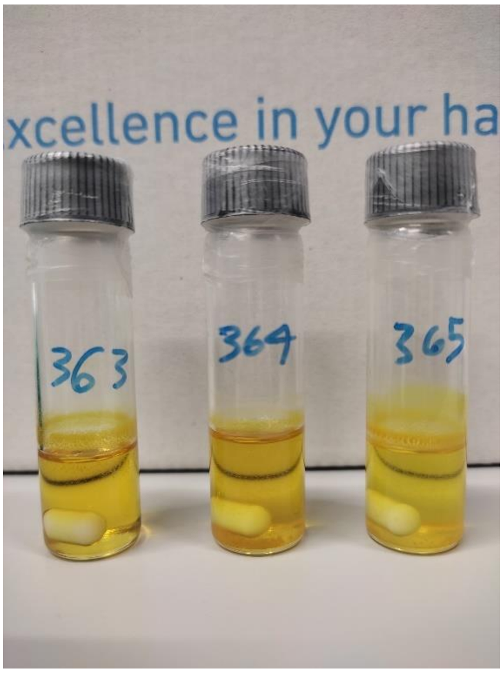

**Figure S5.** Typical reaction vial used for the irradiation. It is sealed with parafilm to keep the oxygen pressure inside relatively constant.

## 8 Scope

*Note: All the products were purified using the following column conditions: $SiO_2$ (5 cm), ∅ = 3 cm, tubes (16 mm x 100 mm). The eluent used for each substrate is described below separately.*

**1-Phenyl-1*H*-pyrazole (2b)**

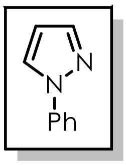


Following **GP1a**, **2a** (13.6 mg, 0.2 mmol, 1.0 equiv.) gave **2b** (69% NMR yield, 6% rsm; 69% isolated yield, 20.0 mg) as a light-yellow oil. Note: *this yield is reported as averages of five experiments on a 0.2 mmol scale.*

**$R_f$** 0.73 [pentane:EtOAc (9:1)].

**Column eluent:** 100 mL pentane:EtOAc (19:1) → 100 mL pentane:EtOAc (9:1).

**$^1$H NMR (600 MHz, $CDCl_3$):** 7.93 (dd, $J$ = 2.5, 0.6 Hz, 1H), 7.73 (d, $J$ = 1.8 Hz, 1H), 7.72 – 7.68 (m, 2H), 7.49 – 7.43 (m, 2H), 7.32 – 7.26 (m, 1H), 6.47 (dd, $J$ = 2.4, 1.8 Hz, 1H).

**$^{13}$C NMR (151 MHz, $CDCl_3$):** δ 141.21, 140.34, 129.56, 126.87, 126.57, 119.35, 107.72.

Data in accordance with the literature.[3]

Following **GP1b**, **2a** (13.9 mg, 0.2 mmol, 1.0 equiv.) gave **2b** (84% NMR yield, 10% rsm) as a light-yellow oil.

**Ethyl 1-Phenyl-1*H*-pyrazole-4-carboxylate (3b)**

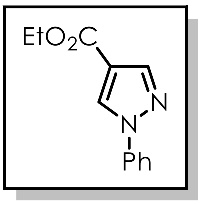


Following **GP1a**, **3a** (28 mg, 0.2 mmol, 1.0 equiv.) gave **3b** (75% NMR yield; 85% isolated yield, 36.7 mg) as white solid.

**$R_f$** 0.86 [pentane:EtOAc (8:2)]

**Column eluent:** 200 mL pentane:EtOAc (9:1)

**$^1$H NMR (600 MHz, CDCl$_3$):** δ 8.40 (d, *J* = 0.6 Hz, 1H), 8.09 (s, 1H), 7.73 – 7.67 (m, 2H), 7.49 – 7.44 (m, 2H), 7.34 (td, *J* = 7.3, 1.2 Hz, 1H), 4.33 (q, *J* = 7.1 Hz, 2H), 1.37 (t, *J* = 7.1 Hz, 3H).

**$^{13}$C NMR (151 MHz, CDCl$_3$):** δ 162.95, 142.28, 139.52, 130.11, 129.70, 127.63, 119.69, 117.04, 60.54, 14.50.

Data in accordance with the literature.[3,4]

### 4-Methyl-1-phenyl-1*H*-pyrazole (4b)

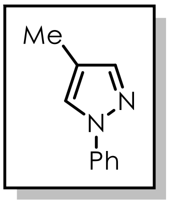


Following **GP1a**, but irradiating the reaction mixture for 4 h, **4a** (16.5 μL mg, 0.2 mmol, 1.0 equiv.) gave **4b** (36% NMR yield, 16% rsm; 37% isolated yield, 11.6 mg) as a light-yellow oil.

**R*f*** 0.55 [pentane:EtOAc (9:1)]

**Column eluent:** 200 mL pentane:EtOAc (19:1).

**$^1$H NMR (600 MHz, CDCl$_3$):** δ 7.72 – 7.68 (m, 1H), 7.65 (dd, *J* = 8.7, 1.2 Hz, 2H), 7.53 (s, 1H), 7.46 – 7.39 (m, 2H), 7.29 – 7.22 (m, 1H), 2.25 – 2.07 (m, 3H).

**$^{13}$C NMR (151 MHz, CDCl$_3$):** δ 141.93, 140.42, 129.49, 126.11, 125.48, 118.86, 118.35, 9.10.

Data in accordance with the literature.[3]

**4-Fluoro-1-phenyl-1*H*-pyrazole (5b)**

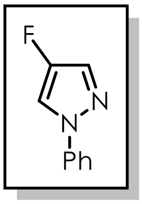


Following **GP1a**, **5a** (17.2 mg, 0.2 mmol, 1.0 equiv.) gave **5b** (40% NMR yield, 30% rsm; 40% isolated yield, 13.0 mg) as light-yellow oil.

**R*f*** 0.63 [pentane:EtOAc (9:1)]

**Column eluent:** 200 mL pentane:EtOAc (97:3).

**$^{1}$H NMR (600 MHz, CDCl$_3$):** δ 7.80 (d, *J* = 4.7 Hz, 1H), 7.65 – 7.60 (m, 2H), 7.58 (d, *J* = 4.1 Hz, 1H), 7.49 – 7.42 (m, 2H), 7.33 – 7.28 (m, 1H).

**$^{13}$C NMR (151 MHz, CDCl$_3$):** δ 151.29 (d, *J* = 248.9 Hz), 140.25, 129.63, 128.62 (d, *J* = 13.6 Hz), 126.87, 118.86, 113.10 (d, *J* = 28.3 Hz).

**$^{19}$F NMR (565 MHz, CDCl$_3$):** δ -175.03 (s).

Data in accordance with the literature.[5]

**4-Chloro-1-phenyl-1*H*-pyrazole (6b)**

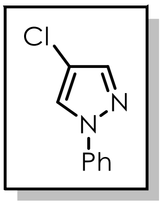


Following **GP1a**, but irradiating the reaction mixture for 4 h, **6a** (20.5 mg, 0.2 mmol, 1.0 equiv.) gave **6b** (51% NMR yield 23% rsm; 51% isolated yield, 19.2 mg) as a white solid.

**R*f*** 0.76 [pentane:EtOAc (19:1)].

**Column eluent:** 200 mL pentane:EtOAc (97:3).

**$^{1}$H NMR (600 MHz, CDCl$_3$):** δ 7.91 (s, 1H), 7.64 (s, 1H), 7.64 (d, *J* = 8.0 Hz, 2H), 7.46 (dd, *J* = 8.6, 7.4 Hz, 1H), 7.35 – 7.29 (m, 1H).

**$^{13}$C NMR (151 MHz, CDCl$_3$):** δ 139.87, 139.61, 129.68, 127.14, 124.96, 119.11, 112.53.

Data in accordance with the literature.[3]

**4-Bromo-1-phenyl-1*H*-pyrazole (7b)**

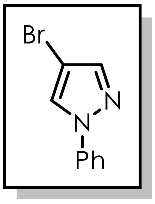


Following **GP1a**, but irradiating the reaction mixture for 4 h, **7a** (29.4 mg, 0.2 mmol, 1.0 equiv.) gave **7b** (35% NMR yield, 36% rsm; 35% isolated yield, 15.6 mg) as a white solid.

**R*f*** 0.71 [pentane:EtOAc (9:1)]

**Column eluent:** 200 mL pentane:EtOAc (97:3).

**$^{1}$H NMR (600 MHz, CDCl$_3$):** δ 7.94 (s, 1H), 7.67 (s, 1H), 7.66 – 7.61 (m, 2H), 7.49 – 7.43 (m, 2H), 7.35 – 7.30 (m, 1H).

**$^{13}$C NMR (151 MHz, CDCl$_3$):** δ 141.66, 139.78, 129.69, 127.19, 127.16, 119.17, 95.77.

Data in accordance with the literature.[3]

**1-Phenyl-1*H*-pyrazole-4-carbaldehyde (8b)**

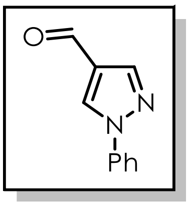


Following **GP1a**, **8a** (36.8 mg, 0.2 mmol, 1.0 equiv.) gave **8b** (52% isolated yield, 17.9 mg) as an orange solid.

**R*f*** 0.12 [hexane:EtOAc (1:1)].

**Column eluent:** 150 mL pentane:$CH_2Cl_2$ (1:1) → 300 mL pentane:EtOAc (9:1).

**$^{1}$H NMR (600 MHz, CDCl$_3$):** δ 9.96 (s, 1H), 8.44 (s, 1H), 8.17 (s, 1H), 7.74 – 7.68 (m, 2H), 7.53 – 7.47 (m, 2H), 7.41 – 7.36 (m, 1H).

**$^{13}$C NMR (151 MHz, CDCl$_3$):** δ 184.22, 141.83, 139.24, 130.13, 129.84, 128.15, 125.78, 119.95.

Data in accordance with the literature.[3]

**1-(1-Phenyl-1*H*-pyrazol-4-yl)ethan-1-one (9b)**

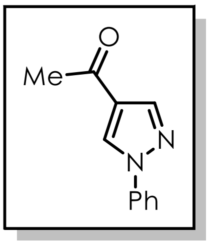

Following **GP1a**, **9a** (22.0 mg, 0.2 mmol, 1.0 equiv.) gave **9b** (70% NMR yield, <5% rsm; 70% isolated yield, 26.2 mg) as a white solid.

**R*f*** 0.10 [pentane:EtOAc (9:1)].

**Column eluent:** 100 mL pentane:EtOAc (9:1) → 250 mL pentane:EtOAc (8:2).

**$^1$H NMR (600 MHz, CDCl$_3$):** δ 8.39 (s, 1H), 8.09 (s, 1H), 7.70 (dd, *J* = 7.6, 1.2 Hz, 2H), 7.52 – 7.44 (m, 2H), 7.36 (td, *J* = 7.4, 1.3 Hz, 1H), 2.50 (d, *J* = 1.1 Hz, 3H).

**$^{13}$C NMR (151 MHz, CDCl$_3$):** δ 192.17, 141.65, 139.40, 129.76, 129.18, 127.86, 125.73, 119.82, 28.15.

Data in accordance with the literature.[3]

**1-Phenyl-4-(trifluoromethyl)-1*H*-pyrazole (10b)**

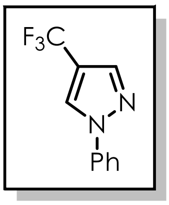

Following **GP1a**, **10a** (27.2 mg, 0.2 mmol, 1.0 equiv.) gave **10b** (66% NMR yield, 9% rsm; 66% isolated yield, 28.1 mg) as a white-solid.

**R*f*** 0.76 [pentane:EtOAc (19:1)].

**Column eluent:** 200 mL pentane:EtOAc (97:3).

**$^1$H NMR (600 MHz, CDCl$_3$):** δ 8.18 (s, 1H), 7.91 (s, 1H), 7.71 – 7.65 (m, 2H), 7.49 (dd, *J* = 8.5, 7.4 Hz, 2H), 7.41 – 7.34 (m, 1H).

**$^{13}$C NMR (151 MHz, CDCl$_3$):** δ 139.43, 138.33 (q, *J* = 2.7 Hz), 129.79, 127.90, 126.43 (q, *J* = 3.6 Hz), 122.60 (q, *J* = 266.1 Hz), 119.89, 115.61 (q, *J* = 38.6 Hz).

$^{19}$**F NMR (565 MHz, CDCl$_3$):** δ -56.69 (s).

Data in accordance with the literature.[3]

### 1-Phenyl-1*H*-pyrazole-4-carbonitrile (11b)

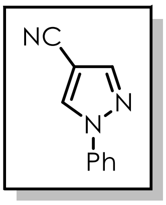


Following **GP1a**, **11a** (18.6 mg, 0.2 mmol, 1.0 equiv.) gave **11b** (74% NMR yield; 74% isolated yield, 27.0 mg) as a white needles.

**R*f*** 0.38 [pentane:EtOAc (19:1)].

**Column eluent:** 200 mL pentane:EtOAc (19:1).

$^{1}$**H NMR (600 MHz, CDCl$_3$):** δ 8.31 (s, 1H), 7.98 (s, 1H), 7.70 – 7.63 (m, 2H), 7.50 (t, *J* = 8.0 Hz, 2H), 7.40 (td, *J* = 7.3, 1.2 Hz, 1H).

$^{13}$**C NMR (151 MHz, CDCl$_3$):** δ 143.30, 138.86, 131.95, 129.89, 128.41, 120.01, 113.15, 94.44.

Data in accordance with the literature.[3]

### 4-Nitro-1-phenyl-1*H*-pyrazole (12b)

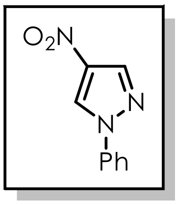


Following **GP1a**, **12a** (22.6 mg, 0.2 mmol, 1.0 equiv.) gave **12b** (73% NMR yield; 73% isolated yield, 27.7 mg) as white needles.

**R*f*** 0.45 [pentane:EtOAc (19:1)].

**Column eluent:** 200 mL pentane:EtOAc (19:1).

$^{1}$**H NMR (600 MHz, CDCl$_3$):** δ 8.63 (s, 1H), 8.26 (s, 1H), 7.70 (d, *J* = 1.0 Hz, 2H), 7.52 (t, *J* = 8.0 Hz, 2H), 7.43 (t, *J* = 7.5 Hz, 1H).

**$^{13}$C NMR (151 MHz, CDCl$_3$):** δ 138.77, 137.43, 136.84, 129.98, 128.80, 125.97, 119.94.

Data in accordance with the literature.[6]

**Ethyl 3,5-Dimethyl-1-phenyl-1*H*-pyrazole-4-carboxylate (13b)**

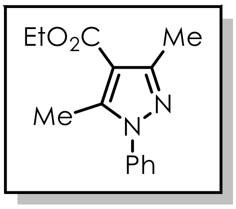


Following **GP1a**, **13a** (47.8 mg, 0.2 mmol, 1.0 equiv.) gave **13b** (75% NMR yield, <10% rsm; 75% isolated yield, 36.8 mg) as a yellow oil.

**R*f*** 0.76 [hexane:EtOAc (8:2)]

**Column eluent:** 150 mL pentane:$CH_2Cl_2$ (1:1) → 150 mL pentane:EtOAc (9:1)

**$^{1}$H NMR (600 MHz, CDCl$_3$):** δ 7.49 – 7.43 (m, 2H), 7.43 – 7.34 (m, 3H), 4.31 (q, *J* = 7.1 Hz, 2H), 2.50 (s, 3H), 2.49 (s, 3H), 1.36 (t, *J* = 7.2 Hz, 3H).

**$^{13}$C NMR (151 MHz, CDCl$_3$):** δ 164.69, 151.55, 144.58, 138.89, 129.28, 128.52, 125.75, 110.92, 59.83, 14.52, 14.43, 12.68.

Data in accordance with the literature.[7,8]

**3,5-Dimethyl-4-nitro-1-phenyl-1*H*-pyrazole (14b)**

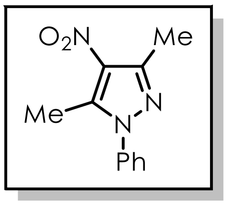


Following **GP1a**, **14a** (28.2 mg, 0.2 mmol, 1.0 equiv.) gave **14b** (83% NMR yield; 83% isolated yield, 35.6 mg) as white crystals.

**R*f*** 0.38 [pentane:EtOAc (19:1)].

**Column eluent:** 200 mL pentane:EtOAc (19:1).

**$^{1}$H NMR (600 MHz, CDCl$_3$):** δ 7.52 (dd, *J* = 8.3, 6.4 Hz, 2H), 7.50 – 7.45 (m, 1H), 7.42 – 7.37 (m, 2H), 2.62 (s, 3H), 2.58 (s, 3H).

**$^{13}$C NMR (151 MHz, CDCl$_3$):** δ 146.99, 140.93, 137.99, 132.27, 129.59, 129.47, 125.76, 14.25, 13.04.

Data in accordance with the literature.[9]

**4-Chloro-3,5-dimethyl-1-phenyl-1*H*-pyrazole (15b)**

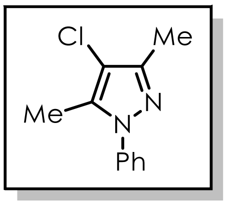


Following **GP1a**, but irradiating the reaction mixture for 4 h, **15a** (26.1 mg, 0.2 mmol, 1.0 equiv.) gave **15b** (54% NMR yield, 24% rsm; 46% isolated yield, 19.3 mg) as a white solid.

**R*f*** 0.54 [pentane:EtOAc (9:1)]

**Column eluent:** 200 mL pentane:EtOAc (19:1).

**$^{1}$H NMR (600 MHz, CDCl$_3$):** δ 7.46 (dd, *J* = 8.4, 7.2 Hz, 2H), 7.43 – 7.39 (m, 2H), 7.39 – 7.34 (m, 1H), 2.30 (s, 6H).

**$^{13}$C NMR (151 MHz, CDCl$_3$):** δ 146.18, 139.89, 135.83, 129.30, 127.85, 124.68, 109.94, 11.53, 10.96.

Data in accordance with the literature.[10]

**4-Bromo-3,5-dimethyl-1-phenyl-1*H*-pyrazole (16b)**

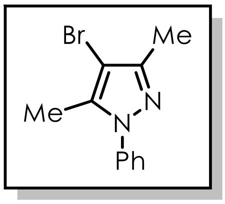


Following **GP1a**, but irradiating the reaction mixture for 4 h, **16a** (35.0 mg, 0.2 mmol, 1.0 equiv.) gave **16b** (59% NMR yield, 33% rsm; 60% isolated yield, 30.3 mg) as a yellow oil.

**R*f*** 0.82 [pentane:EtOAc (8:2)]

**Column eluent:** 200 mL pentane:EtOAc (97:3)

**$^{1}$H NMR (600 MHz, CDCl$_3$):** δ 7.49 – 7.43 (m, 2H), 7.43 – 7.34 (m, 3H), 2.30 (s, 3H), 2.30 (s, 3H).

**$^{13}C$ NMR (151 MHz, $CDCl_3$):** δ 147.69, 139.94, 137.62, 129.29, 127.93, 124.79, 96.49, 12.49, 11.88.

Data in accordance with the literature.[10,11]

**4-Iodo-3,5-dimethyl-1-phenyl-1*H*-pyrazole (17b)**

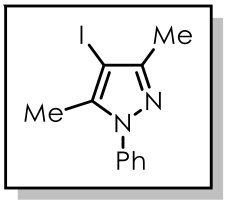


Following **GP1a**, but irradiating the reaction mixture for 6 h, **17a** (44.4 mg, 0.2 mmol, 1.0 equiv.) gave **17b** (54% NMR yield 28% rsm; 55% isolated yield, 18.9 mg) as a yellow oil.

**R*f*** 0.48 [pentane:EtOAc (9:1)].

**Column eluent:** 150 mL pentane:$CH_2Cl_2$ (1:1) → 200 mL pentane:EtOAc (9:1).

**$^{1}H$ NMR (600 MHz, $CDCl_3$):** δ 7.49 – 7.43 (m, 2H), 7.41 – 7.35 (m, 3H), 2.33 (s, 3H), 2.31 (s, 3H).

**$^{13}C$ NMR (151 MHz, $CDCl_3$):** δ 150.88, 141.00, 140.04, 129.26, 128.02, 124.94, 65.41, 14.22, 13.55.

Data in accordance with the literature.[12]

***N*-Methyl-1-phenyl-1*H*-pyrazole-4-carboxamide (18b)**

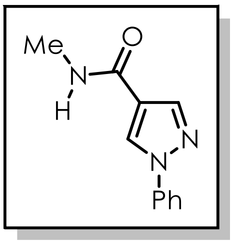


Following **GP1a**, but irradiating the reaction mixture for 4 h, **18a** (25.0 mg, 0.2 mmol, 1.0 equiv.) gave **18b** (54% NMR yield, 27% rsm; 54% isolated yield, 21.7 mg) as light-yellow solid.

**R*f*** 0.10 [pentane:acetone (8:2)].

**Column eluent:** 100 mL pentane:acetone (8:2) → 200 mL pentane:acetone (6:4).

**$^{1}$H NMR (600 MHz, CDCl$_3$):** δ 8.39 (d, *J* = 0.7 Hz, 1H), 7.95 (s, 1H), 7.71 – 7.62 (m, 2H), 7.45 (dd, *J* = 8.6, 7.3 Hz, 2H), 7.37 – 7.29 (m, 1H), 6.21 (br s, 1H), 2.98 (d, *J* = 4.8 Hz, 3H).

**$^{13}$C NMR (151 MHz, CDCl$_3$):** δ 163.13, 139.58, 139.18, 129.72, 128.53, 127.52, 120.46, 119.57, 26.47.

**HRMS (ESI):** found MH$^+$ 202.0973, $C_{11}H_{12}N_3O$ requires 202.0975.

### 3,5-Dibromo-4-nitro-1-phenyl-1*H*-pyrazole (19b)

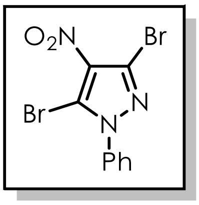


Following **GP1a**, but irradiating the reaction mixture for 4 h, **19a** (54.2 mg, 0.2 mmol, 1.0 equiv.) gave **19b** (35% NMR yield; 35% isolated yield, 24.3 mg) as white needles.

**R*f*** 0.70 [pentane:EtOAc (8:2)].

**Column eluent:** 200 mL pentane:EtOAc (19:1).

**$^{1}$H NMR (600 MHz, CDCl$_3$):** δ 7.59 – 7.53 (m, 3H), 7.53 – 7.48 (m, 2H).

**$^{13}$C NMR (151 MHz, CDCl$_3$):** δ 137.50, 130.63, 129.63, 126.21, 125.20, 116.52.

**HRMS (APCI):** found MH$^+$ 345.8817, $C_9H_6Br_2N_3O_2$ requires 345.8821.

Following **GP3**, **19a** (54.2 mg, 0.2 mmol, 1.0 equiv.) gave **19b** (39% NMR yield; 40% isolated yield, 27.8 mg) as a white solid.

**3,4,5-Tribromo-1-phenyl-1*H*-pyrazole (20b)**

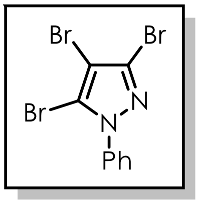


Following **GP1a**, but irradiating the reaction mixture for 4 h, **20a** (60.9 mg, 0.2 mmol, 1.0 equiv.) gave **20b** (79% NMR yield; 79% isolated yield, 60.2 mg) as white crystals.

**R*f*** 0.67 [pentane:EtOAc (19:1)].

**Column eluent:** 200 mL pentane:EtOAc (97:3).

**$^{1}$H NMR (600 MHz, $CDCl_3$):** δ 7.55 – 7.43 (m, 5H).

**$^{13}$C NMR (151 MHz, $CDCl_3$):** δ 138.79, 130.18, 129.43, 129.29, 125.50, 116.57, 102.11.

**HRMS (APCI):** found $MH^+$ 378.8078, $C_9H_6N_2Br_3$ requires 378.8076.

**Dimethyl 1-Phenyl-1*H*-pyrazole-3,5-dicarboxylate (21b)**

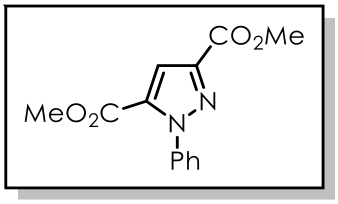


Following **GP1a**, but irradiating the reaction mixture for 4 h, **21a** (36.8 mg, 0.2 mmol, 1.0 equiv.) gave **21b** (84% NMR yield, 10% rsm; 85% isolated yield, 44.1 mg) as colourless needles.

**R*f*** 0.41 [pentane:EtOAc (8:2)]

**Column eluent:** 150 mL pentane:$CH_2Cl_2$ (1:1) → 150 mL pentane:EtOAc (9:1)

**$^{1}$H NMR (600 MHz, $CDCl_3$):** δ 7.51 (s, 1H), 7.46 (dd, *J* = 5.3, 1.9 Hz, 3H), 7.44 – 7.40 (m, 2H), 3.95 (s, 3H), 3.79 (s, 3H).

**$^{13}$C NMR (151 MHz, $CDCl_3$):** δ 162.04, 158.92, 143.54, 139.70, 134.64, 129.52, 128.75, 126.19, 114.78, 52.45, 52.43.

Data in accordance with the literature.[13,14]

### 3,5-Dimethyl-1-phenyl-1*H*-pyrazole (22b)

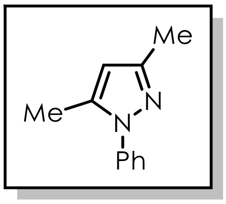


Following **GP1a**, but irradiating the reaction mixture for 4 h, **22a** (19.2 mg, 0.2 mmol, 1.0 equiv.) gave **22b** (54% NMR yield, 28% rsm; 55% isolated yield, 18.9 mg) as a light-yellow oil.

**R*f*** 0.29 [pentane:EtOAc (9:1)]

**Column eluent:** 150 mL pentane:$CH_2Cl_2$ (1:1) → 200 mL pentane:EtOAc (9:1).

**$^1$H NMR (600 MHz, $CDCl_3$):** δ 7.47 – 7.40 (m, 4H), 7.36 – 7.31 (m, 1H), 5.99 (s, 1H), 2.30 (d, *J* = 1.5 Hz, 6H).

**$^{13}$C NMR (151 MHz, $CDCl_3$):** δ 149.07, 140.04, 139.48, 129.09, 127.34, 124.87, 107.01, 13.62, 12.48.

Data in accordance with the literature.[5]

### 3,5-Diisopropyl-1-phenyl-1*H*-pyrazole (23b)

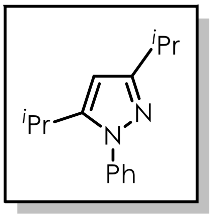


Following **GP1a**, but irradiating the reaction mixture for 4 h, **23a** (30.4 mg, 0.2 mmol, 1.0 equiv.) gave **23b** (33% NMR yield, 32% rsm; 33% isolated yield, 15.1 mg) as a light-yellow oil.

**R*f*** 0.46 [pentane:EtOAc (9:1)]

**Column eluent:** 150 mL pentane:$CH_2Cl_2$ (1:1) → 200 mL pentane:EtOAc (9:1)

**$^1$H NMR (600 MHz, $CDCl_3$):** δ 7.47 – 7.39 (m, 4H), 7.39 – 7.33 (m, 1H), 6.04 (s, 1H), 3.01 (hept, *J* = 1.7 Hz, 1H), 3.01 (hept, *J* = 1.7 Hz, 1H), 1.30 (d, *J* = 6.9 Hz, 6H), 1.17 (d, *J* = 6.8 Hz, 6H).

**$^{13}C$ NMR (151 MHz, $CDCl_3$):** δ 159.27, 150.72, 140.31, 129.03, 127.68, 125.97, 99.54, 27.96, 25.53, 23.06, 22.94.

Data in accordance with the literature.[15]

### 3-Methoxy-4-nitro-1-phenyl-1*H*-pyrazole (24b) & 5-Methoxy-4-nitro-1-phenyl-1*H*-pyrazole (24b')

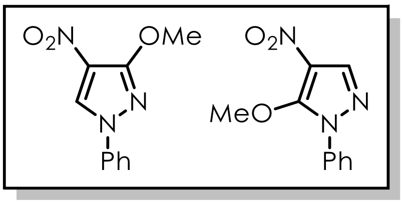


Following **GP1b**, **24a** (28.6 mg, 0.2 mmol, 1.0 equiv.) gave **24b** (41% NMR yield, 34% rsm; 47% total isolated yield, 20.6 mg) and **24b'** (6% NMR yield, 34% rsm; 47% total isolated yield, 20.6 mg) as an inseparable mixture of isomers, as colourless needles. **24b:24b'** = 6.25:1 (the ratio was determined by crude $^{1}H$ NMR).

**$R_f$** 0.46 [pentane:$CH_2Cl_2$ (1:1)].

**Column eluent:** 300 mL pentane:$CH_2Cl_2$ (1:1)

**$^{1}H$ NMR (600 MHz, $CDCl_3$):** δ 8.52 (s, 1H, **24b**), 8.16 (s, 0.17H, **24b'**), 7.69 – 7.64 (m, 2H, **24b**), 7.64 – 7.60 (m, 0.35H, **24b'**), 7.53 – 7.47 (m, 2.38H, **24b** & **24b'**), 7.46 – 7.42 (m, 0.24H, **24b'**), 7.41 – 7.36 (m, 1H, **24b**), 4.17 (s, 0.53H, **24b'**), 4.15 (s, 3H, **24b**).

**$^{13}C$ NMR (151 MHz, $CDCl_3$):** δ 157.16 (**24b**), 150.36 (**24b'**), 138.51 (2C, **24b** & **24b'**), 137.05 (**24b**), 136.97 (**24b'**), 129.89 (**24b**), 129.45 (**24b'**), 128.83 (**24b'**), 128.18 (**24b**), 127.50 (**24b**), 123.43 (**24b'**), 123.33 (**24b'**), 119.04 (**24b**), 63.62 (**24b'**), 57.45 (**24b**).

**HRMS (ESI):** found $MH^+$ 220.0721, $C_{10}H_{10}N_3O_3$ requires 220.0717.

Following **GP1a**, but irradiating the reaction mixture for 6 h, **24a** (28.6 mg, 0.2 mmol, 1.0 equiv.) gave **24b** (27% NMR yield, 43% rsm; 32% total isolated yield, 14.0 mg) and **24b'** (6% NMR yield, 43% rsm; 32% total isolated yield, 14.0 mg) as an inseparable mixture of isomers, as colourless needles. **24b:24b'** = 4.5:1 (the ratio was determined by crude $^{1}H$ NMR).

**2-(1-Phenyl-1*H*-pyrazol-4-yl)ethan-1-ol (25b)**

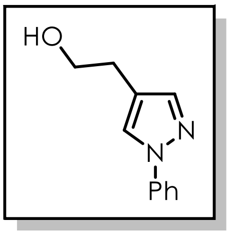


Following **GP1a**, **25a** (22.4 mg, 0.2 mmol, 1.0 equiv.) gave **25b** (32% NMR yield, 42% rsm; 31% isolated yield, 11.6 mg) as a yellow semi-solid.

**R*f*** 0.33 [pentane:acetone (3:1)].

**Column eluent:** 100 mL pentane:acetone (9:1) → 100 mL pentane:acetone (7:3) → 100 mL pentane:acetone (8:2).

**$^{1}$H NMR (600 MHz, $CDCl_3$):** δ 7.80 (d, *J* = 0.9 Hz, 1H), 7.68 – 7.62 (m, 2H), 7.58 (s, 1H), 7.42 (dd, *J* = 8.6, 7.3 Hz, 2H), 7.26 (dt, *J* = 14.8, 1.1 Hz, 1H), 3.82 (t, *J* = 6.4 Hz, 2H), 2.78 (t, *J* = 6.4 Hz, 2H).

**$^{13}$C NMR (151 MHz, $CDCl_3$):** δ 141.27, 140.21, 129.52, 126.38, 125.84, 120.01, 119.00, 63.09, 27.86.

Data in accordance with the literature.[16]

**Dimethyl 1-Phenyl-1*H*-imidazole-4,5-dicarboxylate (26b)**

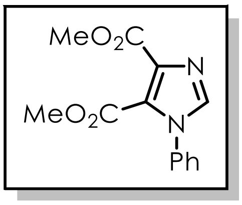


Following **GP1b**, **26a** (36.8 mg, 0.2 mmol, 1.0 equiv.) gave **26b** (75% NMR yield; 75% isolated yield, 39.2 mg) as a brown semi-solid.

**R*f*** 0.43 [pentane:acetone (7:3)].

**Column eluent:** 100 mL pentane:acetone (9:1) → 100 mL pentane:acetone (8:2) → 100 mL pentane:acetone (7:3).

**$^{1}$H NMR (600 MHz, $CDCl_3$):** δ 7.63 (s, 1H), 7.52 – 7.42 (m, 3H), 7.37 – 7.28 (m, 2H), 3.92 (s, 3H), 3.76 (s, 3H).

**$^{13}$C NMR (151 MHz, $CDCl_3$):** δ 162.44, 160.49, 138.76, 135.40, 135.18, 129.69, 129.63, 127.99, 125.27, 52.94, 52.39.

Data in accordance with the literature.[17]

**1-Phenyl-1*H*-imidazole-4,5-dicarbonitrile (27b)**

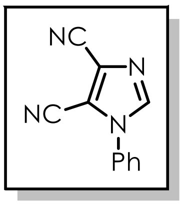


Following **GP1a**, **27a** (23.6 mg, 0.2 mmol, 1.0 equiv.) gave **27b** (60% NMR yield; 60% isolated yield, 23.3 mg) as a yellow solid.

**R*f*** 0.39 [pentane:EtOAc (8:2)].

**Column eluent:** 150 mL pentane:$CH_2Cl_2$ (1:1) → 200 mL pentane:EtOAc (8:2).

**$^1$H NMR (600 MHz, $CDCl_3$):** δ 7.90 (s, 1H), 7.67 – 7.58 (m, 3H), 7.53 – 7.43 (m, 2H).

**$^{13}$C NMR (151 MHz, $CDCl_3$):** δ 140.82, 133.30, 131.08, 130.72, 124.35, 124.33, 112.61, 111.47, 107.95.

**HRMS (ESI):** found $MH^+$ 195.0666, $C_{11}H_7N_4$ requires 195.0665.

**5-Bromo-2-methyl-4-nitro-1-phenyl-1*H*-imidazole (28b) & 4-Bromo-2-methyl-5-nitro-1-phenyl-1*H*-imidazole (28b')**

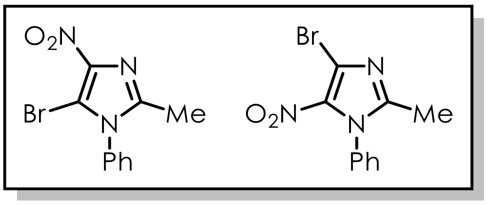


Following **GP3**, **28a** (41.2 mg, 0.2 mmol, 1.0 equiv.) gave **28b** (31% NMR yield, 32% rsm; 30% isolated yield, 16.7 mg) and **28b'** (20% NMR yield, 32% rsm; *not isolated*) as a separable mixture of isomers, as a brown solid. **28b:28b'** = 1.6:1 (the ratio was determined by crude $^1$H NMR). *<u>Note</u>: the carbon peak at 136.34 ppm is quite broad but 2D $^1$H-$^{13}$C HMBC enabled to unambiguously identified this peak as belonging to **28b**.*

<u>Characterisation data for the major isomer **28b**</u>:

**R*f*** 0.33 [pentane:EtOAc (8:2)].

**Column eluent:** 150 mL pentane:$CH_2Cl_2$ (1:1) → 100 mL pentane:EtOAc (9:1) → 100 mL pentane:EtOAc (8:2).

**$^1$H NMR (600 MHz, $CDCl_3$):** δ 7.59 – 7.52 (m, 3H), 7.26 – 7.22 (m, 2H), 2.24 (s, 3H).

**$^{13}$C NMR (151 MHz, $CDCl_3$):** δ 149.48, 136.34, 135.29, 130.36, 130.09, 126.87, 120.25, 14.43.

**HRMS (ESI):** found $MH^+$ 281.9872, $C_{10}H_9BrN_3O_2$ requires 281.9873.

**2-Nitro-1-phenyl-1*H*-imidazole (29b)**

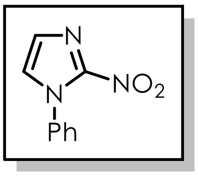


Following **GP3**, **29a** (22.6 mg, 0.2 mmol, 1.0 equiv.) gave **29b** (57% NMR yield <10% rsm; 65% isolated yield, 24.7 mg) as light-brown needles.

**$R_f$** 0.21 [pentane:EtOAc (8:2)].

**Column eluent:** 150 mL pentane:$CH_2Cl_2$ (1:1) → 200 mL pentane:EtOAc (7:3).

**$^1$H NMR (600 MHz, $CDCl_3$):** δ 7.55 – 7.49 (m, 3H), 7.35 – 7.30 (m, 2H), 7.25 (d, *J* = 1.2 Hz, 1H), 7.17 (d, *J* = 1.2 Hz, 1H).

**$^{13}$C NMR (151 MHz, $CDCl_3$):** δ 136.80, 129.93, 129.68, 128.64, 126.84, 125.81.

**HRMS (ESI):** found $MH^+$ 190.0610, $C_9H_8N_3O_2$ requires 190.0611.

Following **GP1a**, but irradiating the reaction mixture for 6 h, **29a** (22.6 mg, 0.2 mmol, 1.0 equiv.) gave **29b** (33% NMR yield, 45% rsm) as a light-yellow solid.

**Ethyl 1-Phenyl-1*H*-1,2,3-triazole-4-carboxylate (30b) & Ethyl 2-Phenyl-2*H*-1,2,3-triazole-4-carboxylate (30b') & Ethyl 1-Phenyl-1*H*-1,2,3-triazole-5-carboxylate (30b'')**

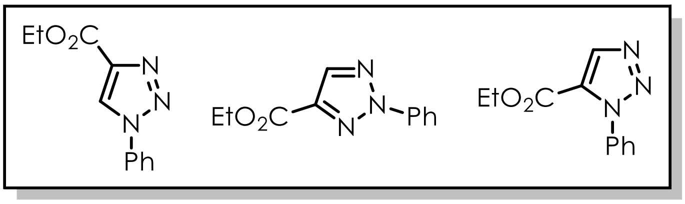


Following **GP1a**, but irradiating the reaction mixture for 4 h, **30a** (28.2 mg, 0.2 mmol, 1.0 equiv.) gave **30b** (10% NMR yield, 15% rsm; 57% total isolated yield with **30b''**, 24.8 mg), **30b'** (15% NMR yield, 15% rsm; 18% isolated yield, 8.0 mg) and **30b''** (47% NMR yield, 15% rsm; 57% total isolated yield **30b**, 24.8 mg) as a separable mixture of isomers. **30b'** could be isolated separately and it was isolated as a white semi-solid. **30b** and **30b''** were isolated as an inseparable mixture of isomers and as a yellow oil. **30b:30b':30b''** = 0.22:0.32:1 (the ratio was determined by crude $^1$H NMR).

Characterisation data for **30b'**:

**R*f*** 0.66 [pentane:EtOAc (8:2)].

**Column eluent:** 100 mL pentane:EtOAc (19:1) → 100 mL pentane:EtOAc (9:1).

**$^1$H NMR (600 MHz, $CDCl_3$):** δ 8.23 (s, 1H), 8.15 (d, *J* = 8.0 Hz, 2H), 7.51 (t, *J* = 8.0 Hz, 2H), 7.42 (t, *J* = 7.4 Hz, 1H), 4.47 (q, *J* = 7.2 Hz, 2H), 1.44 (t, *J* = 7.1 Hz, 3H).

**$^{13}$C NMR (151 MHz, $CDCl_3$):** δ 160.67, 141.13, 139.39, 137.83, 129.39, 128.67, 119.64, 61.66, 14.33.

Data in accordance with the literature.[3]

Characterisation data for **30b** and **30b''**:

**R*f*** 0.26 [pentane:EtOAc (8:2)] for (**30b**).

**R*f*** 0.35 [pentane:EtOAc (8:2)] for (**30b''**).

**Column eluent:** 100 mL pentane:EtOAc (19:1) → 100 mL pentane:EtOAc (9:1). → 200 mL pentane:EtOAc (8:2).

$^{1}$**H NMR (600 MHz, $CDCl_3$):** δ 8.52 (s, 0.22H, **30b''**), 8.27 (s, 1H, **30b**), 7.78 – 7.73 (m, 0.47H, **30b''**), 7.58 – 7.45 (m, 5.78H, **30b** & **30b''**), 4.46 (q, *J* = 7.1 Hz, 0.47H, **30b''**), 4.29 (q, *J* = 7.2 Hz, 2H, **30b**), 1.43 (t, *J* = 7.1 Hz, 0.76H, **30b''**), 1.27 (t, *J* = 7.1 Hz, 3H, **30b**).

$^{13}$**C NMR (151 MHz, $CDCl_3$):** δ 160.78 (**30b**), 157.82 (**30b''**), 140.94 (**30b**), 138.28 (**30b''**), 136.53 (**30b''**), 136.45 (**30b**), 130.25 (**30b''**), 130.08 (**30b**), 129.69 (**30b**), 129.39 (**30b''**), 129.03 (**30b''**), 125.98 (**30b''**), 125.67 (**30b**), 120.96 (**30b**), 62.06 (**30b''**), 61.70 (**30b**), 14.43 (**30b**), 14.07 (**30b''**).

Data in accordance with the literature.[18–20]

### 4,5-Dibromo-1-phenyl-1*H*-1,2,3-triazole (31b & 32b)

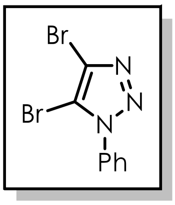


Following **GP1a**, but irradiating the reaction mixture for 4 h, **31a** (45.4 mg, 0.2 mmol, 1.0 equiv.) gave **31b** (56% NMR yield; 60% isolated yield, 34.3 mg) as a light-orange solid.

$\mathbf{R}_f$ 0.78 [pentane:EtOAc (8:2)].

**Column eluent:** 200 mL pentane:EtOAc (97:3).

$^{1}$**H NMR (600 MHz, $CDCl_3$):** δ 7.62 – 7.53 (m, 5H).

$^{13}$**C NMR (151 MHz, $CDCl_3$):** δ 135.86, 130.62, 129.69, 125.24, 124.26, 113.23.

Data in accordance with the literature.[21]

Following **GP1a**, but irradiating the reaction mixture for 4 h, **32a** (45.4 mg, 0.2 mmol, 1.0 equiv.) gave **32b** (41% NMR yield; 38% isolated yield, 22.8 mg) as light-yellow solid.

**1-Phenyl-1*H*-benzo[*d*][1,2,3]triazole (33b)**

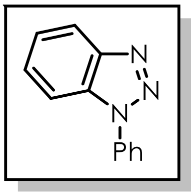

Following **GP1b**, **33a** (23.8 mg, 0.2 mmol, 1.0 equiv.) gave **33b** (46% NMR yield; 50% isolated yield, 23.6 mg) as a yellow-orange solid.

**R*f*** 0.18 [pentane:$CH_2Cl_2$ (6:4)].

**Column eluent:** 150 mL pentane:$CH_2Cl_2$ (6:4) → 200 mL pentane:EtOAc (7:3).

**$^1$H NMR (600 MHz, $CDCl_3$):** δ 8.14 (dt, *J* = 8.4, 0.9 Hz, 1H), 7.82 – 7.77 (m, 2H), 7.75 (dt, *J* = 8.4, 0.9 Hz, 1H), 7.66 – 7.59 (m, 2H), 7.55 (ddd, *J* = 8.2, 7.0, 1.0 Hz, 1H), 7.53 – 7.49 (m, 1H), 7.44 (ddd, *J* = 8.0, 7.0, 0.9 Hz, 1H).

**$^{13}$C NMR (151 MHz, $CDCl_3$):** δ 146.51, 137.06, 132.44, 130.00, 128.85, 128.41, 124.61, 123.02, 120.35, 110.51.

Data in accordance with the literature.[3,4]

Following **GP1a**, but irradiating the reaction mixture for 4 h, **33a** (23.8 mg, 0.2 mmol, 1.0 equiv.) gave **33b** (24% NMR yield, 35% rsm) as a white solid.

**5-Chloro-1-phenyl-1*H*-benzo[*d*][1,2,3]triazole (34b) & 6-Chloro-1-phenyl-1*H*-benzo[*d*][1,2,3]triazole (34b')**

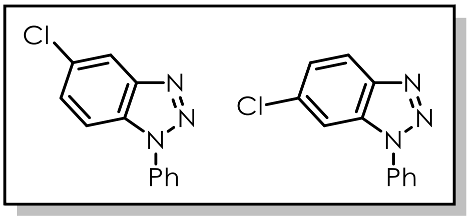

Following **GP1b**, **34a** (30.7 mg, 0.2 mmol, 1.0 equiv.) gave **34b** (30% NMR yield; 66% isolated yield, 18.9 mg) and **34b'** (36% NMR yield; 66% total isolated yield, 30.6 mg) as an inseparable mixture of isomers, as a light-yellow solid. **34b:34b'** = 0.9:1 (the ratio was determined by crude $^1$H NMR).

**R*f*** 0.21 [pentane:$CH_2Cl_2$ (1:1)].

**Column eluent:** 200 mL pentane:$CH_2Cl_2$ (1:1) → 100 mL pentane:EtOAc (9:1).

**$^1$H NMR (600 MHz, $CDCl_3$):** δ 8.11 (d, *J* = 1.8 Hz, 0.92H, **34b**), 8.05 (d, *J* = 8.8 Hz, 1H, **34b'**), 7.77 – 7.71 (m, 4.9H, **34b** & **34b'**), 7.67 (d, *J* = 8.8 Hz, 1H, **34b'**), 7.62 (td, *J* = 7.9, 3.4 Hz, 3.92H, **34b** & **34b'**), 7.52 (t, *J* = 7.5 Hz, 2H, **34b'**), 7.50 (dd, *J* = 8.8, 1.9 Hz, 0.92H, **34b**), 7.39 (dd, *J* = 8.8, 1.8 Hz, 1H, **34b'**).

**$^{13}$C NMR (151 MHz, $CDCl_3$):** δ 147.22, 145.14, 136.70, 136.63, 134.89, 133.01, 131.14, 130.35, 130.12, 130.09, 129.23, 129.15, 125.72, 123.01, 122.97, 121.32, 119.75, 111.43, 110.33.

Data in accordance with the literature.[22]

**1-Phenyl-1*H*-benzo[*d*][1,2,3]triazole-6-carbonitrile (35b) & 1-Phenyl-1*H*-benzo[*d*][1,2,3]triazole-5-carbonitrile (35b') & 2-Phenyl-2*H*-benzo[*d*][1,2,3]triazole-5-carbonitrile (35b")**

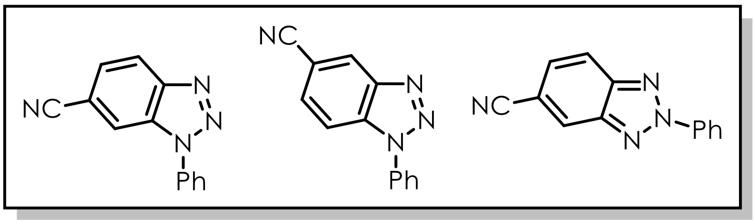


Following **GP1a**, but irradiating the reaction mixture for 4 h, **35a** (28.8 mg, 0.2 mmol, 1.0 equiv.) gave **35b** (49% NMR yield; 76% total isolated yield, 33.5 mg) and **35b'** (27% NMR yield; 76% total isolated yield, 33.5 mg) and **35b"** (14% NMR yield; *not isolated*) as an inseparable mixture of isomers (for **35b** and **35b'**), as a white solid. **35b:35b':35b"** = 0.54:0.3:0.16 (the ratio was determined by crude $^1$H NMR).

Characterisation data for **35b** and **35b'**:

**R*f*** 0.42 [pentane:EtOAc (8:2)].

**Column eluent:** 100 mL pentane:EtOAc (19:1) → 200 mL pentane:EtOAc (9:1) → 100 mL pentane:EtOAc (8:2).

**$^1$H NMR (600 MHz, $CDCl_3$):** δ 8.56 – 8.51 (m, 0.66H, **35b'**), 8.26 (dd, *J* = 8.6, 0.9 Hz, 1H, **35b**), 8.16 – 8.11 (m, 1H, **35b**), 7.84 (dd, *J* = 8.6, 0.9 Hz, 0.7H, **35b'**), 7.79 – 7.72 (m, 4H, **35b** & **35b'**), 7.70 – 7.62 (m, 4.36H, **35b** & **35b'**), 7.62 – 7.55 (m, 1.69H, **35b** & **35b'**).

**$^{13}$C NMR (151 MHz, $CDCl_3$):** δ 147.74 (**35b**), 145.75 (**35b'**), 136.16 (**35b'**), 136.14 (**35b**), 134.09 (**35b'**), 131.91 (**35b**), 130.58 (**35b**), 130.36 (**35b**), 130.28 (**35b'**), 129.79 (**35b**),

129.70 (**35b'**), 126.93 (**35b**), 126.51 (**35b'**), 123.25 (**35b'**), 123.18 (**35b**), 121.93 (**35b**), 118.45 (**35b'**), 118.39 (**35b'**), 116.35 (**35b**), 112.01 (**35b**), 111.94 (**35b'**), 108.40 (**35b'**).

Data in accordance with the literature.[23]

Following **GP1b**, **35a** (28.8 mg, 0.2 mmol, 1.0 equiv.) gave **35b** (45% NMR yield) and **35b'** (26% NMR yield) and **35b"** (18% NMR yield) as an inseparable mixture of isomers (for **35b** and **35b'**), as a white solid. **35b:35b':35b"** = 0.45:0.33:0.22 (the ratio was determined by crude $^{1}$H NMR).

**4-Nitro-1-phenyl-1*H*-benzo[*d*][1,2,3]triazole (36b)**

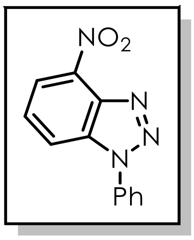


Following **GP1a**, but irradiating the reaction mixture for 4 h, **36a** (32.8 mg, 0.2 mmol, 1.0 equiv.) gave **36b** (52% NMR yield, 20% rsm; 50% isolated yield, 25.0 mg) as yellow crystals. *Note: the product was isolated alongside ~8% of starting material.*

**R*f*** 0.22 [pentane:EtOAc (8:2)].

**Column eluent:** 150 mL pentane:$CH_2Cl_2$ (1:1) → 250 mL pentane:EtOAc (8:2).

**$^{1}$H NMR (600 MHz, $CDCl_3$):** δ 8.29 (d, *J* = 7.7 Hz, 1H), 8.05 (d, *J* = 8.4 Hz, 1H), 7.74 – 7.65 (m, 3H), 7.63 (dd, *J* = 8.5, 7.1 Hz, 2H), 7.59 – 7.53 (m, 1H).

**$^{13}$C NMR (151 MHz, $CDCl_3$):** δ 139.05, 138.89, 135.84, 134.88, 130.22, 129.94, 127.57, 123.66, 121.65, 117.44.

**Methyl 1-Phenyl-1*H*-tetrazole-5-carboxylate (37b) & Methyl 2-Phenyl-2*H*-tetrazole-5-carboxylate (37b')**

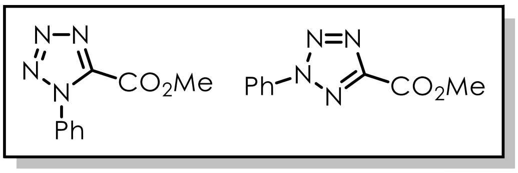


Following **GP1a**, **37a** (25.6 mg, 0.2 mmol, 1.0 equiv.) gave **37b** (42% NMR yield, 20% rsm; 20% isolated yield, 8.2 mg) and **37b'** (27% NMR yield, 20% rsm; 20% isolated yield, 8.2 mg) as a separable mixture of isomers, as a white solid for **37b** and as light-yellow crystals for **37b'**. **37b:37b'** = 1.6:1 (the ratio was determined by crude $^{1}H$ NMR).

Characterisation data for **37b**:

**R*f*** 0.3 [pentane:EtOAc (8:2)].

**Column eluent:** 150 mL pentane:$CH_2Cl_2$ (1:1) → 200 mL pentane:EtOAc (7:3).

**$^{1}H$ NMR (600 MHz, $CDCl_3$):** δ 7.64 – 7.54 (m, 3H), 7.52 – 7.46 (m, 2H), 4.43 (q, *J* = 7.1 Hz, 2H), 1.36 (t, *J* = 7.1 Hz, 3H).

**$^{13}C$ NMR (151 MHz, $CDCl_3$):** δ 155.99, 146.29, 134.08, 131.10, 129.51, 125.60, 63.78, 13.98.

**HRMS (ESI):** found $MH^+$ 205.0721, $C_9H_9N_4O_2$ requires 205.0720.

Data in accordance with the literature.[24]

Characterisation data for **37b'**:

**R*f*** 0.5 [pentane:EtOAc (8:2)].

**Column eluent:** 150 mL pentane:$CH_2Cl_2$ (1:1) → 200 mL pentane:EtOAc (7:3).

**$^{1}H$ NMR (600 MHz, $CDCl_3$):** δ 8.25 – 8.15 (m, 2H), 7.64 – 7.51 (m, 3H), 4.57 (q, *J* = 7.2 Hz, 2H), 1.49 (t, *J* = 7.2 Hz, 3H).

**$^{13}C$ NMR (151 MHz, $CDCl_3$):** δ 157.83, 136.41, 130.66, 129.86, 129.62, 120.35, 62.85, 14.22.

**HRMS (ESI):** found $MH^+$ 205.0721, $C_9H_9N_4O_2$ requires 205.0720.

Data in accordance with the literature.[25]

**5-(4-(Trifluoromethyl)phenyl)-1*H*-tetrazole (38b)**

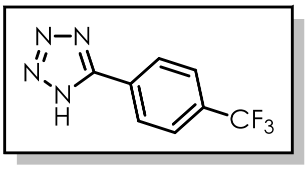

Following **GP1a**, but irradiating the reaction mixture for 4 h, **38a** (41.8 mg, 0.2 mmol, 1.0 equiv.) gave **38b** (59% NMR yield; 54% isolated yield, 31.3 mg) as a white solid.

**R*f*** 0.86 [pentane:EtOAc (8:2)].

**Column eluent:** 200 mL pentane:EtOAc (98:2).

**$^{1}$H NMR (600 MHz, $CDCl_3$):** δ 8.38 (d, *J* = 8.0 Hz, 2H), 8.20 (d, *J* = 8.0 Hz, 2H), 7.79 (d, *J* = 8.1 Hz, 2H), 7.59 (t, *J* = 7.8 Hz, 2H), 7.53 (t, *J* = 7.4 Hz, 1H).

**$^{13}$C NMR (151 MHz, $CDCl_3$):** δ 164.14, 136.89, 132.44 (q, *J* = 32.7 Hz), 130.66, 130.08, 129.89, 127.47, 126.11 (q, *J* = 3.8 Hz), 124.01 (q, *J* = 272.3 Hz), 120.04.

**$^{19}$F NMR (565 MHz, $CDCl_3$):** δ -62.88 (s).

Data in accordance with the literature.[26]

**2-Butyl-3-(tetrazolo[1,5-*f*]phenanthridin-6-ylmethyl)-1,3-diazaspiro[4.4]non-1-en-4-one (39b)**

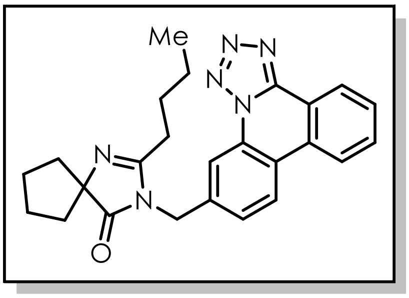

A dry 8 mL screw neck vial N15 equipped with a stirring bar was charged with **39a** (85.7 mg, 0.2 mmol, 1.0 equiv.) and **Luc/$BF_4$** (3.5 mol%, 7 µmol, 3.9 mg). Under air, dry MeOH (2 mL, *c* = 0.1 M) was added. The vial was capped with a screw cap fitted with a Silicone/PTFE septum. The resulting solution was sparged with oxygen for 5 min (either with a 1 atm balloon or directly from an oxygen tank fitted with a pressure regulator). The vial was placed in the Penn PhD Photoreactor M2 equipped with a 450 nm light source.

The parameters used for the irradiation are described in **part 1.2**. The mixture was stirred under irradiation for 4 h. Upon completion, the solvent was evaporated, and the residue was directly purified by column chromatography on silica gel to give **39b** (79% NMR yield, 16% rsm; 56% isolated yield, 48.6 mg) as a white solid.

**R*f*** 0.44 [$CH_2Cl_2$:MeOH (98:2)]

**Column eluent:** 200 mL $CH_2Cl_2$:MeOH (99:1) → 100 mL $CH_2Cl_2$:MeOH (98:2)

**$^1$H NMR (600 MHz, CDCl$_3$):** δ 8.62 (d, *J* = 7.8 Hz, 1H), 8.36 (d, *J* = 11.7 Hz, 1H), 8.35 (s, 1H), 8.32 (d, *J* = 8.2 Hz, 1H), 7.84 – 7.76 (m, 1H), 7.71 (t, *J* = 7.5 Hz, 1H), 7.46 (dd, *J* = 8.4, 1.9 Hz, 1H), 4.93 (s, 2H), 2.41 – 2.32 (m, 2H), 2.09 – 1.91 (m, 6H), 1.91 – 1.82 (m, 2H), 1.60 (p, *J* = 7.7 Hz, 2H), 1.31 (h, *J* = 7.4 Hz, 2H), 0.81 (t, *J* = 7.3 Hz, 3H).

**$^{13}$C NMR (151 MHz, CDCl$_3$):** δ 186.85, 160.99, 147.26, 139.49, 132.11, 129.59, 129.45, 129.34, 126.47, 126.05, 124.92, 122.98, 121.74, 118.51, 115.35, 43.27, 37.58, 28.87, 27.72, 26.20, 22.34, 13.76.

**HRMS (ESI):** found MH$^+$ 427.2239, $C_{25}H_{27}N_6O$ requires 427.2241.

**1-(Phenyl-*d*$_5$)-1*H*-pyrazole (40b)**

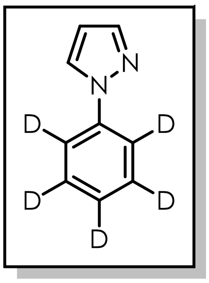


Following **GP2**, **40a** (2 mL, 110 equiv.) and **2a** (13.6 mg, 0.2 mmol, 1.0 equiv.) gave **40b** (58% NMR yield, 8% rsm; 53% isolated yield, 15.8 mg) as a light-yellow oil.

**R*f*** 0.73 [pentane:EtOAc (9:1)].

**Column eluent:** 100 mL pentane:EtOAc (19:1) → 100 mL pentane:EtOAc (9:1).

**$^1$H NMR (600 MHz, CDCl$_3$):** δ 7.93 (d, *J* = 2.4 Hz, 1H), 7.73 (d, *J* = 1.8 Hz, 1H), 6.47 (t, *J* = 2.1 Hz, 1H).

**$^{13}$C NMR (151 MHz, CDCl$_3$):** δ 141.20, 140.23, 129.05 (t, *J* = 24.5 Hz), 126.87, 126.05 (t, *J* = 24.5 Hz), 118.94 (t, *J* = 24.8 Hz), 107.71.

**$^2$H NMR (92 MHz, CDCl$_3$):** δ 7.75 (s, 2H), 7.51 (s, 2H), 7.34 (s, 1H).

**HRMS (ESI):** found $MH^+$ 150.1074, $C_9H_4D_5N_2$ requires 150.1074.

**1-(*p*-Tolyl)-1*H*-pyrazole (41b) & 1-(*m*-Tolyl)-1*H*-pyrazole (41b') & 1-(*o*-Tolyl)-1*H*-pyrazole (41b")**

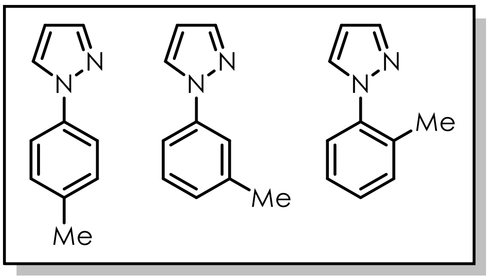


Following **GP2**, **41a** (2 mL, 94 equiv.) and **2a** (13.6 mg, 0.2 mmol, 1.0 equiv.) gave **41b** (46% NMR yield, <10% rsm; 75% total isolated yield, 23.8 mg), **41b'** (5% NMR yield, <10% rsm; 75% total isolated yield, 23.8 mg) and **41b"** (25% NMR yield, <10% rsm; 75% total isolated yield, 23.8 mg) as an inseparable mixture of isomers, as a light-yellow oil. **41b:41b':41b"** = 61:6:33 (the ratio was determined by $^1$H NMR analysis of the isolated products).

**R*f*** 0.46 [pentane:EtOAc (9:1)].

**Column eluent:** 100 mL pentane → 200 mL pentane:EtOAc (19:1) → 100 mL pentane:EtOAc (9:1).

**$^1$H NMR (600 MHz, $CDCl_3$):** δ 7.91 (dd, *J* = 2.4, 0.6 Hz, 0.10H, **41b'**), 7.88 (dd, *J* = 2.5, 0.7 Hz, 1H, **41b**), 7.73 (d, *J* = 1.9 Hz, 0.64H, **41b'** & **41b"**), 7.71 (d, *J* = 1.8 Hz, 1H, **41b**), 7.60 (dd, *J* = 2.4, 0.6 Hz, 0.55H, **41b"**), 7.59 – 7.54 (m, 2.13H, **41b** & **41b'**), 7.46 (dd, *J* = 8.1, 2.3 Hz, 0.11H, **41b'**), 7.35 – 7.30 (m, 1.75H, **41b'** & **41b"**), 7.30 – 7.27 (m, 0.58H, **41b'** & **41b"**), 7.27 – 7.22 (m, 2H, **41b**), 7.10 (dt, *J* = 7.6, 0.8 Hz, 0.09H, **41b'**), 6.47 – 6.42 (m, 1.64H, **41b** & **41b'** & **41b"**), 2.42 (s, 0.30H, **41b'**), 2.38 (s, 3H, **41b**), 2.25 (s, 1.67H, **41b"**).

**$^{13}$C NMR (151 MHz, $CDCl_3$):** δ 141.04 (**41b'**), 140.89 (**41b**), 140.36 (2C, **41b'** & **41b"**), 140.12 (**41b"**), 138.12 (**41b**), 136.37 (**41b**), 133.87 (**41b"**), 131.38 (**41b"**), 130.63 (**41b'**), 130.04 (**41b"**), 129.32 (**41b'**), 128.48 (**41b"**), 127.35 (**41b'**), 126.90 (**41b'**), 126.80 (**41b**), 126.65 (**41b"**), 126.28 (**41b"**), 120.15 (**41b'**), 119.32 (**41b**), 116.36 (**41b'**), 107.56 (**41b"**), 107.42 (**41b**), 106.28 (**41b'**), 21.59 (**41b"**), 21.03 (**41b**), 18.17 (**41b'**).

Data in accordance with the literature.[27,28]

**1-(4-Fluorophenyl)-1*H*-pyrazole (42b) & 1-(3-Fluorophenyl)-1*H*-pyrazole (42b') & 1-(2-Fluorophenyl)-1*H*-pyrazole (42b'') & 4-(1*H*-Pyrazol-1-yl)phenol (42c)**

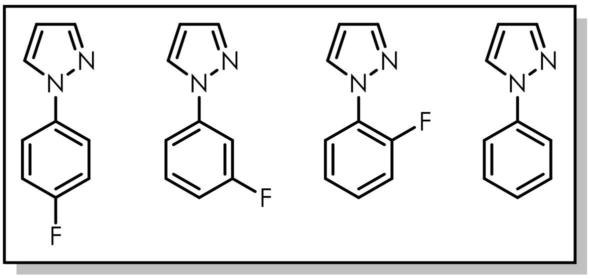
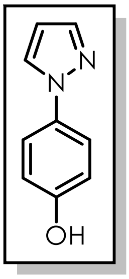

Following **GP2**, **42a** (1 mL, 55 equiv.) and **2a** (13.6 mg, 0.2 mmol, 1.0 equiv.) gave **2b** (*product from* S$_N$*Ar*), **42b**, **42b'** and **42b''** (20% total isolated yield, 6.4 mg) as an inseparable mixture, as a light-yellow oil. **42b**:**42b'**:**42b''**:**2b** = 0.43:0.20:0.28:1.0 (the ratio was determined by $^1$H NMR analysis of the isolated products).

**$R_f$** 0.53 [pentane:EtOAc (9:1)].

**Column eluent:** 100 mL pentane:EtOAc (19:1) → 100 mL pentane:EtOAc (9:1) → 100 mL pentane:EtOAc (7:3).

**$^1$H NMR (600 MHz, CDCl$_3$):** δ 8.01 (d, *J* = 2.5 Hz, 0.28H, **42b''**), 7.93 (d, *J* = 2.5 Hz, 1H, **2b**), 7.92 (d, *J* = 2.5 Hz, 0.20H, **42b'**), 7.86 (d, *J* = 2.4 Hz, 0.44H, **42b**), 7.75 (d, *J* = 1.8 Hz, 0.20H, **42b'**), 7.73 (d, *J* = 1.8 Hz, 1.34H, **42b''** & **2b**), 7.72 (d, *J* = 1.8 Hz, 0.5H, **42b**), 7.71 – 7.68 (m, 2H, **2b**), 7.67 – 7.63 (m, 0.86H, **42b**), 7.48 – 7.43 (m, 2.23H, **42b'** & **2b**), 7.38 (dd, *J* = 8.1, 1.5 Hz, 0.29H, **42b''**), 7.32 – 7.27 (m, 1H, **2b**), 7.20 – 7.09 (m, 1.48H, **42b** & **42b'** & **42b''**), 7.01 – 6.96 (m, 0.21H, **42b'**), 6.92 (ddd, *J* = 8.5, 7.4, 1.5 Hz, 0.31H, **42b''**), 6.51 (t, *J* = 2.3 Hz, 0.29H, **42b''**), 6.50 – 6.45 (m, 1.69H, **42b** & **42b'** & **2b**).

**$^{13}$C NMR (151 MHz, CDCl$_3$):** δ 163.40 (d, *J* = 246.4 Hz, **42b'**), 161.25 (d, *J* = 245.8 Hz, **42b**), 153.66 (d, *J* = 248.6 Hz, **42b''**), 141.60 (2C, **42b'**), 141.26 (**42b**), 141.21 (**2b**), 140.98 (**42b''**), 140.33 (**2b**), 136.71 (**42b**), 130.94 (d, *J* = 10.4 Hz, **42b''**), 130.86 (d, *J* = 9.3 Hz, **42b'**), 129.56 (**2b**), 127.82 (d, *J* = 7.7 Hz, **42b''**), 127.75 (**42b**), 127.03 (**42b'**), 126.92 (d, *J* = 5.8 Hz, **42b''**), 126.90 (**2b**), 126.59 (**2b**), 125.06 (d, *J* = 3.7 Hz, **42b''**), 124.52 (**42b''**), 121.15 (d, *J* = 8.4 Hz, **42b**), 119.37 (**2b**), 116.95 (d, *J* = 20.7 Hz, **42b''**), 116.34 (d, *J* = 23.0 Hz, **42b**), 114.43 (d, *J* = 3.2 Hz, **42b'**), 113.29 (d, *J* = 21.3 Hz, **42b'**), 108.20 (**42b'**), 107.84 (**42b**), 107.72 (**2b**), 106.98 (d, *J* = 26.0 Hz, **42b'**), 106.91 (**42b''**).

**$^{19}$F NMR (565 MHz, CDCl$_3$):** δ -110.96 (q, *J* = 8.2 Hz, **42b'**), -115.92 – -116.07 (m, **42b**), -125.08 – -125.22 (m, **42b''**).

Data in accordance with the literature.[3,29–31]

Under the same conditions, **42c** (*product from* S$_N$*Ar*; 19% isolated yield, 6.2 mg) was also isolated as a brown oil.

**$R_f$** 0.10 [pentane:EtOAc (9:1)].

**Column eluent:** 100 mL pentane:EtOAc (19:1) → 100 mL pentane:EtOAc (9:1) → 100 mL pentane:EtOAc (7:3).

**$^{1}$H NMR (600 MHz, $CDCl_3$):** δ 7.78 (d, *J* = 2.4 Hz, 1H), 7.72 (d, *J* = 1.9 Hz, 1H), 7.41 (d, *J* = 8.8 Hz, 2H), 6.81 (d, *J* = 8.8 Hz, 2H), 6.45 (t, *J* = 2.1 Hz, 1H).

**$^{13}$C NMR (151 MHz, $CDCl_3$):** δ 155.77, 140.57, 133.18, 128.02, 122.10, 116.37, 107.30.

Data in accordance with the literature.[32]

**1-(4-Chlorophenyl)-1*H*-pyrazole (43b) & 1-(2-Chlorophenyl)-1*H*-pyrazole (43b')**

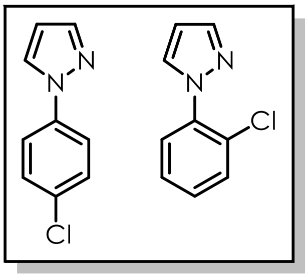


Following **GP2**, **43a** (2 mL, 100 equiv.) and **2a** (13.6 mg, 0.2 mmol, 1.0 equiv.) gave **43b** (38% NMR yield, 9% rsm; 49% total isolated yield, 17.6 mg) and **43b'** (11% NMR yield, 9% rsm; 49% total isolated yield, 17.6 mg) as an inseparable mixture of isomers, as a light-yellow oil. **43b:43b'** = 1:0.3 (the ratio was determined by $^{1}$H NMR analysis of the isolated products).

**$R_f$** 0.53 [pentane:EtOAc (9:1)].

**Column eluent:** 100 mL pentane:EtOAc (19:1) → 100 mL pentane:EtOAc (9:1) → 100 mL pentane:EtOAc (7:3).

**$^{1}$H NMR (600 MHz, $CDCl_3$):** δ 7.88 (d, *J* = 2.5 Hz, 1H, **43b**), 7.87 (d, *J* = 2.5 Hz, 0.32H, **43b'**), 7.76 (d, *J* = 1.8 Hz, 0.36H, **43b'**), 7.72 (d, *J* = 1.8 Hz, 1H, **43b**), 7.63 (d, *J* = 8.8 Hz, 2H, **43b**), 7.58 (dd, *J* = 7.9, 1.7 Hz, 0.36H, **43b'**), 7.51 (dd, *J* = 8.0, 1.5 Hz, 0.31H, **43b'**), 7.41 (d, *J* = 8.8 Hz, 2H, **43b**), 7.37 (td, *J* = 7.7, 1.5 Hz, 0.37H, **43b'**), 7.33 (td, *J* = 7.8, 1.8 Hz, 0.31H, **43b'**), 6.48 (t, 0.34H, **43b'**), 6.47 (t, *J* = 2.2 Hz, 1H, **43b**).

**$^{13}$C NMR (151 MHz, $CDCl_3$):** δ 141.48 (**43b**), 141.02 (**43b'**), 138.83 (**43b**), 138.28 (**43b'**), 132.03 (**43b**), 131.47 (**43b'**), 130.76 (**43b'**), 129.63 (**43b**), 129.16 (**43b'**), 128.52 (**43b'**), 127.93 (**43b'**), 127.78 (**43b'**), 126.86 (**43b**), 120.45 (**43b**), 108.10 (**43b**), 106.79 (**43b'**).

Data in accordance with the literature.[29,33]

**1-(4-Bromophenyl)-1*H*-pyrazole (44b) & 1-(2-Bromophenyl)-1*H*-pyrazole (44b')**

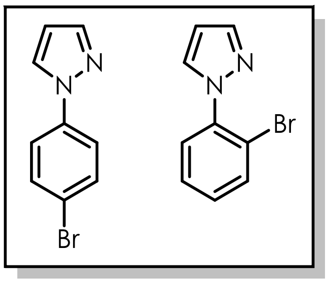


Following **GP2**, **44a** (2 mL, 95 equiv.) and **2a** (13.6 mg, 0.2 mmol, 1.0 equiv.) gave **44b** (39% NMR yield, 8% rsm; 46% total isolated yield, 20.5 mg) and **44b'** (13% NMR yield, 8% rsm; 46% total isolated yield, 20.5 mg) as an inseparable mixture of isomers, as a light-yellow oil. **44b:44b'** = 1:0.35 (the ratio was determined by $^{1}$H NMR analysis of the isolated products).

**R*f*** 0.53 [pentane:EtOAc (9:1)].

**Column eluent:** 100 mL pentane:EtOAc (19:1) → 100 mL pentane:EtOAc (9:1) → 100 mL pentane:EtOAc (7:3).

**$^{1}$H NMR (600 MHz, $CDCl_3$):** δ 7.91 – 7.86 (m, 1H, **44b**), 7.82 (d, *J* = 2.4 Hz, 0.35H, **44b'**), 7.75 (d, *J* = 1.8 Hz, 0.35H, **44b'**), 7.72 (s, 1H, **44b**), 7.69 (dd, *J* = 8.1, 1.3 Hz, 0.38H, **44b'**), 7.60 – 7.53 (m, 4H, **44b**), 7.51 (dt, *J* = 7.9, 1.4 Hz, 0.35H, **44b'**), 7.41 (td, *J* = 7.7, 1.3 Hz, 0.35H, **44b'**), 7.29 – 7.25 (m, 0.35H, **44b'**), 6.51 – 6.43 (m, 1.35H, **44b** & **44b'**).

**$^{13}$C NMR (151 MHz, $CDCl_3$):** δ 141.50 (**44b**), 140.96 (**44b'**), 139.97 (**44b'**), 139.29 (**44b**), 133.87 (**44b'**), 132.55 (**44b**), 131.41 (**44b'**), 129.70 (**44b'**), 128.46 (**44b'**), 128.33 (**44b'**), 126.75 (**44b**), 120.69 (**44b**), 119.72 (**44b**), 118.70 (**44b'**), 108.13 (**44b**), 106.62 (**44b'**).

Data in accordance with the literature.[27,33]

**1-(4-Methoxyphenyl)-1*H*-pyrazole (45b) & 1-(2-Methoxyphenyl)-1*H*-pyrazole (45b')**

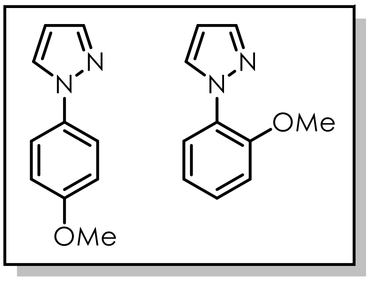

Following **GP2**, but irradiating the reaction mixture for 3 h, **45a** (2.4 mL, 110 equiv.) and **2a** (13.6 mg, 0.2 mmol, 1.0 equiv.) gave **45b** and **45b'** (16% total NMR yield, 78% rsm; 14% isolated yield) as a colorless oil. **45b:45b'** = 1:7 (the ratio was determined by crude $^{1}$H NMR).

**$^{1}$H NMR (600 MHz, $CDCl_3$):** 8.03 (d, *J* = 2.5 Hz, 1H, **45b'**), 7.82 (d, *J* = 2.5 Hz, 1H, **45b**), 7.72 (d, *J* = 2.6 Hz, **45b'**), 7.70 (d, *J* = 2.0 Hz, 1H, **45b**), 7.69 (dd, *J* = 5.6, 2.3 Hz, 2H, **45b'**), 7.59 (d, *J* =9.0 Hz, 2H, **45b**), 7.30 (td, *J* = 7.9, 1.7 Hz, 1H, **45b'**), 7.08 – 7.02 (m, 2H, **45b'**), 6.97 (d, *J* = 9.1 Hz, 2H, **45b**), 6.44 (t, *J* = 2.1 Hz, 1H, **45b'**), 6.44 (t, *J* = 2.2 Hz, 1H, **45b**), 3.86 (s, 3H, **45b'**), 3.80 (s, 3H, **45b**).

**$^{13}$C NMR (151 MHz, $CDCl_3$):** δ 158.45 (**45b**), 151.55 (**45b'**), 140.85 (**45b**), 140.15 (**45b'**), 134.30 (**45b**), 131.75 (**45b'**), 129.85 (**45b'**), 128.25 (**45b'**),126.95 (**45b**), 125.45 (**45b'**), 121.25 (**45b'**), 120.98 (**45b**), 114.65 (**45b**), 112.45 (**45b'**), 107.35 (**45b**), 106.25 (**45b'**), 56.15 (**45b'**), 55.75 (**45b**).

Data in accordance with the literature.[34,35]

**1-(2,4-Dimethylphenyl)-1*H*-pyrazole (48b) & 1-(2,6-Dimethylphenyl)-1*H*-pyrazole (48b')**

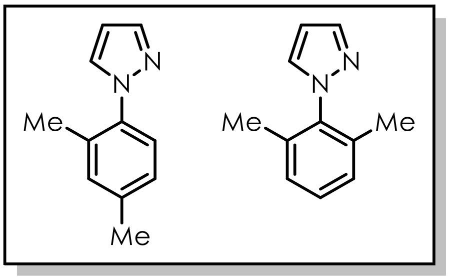

Following **GP2**, **48a** (2 mL, 80 equiv.) and **2a** (13.6 mg, 0.2 mmol, 1.0 equiv.) gave **48b** (79% NMR yield, <10% rsm; 84% total isolated yield, 28.9 mg) and **48b'** (6% NMR yield,

<10% rsm; 84% total isolated yield, 28.9 mg) as an inseparable mixture of isomers, as a light-yellow oil. **48b:48b'** = 93:7 (the ratio was determined by $^{1}$H NMR analysis of the isolated products).

**$R_f$** 0.37 [pentane:EtOAc (9:1)].

**Column eluent:** 100 mL pentane → 200 mL pentane:EtOAc (19:1) → 100 mL pentane:EtOAc (9:1).

**$^{1}$H NMR (600 MHz, $CDCl_3$):** δ 7.74 (dd, *J* = 1.9, 0.6 Hz, 0.07H, **48b'**), 7.71 (dd, *J* = 1.8, 0.7 Hz, 1H, **48b**), 7.56 (dd, *J* = 2.4, 0.7 Hz, 1H, **48b**), 7.45 (dd, *J* = 2.3, 0.7 Hz, 0.07H, **48b'**), 7.27 (d, *J* = 7.5 Hz, 0.07H, **48b'**), 7.20 (d, *J* = 8.0 Hz, 1H, **48b**), 7.16 (d, *J* = 7.6 Hz, 0.14H, **48b'**), 7.07 (ddt, *J* = 7.9, 2.0, 0.7 Hz, 1H, **48b**), 6.45 (t, *J* = 2.1 Hz, 0.07H, **48b'**), 6.42 (t, *J* = 2.1 Hz, 1H, **48b**), 2.37 (s, 3H, **48b**), 2.19 (s, 3H, **48b**), 2.01 (s, 0.42H, **48b'**).

**$^{13}$C NMR (151 MHz, $CDCl_3$):** δ 140.19 (**48b**), 140.15 (**48b'**), 138.35 (**48b**), 137.74 (**48b**), 136.34 (**48b'**), 133.59 (**48b**), 131.91 (**48b**), 130.78 (**48b'**), 130.65 (**48b**), 129.03 (**48b'**), 128.22 (**48b'**), 127.20 (**48b**), 126.10 (**48b**), 106.09 (**48b**), 105.95 (**48b'**), 21.15 (**48b**), 17.99 (**48b**), 17.38 (**48b'**).

Data in accordance with the literature.[28]

**1-Mesityl-1*H*-pyrazole (49b)**

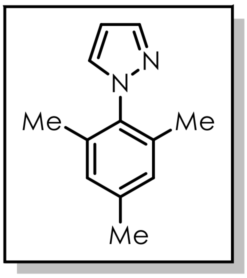


Following **GP2**, **49a** (150 µL, 1.4 mmol, 7 equiv.) and **2a** (13.6 mg, 0.2 mmol, 1.0 equiv.) gave **49b** (90% NMR yield, <5% rsm; 89% isolated yield, 33.2 mg) as a white solid.

**$R_f$** 0.52 [pentane:EtOAc (9:1)]. *Poorly UV-visible, revealed with $KMnO_4$.*

**Column eluent:** 300 mL pentane:EtOAc (97:3).

**$^{1}$H NMR (600 MHz, $CDCl_3$):** δ 7.72 (d, *J* = 1.9 Hz, 1H), 7.43 (d, *J* = 2.3 Hz, 1H), 6.94 (s, 2H), 6.43 (t, *J* = 2.1 Hz, 1H), 2.33 (s, 3H), 1.96 (s, 6H).

**$^{13}$C NMR (151 MHz, $CDCl_3$):** δ 140.07, 138.83, 137.05, 135.97, 130.94, 128.85, 105.83, 21.18, 17.29.

Data in accordance with the literature.[28]

**1-(4-Methylnaphthalen-1-yl)-1*H*-pyrazole (50b) & 1-(4-Methylnaphthalen-2-yl)-1*H*-pyrazole (50b') & 1-(1-Methylnaphthalen-2-yl)-1*H*-pyrazole (50b")**

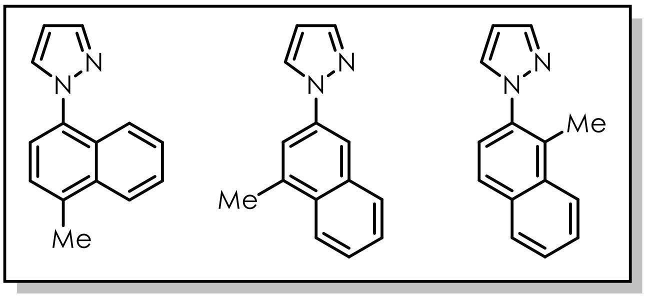


Following **GP2**, **50a** (3 mL, 110 equiv.) and **2a** (13.6 mg, 0.2 mmol, 1.0 equiv.) gave an inseparable mixture of isomers with **50b** (25% NMR yield; 22% total isolated yield, 9.4 mg) as the major isomer (>90% as determined by $^1$H NMR analysis of the isolated products), as a light-yellow oil.

**R*f*** 0.31 [pentane:EtOAc (9:1)].

**Column eluent:** 100 mL pentane → 200 mL pentane:EtOAc (19:1) → 100 mL pentane:EtOAc (9:1).

**$^1$H NMR (600 MHz, $CDCl_3$):** δ 8.06 (dt, *J* = 8.3, 1.0 Hz, 1H), 7.84 – 7.82 (m, 1H), 7.76 (dd, *J* = 2.3, 0.7 Hz, 1H), 7.75 – 7.72 (m, 1H), 7.58 (ddd, *J* = 8.3, 6.8, 1.4 Hz, 2H), 7.51 (ddd, *J* = 8.2, 6.8, 1.2 Hz, 1H), 7.43 (d, *J* = 7.4 Hz, 1H), 7.38 (dd, *J* = 7.4, 1.0 Hz, 1H), 6.53 (t, *J* = 2.1 Hz, 1H), 2.76 (s, 3H).

**$^{13}$C NMR (151 MHz, $CDCl_3$):** δ 140.80, 136.10, 135.79, 133.30, 131.94, 129.57, 126.96, 126.59, 125.87, 124.53, 123.84, 123.16, 106.44, 19.73.

***tert*-Butyl Phenylcarbamate (51b)**

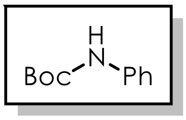


Following **GP1a**, but irradiating the reaction mixture for 6 h, **51a** (23.4 mg, 0.2 mmol, 1.0 equiv.) gave **51b** (47% NMR yield, 29% rsm; 30% isolated yield, 11.7 mg) as a white solid.

**R*f*** 0.61 [pentane:EtOAc (9:1)]. *Poorly UV-visible, revealed with $KMnO_4$.*

**Column eluent:** 200 mL pentane:EtOAc (19:1).

**$^{1}$H NMR (600 MHz, $CDCl_3$):** δ 7.36 (d, *J* = 8.0 Hz, 2H), 7.29 (dd, *J* = 8.6, 7.3 Hz, 3H), 7.08 – 6.99 (m, 1H), 6.47 (s, 1H), 1.52 (s, 10H).

**$^{13}$C NMR (151 MHz, $CDCl_3$):** δ 152.88, 138.46, 129.11, 123.17, 118.65, 28.48.

Data in accordance with the literature.[36]

### *N*-phenylacetamide (52b)

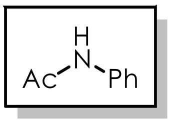

Following **GP1a**, **52a** (11.8 mg, 0.2 mmol, 1.0 equiv.) gave **52b** (20% NMR yield 73% rsm; 15% isolated yield) as a pale-brown solid.

**$R_f$** 0.30 [$CH_2Cl_2$:MeOH (95:5)].

**$^{1}$H NMR (600 MHz, $CDCl_3$):** δ 7.51 (d, *J* = 7.8 Hz, 2 H). 7.30 (t, *J* = 7.7 Hz, 2 H), 7.09 (t, *J* = 7.4 Hz, 1 H), 2.15 (s, 3 H)

**$^{13}$C NMR (151 MHz, $CDCl_3$):** δ 169.32, 138.31, 129.15, 124.52, 120.53, 24.32.

Data in accordance with the literature.[37,38]

## 8.1 Additional Substrate Scope Examples

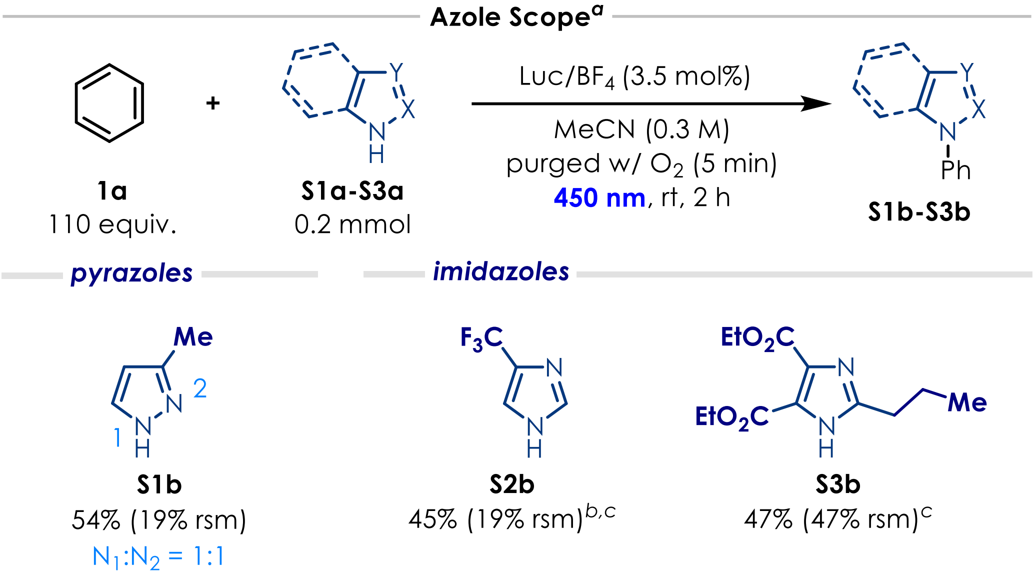


**Figure S6.** Additional Scope of Azoles. [a] Isolated yields are reported. Reactions were performed using 0.2 mmol of heterocycle, arene (2 mL), **Luc/BF$_4$** (3.5 mol %), and MeCN (c = 0.3 M, 0.65 mL) under irradiation in a Penn Photoreactor M2 with a 450 nm light source. [b] The reaction mixture was irradiated for 4 h. [c] HFIP was used as solvent.

### 3-Methyl-1-phenyl-1*H*-pyrazole (S1b) & 5-Methyl-1-phenyl-1*H*-pyrazole (S1b')

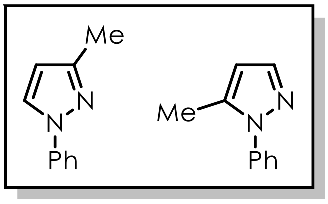

Following **GP1a**, **S1a** (16.1 µL, 0.2 mmol, 1.0 equiv.) gave **S1b** (27% NMR yield, 19% rsm; 24% isolated yield, 14.6 mg) and **S1b'** (27% NMR yield, 19% rsm; 22% isolated yield, 14.0 mg) as a separable mixture of isomers, as light-yellow oils. **S1b: S1b'** = 1:1 (the ratio was determined by crude $^1$H NMR).

Characterisation data for **S1b**:

**R*f*** 0.67 [pentane:EtOAc (9:1)].

**Column eluent:** 100 mL pentane:EtOAc (19:1) → 100 mL pentane:EtOAc (9:1).

**$^1$H NMR (600 MHz, CDCl$_3$):** δ 7.81 (d, *J* = 2.4 Hz, 1H), 7.68 – 7.62 (m, 2H), 7.46 – 7.39 (m, 2H), 7.29 – 7.22 (m, 1H), 6.24 (d, *J* = 2.4 Hz, 1H), 2.38 (s, 3H).

**$^{13}$C NMR (151 MHz, $CDCl_3$):** δ 150.69, 140.36, 129.49, 127.49, 126.06, 118.97, 107.65, 13.88.

Data in accordance with the literature.[27,39]

<u>Characterisation data for **S1b'**</u>:

**R*f*** 0.47 [pentane:EtOAc (9:1)].

**Column eluent:** 100 mL pentane:EtOAc (19:1) → 100 mL pentane:EtOAc (9:1).

**$^{1}$H NMR (600 MHz, $CDCl_3$):** δ 7.58 (d, *J* = 1.7 Hz, 1H), 7.50 – 7.42 (m, 4H), 7.41 – 7.35 (m, 1H), 6.20 (d, *J* = 1.4 Hz, 1H), 2.35 (s, 3H).

**$^{13}$C NMR (151 MHz, $CDCl_3$):** δ 140.05, 139.99, 138.79, 129.17, 127.72, 125.02, 107.00, 12.55.

Data in accordance with the literature.[27,39]

**1-Phenyl-5-(trifluoromethyl)-1*H*-imidazole (*S*2b)**

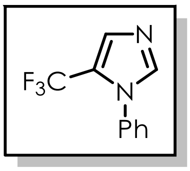


Following **GP1b**, but irradiating the reaction mixture for 4 h, **S2a** (27.2 mg, 0.2 mmol, 1.0 equiv.) gave **S2b** (45% NMR yield, 19% rsm; 25% isolated yield, 10.4 mg) as a light-yellow oil.

**R*f*** 0.32 [pentane:EtOAc (9:1)]. *<u>Note</u>: barely visible by UV. The product streaks on the TLC and seems to decompose with $SiO_2$.*

**Column eluent:** 100 mL pentane:EtOAc (9:1) → 100 mL pentane:EtOAc (8:2).

**$^{1}$H NMR (600 MHz, $CDCl_3$):** δ 7.90 (s, 1H), 7.66 – 7.60 (m, 1H), 7.56 (t, *J* = 7.7 Hz, 2H), 7.53 – 7.46 (m, 1H), 7.46 – 7.38 (m, 2H).

**$^{13}$C NMR (151 MHz, $CDCl_3$)**: δ 136.76, 136.44, 133.83 (q, *J* = 39.2 Hz), 130.33, 128.82, 122.16, 121.54 (d, *J* = 266.8 Hz), 118.57 (q, *J* = 3.9 Hz).

**$^{19}$F NMR (565 MHz, $CDCl_3$):** δ -62.91 (s).

**HRMS (ESI):** found $MH^+$ 213.0632, $C_{10}H_8F_3N_2$ requires 213.0634.

**Diethyl 2-Propyl-1-phenyl-1*H*-imidazole-4,5-dicarboxylate (S3b)**

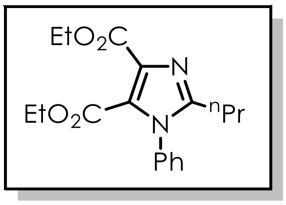


Following **GP1b**, **S3a** (50.8 mg, 0.2 mmol, 1.0 equiv.) gave **S3b** (47% NMR yield, 47% rsm; 53% isolated yield, 35.6 mg) as yellow oil.

**R*f*** 0.50 [pentane:EtOAc (1:1)].

**Column eluent:** 100 mL pentane:$CH_2Cl_2$ (1:1) → 100 mL pentane:EtOAc (8:2) → 200 mL pentane:EtOAc (6:4).

**$^1$H NMR (600 MHz, $CDCl_3$):** δ 7.49 (dd, *J* = 5.2, 1.9 Hz, 3H), 7.25 – 7.20 (m, 2H), 4.39 (q, *J* = 7.1 Hz, 2H), 4.10 (q, *J* = 7.1 Hz, 2H), 2.49 (t, *J* = 7.8 Hz, 2H), 1.62 (h, *J* = 7.3 Hz, 2H), 1.37 (t, *J* = 7.2, 3H), 1.05 (t, *J* = 7.1, 3H), 0.82 (t, *J* = 7.4, 3H).

**$^{13}$C NMR (151 MHz, $CDCl_3$):** δ 162.81, 159.95, 151.52, 135.78, 134.57, 129.70, 129.48, 127.93, 127.37, 61.58, 61.34, 29.07, 21.56, 14.39, 13.85, 13.77.

**HRMS (ESI):** found $MH^+$ 331.1650, $C_{18}H_{23}N_2O_4$ requires 331.1652.

# 9 UV-Vis absorption studies

## 9.1 UV-Vis spectra and Beer-Lambert law

Various stock solutions of the corresponding photocatalyst (*i.e.* **Luc/BF$_4$** or **Luc/NO$_3$**) were prepared under air at the corresponding concentration using a volumetric flask. UV/Vis absorption spectra were recorded on an Agilent Cary 8454 spectrophotometer or a JASCO V-770 in a 1 cm quartz cuvette.

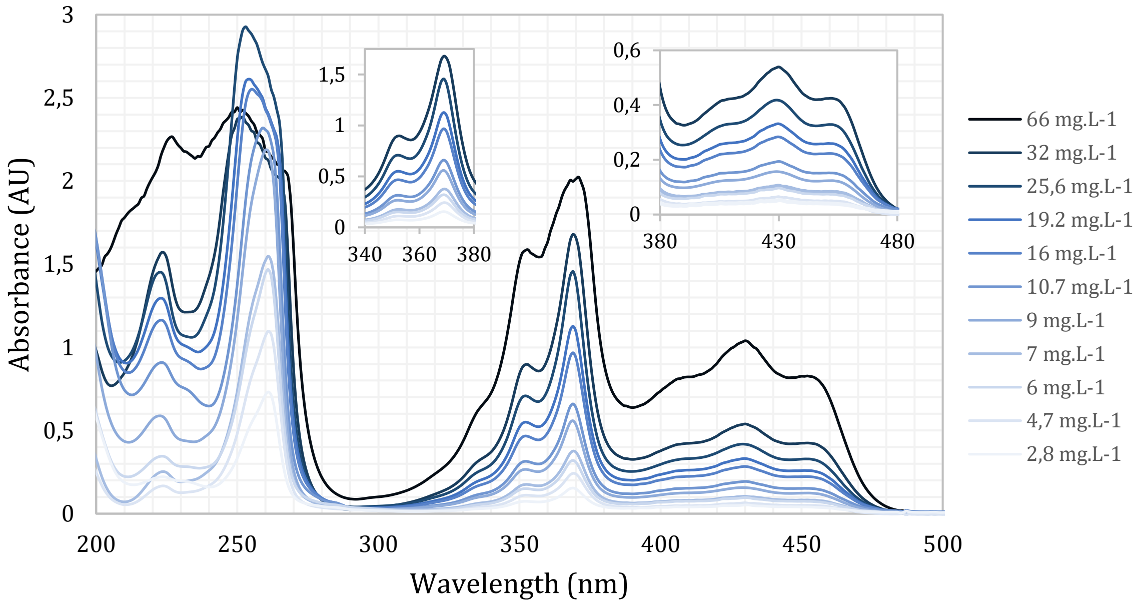


**Figure S7.** UV-vis spectra of **Luc/BF$_4$** (from 2.8 mg/L to 66 mg/L) in MeCN. Measured in a 1 cm quartz cuvette.

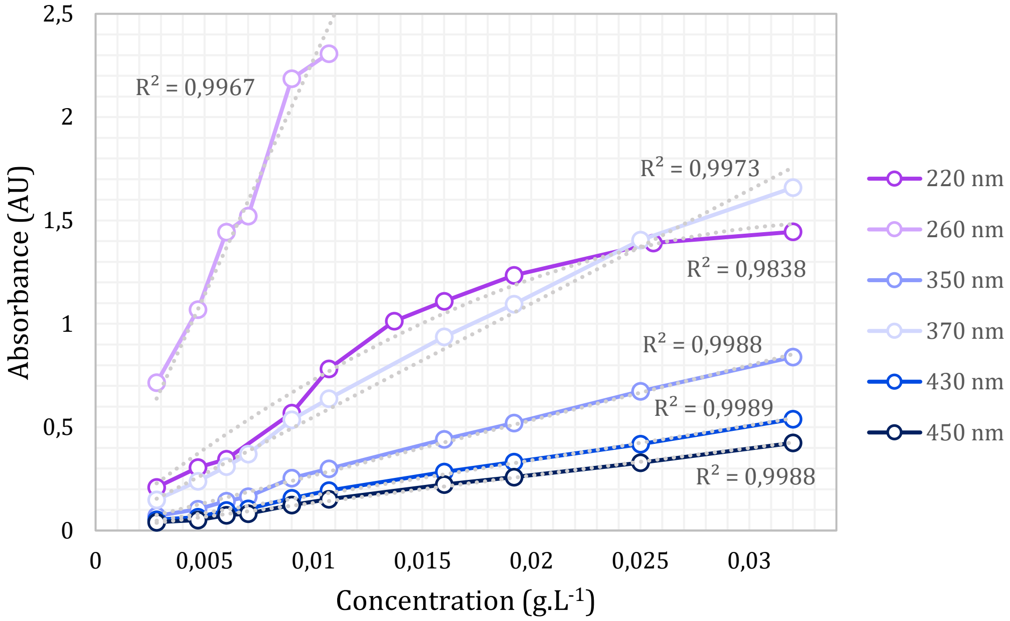


**Figure S8.** Beer-Lambert law and corresponding linear fits for local maxima in the UV-Vis spectrum.

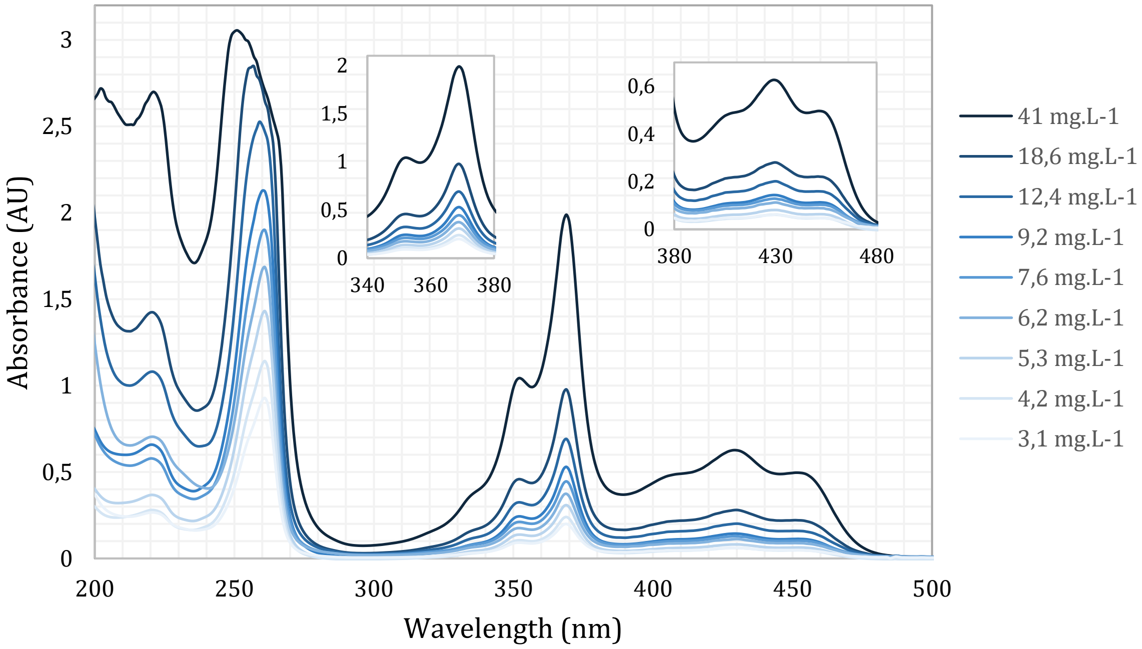


**Figure S9.** UV-vis spectra of **Luc/NO$_3$** (from 3.1 mg/L to 41 mg/L) in MeCN. Measured in a 1 cm quartz cuvette.

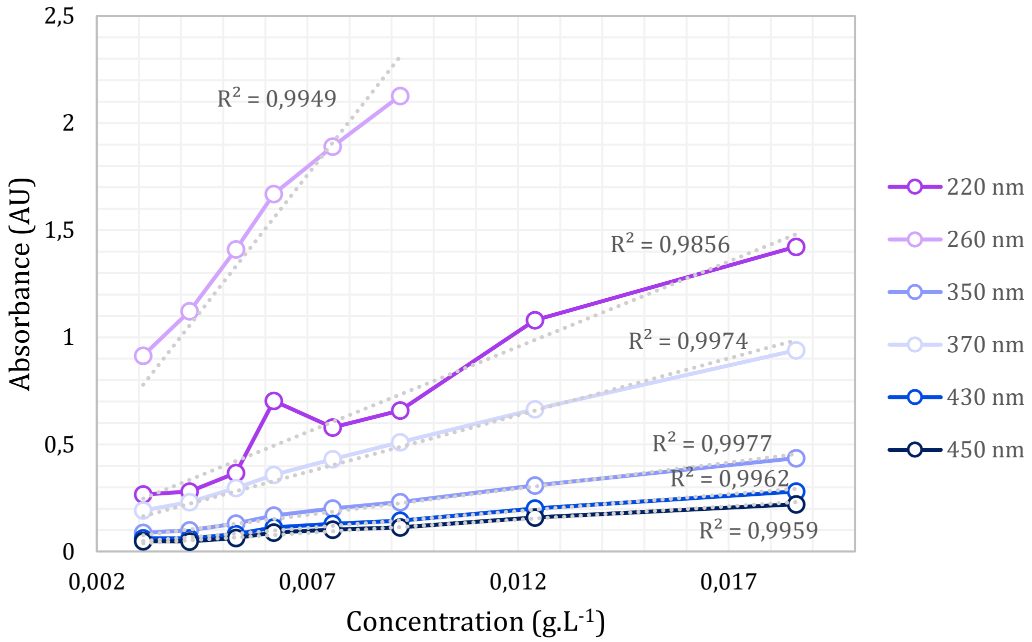


**Figure S10.** Beer-Lambert law and corresponding linear fits for local maxima in the UV-Vis spectrum.

### 9.2 Solvent effect on the UV-Vis absorption of Luc/BF$_4$

Various stock solutions of **Luc/BF$_4$** were prepared under air with the corresponding solvent using a volumetric flask. The concentrations used were 1.6 mg.L$^{-1}$ for MeCN and 1.5 mg.L$^{-1}$ for the remaining solvents. UV/Vis absorption spectra were recorded on an Agilent Cary 8454 spectrophotometer or a JASCO V-770 in a 1 cm quartz cuvette.

*Note: In acetone and MeOH, the solution was sonicated for at least 5 min to ensure complete dissolution of the solid. Relatively apolar solvents such as $CH_2Cl_2$, toluene, benzene, THF, 1,4-dioxane could not be used as **Luc/BF$_4$** was partially or completely insoluble.*

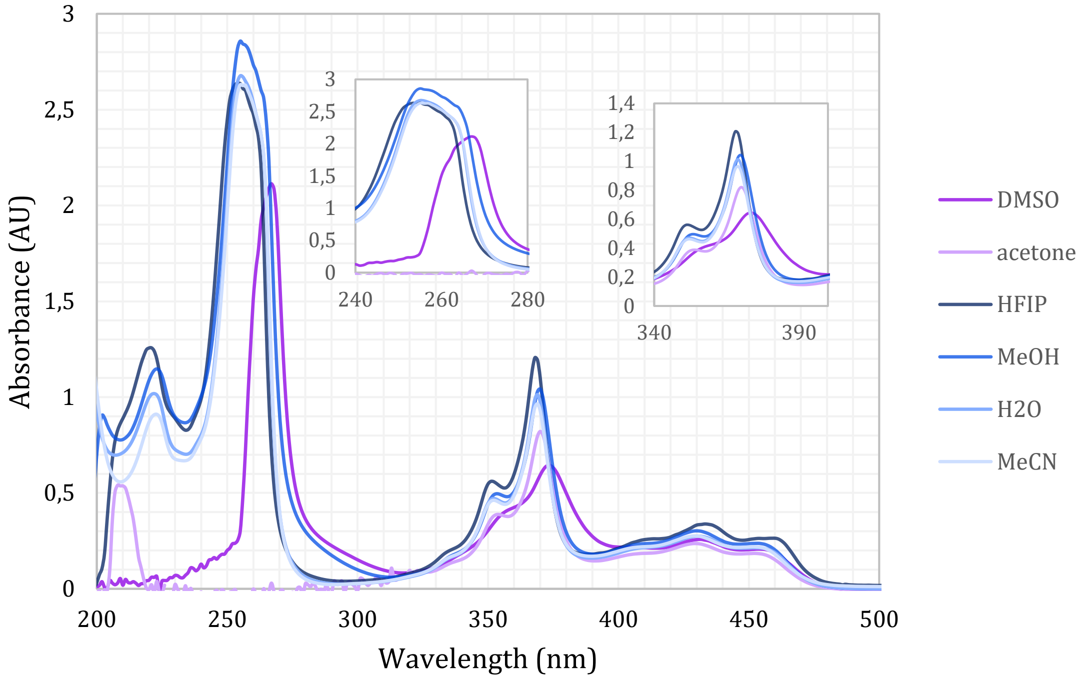


**Figure S11.** Influence of various solvents on the UV-Vis profile of **Luc/$BF_4$**. Spectrum between 240-280 nm and 340-390 nm is magnified.

### 9.3 EDA between benzene and Luc/$BF_4$ or pyrazole and Luc/$BF_4$

For the study regarding a plausible EDA complex between the photocatalyst and benzene or the photocatalyst and pyrazole, stock solutions of **Luc/$BF_4$** in MeCN (32 $mg.L^{-1}$ or 14 $mg.L^{-1}$), benzene in MeCN (34 mM) and pyrazole in MeCN (0.79 mM) were used. UV/Vis absorption spectra were recorded on an Agilent Cary 8454 spectrophotometer or a JASCO V-770 in a 1 cm quartz cuvette.

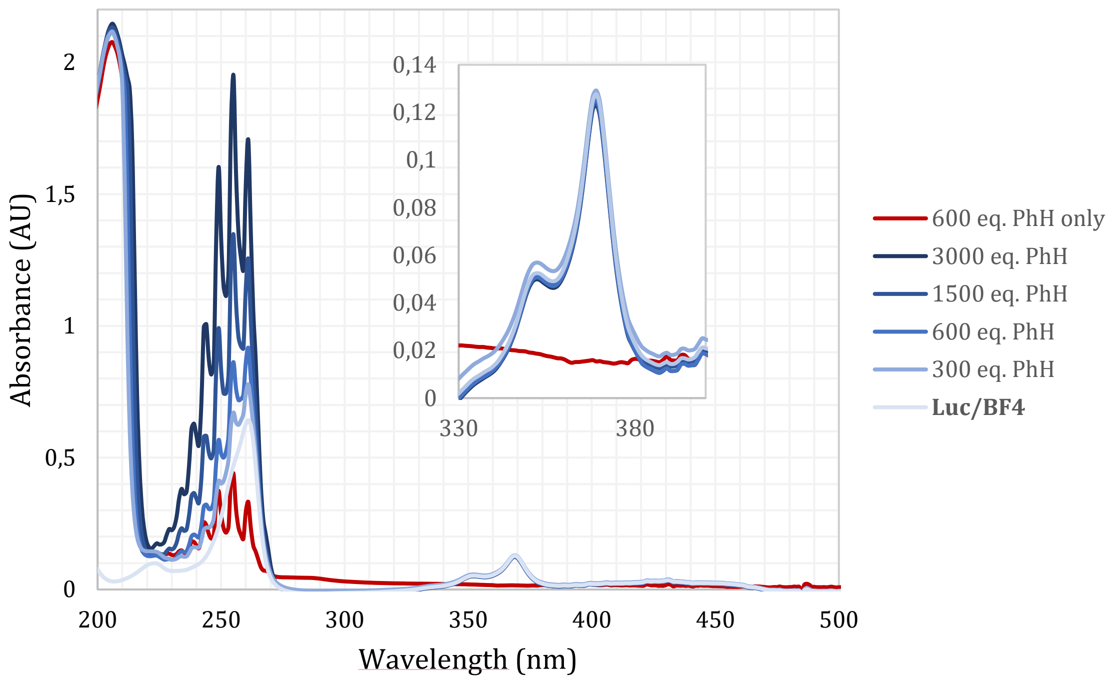


**Figure S12.** Effect on the UV-Vis profile of **Luc/BF$_4$** by adding an increasing amount of benzene, reflecting the amount present in the model reaction. Spectrum between 330-390 nm is magnified.

For benzene, 0.2 mL of the **Luc/BF$_4$** stock solution (32 mg.L$^{-1}$) was added to the cuvette followed by 0.1 mL, 0.2 mL, 0.5 mL and 1 mL of the benzene stock solution. The cuvette was finally filled with MeCN until a total volume of 3 mL of solvent was obtained. Such a large excess of benzene was used to mimic the actual experimental conditions.

Between 300 nm and 500 nm (where benzene is not absorbing), no discernible change was observed regarding the relative absorption of **Luc/BF$_4$**. This result seems to indicate the absence of an EDA complex between **Luc/BF$_4$** and benzene.

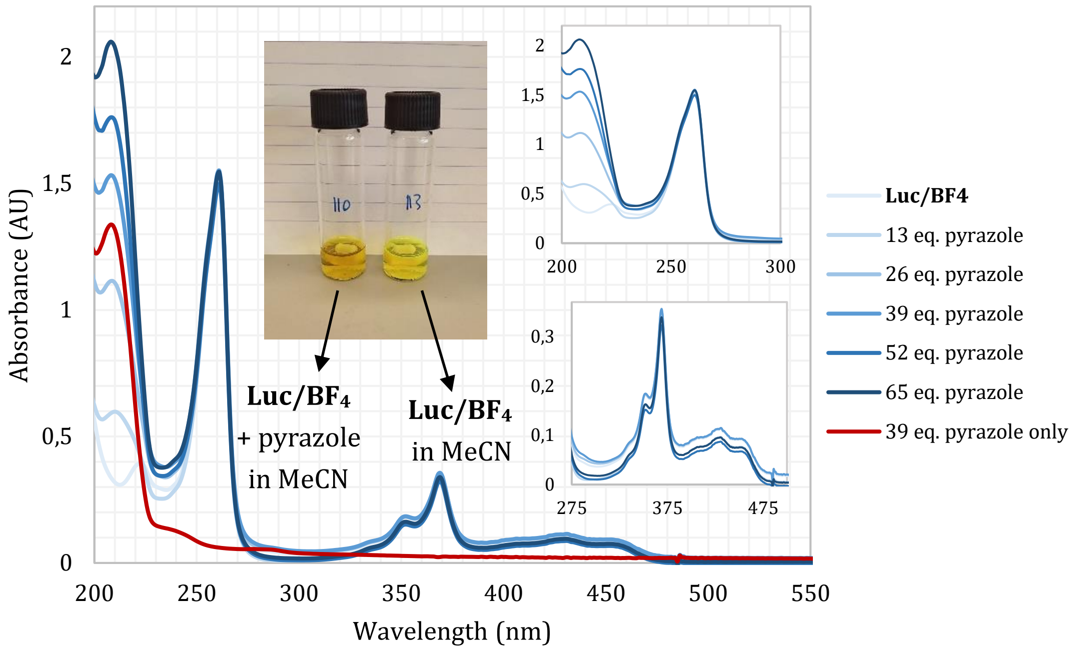


**Figure S13.** Effect on the UV-Vis profile of **Luc/BF$_4$** by adding an increasing amount of pyrazole. Spectrum between 200-300 nm and 275-475 nm is magnified. A picture representing two reaction vials containing **Luc/BF$_4$** and pyrazole (on the left; with the same amount as for the model reaction) and only **Luc/BF$_4$** (on the right).

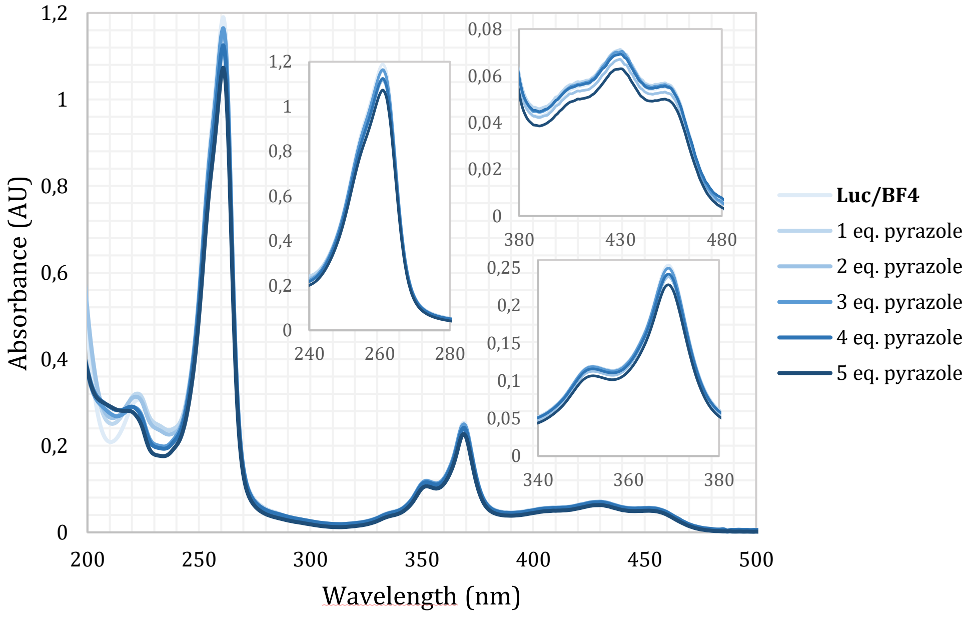


**Figure S14.** Effect on the UV-Vis profile of **Luc/BF$_4$** by adding an increasing amount of pyrazole. Spectrum between 240-280 nm, 340-380 nm and 380-480 nm is magnified.

For the spectra overlay in **Figure S13**, 0.5 mL of the **Luc/BF$_4$** stock solution (32 mg.L$^{-1}$) was added to the cuvette followed by 0.5 mL, 1 mL, 1.5 mL, 2 mL and 2.5 mL of the pyrazole stock solution. For the spectra overlay in **Figure S14**, 1 mL of the **Luc/BF$_4$** stock

solution (14 mg.L$^{-1}$), was added to the cuvette followed by 31.5 µL, 63 µL, 94.5 µL, 126 µL and 157.5 µL of the pyrazole stock solution. The cuvette was finally filled with MeCN until a total volume of 3 mL of solvent was obtained.

From the picture shown in **Figure S13**, a visible colour change can be observed when pyrazole is added to the solution of photocatalyst in MeCN. However, between 240 nm and 500 nm (where pyrazole is mostly not absorbing), no discernible change was observed regarding the relative absorption of **Luc/BF$_4$**. This result seems to indicate the absence of an EDA complex between **Luc/BF$_4$** and pyrazole.

### 9.4 EDA between benzene and pyrazole

For the study regarding a plausible EDA complex between pyrazole and benzene, stock solutions of pyrazole in MeCN (72 mM) and benzene in MeCN (56 mM mM) were used. UV/Vis absorption spectra were recorded on an Agilent Cary 8454 spectrophotometer or a JASCO V-770 in a 1 cm quartz cuvette.

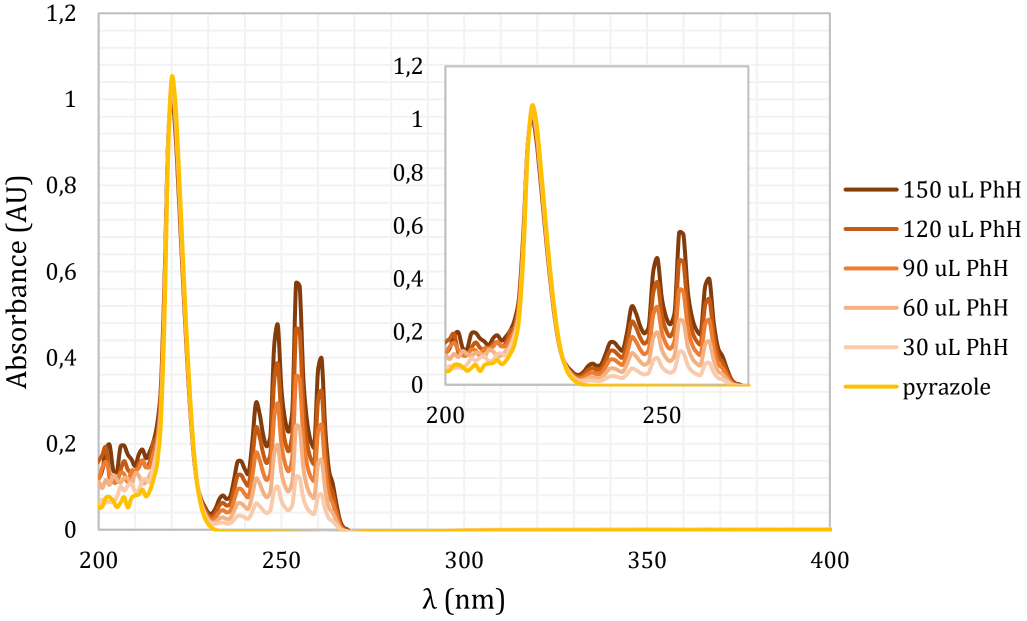


**Figure S15.** Effect on the UV-Vis profile of pyrazole (*c* = 1.1 mM) by adding an increasing amount of benzene (from a 56 mM solution). Spectrum between 200-270 nm is magnified.

For benzene, 30 µL of the pyrazole stock solution was added to the cuvette followed by 30 µL, 60 µL, 90 µL, 120 µL and 150 µL of the benzene stock solution. The cuvette was finally filled with 2 mL MeCN.

Between 200 nm and 300 nm, no discernible change was observed regarding the relative absorption of pyrazole (**Figure S15**). This result seems to indicate the absence of an EDA complex between pyrazole and benzene.

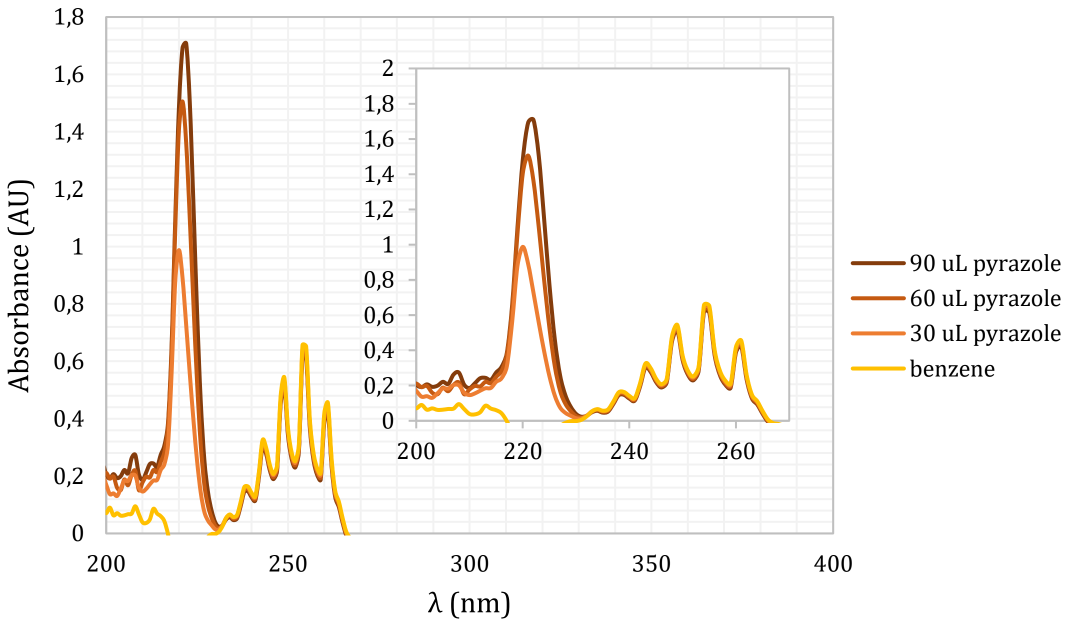


**Figure 16.** Effect on the UV-Vis profile of benzene ($c$ = 5 mM) by adding an increasing amount of pyrazole (from a 72 mM solution). Spectrum between 200-270 nm is magnified.

For pyrazole, 180 µL of the benzene stock solution was added to the cuvette followed by 30 µL, 60 µL and 90 µL of the pyrazole stock solution. The cuvette was finally filled with 2 mL MeCN.

Between 200 nm and 300 nm, no discernible change was observed regarding the relative absorption of benzene (**Figure S16**). This result seems to indicate the absence of an EDA complex between pyrazole and benzene.

### 9.5 UV-Vis spectra of Luc/BF$_4$ before and after irradiation

Stock solutions of **Luc/BF$_4$** in MeCN (32 mg.L$^{-1}$), benzene in MeCN (34 mM) and pyrazole in MeCN (0.79 mM) were used. UV/Vis absorption spectra were recorded on an Agilent Cary 8454 spectrophotometer or a JASCO V-770 in a 1 cm quartz cuvette.

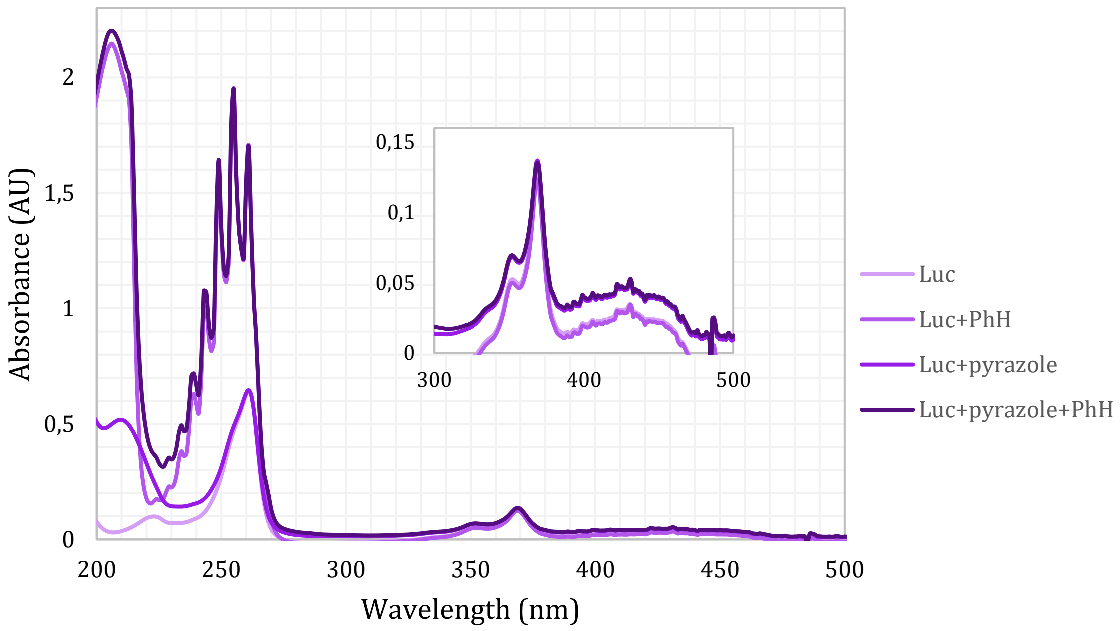


**Figure S17.** Effect of the addition of benzene, pyrazole and pyrazole+benzene (respectively) on the UV-Vis profile of **Luc/BF$_4$**.

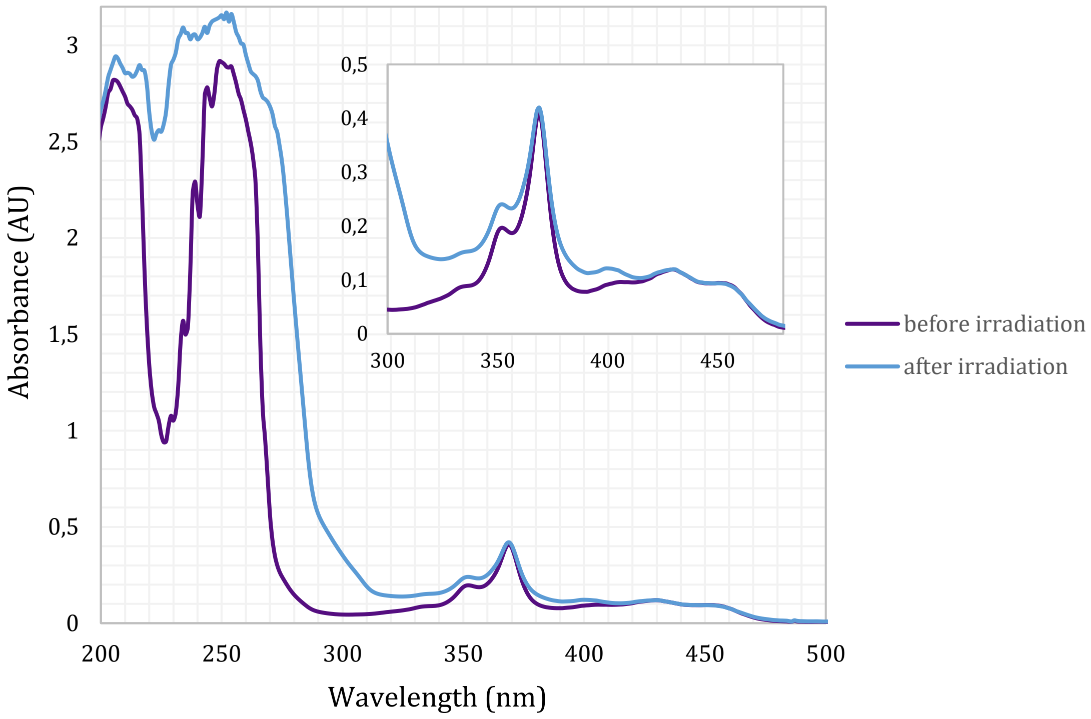


**Figure S18.** Effect of the irradiation on the UV-Vis profile of **Luc/BF$_4$**.

# 10 Cyclic Voltammetry (CV) Experiments

All electrochemical experiments were carried out using a three-electrode setup using a Palmsense 4 potentiostat. Pt wire was used as a counter electrode throughout the measurements. Unless otherwise stated a glassy carbon (GC) disk working electrode (3 mm diameter) was used. Prior to each experiment the working electrode surface was polished using a 0.05 µm alumina slurry. In organic solvents a non-aqueous Ag/AgCl reference electrode (was employed. Unless otherwise stated, a scan rate of 100 mV/s was used for all CV experiments, and experiments were carried out under ambient conditions in HPLC grade solvents and not in an air-tight setup. Depending on measurement, the solution was sparged for 1 minute with either $N_2$ or $O_2$. $TBAPF_6$ was used as supporting electrolyte in all cases (100 mM for standard voltametric experiments) and 2.5 mL solutions were prepared for measurements. Unless otherwise stated, all measurements were started from 0 V vs Ag/AgCl and performed in the anodic direction first. No iR compensation was carried out. All CV measurements were carried out by measuring 3 cycles. For analysis purposes, scan 2 was used unless otherwise stated.

## 10.1 Cyclic voltammograms of Luc/$BF_4$ in MeCN

Cyclic voltammograms of **Luc/$BF_4$** were measured both in the absence and presence of oxygen for 3 scans in each case. In the presence of oxygen, we observe an increased current at -1.0 V vs Ag/AgCl suggesting superoxide formation.

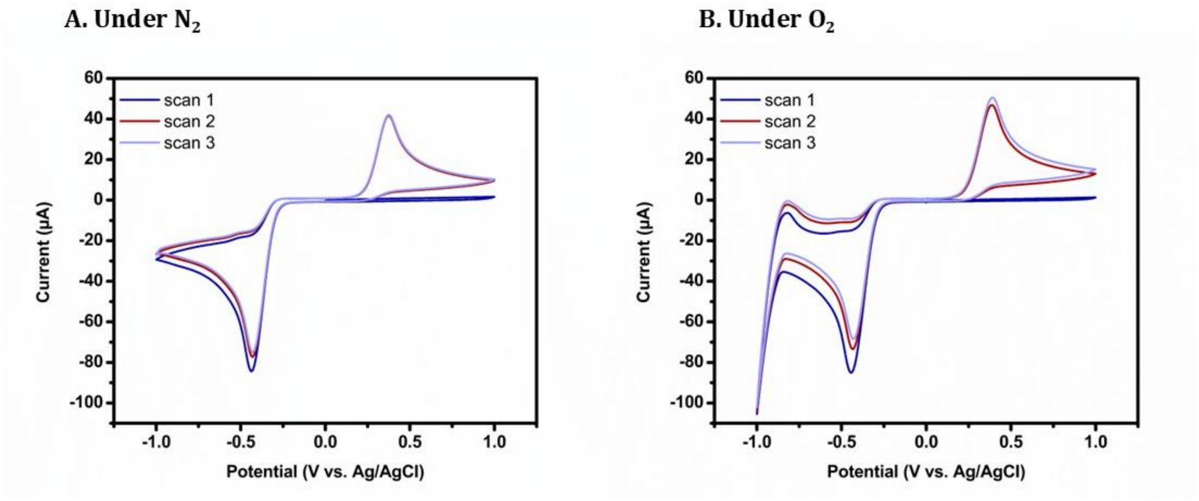


**Figure S19.** CV of **Luc/BF$_4$** in MeCN, 100 mM TBAPF$_6$ under (A) $N_2$ and (B) $O_2$ atmosphere, anodic scanning direction. The standard setup and scanning parameters were used.

The direction of the cyclic voltammogram suggests that scanning in the anodic direction first, there is no species that can be oxidized, thus, we only observe an oxidation signal only in the second scan. When scanning in the cathodic direction, reduction occurs first, thus, we observe a full redox cycle in the first scan.

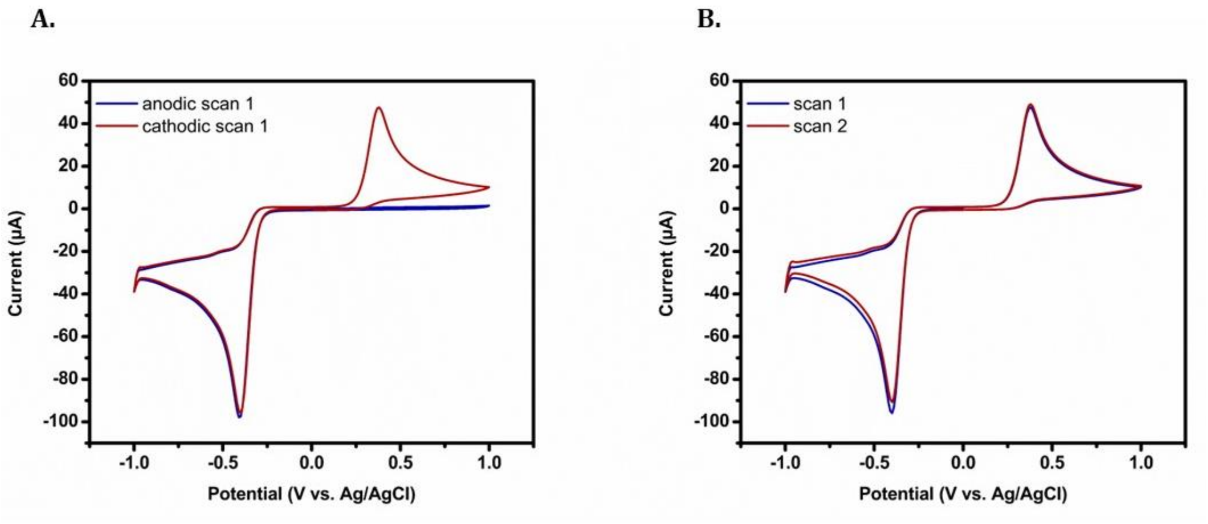


**Figure S20.** CV of **Luc/BF$_4$** in MeCN under N2 atmosphere, 100 mM TBAPF$_6$ comparing (A) Scan 1 based on anodic (blue) and cathodic (red) scanning direction and (B) Scan 1 and 2 when scanning cathodic direction first. The standard setup and scanning parameters were used.

Based on the scan rate variation in both $N_2$ and $O_2$ atmosphere, we observe a quasi-reversible redox cycle, with increasing currents as scanning rate increases. The reversibility trend does not change based on atmosphere, only the current magnitudes.

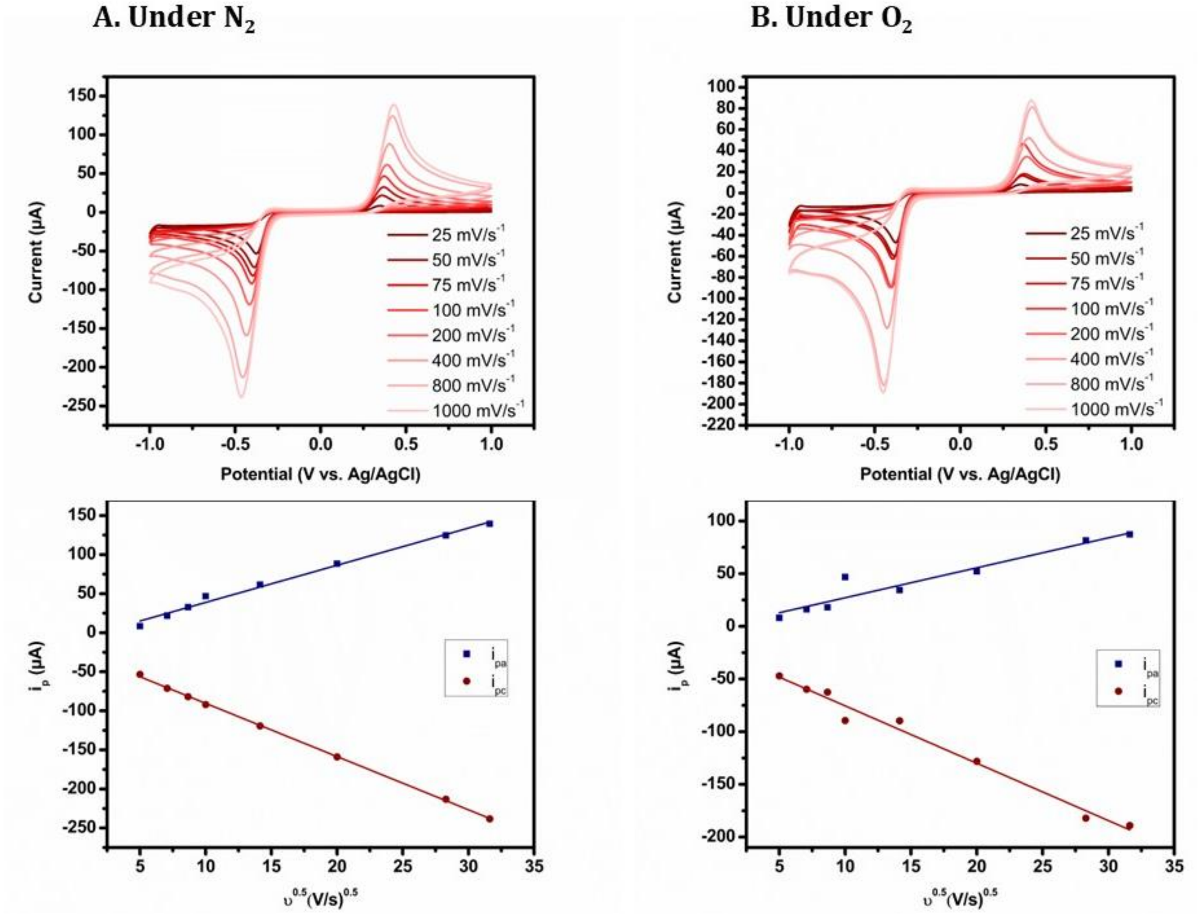


**Figure S21.** CV of **Luc/BF₄** in MeCN, 100 mM $TBAPF_6$ under (A) $N_2$ and (B) $O_2$ atmosphere comparing scan rate variation (top) and linear fits of the anodic (dark blue) and cathodic (dark red) peak currents (bottom). The standard setup was used while scan rate was varied between 25 mV/s and 1000 mV/s. Between measurements, respective atmosphere purging was performed for 1 minute. The linear fits of the anodic and cathodic peak currents are depicted as a function of the square root of the scan rate.

When longevity tests are performed, we only observe a small decrease in current values between the second scan (the first full redox scan) and the 20th scan in both $N_2$ and $O_2$ atmosphere, confirming the stability of the catalyst.

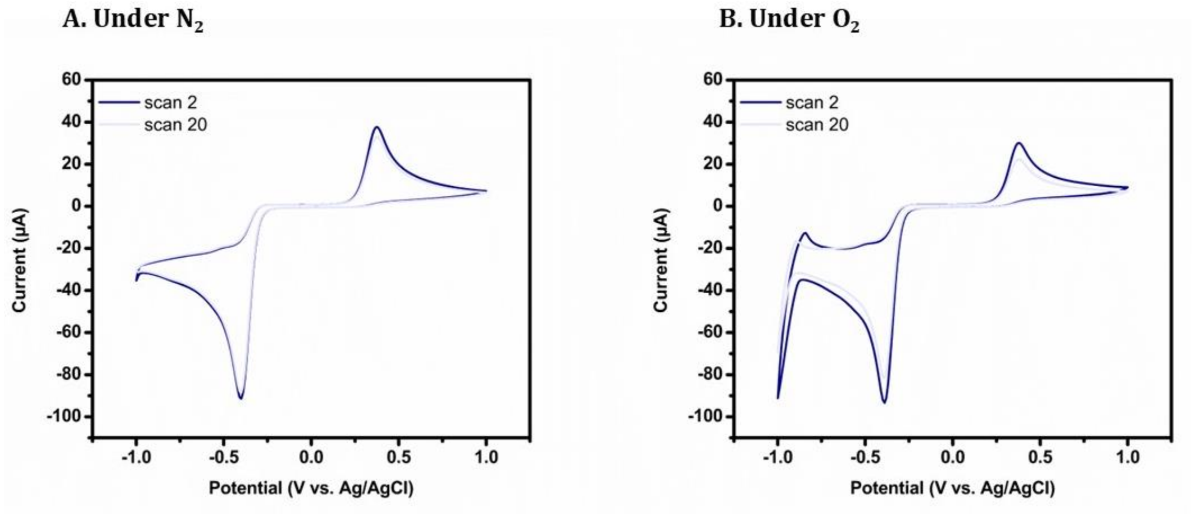


**Figure S22.** CV longevity experiments of **Luc/BF₄** in MeCN, 100 mM $TBAPF_6$ in (A) $N_2$ atmosphere and (B) $O_2$ atmosphere respectively, comparing scan 2 (dark blue) and scan 20 (light purple). The standard setup and scanning parameters were used and no additional purging between measurements was performed.

## 10.2 Cyclic voltammograms of Luc/$NO_3$ in MeCN

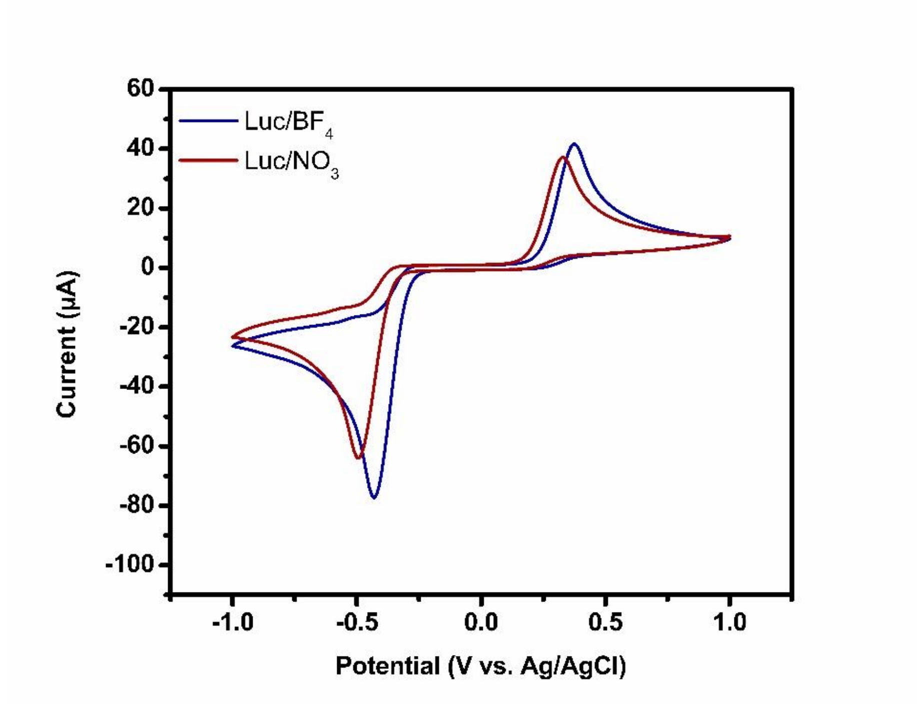


**Figure S23.** CV of **Luc/$BF_4$** vs **Luc/$NO_3$** in MeCN, 100 mM $TBAPF_6$ under $N_2$ atmosphere, anodic scanning direction. The standard setup and scanning parameters were used.

Contrary to the observed stability of **Luc/$BF_4$** over 20 scanning cycles, **Luc/$NO_3$** exhibits large decrease in current over 20 scanning cycles.

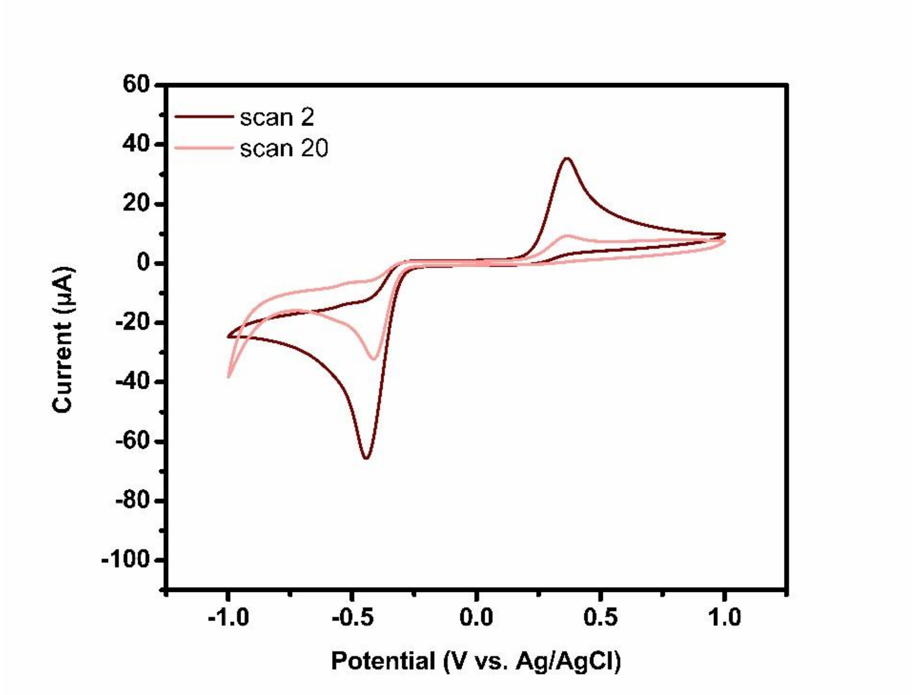


**Figure S24.** CV longevity experiments of **Luc/NO$_3$** in MeCN, 100 mM TBAPF$_6$ comparing scan 2 (dark red) and scan 20 (light pink) under $N_2$ atmosphere. The standard setup and scanning parameters were used and no additional purging between measurements performed.

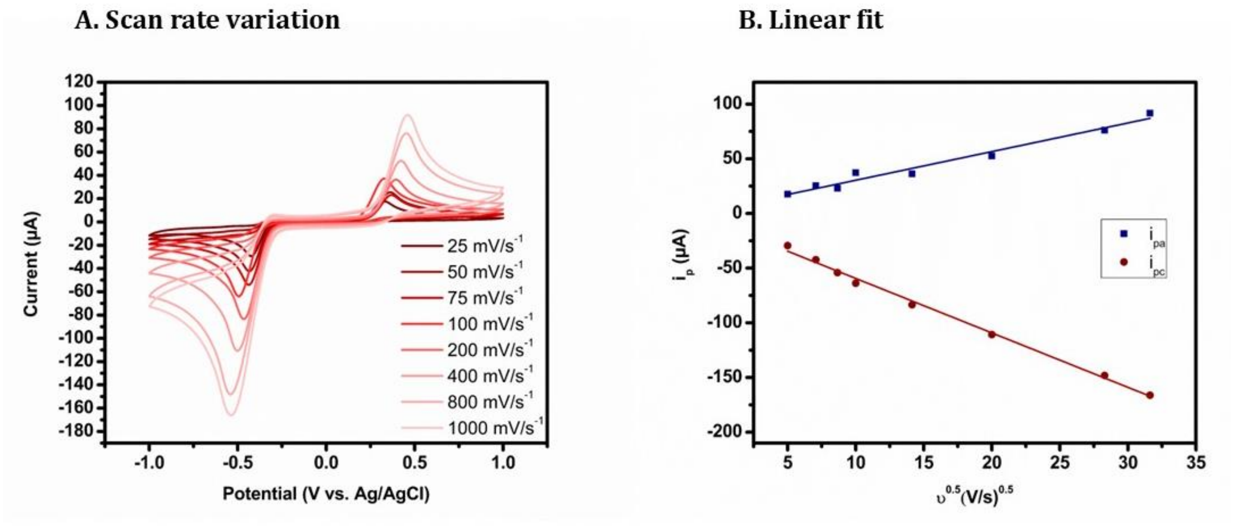


**Figure S25.** CV of **Luc/NO$_3$** in MeCN, 100 mM TBAPF$_6$ comparing (A) scan rate variation under $N_2$ atmosphere and (B) linear fits of the anodic (dark blue) and cathodic (dark red) peak currents as a function of the square root of the scan rate. The standard setup was used while scan rate was varied between 25 mV/s and 1000 mV/s. Between measurements, purging with $N_2$ was performed for 1 minute. The linear fits of the anodic and cathodic peak currents are depicted as a function of the square root of the scan rate.

### 10.3 Cyclic voltammograms of Luc/$BF_4$ in HFIP

In HFIP, **Luc/$BF_4$** exhibits generally lower currents than in MeCN and the redox features are less prominent.

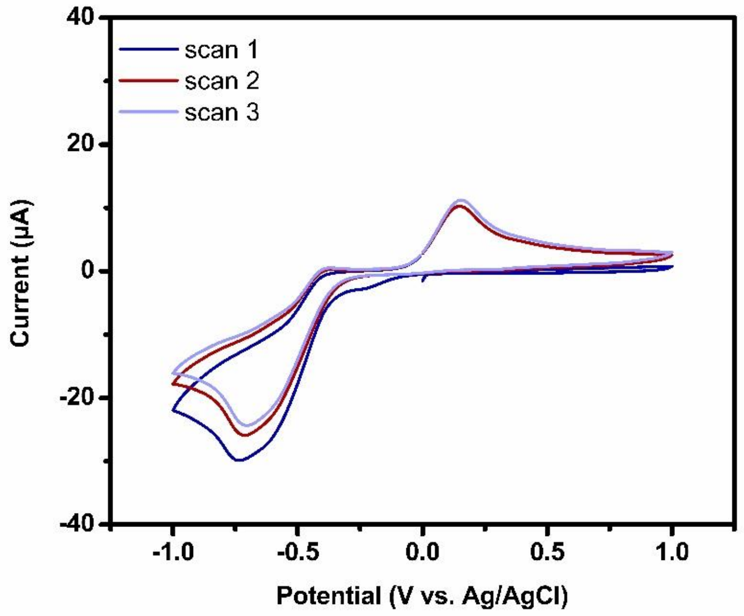


**Figure S26.** CV of **Luc/$BF_4$** in HFIP, 100 mM $TBAPF_6$ under $N_2$ atmosphere, anodic scanning direction. The standard setup and scanning parameters were used.

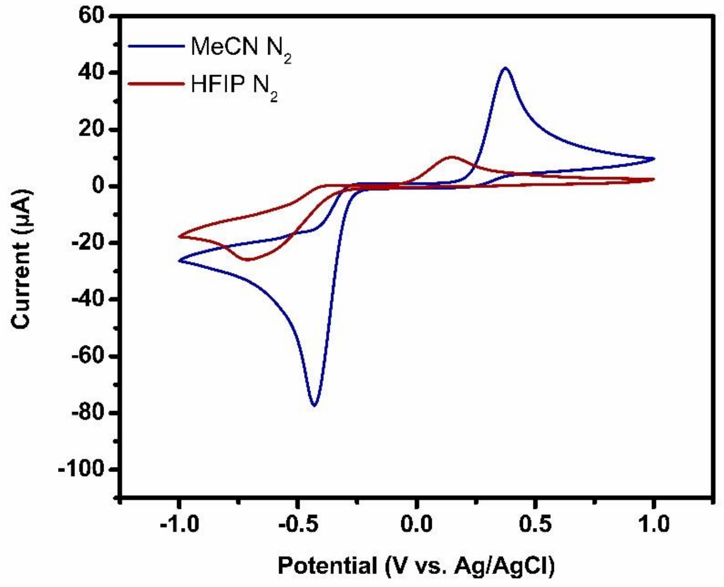


**Figure S27.** CV of **Luc/$BF_4$** in MeCN (dark blue) vs HFIP (dark red), 100 mM $TBAPF_6$ under $N_2$ atmosphere, anodic scanning direction. The standard setup and scanning parameters were used.

## 10.4 Cyclic voltammograms of different azoles and arenes in MeCN

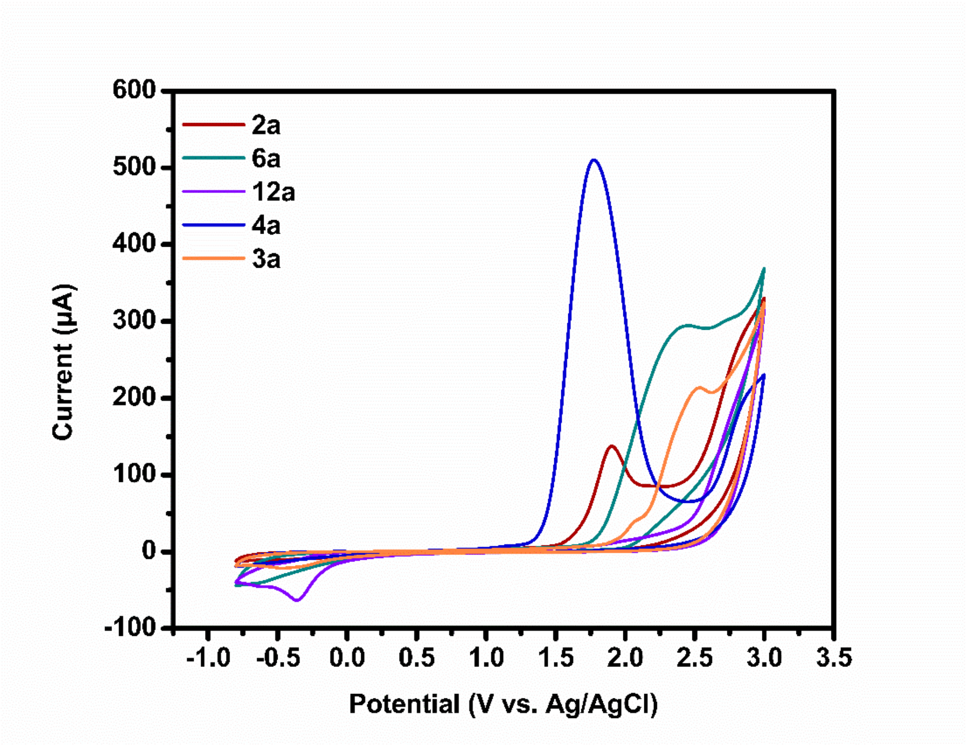


**Figure S28.** CV measurements of different azoles in MeCN, 100 mM $TBAPF_6$ under $N_2$ atmosphere, anodic scanning direction. The standard setup and scanning parameters were used.

Respective Epa values for the azoles were determined as follow:

- **2a** $(E_{ox}^{1/2} = 1.89\ V\ vsAg/AgCl\ or\ 1.85\ V\ vs\ SCE)$
- **6a** $(E_{ox}^{1/2} = 2.45\ V\ vs\ Ag/AgCl\ or\ 2.41\ V\ vs\ SCE)$
- **12a** $(E_{ox}^{1/2}\ is\ outside\ solvent\ potential\ window)$
- **4a** $(E_{ox}^{1/2} = 1.82\ V\ vs\ Ag/AgCl\ or\ 1.78\ V\ vs\ SCE)$
- **3a** $(E_{ox}^{1/2} = 2.54\ V\ vs\ Ag/AgCl\ or\ 2.50\ V\ vs\ SCE)$.

## 11 Fluorescence, emission spectra and determination of $E^{*}_{red}$

Fluorescence spectra were recorded on a JASCO FP-6200 or FS5 v2 Spectrofluorometer from Edinburgh Instruments. Fluorescence spectra were recorded using 5 nm excitation and 2 nm emission slit widths. A 1 cm High Precision Cell made of quartz from Hellma Analytics (117.100F-QS 10x10 mm, macro fluorescence sealable cuvette with an open cap with Silicone rubber seal) was used for all the measurements.

Emission spectra of compound **Luc/BF$_4$** (15 µL in 2.5 mL of MeCN; 14 mg.L$^{-1}$) were recorded in MeCN from 450 to 650 nm with an excitation wavelength of 375 (or 430) nm.

The data obtained were normalized and plotted as shown in **Figure S29**.

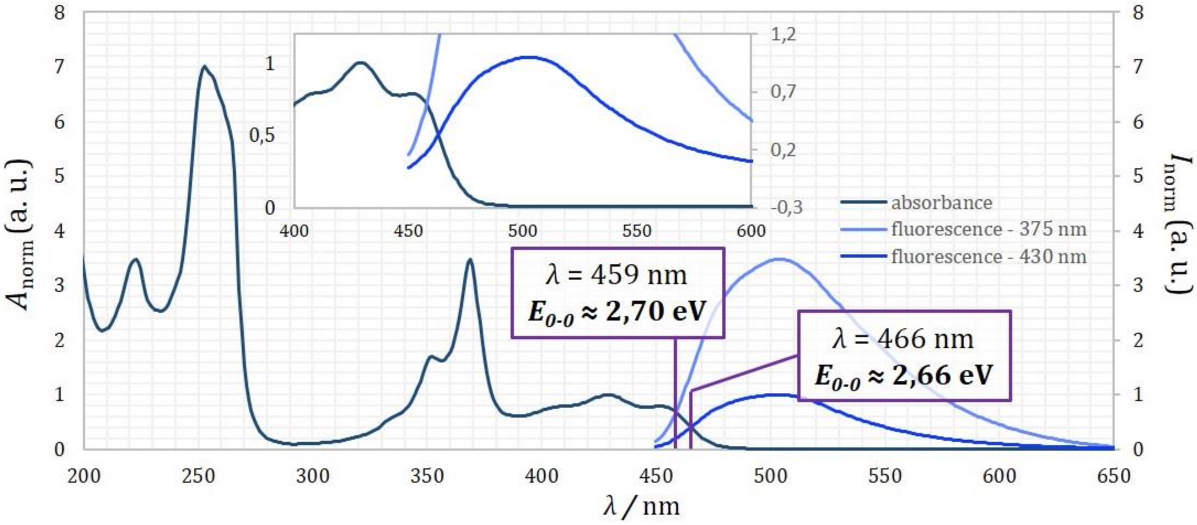


**Figure S29.** Calibrated UV–Vis absorption spectra and normalized emission spectra of **Luc/BF$_4$** in MeCN, along with $E_{0\text{-}0}$ determination.

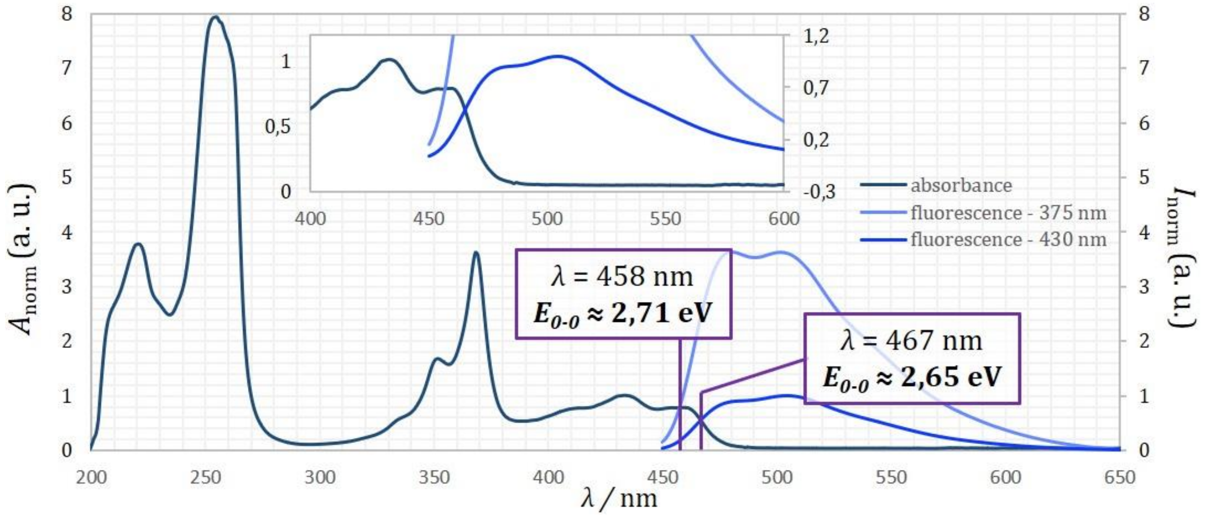


**Figure S30.** Calibrated UV−Vis absorption spectra and normalized emission spectra of **Luc/BF$_4$** in HFIP, along with $E_{0\text{-}0}$ determination.

The Rehm-Weller equation for the calculation of the excited state potential is given by the following equation:

$$E^*_{red} = E^{1/2}_{red} + E_{0\text{-}0}$$

Where, $E^*_{red}$ is the excited-state reduction potential of **Luc/BF$_4$** in V, $E^{1/2}_{red}$ is the ground-state redox potential in V (known by experiment, see **part 10.4**) and $E_{0\text{-}0}$ refers to the transition between the lowest energy vibrational state ($v$ = 0) of $S_1$ to $v$ = 0 of $S_0$ in eV.

For **Luc/BF$_4$** in MeCN,

$$E^{1/2}_{red} = -0.429\ V (from\ CV\ vs\ Ag/AgCl\ in\ MeCN)$$

$$E_{0\text{-}0} = 2.70\ eV\ (for\ 375\ nm\ in\ MeCN)$$

$$E_{0\text{-}0} = 2.66\ eV\ (for\ 430\ nm\ in\ MeCN)$$

Therefore,

$$E^*_{red} = 2.27\ V\ vs\ Ag/AgCl\ (for\ 375\ nm\ in\ MeCN)$$

$$E^*_{red} = 2.23\ V\ s\ Ag/AgCl\ (for\ 430\ nm\ in\ MeCN)$$

Converted to $V\ vs\ SCE$,

$$E^*_{red} = 2.23\ V\ vs\ SCE\ (for\ 375\ nm\ in\ MeCN)$$

$$E^{*}_{red} \ = \ 2.19\,V\ s\ SCE\ (for\ 430\ nm\ in\ MeCN)$$

For **Luc/BF$_4$** in HFIP,

$$E^{1/2}_{red} \ = \ -0.709\ (from\ CV\ vs\ Ag/AgCl\ in\ HFIP)$$

$$E_{0\text{-}0} \ = \ 2.71\ eV\ (for\ 375\ nm\ in\ HFIP)$$

$$E_{0\text{-}0} \ = \ 2.65\ eV\ (for\ 430\ nm\ in\ HFIP)$$

Therefore,

$$E^{*}_{red} \ = \ 2.00\,V\ vs\ Ag/AgCl\ (for\ 375\ nm\ in\ HFIP)$$

$$E^{*}_{red} \ = \ 1.96\,V\ vs\ Ag/AgCl\ (for\ 430\ nm\ in\ HFIP)$$

Converted to $V\ vs\ SCE$,

$$E^{*}_{red} \ = \ 1.96\,V\ vs\ SCE\ (for\ 375\ nm\ in\ HFIP)$$

$$E^{*}_{red} \ = \ 1.92\,V\ vs\ SCE\ (for\ 430\ nm\ in\ HFIP)$$

## 12 Initial fluorescence quenching experiments and Stern-Volmer studies

Fluorescence quenching spectra were recorded on a FS5 v2 Spectrofluorometer from Edinburgh Instruments. Fluorescence spectra were recorded using 5 nm excitation and 2 nm emission slit widths. The emission scan was carried out between 450 nm and 650 nm using a step of 0.5 nm and a dwell time of 0.3 s. A 1 cm High Precision Cell made of quartz from Hellma Analytics (117.100F-QS 10x10 mm, macro fluorescence sealable cuvette with an open cap with Silicone rubber seal) was used for all the measurements. The quenching studies were recorded in MeCN as solvent, and the background was automatically subtracted in the spectrofluorometer software Fluoracle®. All solvents and stock solutions were degassed and kept under $N_2$ atmosphere prior to measuring spectra. Prior to adding the respective quenchers, a solution of **Luc/BF$_4$** (15 µL in 2.5 mL of solvent; 14 mg.L$^{-1}$) was measured first. Then quenchers were added in the following steps 10 µL, 10 µL, 10 µL, 10 µL, 10 µL, 20 µL, 20 µL (to a total of 90 µL). The data is shown as both quenching series and linear fit for the calculation of quenching rates as follows.

## 12.1 Stern-Volmer quenching in MeCN

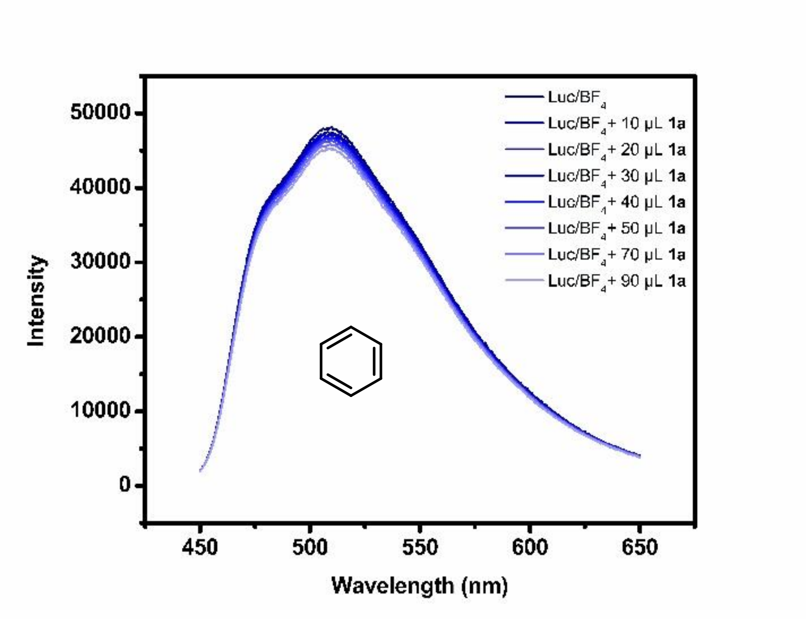


**Figure S31.** Fluorescence emission spectra (Ex. 375) of compound **Luc/BF₄** (15 µL of 14 mg.L⁻¹ stock solution) in MeCN (2.5 mL) with increasing amounts of **1a** (34 mM stock solution).

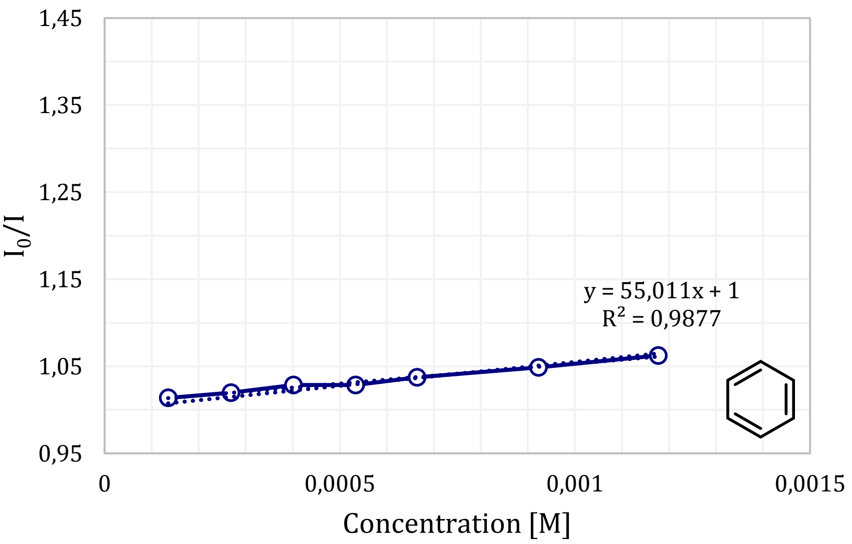


**Figure S32.** Stern-Volmer plot (Ex. 375) of compound **Luc/BF₄** (15 µL of 14 mg.L⁻¹ stock solution) in MeCN (2.5 mL) with increasing amounts of **1a** (34 mM stock solution) in the following steps 10 µL, 20 µL, 30 µL, 40 µL, 50 µL, 70 µL, 90 µL.

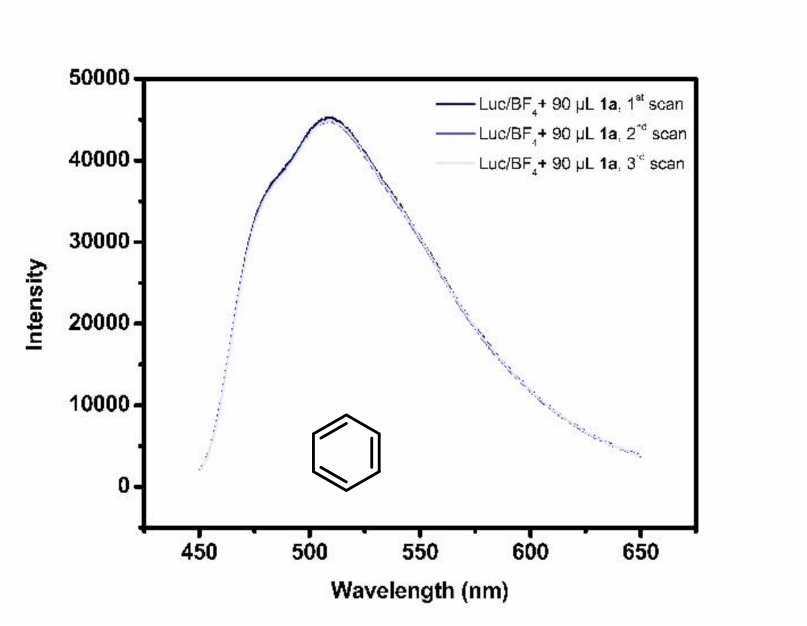


**Figure S33.** Fluorescence emission spectra (Ex. 375) of compound **Luc/BF$_4$** (15 µL of 14 mg.L$^{-1}$ stock solution) in MeCN (2.5 mL) with 90 µL of **1a** (34 mM stock solution) measured in triplicate for reproducibility.

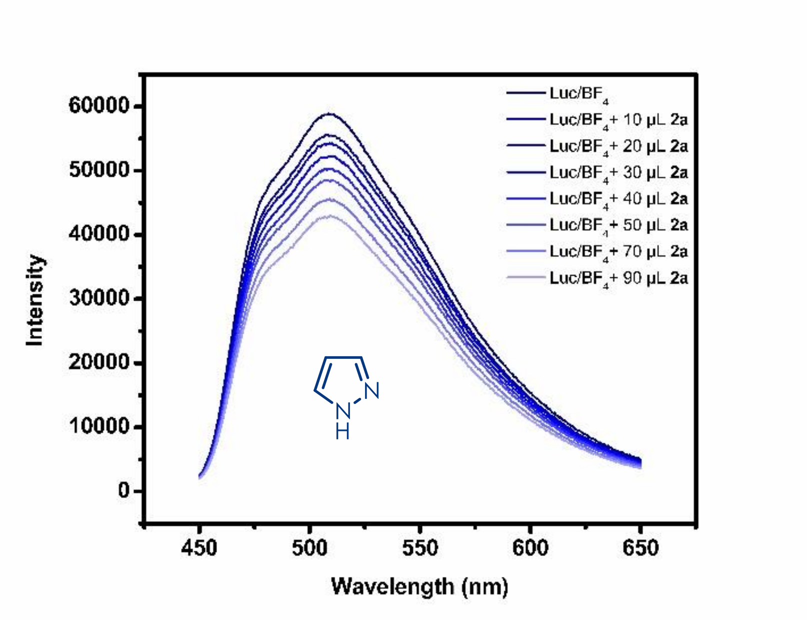


**Figure S34.** Fluorescence emission spectra (Ex. 375) of compound **Luc/BF$_4$** (15 µL of 14 mg.L$^{-1}$ stock solution) in MeCN (2.5 mL) with increasing amounts of **2a** (34 mM stock solution).

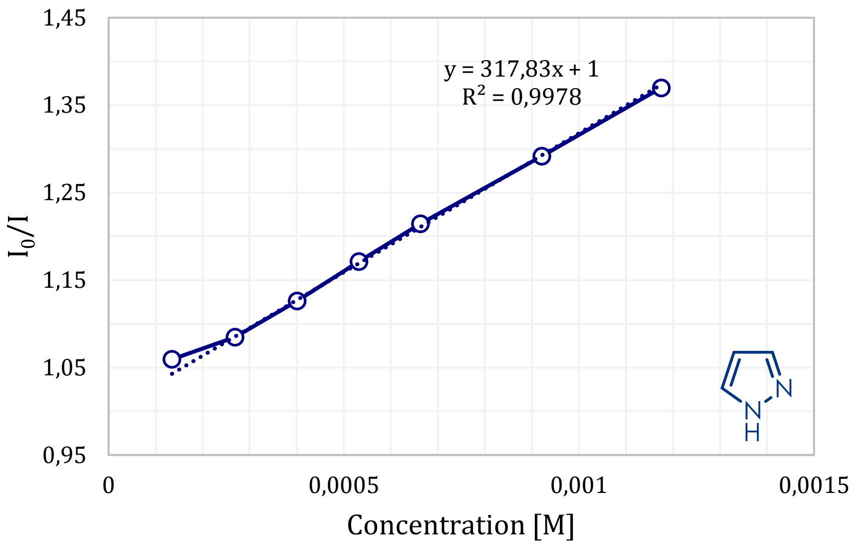


**Figure S35.** Stern-Volmer plot (Ex. 375) of compound **Luc/BF$_4$** (15 µL of 14 mg.L$^{-1}$ stock solution) in MeCN (2.5 mL) with increasing amounts of **2a** (34 mM stock solution) in the following steps 10 µL, 20 µL, 30 µL, 40 µL, 50 µL, 70 µL, 90 µL.

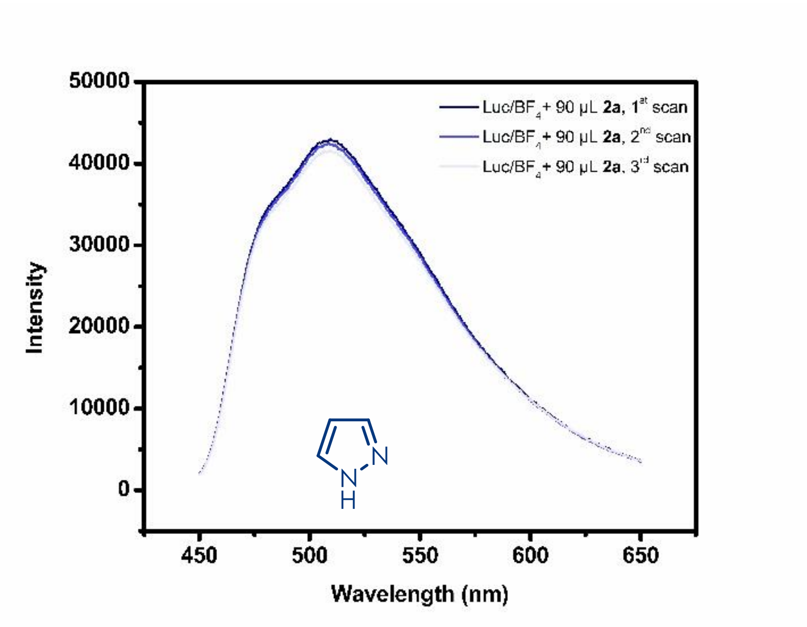


**Figure S36.** Fluorescence emission spectra (Ex. 375) of compound **Luc/BF$_4$** (15 µL of 14 mg.L$^{-1}$ stock solution) in MeCN (2.5 mL) with 90 µL of **2a** (34 mM stock solution) measured in triplicate for reproducibility.

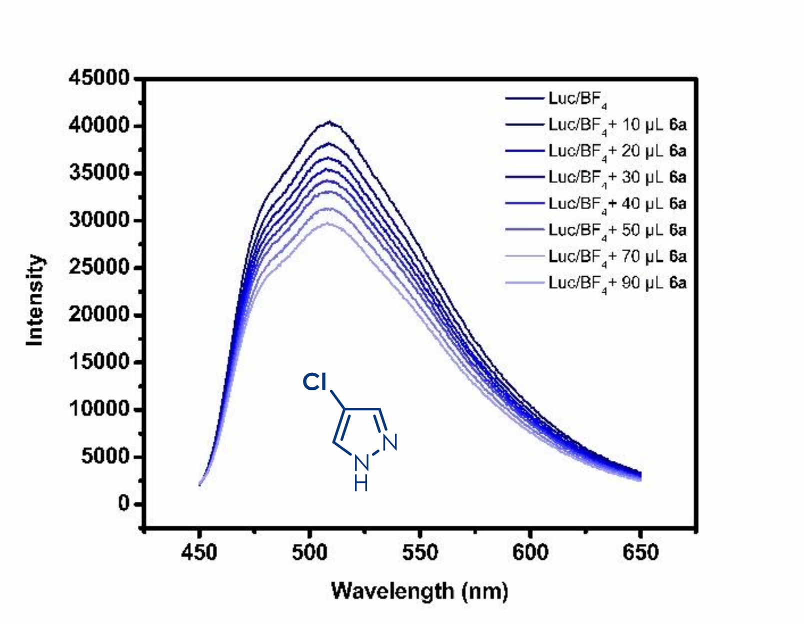


**Figure S37.** Fluorescence emission spectra (Ex. 375) of compound **Luc/BF₄** (15 µL of 14 mg.$L^{-1}$ stock solution) in MeCN (2.5 mL) with increasing amounts of **6a** (34 mM stock solution).

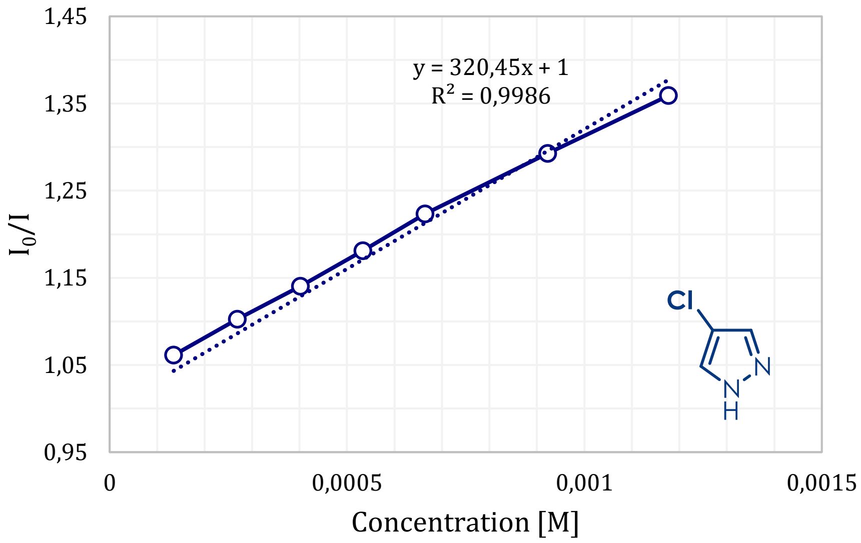


**Figure S38.** Stern-Volmer plot (Ex. 375) of compound **Luc/BF₄** (15 µL of 14 mg.$L^{-1}$ stock solution) in MeCN (2.5 mL) with increasing amounts of **6a** (34 mM stock solution) in the following steps 10 µL, 20 µL, 30 µL, 40 µL, 50 µL, 70 µL, 90 µL.

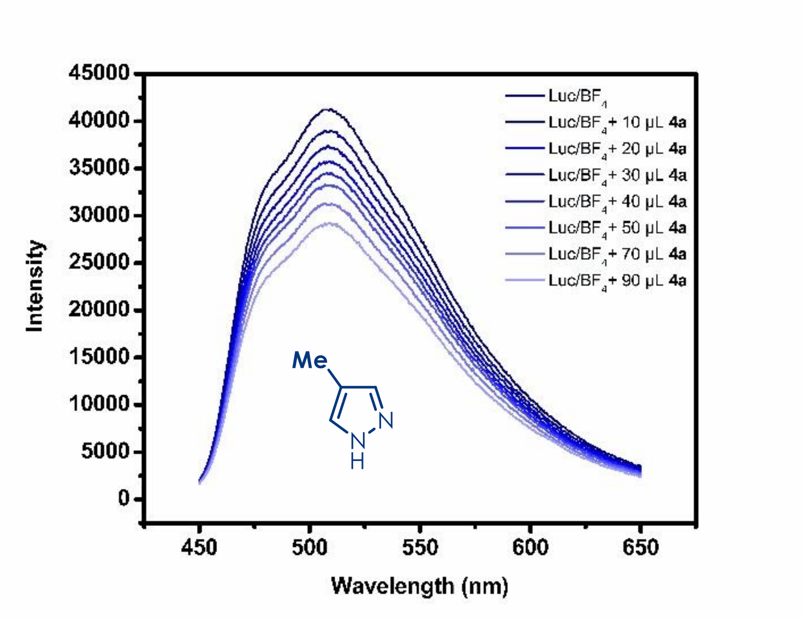


**Figure S39.** Fluorescence emission spectra (Ex. 375) of compound **Luc/BF$_4$** (15 µL of 14 mg.L$^{-1}$ stock solution) in MeCN (2.5 mL) with increasing amounts of **4a** (34 mM stock solution).

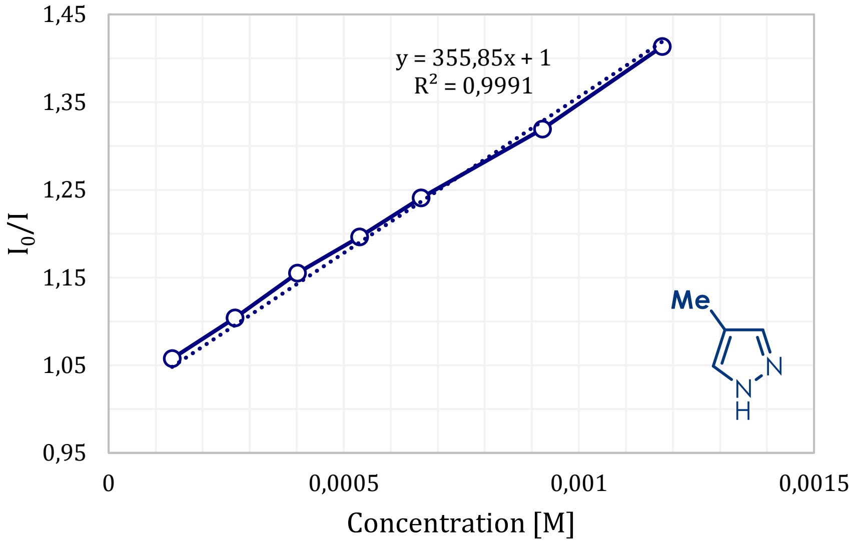


**Figure S40.** Stern-Volmer plot (Ex. 375) of compound **Luc/BF$_4$** (15 µL of 14 mg.L$^{-1}$ stock solution) in MeCN (2.5 mL) with increasing amounts of **4a** (34 mM stock solution) in the following steps 10 µL, 20 µL, 30 µL, 40 µL, 50 µL, 70 µL, 90 µL.

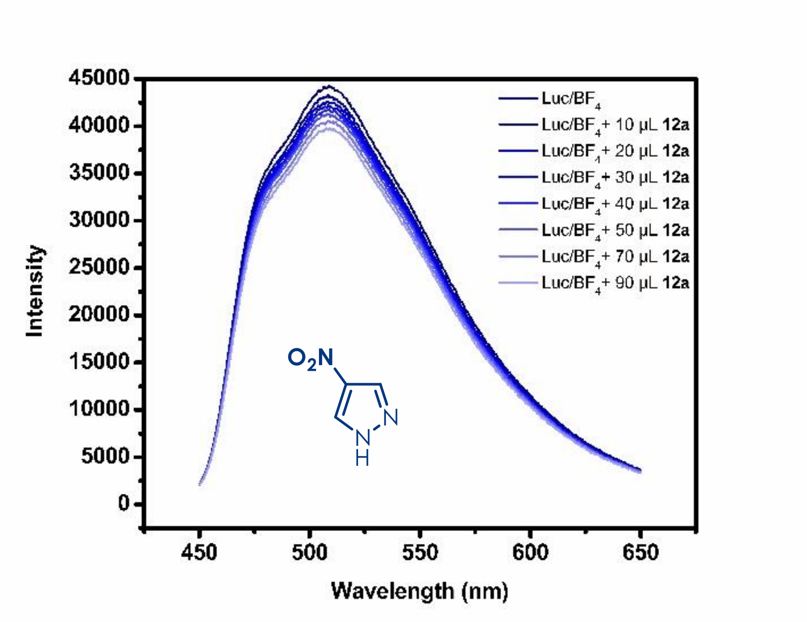


**Figure S41.** Fluorescence emission spectra (Ex. 375) of compound **Luc/BF$_4$** (15 µL of 14 mg.L$^{-1}$ stock solution) in MeCN (2.5 mL) with increasing amounts of **12a** (34 mM stock solution).

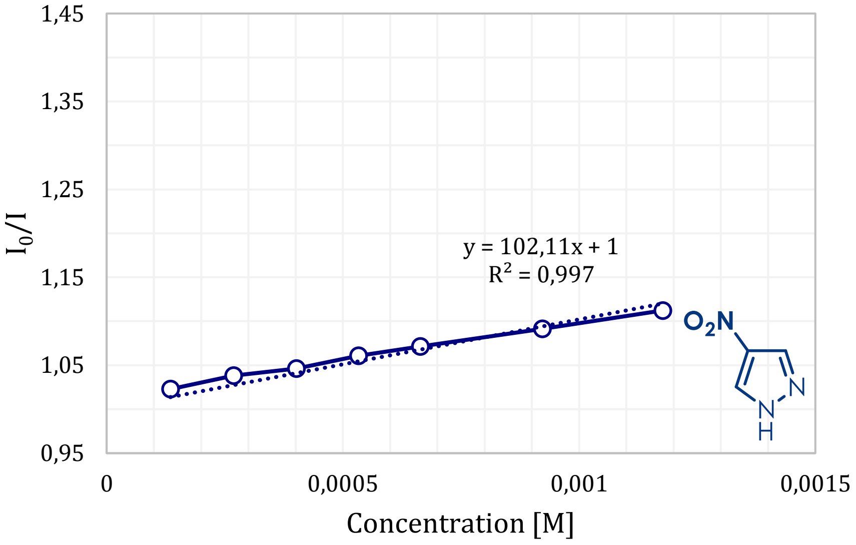


**Figure S42.** Stern-Volmer plot (Ex. 375) of compound **Luc/BF$_4$** (15 µL of 14 mg.L$^{-1}$ stock solution) in MeCN (2.5 mL) with increasing amounts of **12a** (34 mM stock solution) in the following steps 10 µL, 20 µL, 30 µL, 40 µL, 50 µL, 70 µL, 90 µL.

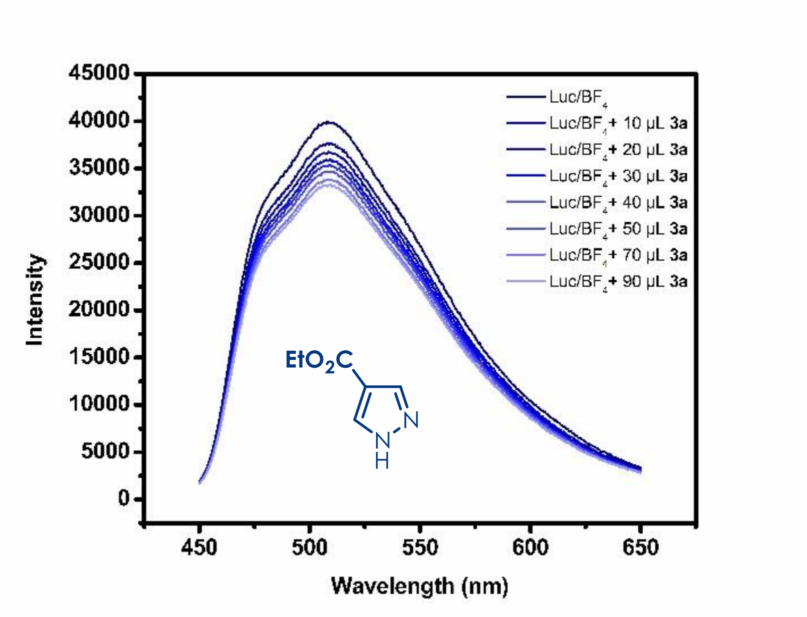


**Figure S43.** Fluorescence emission spectra (Ex. 375) of compound **Luc/BF$_4$** (15 µL of 14 mg.L$^{-1}$ stock solution) in MeCN (2.5 mL) with increasing amounts of **3a** (34 mM stock solution).

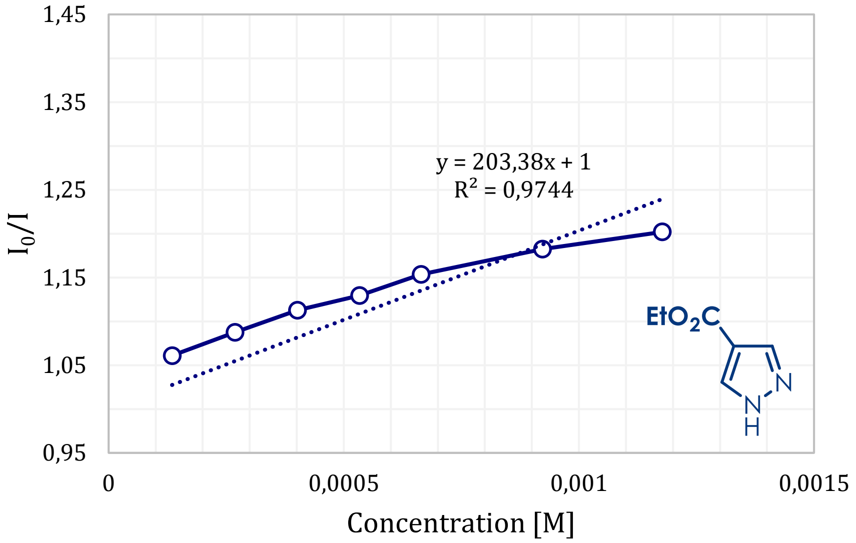


**Figure S44.** Stern-Volmer plot (Ex. 375) of compound **Luc/BF$_4$** (15 µL of 14 mg.L$^{-1}$ stock solution) in MeCN (2.5 mL) with increasing amounts of **3a** (34 mM stock solution) in the following steps 10 µL, 20 µL, 30 µL, 40 µL, 50 µL, 70 µL, 90 µL.

- **Additional Stern-Volmer quenching studies**

Considering the large excess of benzene under the reaction conditions, formation of the benzene radical cation cannot be excluded. To better approximate the reaction conditions, additional fluorescence quenching experiments were performed in the presence of

benzene. In these experiments, the quenching study with pyrazole **2a** was repeated with the following modification: 100 µL of a benzene **1a** stock solution (340 mM) was added first to a solution of **Luc/BF**$_4$ (15 µL of 14 mg.L$^{-1}$ stock solution) in MeCN (2.5 mL), before incremental addition of pyrazole **2a** as the quencher. The emission intensity of this initial benzene-containing solution was measured first and taken as $I_0$. Addition of benzene in this amount was intended to approximate the 110:1 benzene:pyrazole ratio used in the standard reaction mixture more closely. As shown in **Figure S45** and **Figure S46**, pyrazole **2a** still quenches the fluorescence more efficiently than benzene (with K=247), even when benzene is present in excess relative to pyrazole.

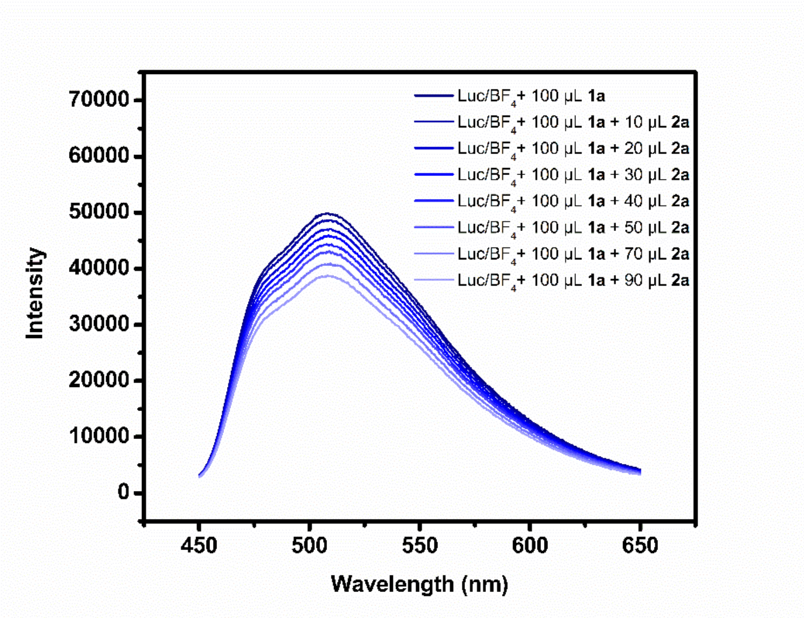


**Figure S45.** Fluorescence emission spectra (Ex. 375) of mixture between **Luc/BF**$_4$ (15 µL of 14 mg.L$^{-1}$ stock solution) and 100 µL of **1a** (340 mM stock solution) in MeCN (2.5 mL) with increasing amounts of **2a** (34 mM stock solution).

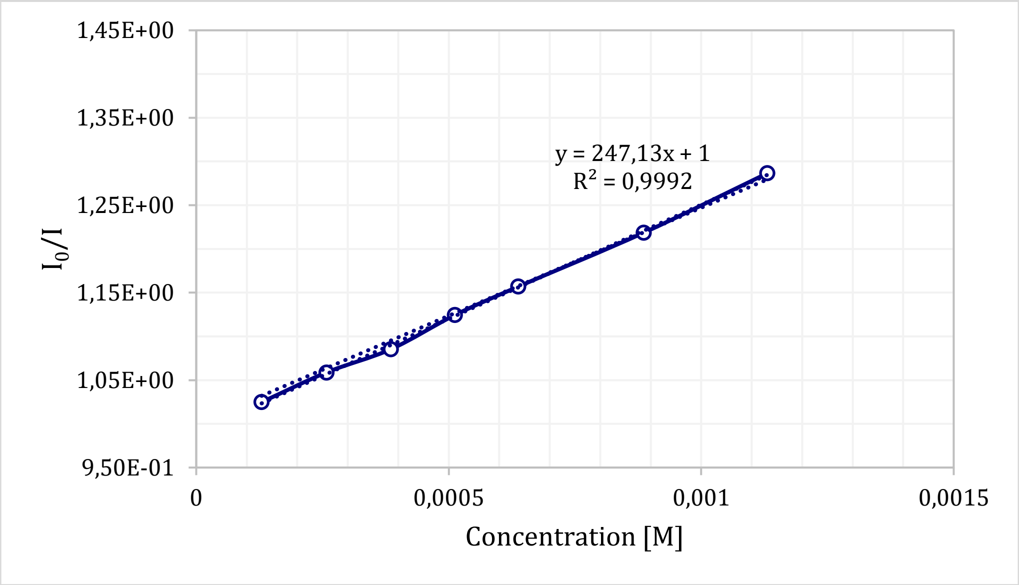


**Figure S46.** Stern-Volmer plot (Ex. 375) of mixture between **Luc/BF4** (15 µL of 14 mg.L$^{-1}$ stock solution) and 100 µL of **1a** (340 mM stock solution) in MeCN (2.5 mL) with increasing amounts of **2a** (34 mM stock solution) in the following steps 10 µL, 20 µL, 30 µL, 40 µL, 50 µL, 70 µL, 90 µL.

Considering the poor reactivity of some arene substrates, such as anisole **45a**, we also conducted fluorescence quenching studies to observe the rate of quenching in the presence of our photocatalyst. In this experiment, the quenching study with arene **45a** was conducted in the presence of **Luc/BF4** (15 µL of 14 mg.L$^{-1}$ stock solution) in MeCN (2.5 mL), with incremental addition of arene **45a** as the quencher. The quenching rate (K = 511) with anisole is much larger than that of benzene **1a** (K = 55) or some of the best performing pyrazoles, for example **3a** (K = 203 )or **12a** (K = 102), confirming our previous observations that quenching rate cannot be directly correlated with the reactivity efficiency of the different substrates.

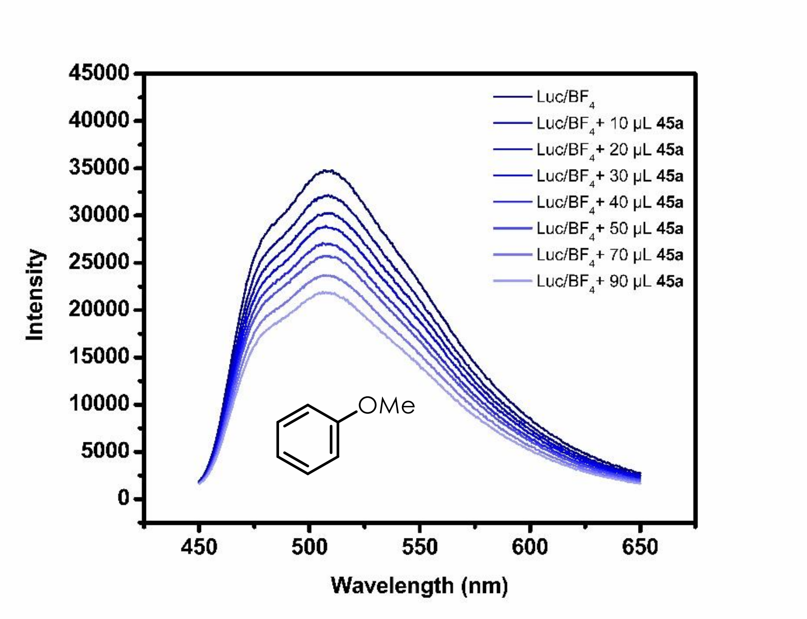


**Figure S47.** Fluorescence emission spectra (Ex. 375) of compound **Luc/BF4** (15 µL of 14 mg.L-1 stock solution) in MeCN (2.5 mL) with increasing amounts of **45a** (34 mM stock solution).

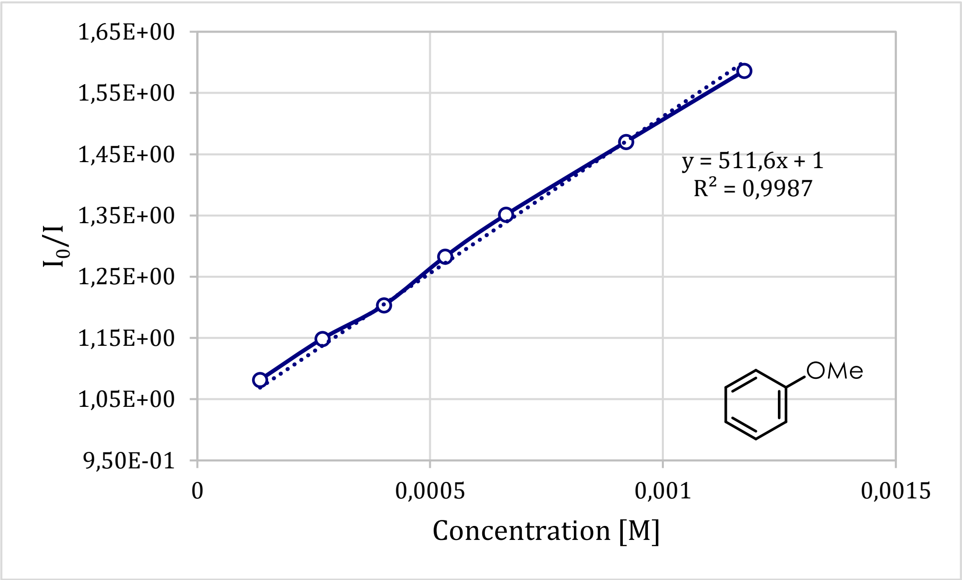


**Figure S48.** Stern-Volmer plot (Ex. 375) of compound **Luc/BF4** (15 µL of 14 mg.L-1 stock solution) in MeCN (2.5 mL) with increasing amounts of **45a** (34 mM stock solution) in the following steps 10 µL, 20 µL, 30 µL, 40 µL, 50 µL, 70 µL, 90 µL.

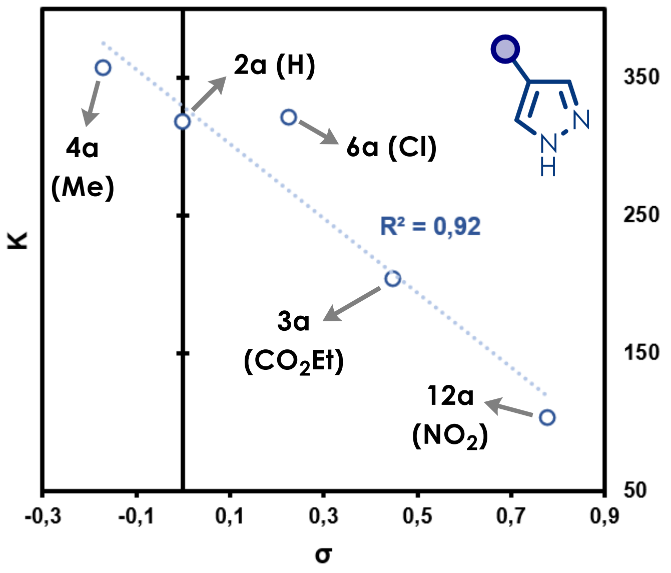


**Figure S49.** Plot of the Stern-Volmer quenching constants vs the corresponding Hammett substituent constants.

For **Figure S49**, the following Hammett substituent constants for *para*-substituents on benzene derivatives were used*:*

- $\sigma_p$ (R = H) = 0
- $\sigma_p$ (R = Me) = -0.17
- $\sigma_p$ (R = Cl) = 0.227
- $\sigma_p$ (R = $CO_2Et$) = 0.45
- $\sigma_p$ (R = $NO_2$) = 0.778

# 13 Mechanistic Study

## 13.1 Light on & off experiment

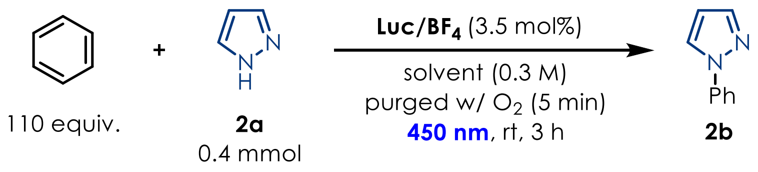


A dry 8 mL screw neck vial N15 equipped with a stirring bar was charged with pyrazole (27.2 mg, 0.4 mmol, 1.0 equiv.) and **Luc/BF4** (3.5 mol%, 14 µmol, 7.8 mg). Under air, dry MeCN (1.3 mL, *c* = 0.3 M) and dry benzene (4 mL, 44 mmol, 110 equiv.) were added. The vial was capped with a screw cap fitted with a Silicone/PTFE septum. The resulting

solution was sparged with oxygen for 5 min (either with a 1 atm balloon or directly from an oxygen tank fitted with a pressure regulator). The vial was placed in the Penn PhD Photoreactor M2 equipped with a 450 nm light source. The parameters used for the irradiation are described in **part 1.2**.

The progress of the reaction was monitored by drying 0.3 mL of the reaction mixture (while keeping the vial under an oxygen atmosphere) and a stock solution of 1,3-dinitrobenzene (using 0.5 mL of 0.4 M solution in MeCN) was used as an internal standard to determine the yield of oxidative coupling product in DMSO-$d_6$.

During the designated "dark" time period, the reaction vial was wrapped with aluminum foil and kept in the Penn photoreactor with continuous stirring. After the dark period, the foil was removed, and the reaction was allowed to proceed with irradiation.

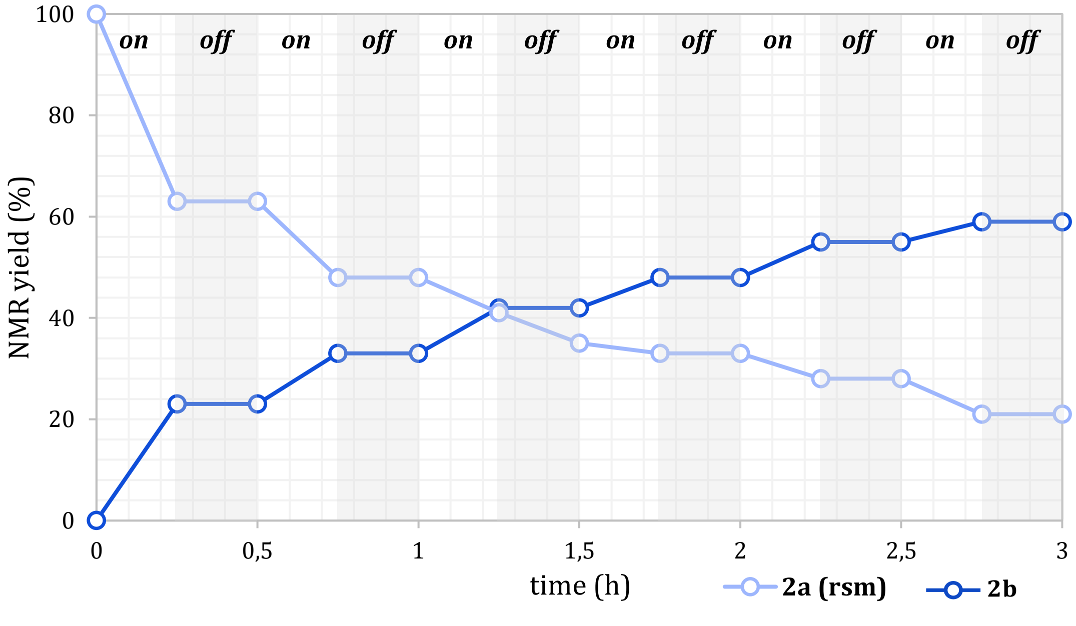


**Figure S50.** Light On/Off experiments for the model reaction.

### 13.2 Reaction profile

All entries in **Table S11** and **Table S12** were run using a similar procedure to **GP1a**. To ensure the best reproducibility possible given the reactions were run in parallel, a stock solution of pyrazole and **Luc/BF$_4$** in acetonitrile was prepared ($c$ = 0.3 M so 0.67 mL is used for one reaction). For the analysis, a stock solution of 1,3-dinitrobenzene (using 0.5

mL of 0.4 M solution in MeCN or EtOAc) was added, The resulting mixture was stirred vigorously, the solvent was evaporated, and the residue was diluted with DMSO-$d_6$ (0.5 mL) and analysed by $^1$H NMR spectroscopy to determine the NMR yield.

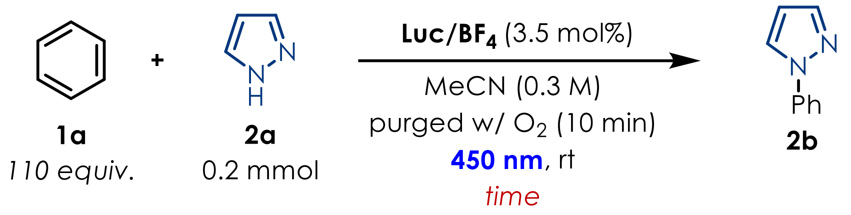


**Table S11.** Reaction profile results for the model reaction when benzene is used.

| entry | time (s) | time (h) | 2a (%) | 2b (%) |
|---|---|---|---|---|
| 1 | 60 | 0,0167 | 69 | 2 |
| 2 | 180 | 0,05 | 52 | 5 |
| 3 | 300 | 0,083 | 52 | 8 |
| 4 | 390 | 0,1083 | 56 | 11 |
| 5 | 480 | 0,1333 | 60 | 13 |
| 6 | 600 | 0,1667 | 63 | 15 |
| 7 | 1200 | 0,3333 | 50 | 21 |
| 8 | 2400 | 0,6667 | 32 | 45 |
| 9 | 3600 | 1 | 27 | 49 |
| 10 | 7200 | 2 | 9 | 68 |
| 11 | 14400 | 4 | 12 | 66 |

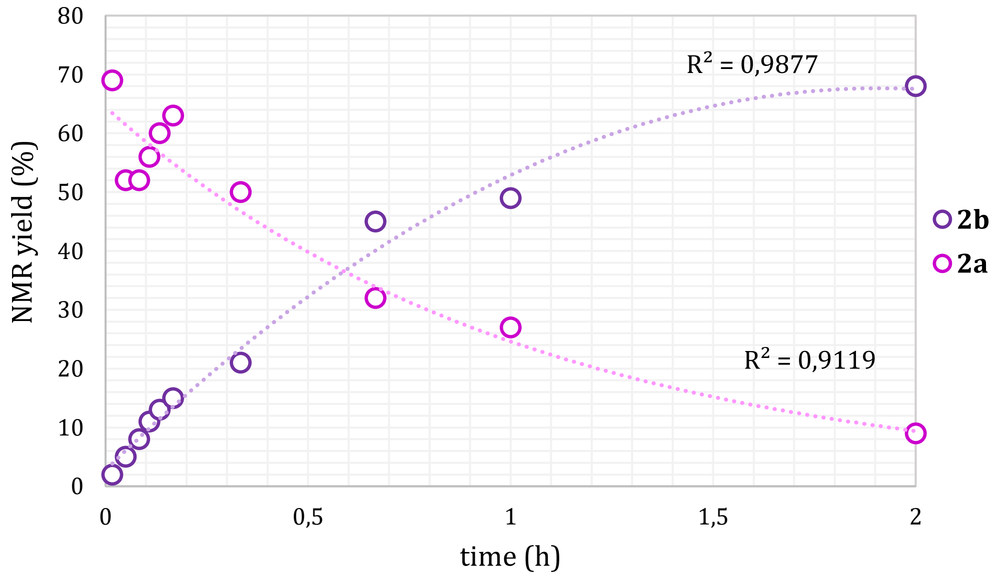


**Figure S51.** Reaction profile for the model reaction. For **2a**, an exponential fitting function was used ($y = a \cdot e^{bx},\ \forall(a, b) \in \boldsymbol{R}^2$). For **2b**, a second order polynomial fitting function was used ($y = ax^2 + bx + c,\ (a, b, c) \in \boldsymbol{R}^3$).

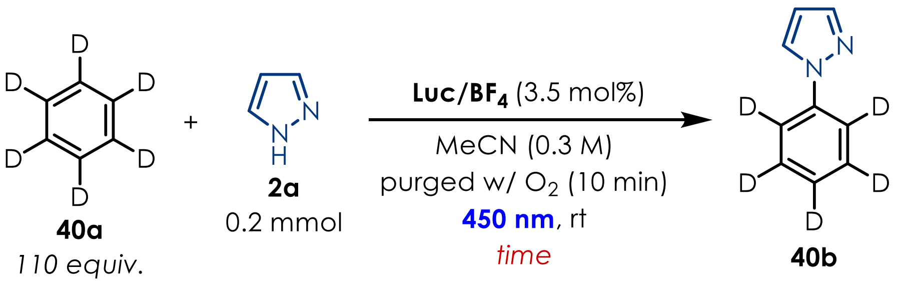


**Table S12.** Reaction profile results for the model reaction when benzene-$d_6$ is used.

| entry | time (s) | time (h) | 40a (%) | 40b (%) |
|---|---|---|---|---|
| 1 | 60 | 0,0167 | 71 | 1 |
| 2 | 180 | 0,05 | 78 | 4 |
| 3 | 300 | 0,083 | 71 | 7 |
| 4 | 390 | 0,1083 | 64 | 11 |
| 5 | 480 | 0,1333 | 54 | 10 |
| 6 | 600 | 0,1667 | 62 | 12 |
| 7 | 1200 | 0,3333 | 54 | 26 |
| 8 | 2400 | 0,6667 | 39 | 34 |
| 9 | 3600 | 1 | 17 | 41 |
| 10 | 7200 | 2 | 13 | 58 |
| 11 | 14400 | 4 | 12 | 56 |

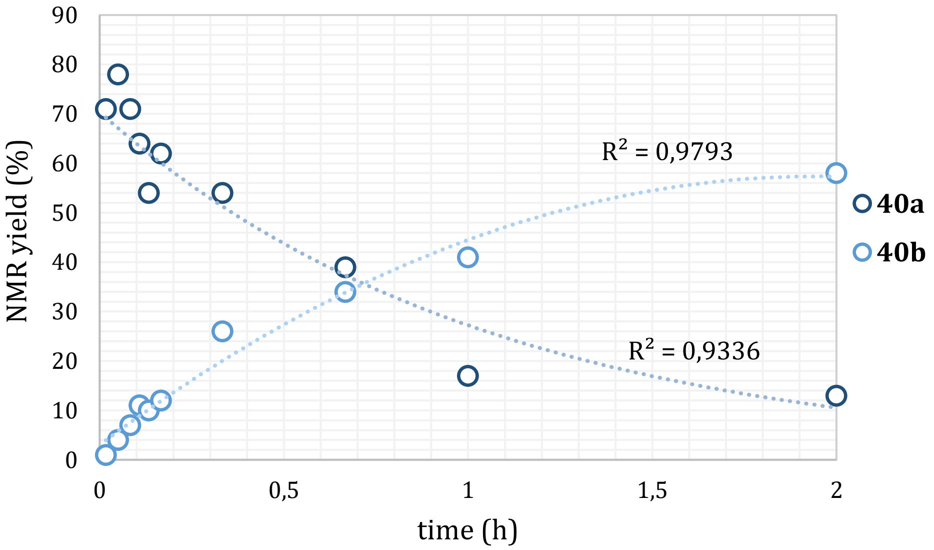


**Figure S52** Reaction profile for the model reaction with benzene-$d_6$. For **40a**, an exponential fitting function was used ($y = a \cdot e^{bx},\ \forall(a,b) \in \boldsymbol{R^2}$). For **40b**, a second order polynomial fitting function was used ($y = ax^2 + bx + c,\ (a,b,c) \in \boldsymbol{R^3}$).

### 13.3 Kinetic Isotope Effect experiments (KIE)

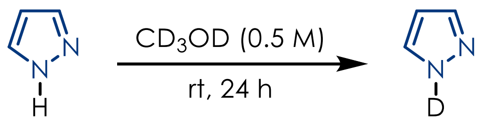


The procedure was adapted from a reported protocol.[40] A 25 mL round-bottom flask equipped with a magnetic stir bar was charged with **2a** (400 mg, 5.87 mmol). The solid was then dissolved in *d*$_4$-MeOH (12 mL) to obtain a 0.5 M solution. The reaction mixture was then stirred at 23 ºC for 48 hours. The solution was then concentrated in vacuo to afford the title compound **2a-*d*$_1$** as a white solid quantitatively (>99%).

$^1$H NMR shows >95% D incorporation.

**$^1$H NMR (600 MHz, CDCl3)** δ 7.63 (d, J = 2.10 Hz, 2H), 6.36 (t, J = 2.12 Hz, 1H).

**$^2$H NMR (92 MHz, CHCl3)** δ 5.84 (br s, 1H).

Data in accordance with the literature.[40]

- **KIE via parallel reactions**

**Figure S53** compare the yields and products obtained after 2 h for the model reaction when respectively benzene, benzene-*d*$_6$ or pyrazole-*d*$_1$ is used.

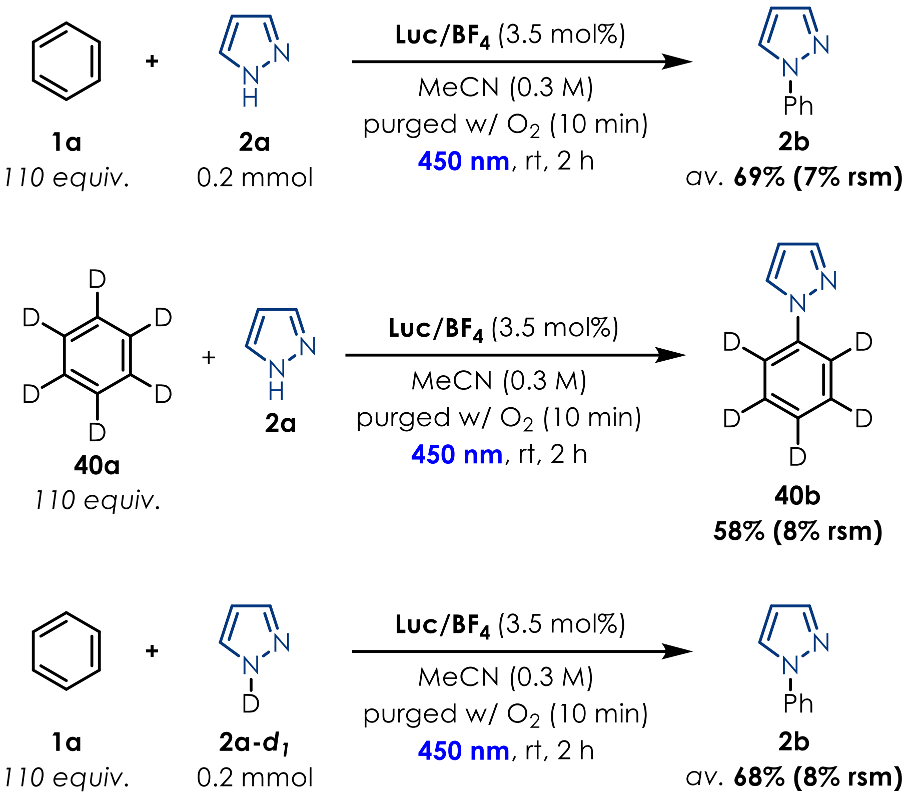


**Figure S53.** Overview of the results obtained for the model reaction when respectively benzene, benzene-$d_6$ or pyrazole-$d_1$ is used.

All entries in **Table S13** were run using a similar procedure to **GP1a**. To ensure the best reproducibility possible given the reactions were run in parallel, a stock solution of pyrazole and **Luc/BF$_4$** in acetonitrile was prepared ($c$ = 0.3 M so 0.67 mL is used for one reaction). For the analysis, a stock solution of 1,3-dinitrobenzene (using 0.5 mL of 0.4 M solution in MeCN or EtOAc) was added. The resulting mixture was stirred vigorously, the solvent was evaporated, and the residue was diluted with DMSO-$d_6$ (0.5 mL) and analysed by $^1$H NMR spectroscopy to determine the NMR yield.

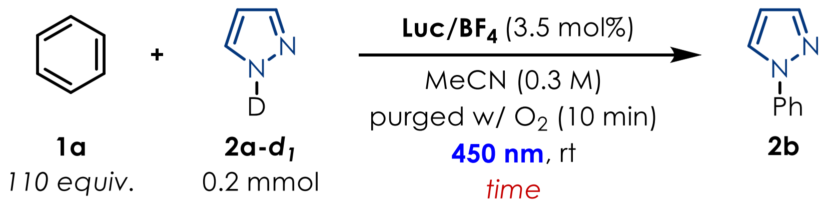


**Table S13.** Reaction profile results for the model reaction when pyrazole-$d_1$ is used.

| entry | time (s) | time (h) | 2a-$d_1$ (%) | 2b (%) |
|---|---|---|---|---|
| 1 | 120 | 0,0333 | 58 | 8 |
| 2 | 240 | 0,0667 | 59 | 18 |
| 3 | 360 | 0,1 | 54 | 21 |
| 4 | 480 | 0,1333 | 54 | 27 |
| 5 | 600 | 0,1667 | 51 | 29 |

The KIE for each parallel reaction was determined by estimating the kinetic constant via the initial rate method. At low conversions (*i.e.* less than 10-20% conversion), the rates of the reaction can be approximated as a linear equation: $[P] = k_0 \cdot t$ , where $k_0$ corresponds to the initial rate.[41–43]

The data used were described in part **13.2** for the reaction profiles of the model reaction involving respectively benzene and benzene-$d_6$ (see **Table S11** and **Table S12**). Finally, we also used the data for the reaction profile of the model reaction involving pyrazole-$d_1$ described in **Table S13**.

**Figure S54** and **Figure S55** represent the plot of NMR yield vs time and the corresponding linear fit, R-squarred value and linear equation from which we can extract the initial rate —it will be assumed to be respectively $k_H$ and $k_D$ here—that we will use to calculate the KIE.

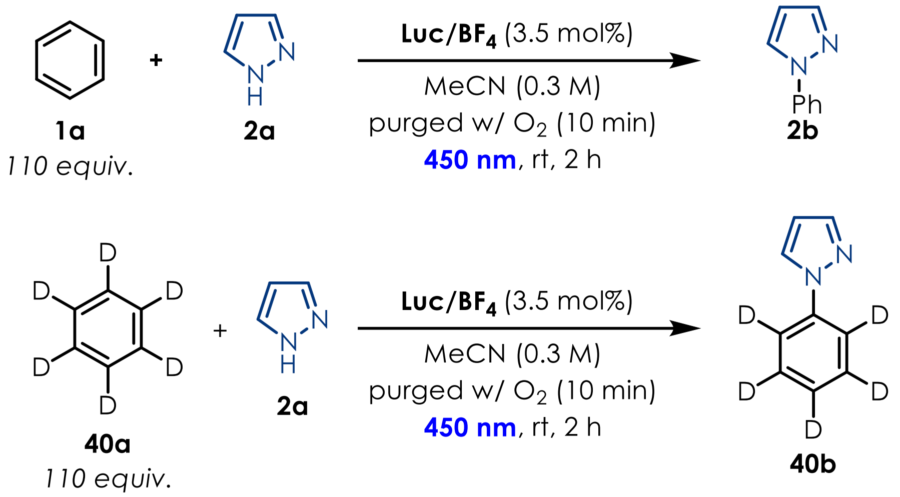


$k_H/k_D$ = 1.20

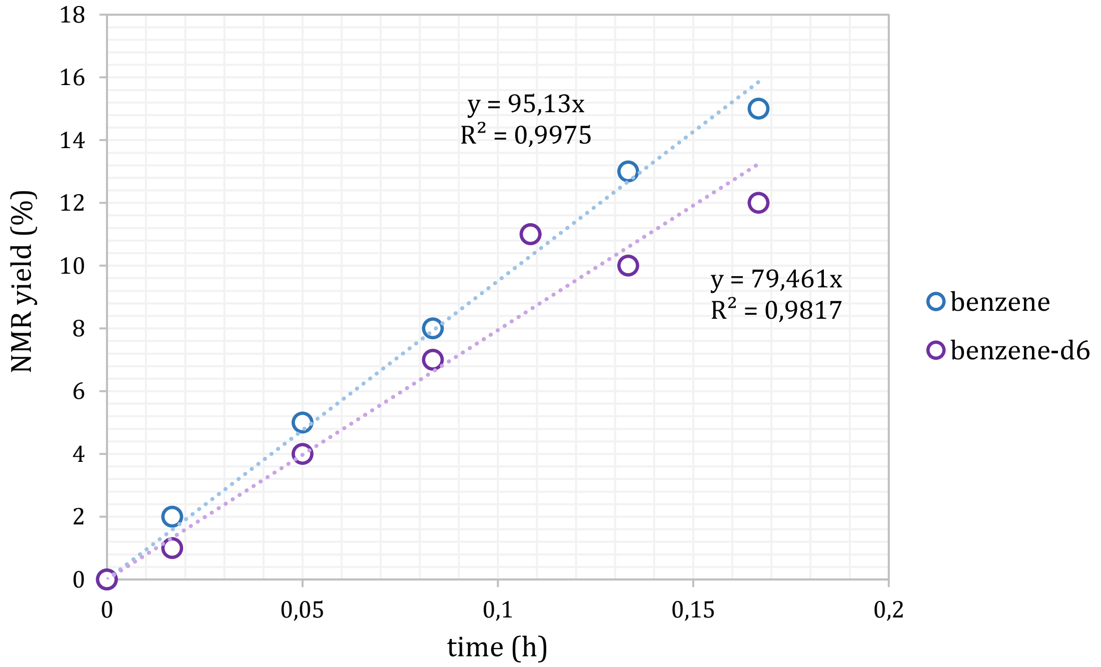


**Figure S54.** Comparison of the reaction profile at low conversion for the model reaction using respectively benzene and benzene-$d_6$.

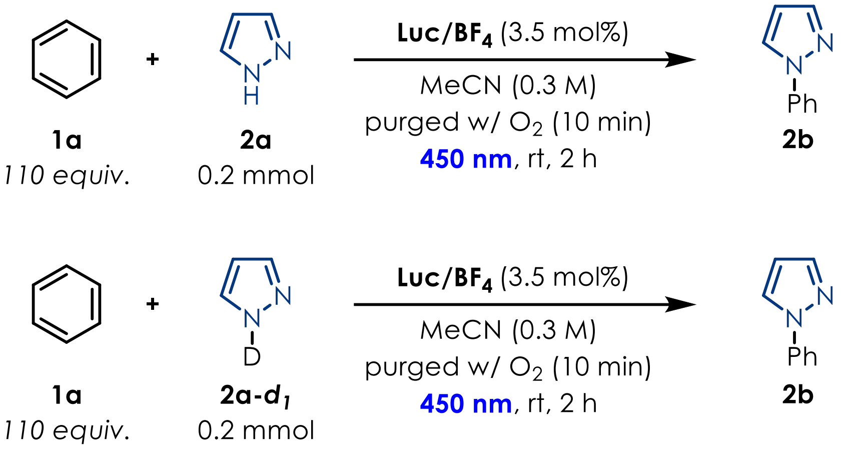


$k_H/k_D = 0.44$

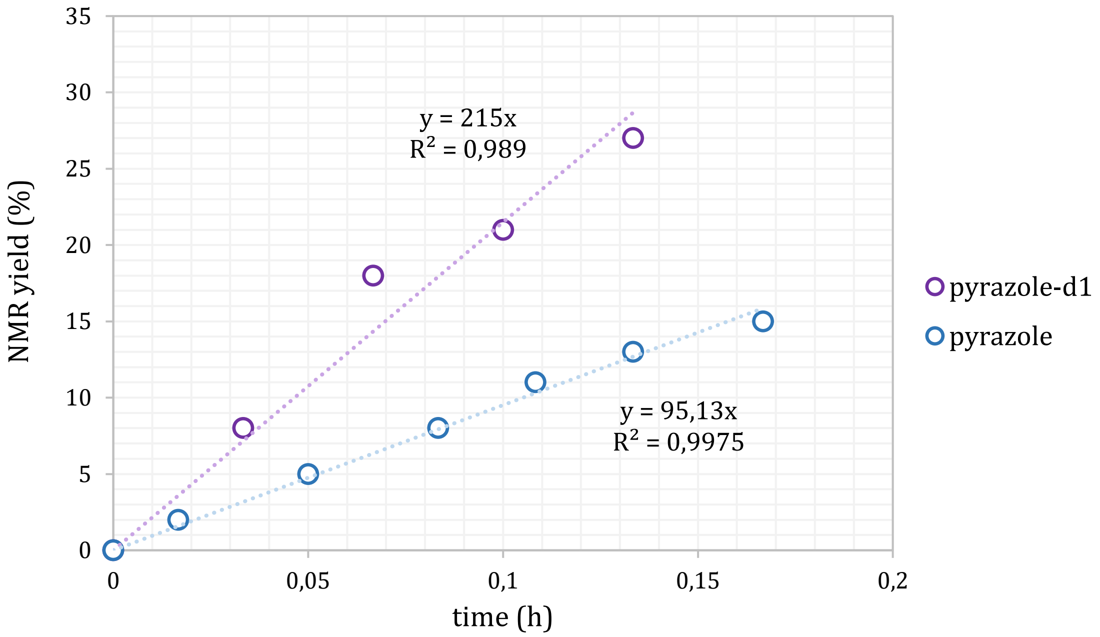


**Figure 55.** Comparison of the reaction profile at low conversion for the model reaction using respectively pyrazole and pyrazole-$d_1$.

**KIE via intermolecular competition**

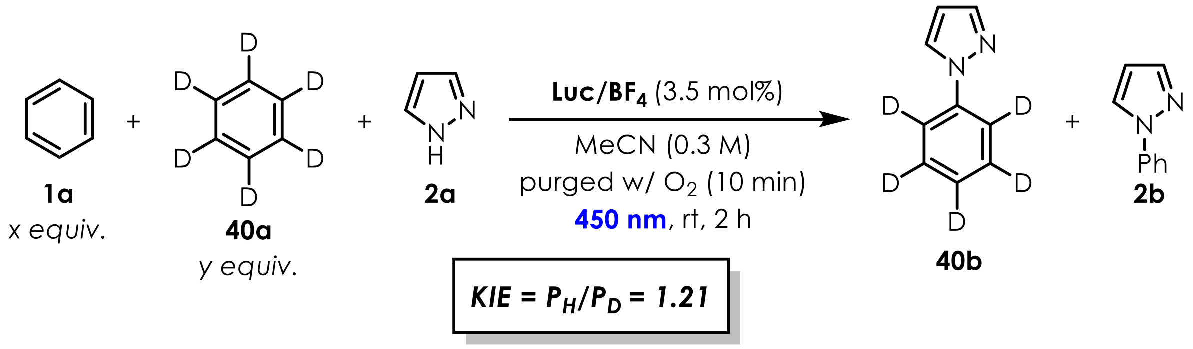


A dry 8 mL screw neck vial N15 equipped with a stirring bar was charged with pyrazole (13.6 mg, 0.2 mmol, 1.0 equiv.) and **Luc/BF$_4$** (3.5 mol%, 7 µmol, 3.9 mg). Under air, dry MeCN (0.65 mL, *c* = 0.3 M) a mixture of benzene and benzene-$d_6$ (see the **Table S14** for the ratio, 110 equiv. of arene in total) were added. The vial was capped with a screw cap fitted with a Silicone/PTFE septum. The resulting solution was sparged with oxygen for 5 min (either with a 1 atm balloon or directly from an oxygen tank fitted with a pressure regulator). The vial was placed in the Penn PhD Photoreactor M2 equipped with a 450 nm light source. The parameters used for the irradiation are described in **part 1.2**. The mixture was stirred under irradiation for 2 h. Upon completion, a stock solution of 1,3-dinitrobenzene (using 0.5 mL of 0.4 M solution in MeCN or EtOAc) was added. The resulting mixture was stirred vigorously, the solvent was evaporated, and the residue was diluted with DMSO-$d_6$ (0.5 mL) and analysed by $^1$H NMR spectroscopy to determine the NMR yield.

**Table S14.** KIE study via intermolecular competition and the observed KIE depending on the ratio of benzene and benzene-$d_6$ used.

| entry | x:y | 2a (%) | 2b (%) | 40b (%) | $P_H/P_D$ |
|---|---|---|---|---|---|
| 1 | 2:1 | 15 | 36 | 16 | 2.25 |
| 2 | 1:1 | 15 | 29 | 24 | 1.21 |
| 3 | 1:2 | 14 | 18 | 34 | 0.53 |

When a 1:1 ratio of benzene and benzene-$d_6$ is used, the KIE can be directly calculated by dividing the NMR yield obtained for the deuterated product **40b** and the non-deuterated product **2b**. The calculated KIE here was: $\boxed{KIE = \frac{P_H}{P_D} = 1.21}$.

### 13.4 Control experiments

We finally carried out several control experiments in order to understand the role of each substrate in the mechanism as well as possible side reactivity and decomposition. All reactions were carried out in a similar way to what is described in **GP1a.**

The product of the model reaction 1-phenyl-1*H*-pyrazole (**2b**) did not decompose in the absence of photocatalyst at 450 nm, with and without oxygen purge before irradiation (see **Figure S56**).

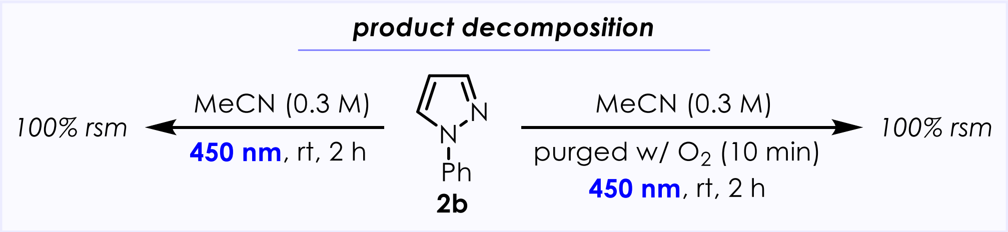


**Figure S56.** Product decomposition under irradiation at 450 nm, with and without oxygen purge.

The photocatalyst used for the scope of the process did not significantly decompose under irradiation: 90% was recovered after irradiation at 450 nm, with and without oxygen purge before irradiation. We observed the same kind of behaviour in the model reaction, *i.e.* partial recovery of the photocatalyst is observed (50-80% in general) (see **Figure S57**).

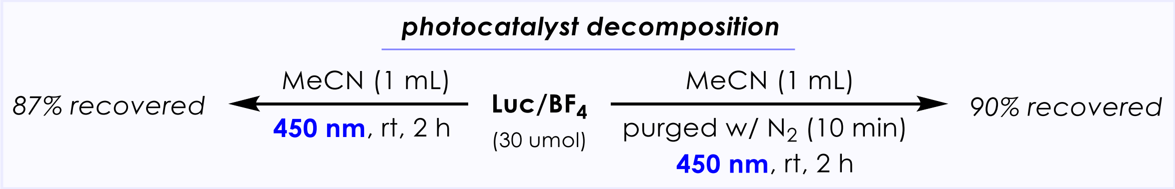


**Figure S57.** Photocatalyst decomposition under irradiation at 450 nm, with and without oxygen purge.

Electron-rich arenes are poorly reactive under our optimal set of conditions. As shown in the manuscript, anisole formed the corresponding product in only 16% yield after 2 h

with 22% conversion by NMR (see **Figure S58**)., while phenol did not react at all into desired product (83% rsm after 2 h).

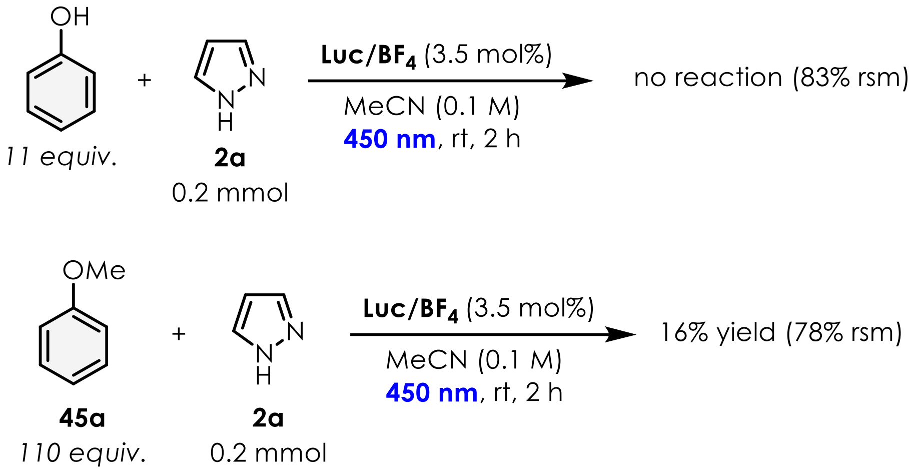


**Figure S58.** Reactivity of electron-rich arenes.

We then evaluated the decomposition of each reagent/product one by one and in combination under the standard reaction conditions described in **GP1a**. Benzene did not show any sign of decomposition or the formation of other products by NMR (see **Figure S59**).

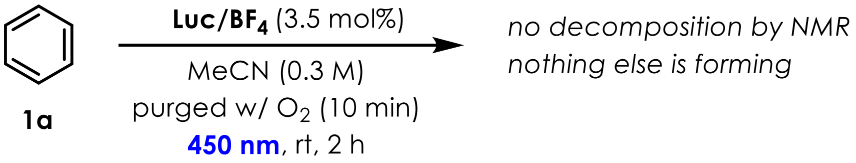


**Figure S59.** Decomposition of benzene under the standard conditions.

Notably, when a pyrazole solution was irradiated without benzene, we observed a significant amount of decomposition. The NMR became quite messy after the reaction completion but a single major product could not be easily identified. By GC-MS analysis, we managed to identify the formation of a possible side product. One of the main ion peaks had a mass of 134.1 with the following mass for the fragments: 106, 80, 67 and 53. This pattern could match with the structure of a bipyrazole derivative (see the grey box in **Figure S60**). At the moment, the exact structure is this intermediate is not known; however, the fragments obtained from 1'*H*-1,3'-bipyrazole (via the loss of "HCN" or "CN"

molecules as shown in **Figure S60**) seem to suggest that it could be a plausible structure as it would match with the mass of the fragments observed.

The formation of this side product seems to support the proposed mechanism involving the formation of the pyrazole radical cation that could dimerize into 1'*H*-1,3'-bipyrazole (probably via radical addition of the pyrazole radical cation onto another pyrazole and subsequent oxidation with **Luc/BF$_4$** to deliver the product) but further study are required to fully support the formation of an azole radical cation.

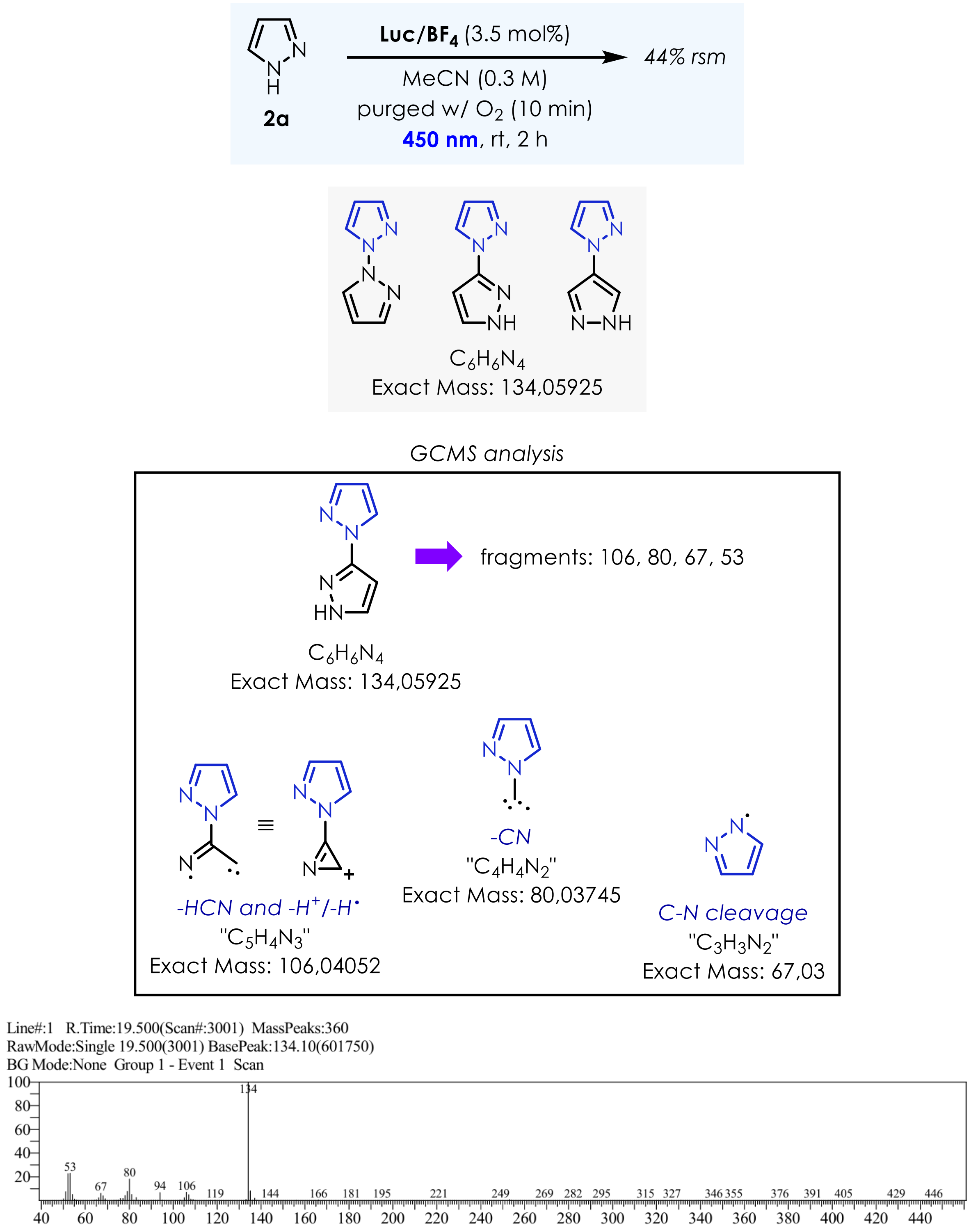


**Figure S60.** Decomposition of pyrazole under the standard conditions and plausible structures for the intermediate based on mass spectra analysis.

1-Phenyl-1*H*-pyrazole (**2b**) did not show any sign of decomposition under the standard conditions (see **Figure S61**).

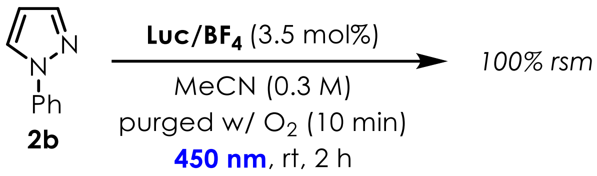


**Figure S61.** Decomposition of 1-phenyl-1*H*-pyrazole (**2b**) under the standard conditions.

A combination of pyrazole (**2a**) and 1-phenyl-1*H*-pyrazole (**2b**) showed a small amount of decomposition in the case of pyrazole (**2a**) and no decomposition in the case of 1-phenyl-1*H*-pyrazole (**2b**). No other product was observed by NMR (see **Figure S62**).

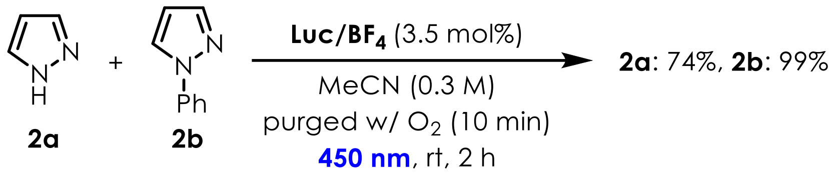


**Figure S62.** Decomposition of pyrazole (**2a**) in combination with 1-phenyl-1*H*-pyrazole (**2b**) under the standard conditions.

A combination of benzene (**1a**) and 1-phenyl-1*H*-pyrazole (**2b**) showed no sign of decomposition and no other product was observed by NMR (see **Figure S63**).

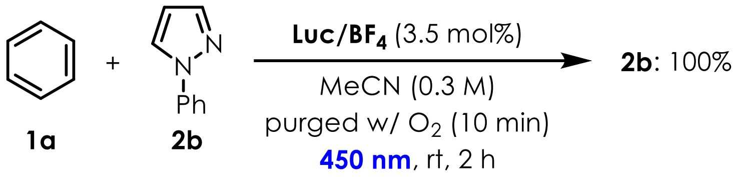


**Figure S63.** Decomposition of benzene (**1a**) in combination with 1-phenyl-1H-pyrazole (**2b**) under the standard conditions.

When the reaction was carried out with various amount of TEMPO, almost complete starting material recovery was observed and only low amount or traces of product were present by NMR depending on the amount of TEMPO used (see **Table S15**).

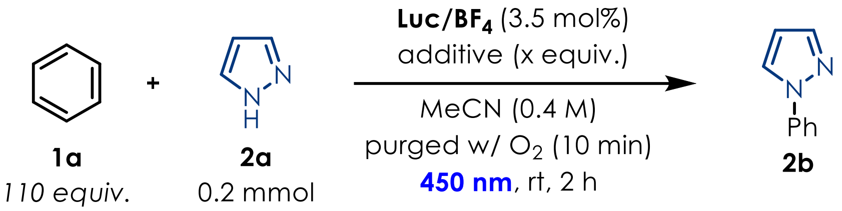


**Table S15.** Influence of the addition of TEMPO for the model reaction.

| entry | additive (equiv.) | sm (%) | 2b (%) |
|---|---|---|---|
| 1 | TEMPO (0.75 equiv.) | 70 | 12 |
| 2 | TEMPO (1.0 equiv.) | 67 | 9 |
| 3 | TEMPO (1.5 equiv.) | 77 | 4 |
| 4 | TEMPO (2.0 equiv.) | 81 | 3 |

### 13.5 $H_2O_2$ Detection Experiment

A dry 8 mL screw neck vial N15 equipped with a stirring bar was charged with 1*H*-pyrazole (0.2 mmol, 1.0 equiv., 13.6 mg) and **Luc/BF4** (3.5 mol%, 7 µmol, 3.9 mg). Under air, then dry acetonitrile (0.65 mL, *c* = 0.3 M) and dry benzene (2 mL, 22 mmol, 110 equiv.) were added. The vial was capped with a screw cap fitted with a Silicone/PTFE septum. The resulting solution was sparged with oxygen for 5 min (directly from an oxygen tank fitted with a pressure regulator). The vial was placed in the Penn PhD Photoreactor M2 equipped with a 450 nm light source. The parameters used for the irradiation are described in **part 1.2**. The mixture was stirred under irradiation for 2 h. Upon completion, the reaction mixture was poured into a 100 mL separatory funnel and dichloromethane (20 mL) was added. The organic phase was then extracted with distilled water (3x20 mL) and the aqueous layers were combined; the pH of the aqueous solution was estimated around 6.

Two types of peroxide strips were used to determine the presence of $H_2O_2$ in the aqueous layer. Peroxide test strips contain an organic redox indicator which, upon contact with peroxides, change colour. The peroxide concentration is determined semiquantitatively by visual comparison of the reaction zone of the test strip with the fields of a colour scale. For accurate measuring the pH of the samples should be within the range 2-12 (for the peroxide test strips measuring concentrations of up to 100 mg/L) and within the range 2-

7 (for the peroxide test strips measuring concentrations of up to 1000 mg/L). Measurements were performed by immersing the test strips for 1 sec in the aqueous solution. Excess liquid was allowed to run off and after approximately 5 sec (for the peroxide test strips measuring concentrations of up to 100 mg/L) and 30 sec (for the peroxide test strips measuring concentrations of up to 1000 mg/L) a comparison was made using the respective colour scales. Peroxide test strips are depicted in **Figure S64** indicating $H_2O_2$ with concentrations between 100 and 200 mg/L for the performed reaction, confirming that $H_2O_2$ is produced in our reaction.

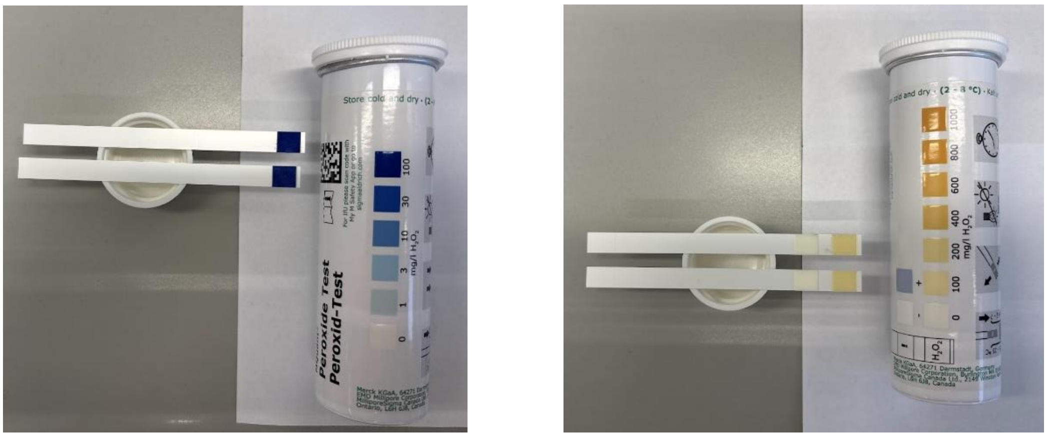


**Figure 64.** Semiquantitative analysis by two peroxide test strips measuring concentrations of up to 100 mg/L $H_2O_2$ (left) and up to 1000 mg/L $H_2O_2$ (right).

### 13.6 Mechanistic proposal

**Figure S65** gives an overview of a plausible mechanism based on the mechanistic studies reported in the previous part and extensively described in the main text and the Supporting Information. First, photoexcitation of Lucigenin would create *in situ* a strong photooxidant ($E^{*}_{red} \geq 2.23\ V\ vs\ SCE$) that could in theory oxidize either **1a** or **2a**. Fluorescence quenching studies and most of the pyrazole studied in the substrate scope suggest that pyrazole (**2a**) (or the azole) could be getting oxidized faster and therefore, radical cation intermediate **A** is generated via a one-electron oxidation. This highly electrophilic radical can then add onto benzene to form the radical cation intermediate **B** that can transform into the radical intermediate **C** after deprotonation. The delocalised radical on the phenyl ring can then be trapped by oxygen and then get oxidized to the expected product **2b** after elimination of hydroperoxyl radical. Alternatively, the

superoxide anion ($O_2^{\bullet-}$) forming during the photocatalyst regeneration with oxygen can also directly abstract one hydrogen from the radical intermediate **C** to deliver the expected product along with hydrogen peroxide.[3,41]

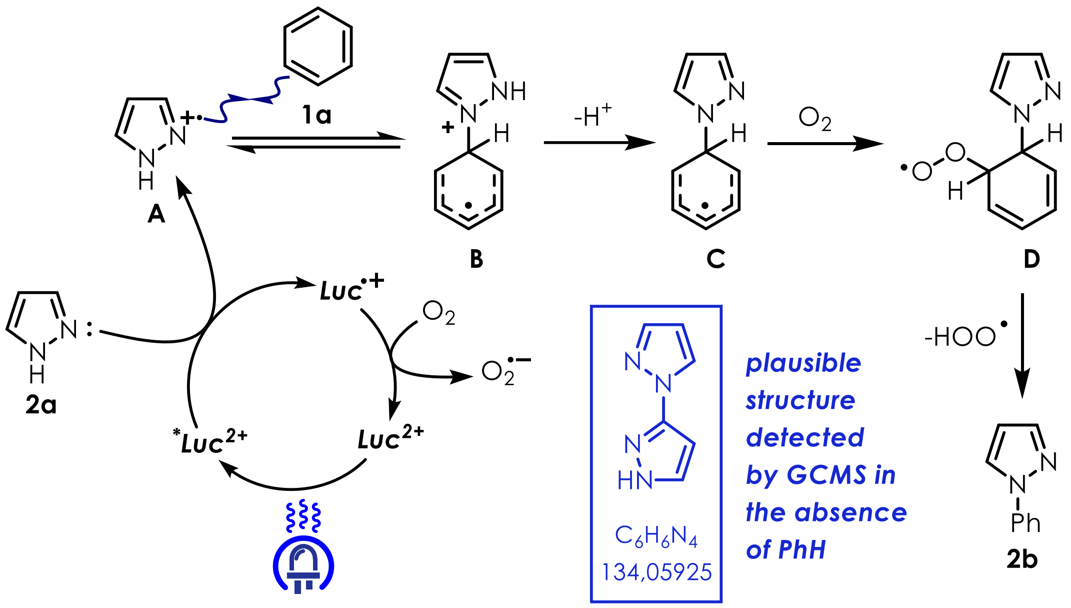


**Figure S65.** Mechanistic proposal.

Given the excess of benzene used under the standard reaction conditions, the proposed mechanism remains conjectural. Further study is required to substantiate the formation of the azole radical cation.

Several unanswered questions have also emerged from the optimisation campaign and the azole scope:

- The use of 220 equivalents of benzene, compared to 110 equivalents, significantly decreased the yield. This result suggests that solubility may also play a key role in this reactivity (*Note*: **Luc/BF4** is completely insoluble in benzene).
- Unlike previous reports, this protocol does not require a constant oxygen atmosphere.
- Electron-poor azoles react more efficiently than electron-rich ones, despite the expectation that electron-rich azoles should be more readily oxidized. Even if

benzene oxidation takes place, electron-rich azoles should exhibit greater nucleophilicity than their electron-poor counterparts.

The actual mechanism may involve an interplay between two “competing mechanisms”, where the oxidation of benzene or the oxidation of azole could predominate depending of the specific azole, arene, solvent or concentration used.

# 14 Comparison with the literature

## 14.1 Comparison with other methodologies utilising photooxidants

**Table S16** and **Table S17** are gathering the results from this work and most of the reports from the last 10 years regarding the oxidative C-H amination of arenes using a photooxidant. Yields reported in **Table S16** and **Table S17** were taken from the manuscripts cited in the main text (the corresponding author is cited in the tables for each one).

Overall, this work compares relatively well in term of yields on the “simple” substrates compared to the literature (see **Table S16** and **Table S17**). In particular, rows highlighted in light blue emphasise this direct comparison for pyrazole (**2a**), ethyl 4-pyrazolecarboxylate (**3a**) and pyrazole-4-carbonitrile (**12a**):

- For pyrazole (**2a**), our protocol provided the desired product in a higher yield than for the methods used by Wickens, Konig, Choudhury or Sanford and performed slightly worse compared to the work by the Raha Roy and Kerzig groups (using HFIP in both cases) (**Table S16**).
- For ethyl 4-pyrazolecarboxylate (**3a**) and pyrazole-4-carbonitrile (**12a**), our protocol performed relatively similar (or slightly worse) compared to all previous methods (**Table S16**).

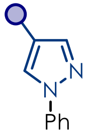

**Table S16.** Yield comparison with literature for some of the substrates from the scope

| Substrate | ○ | ***Yield (%)*** This work | Raha Roy | Kerzig | Wickens | König | Barham | Lambert | Choudhury | Sanford |
|---|---|---|---|---|---|---|---|---|---|---|
| **2b** | **H** | 70 | 83 | 78 | 38 | 22 | | | 40 | 62 |
| **3b** | **$CO_2Et$** | 75 | 86 | 87 | 81 | | 81 | 65 | 83 | 40 |
| **4b** | **Me** | 36 | 64 | 45 | 22 | | | | | |
| **5b** | **F** | 40 | 76 | | | | | | | |
| **6b** | **Cl** | 51 | 91 | 61 | 58 | | | 45 | 80 | 31 |
| **7b** | **Br** | 35 | 79 | 52 | 45 | 74 | 62 | | 70 | |
| **8b** | **CHO** | 52 | 80 | 72 | 71 | | | 61 | 87 | 40 |
| **9b** | **C(O)Me** | 70 | 78 | 81 | 88 | | 58 | 60 | | 46 |
| **10b** | **$CF_3$** | 66 | 82 | 74 | 70 | | | | | 64 |
| **11b** | **CN** | 74 | 96 | 81 | 87 | | 76 | | | 80 |
| **12b** | **$NO_2$** | 73 | | | | | | | 82 | 78 |

**Table S17.** Yield comparison with literature for some of the substrates from the scope

| Substrate | | ***Yield (%)*** This work | Raha Roy | Kerzig | Wickens | König | Barham | Lambert | Choudhury | Sanford |
|---|---|---|---|---|---|---|---|---|---|---|
| **30b** | 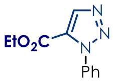 | 72 | 81 | 68 | | | 45 | | | |
| **33b** | 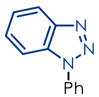 | 46 | 77 | 71 | | | 49 | | 75 | |

## 14.2 Comparison with cross-coupling methods (Chan-Lam/Ullmann)

This part aims at showing the relevance of the protocol disclosed in this manuscript in comparison to the cross-coupling literature that usually employed Ullmann or Chan-Lam conditions to synthesise some *N*-arylated compounds (often containing nitro moieties as they are useful in medicinal chemistry to introduce an amide bond).

Some of the yields shown in the main text are quite comparable to what is obtained in the literature (**Figure S66**, **Figure S67** and **Figure S68**). Therefore, this methodology can offer a nice alternative to this classical cross-coupling reactions which require the use of a metal catalyst, a prefunctionalised arene substrates (*i.e.* iodobenzene or phenylboronic acid) or even forcing conditions in some cases (**Figure S67**, **Figure S68** and **Figure S70**).

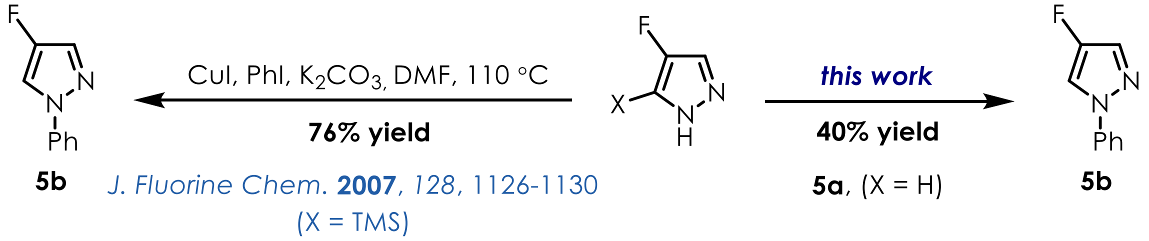


**Figure S66.** Direct oxidative azole protocol versus literature protocol using Ullmann conditions for substrate **5a**.

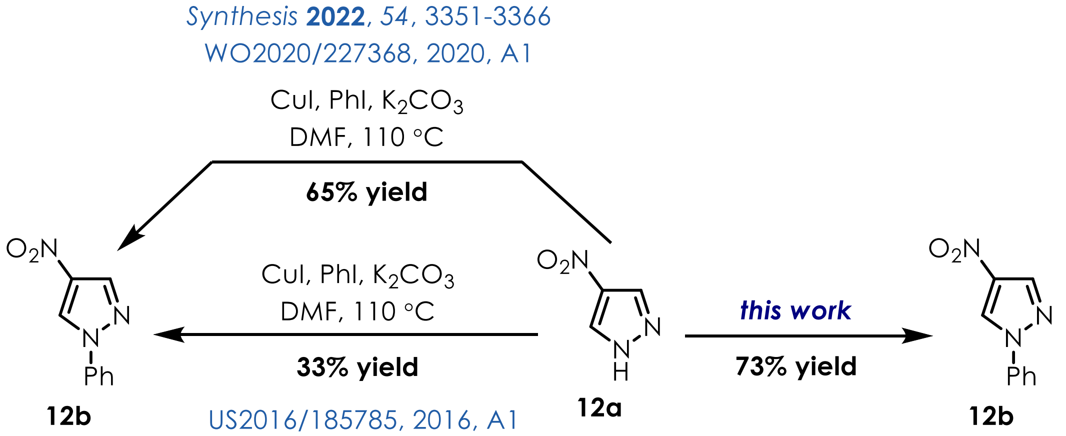


**Figure S67.** Direct oxidative azole protocol versus literature protocol using Ullmann conditions for substrate **12a**.

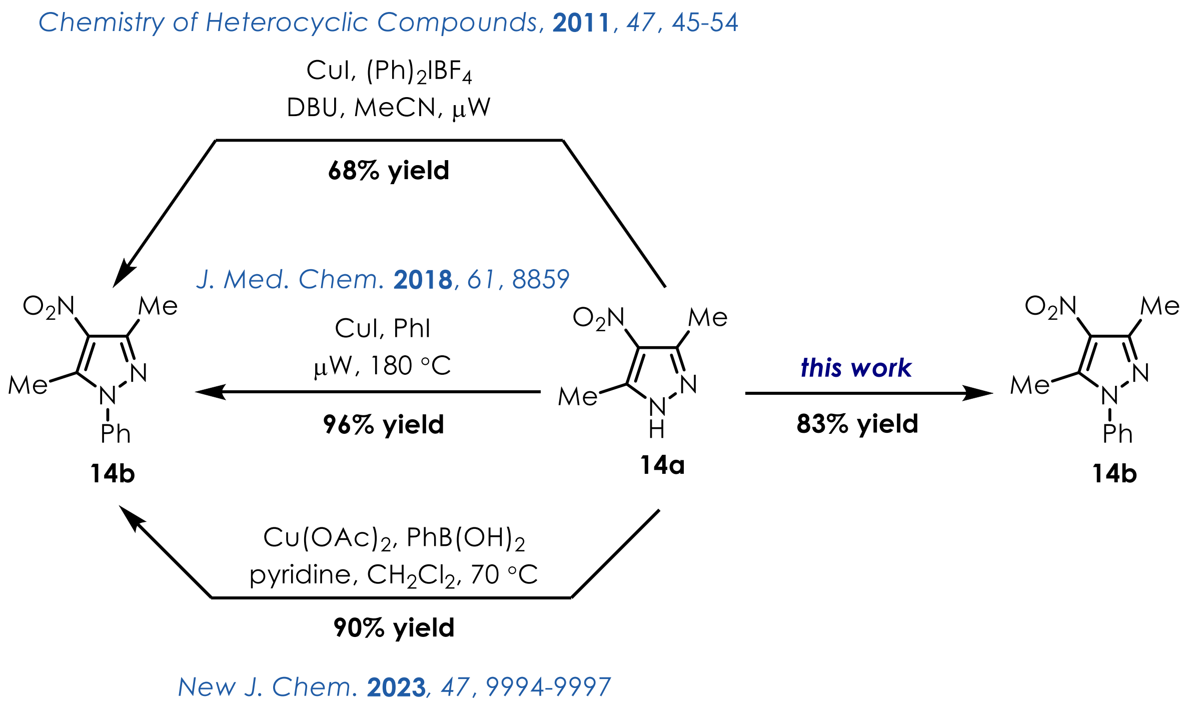


**Figure S68.** Direct oxidative azole protocol versus literature protocol using Chan-Lam or Ullmann conditions for substrate **14a**.

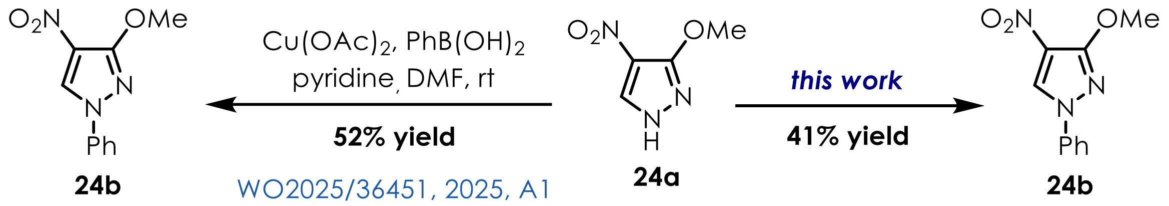


**Figure S69.** Direct oxidative azole protocol versus literature protocol using Chan-Lam conditions for substrate **24a**.

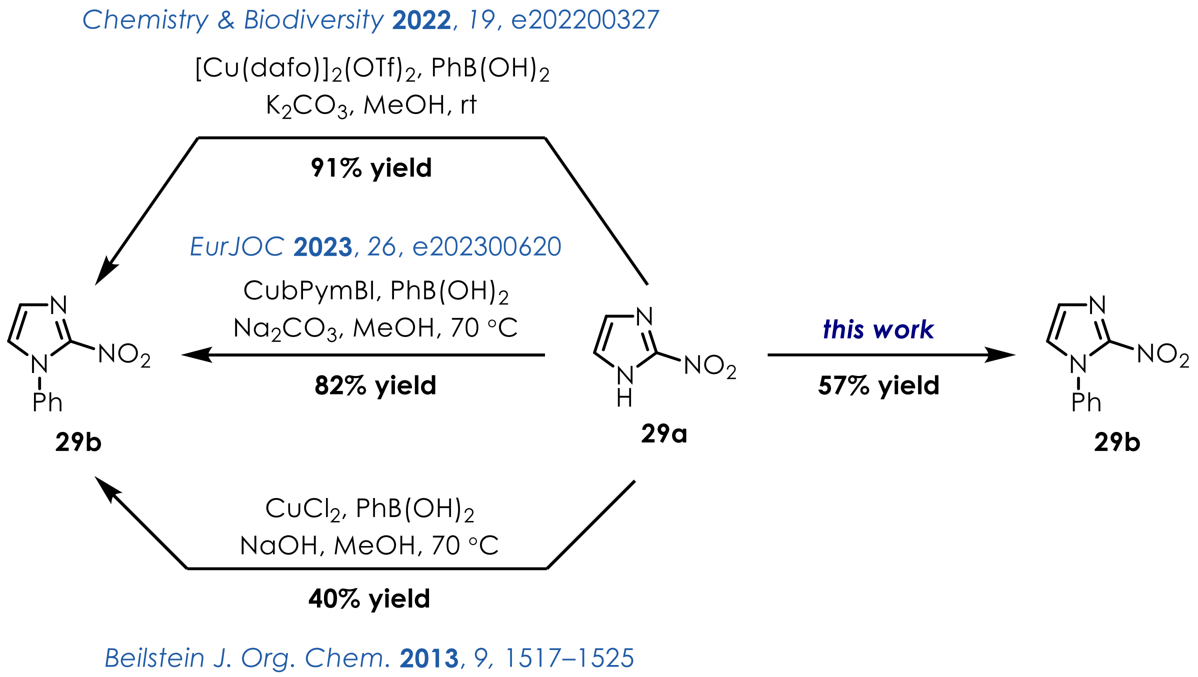


**Figure S70.** Direct oxidative azole protocol versus literature protocol using Chan-Lam conditions for substrate **29a**. dafo = 4,5-Diazafluoren-9-one; bPymBI = 1,3-bis(pyridine-2-ylmethyl)-1*H*-benzo[*d*]imidazole-3-ium

## 15 NMR Spectra

### Luc/ClO4 – $^{1}$H NMR (600 MHz, DMSO-$d_6$)

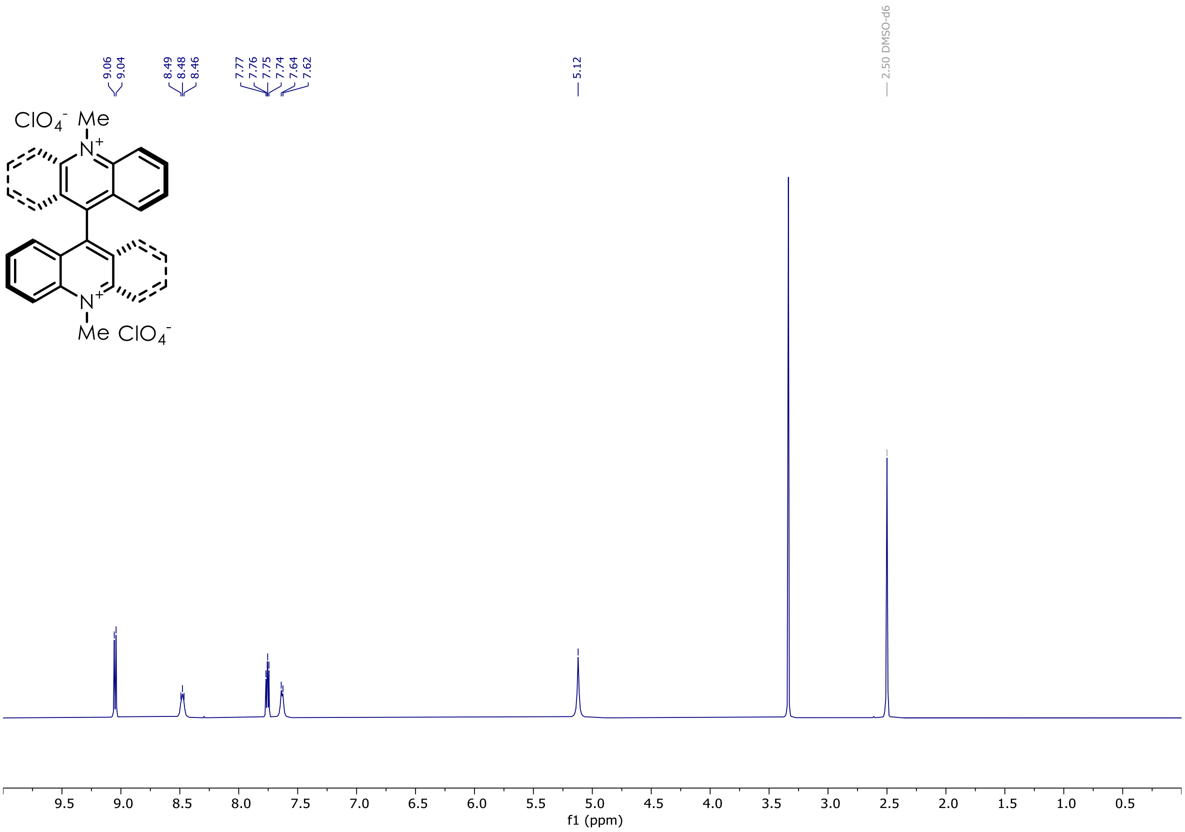


### Luc/ClO4 – $^{13}$C NMR (151 MHz, DMSO-$d_6$)

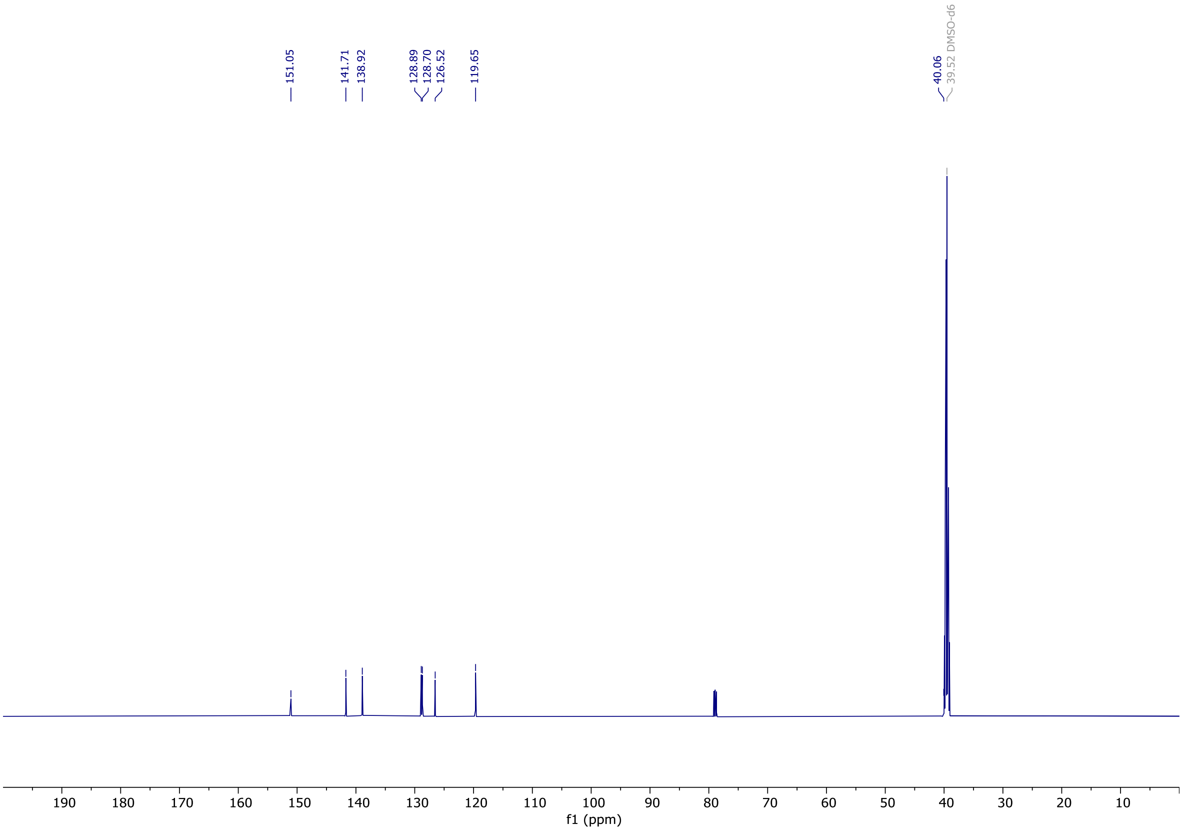

## Luc/BF$_4$ – $^1$H NMR (600 MHz, DMSO-$d_6$)

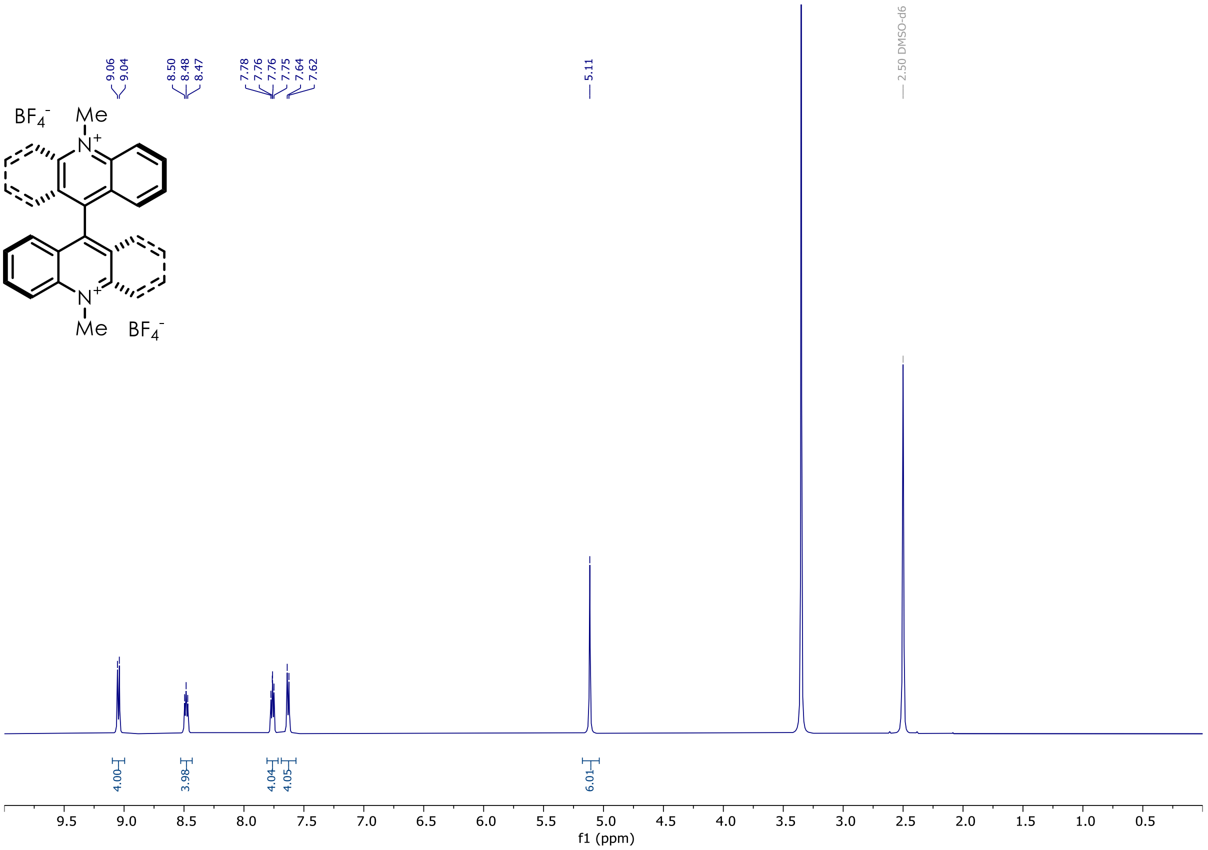


## Luc/BF$_4$ – $^{13}$C NMR (151 MHz, DMSO-$d_6$)

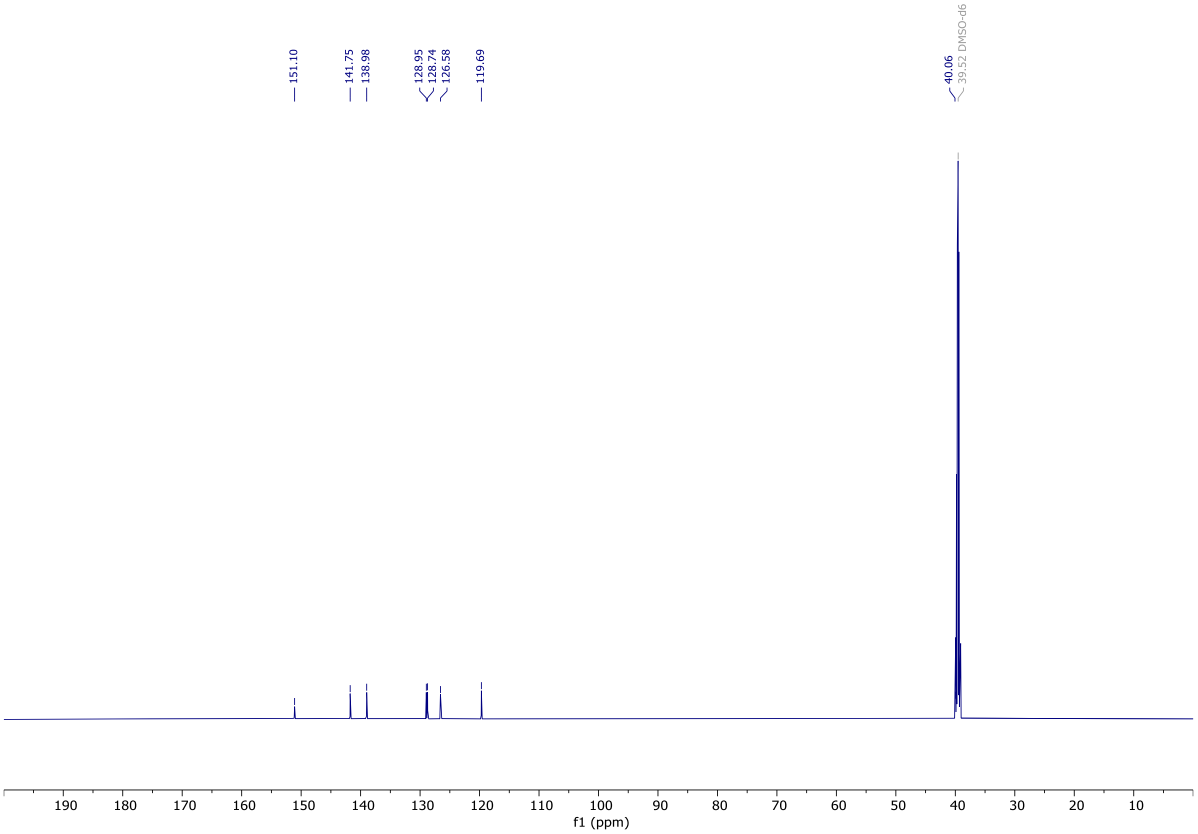

**Luc/BF$_4$ – $^{19}$F NMR (565 MHz, DMSO-$d_6$)**

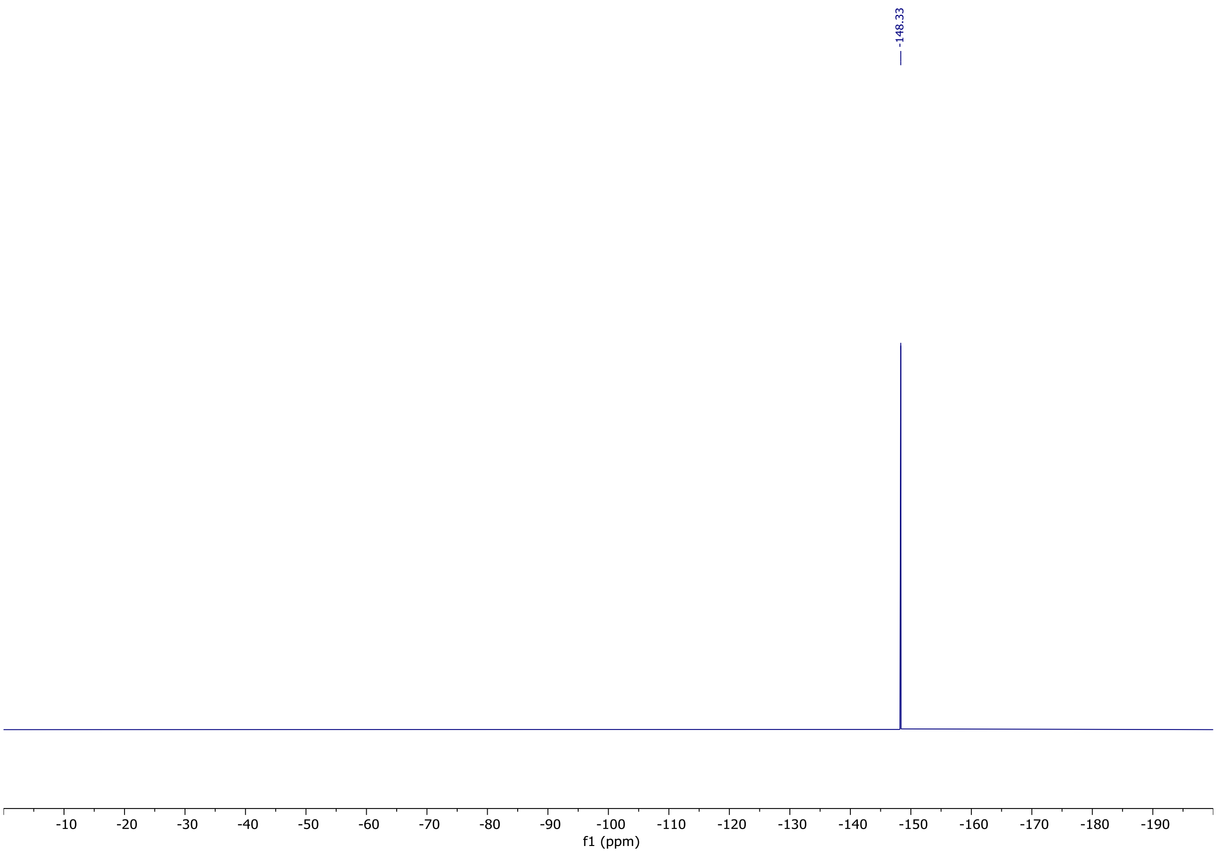


**Luc/BF$_4$ – $^{11}$B NMR (193 MHz, DMSO-$d_6$)**

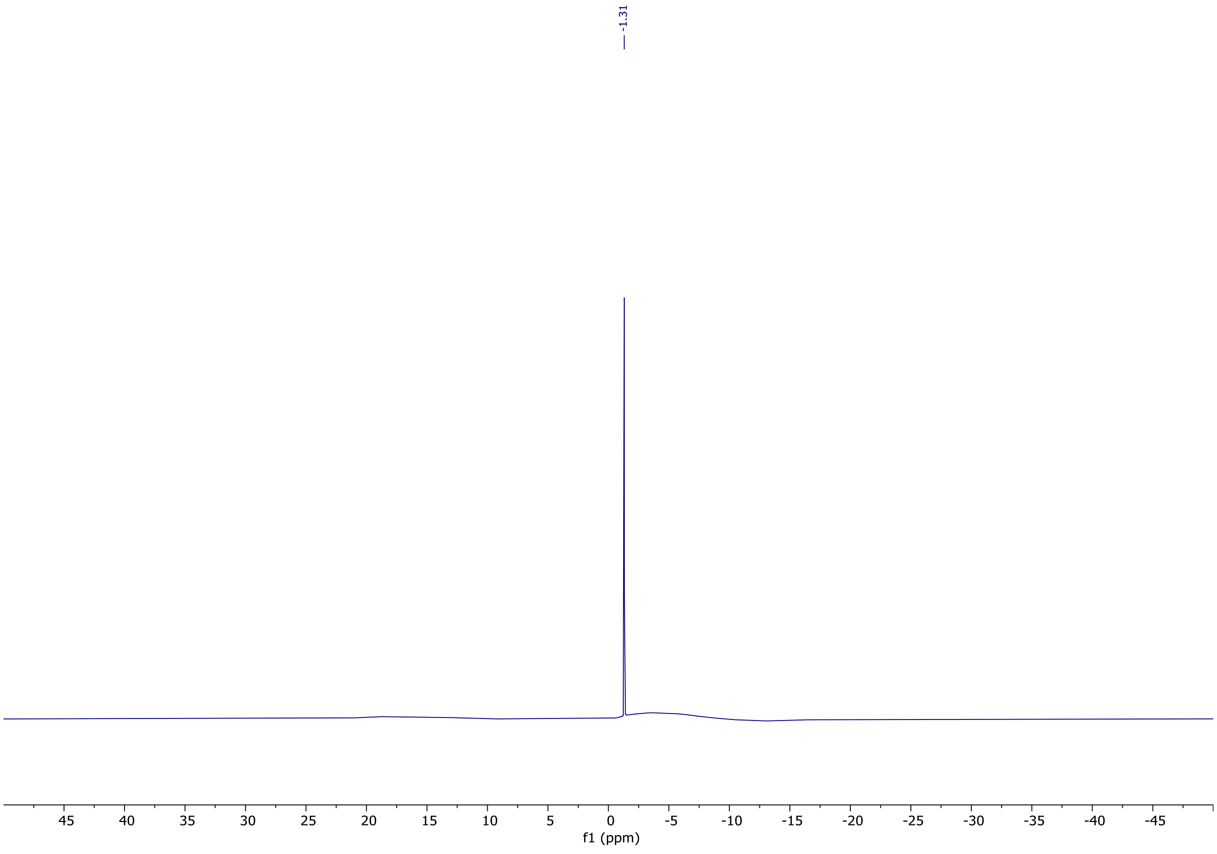

## Luc/OTf – $^{1}$H NMR (600 MHz, DMSO-$d_6$)

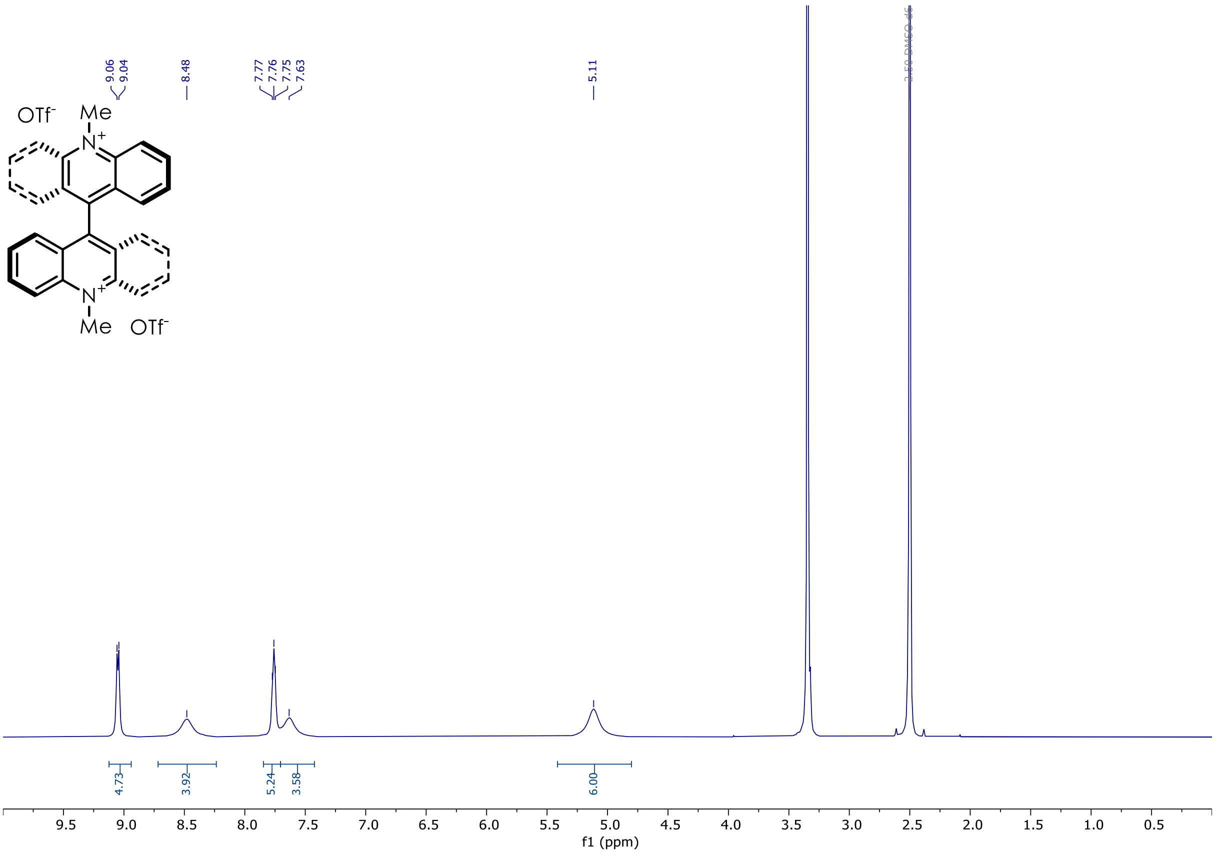


## Luc/OTf – $^{19}$F NMR (565 MHz, DMSO-$d_6$)

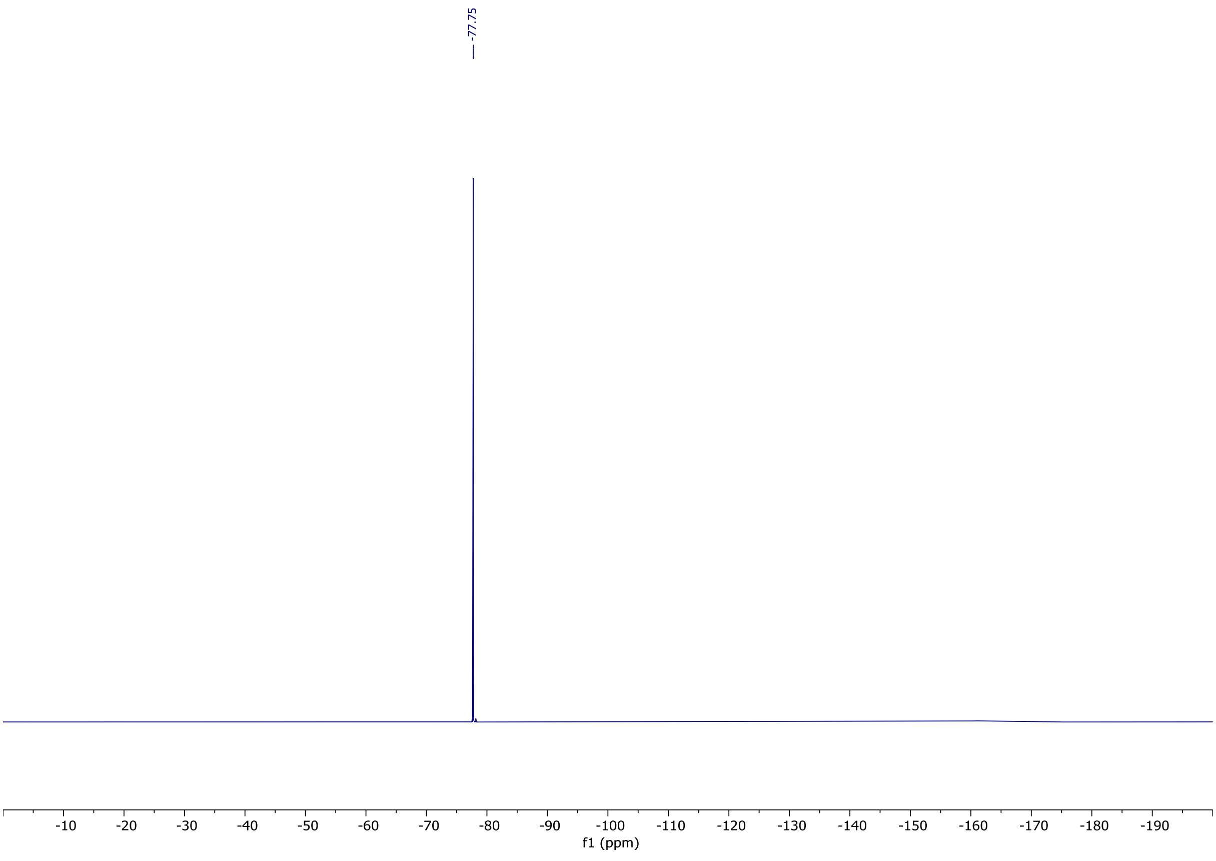

## Luc/PF$_6$ – $^1$H NMR (600 MHz, DMSO-$d_6$)

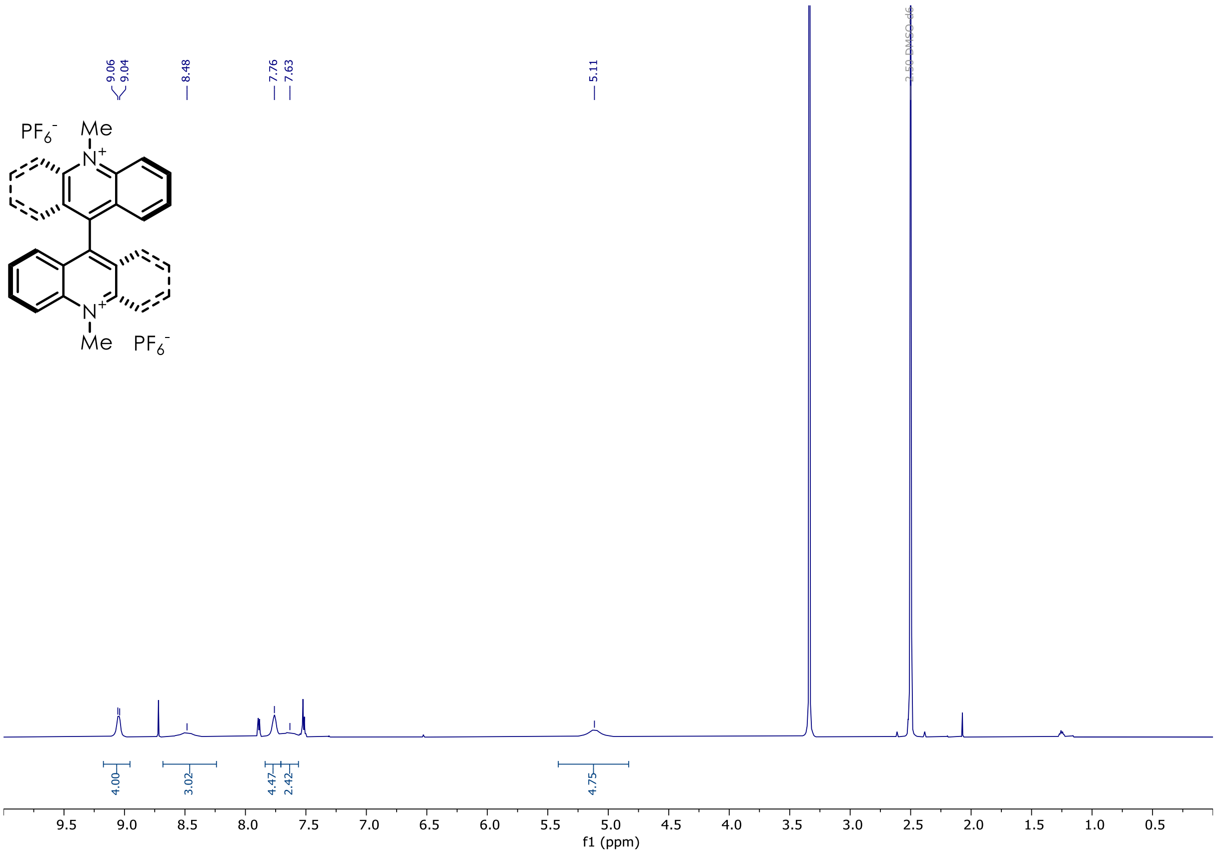


## Luc/PF$_6$ – $^{19}$F NMR (565 MHz, DMSO-$d_6$)

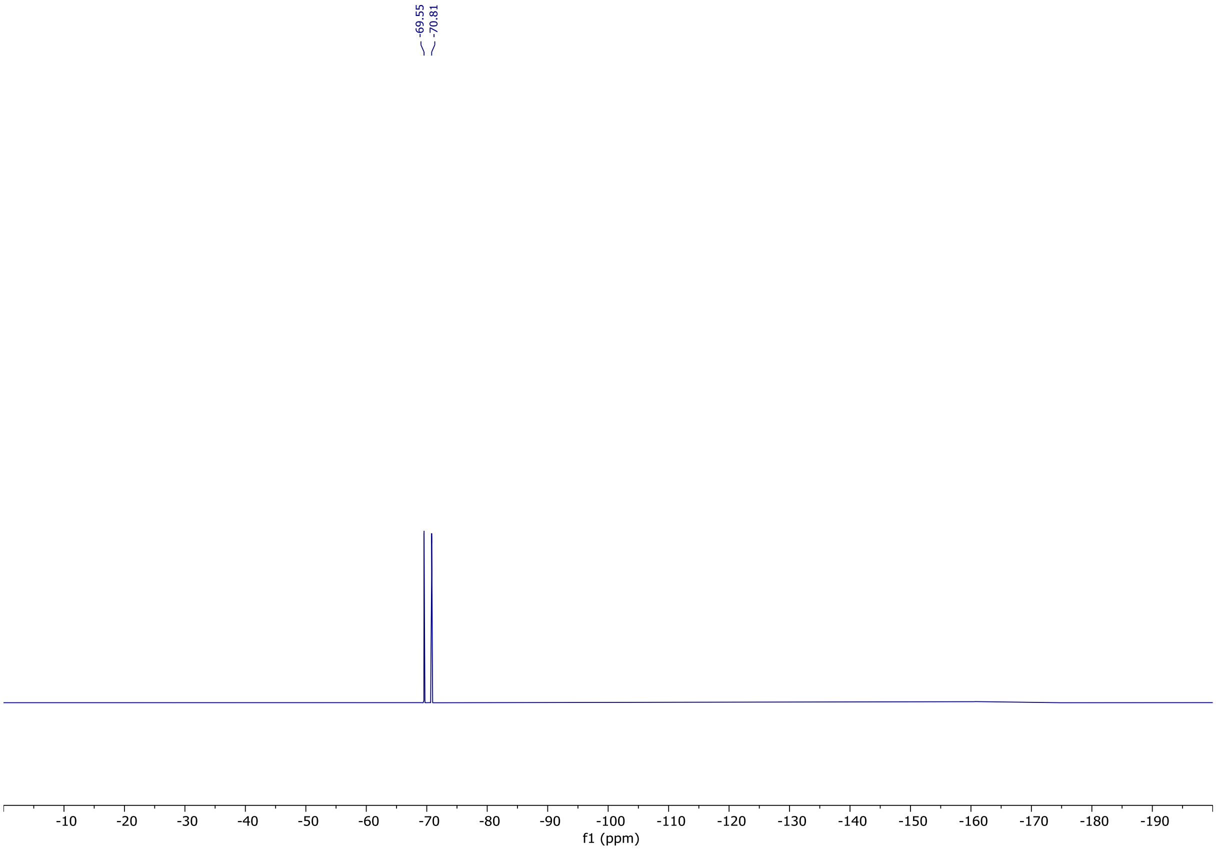

## Luc/BArF$_4$ – $^{1}$H NMR (600 MHz, DMSO-$d_6$)

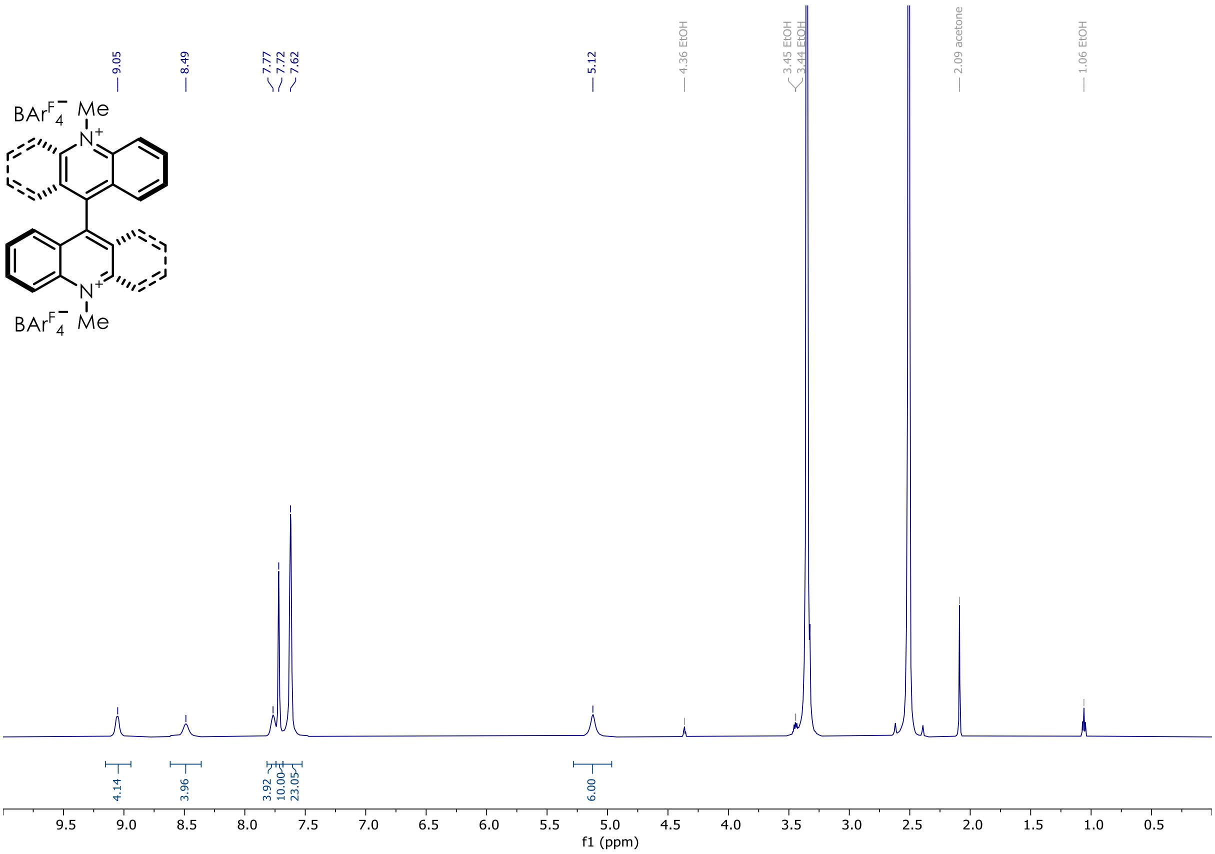


## Luc/BArF$_4$ – $^{13}$C NMR (151 MHz, DMSO-$d_6$)

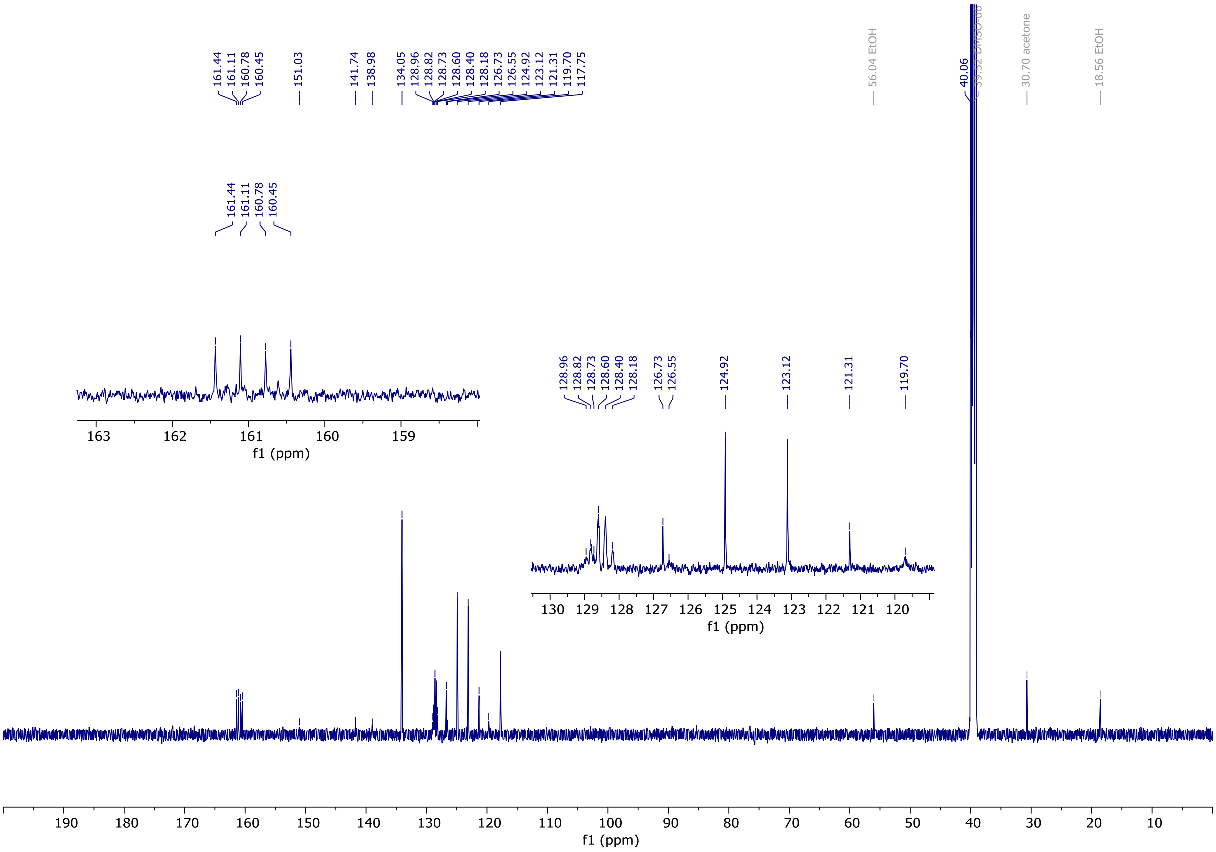

## Luc/BAr$^F_4$ – $^{19}$F NMR (565 MHz, DMSO-$d_6$)

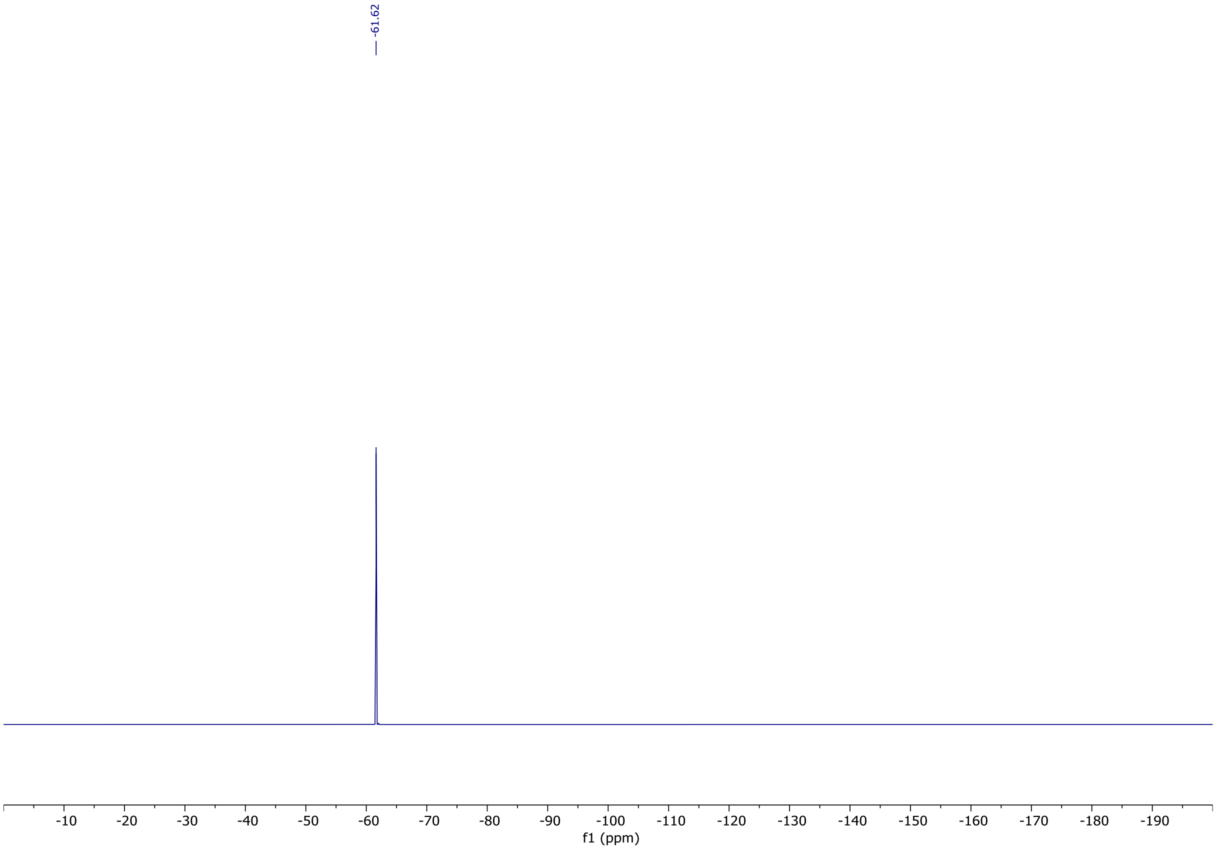


## Luc/BAr$^F_4$ – $^{11}$B NMR (193 MHz, DMSO-$d_6$)

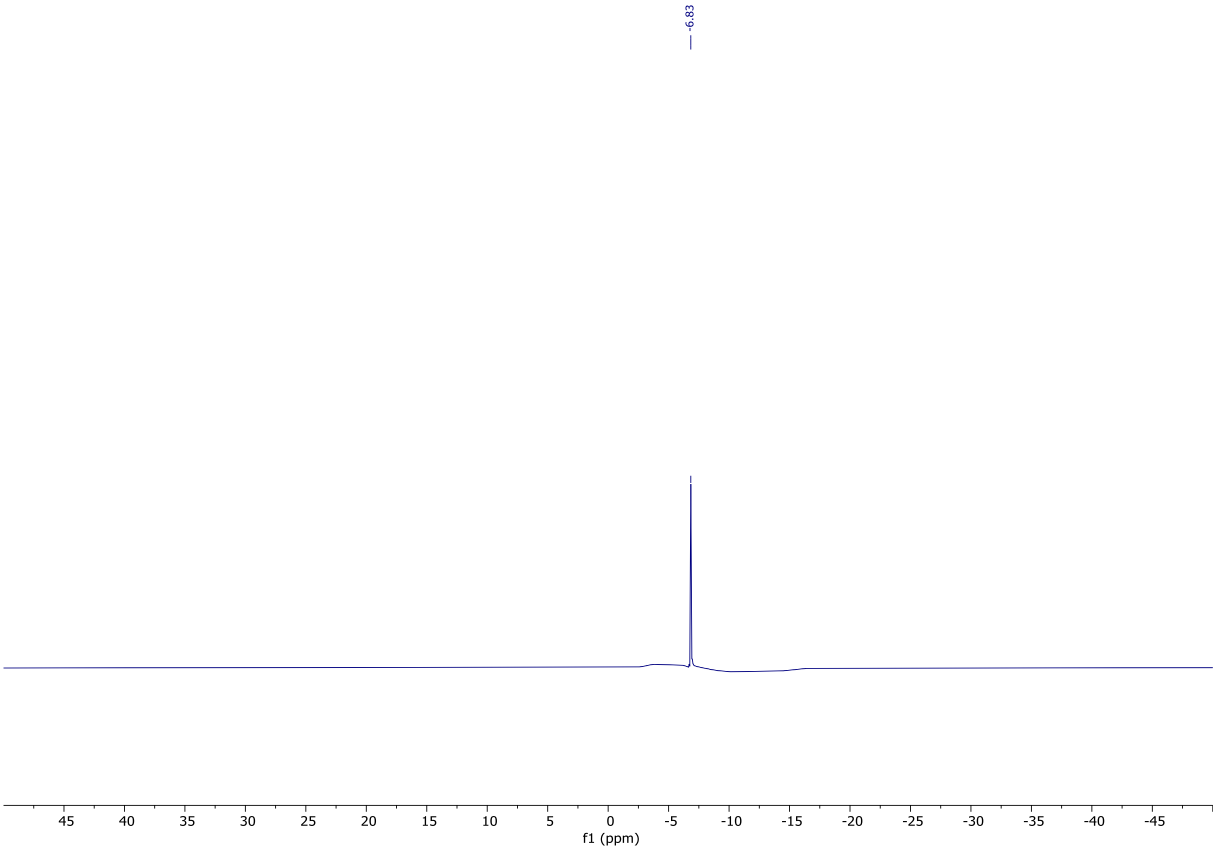

## A/NO3 – $^{1}$H NMR (600 MHz, DMSO-*d6*)

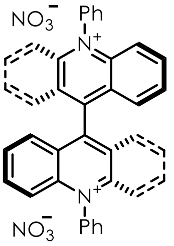


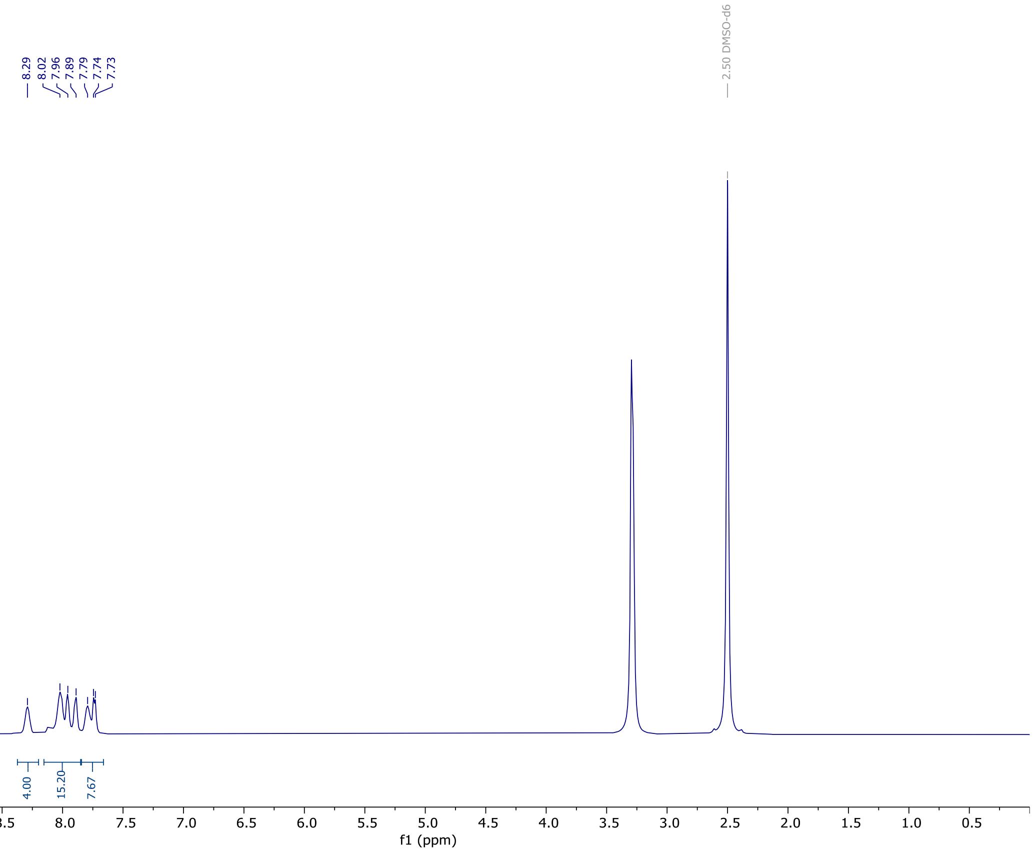


## A/NO3 – $^{13}$C NMR (151 MHz, DMSO-*d6*)

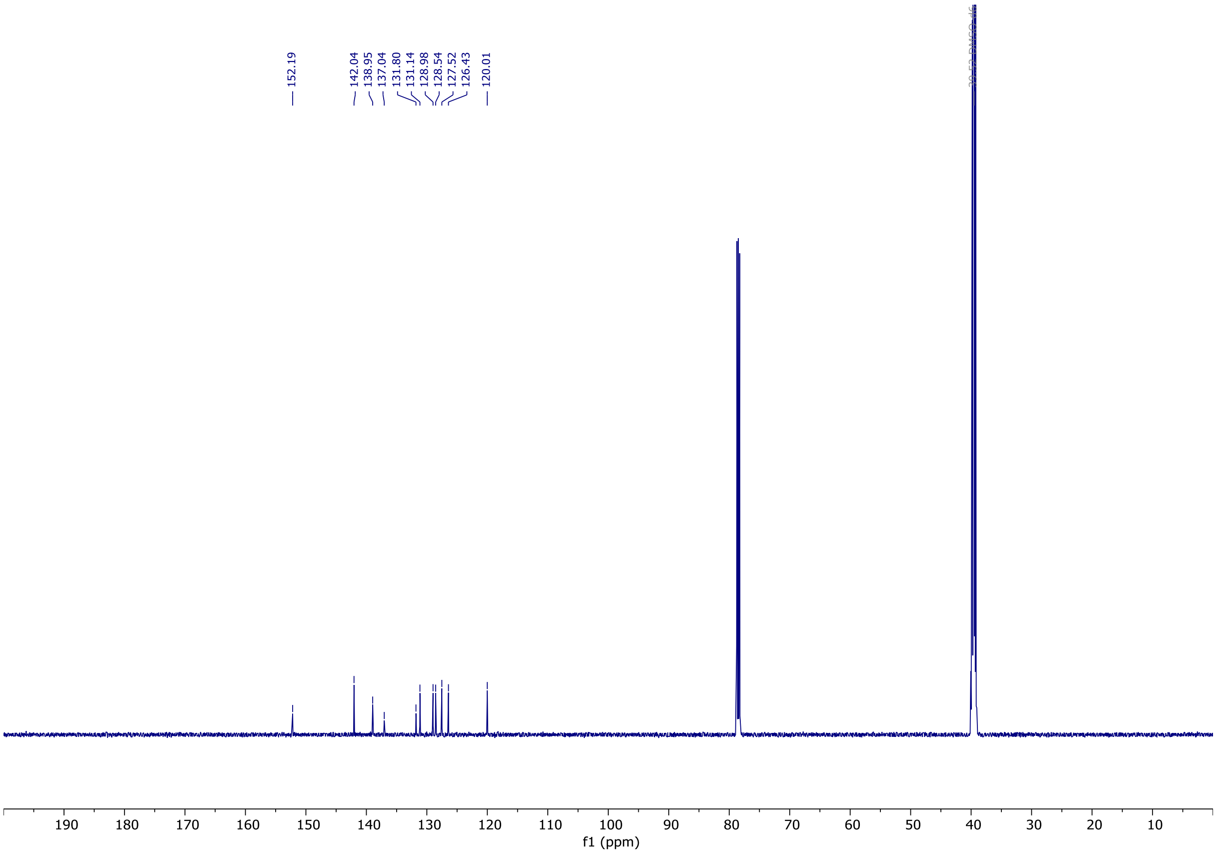

## 2b – $^{1}$H NMR (600 MHz, $CDCl_3$)

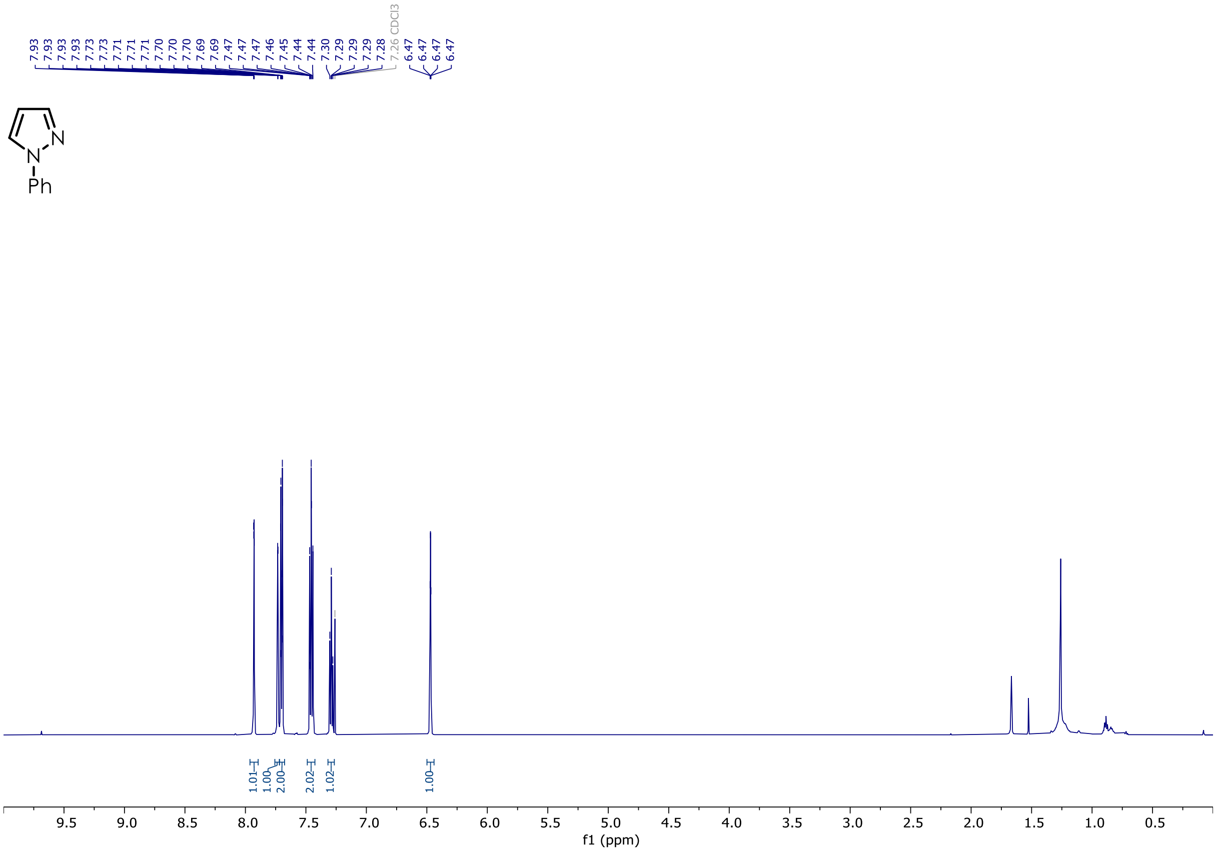


## 2b – $^{13}$C NMR (151 MHz, $CDCl_3$)

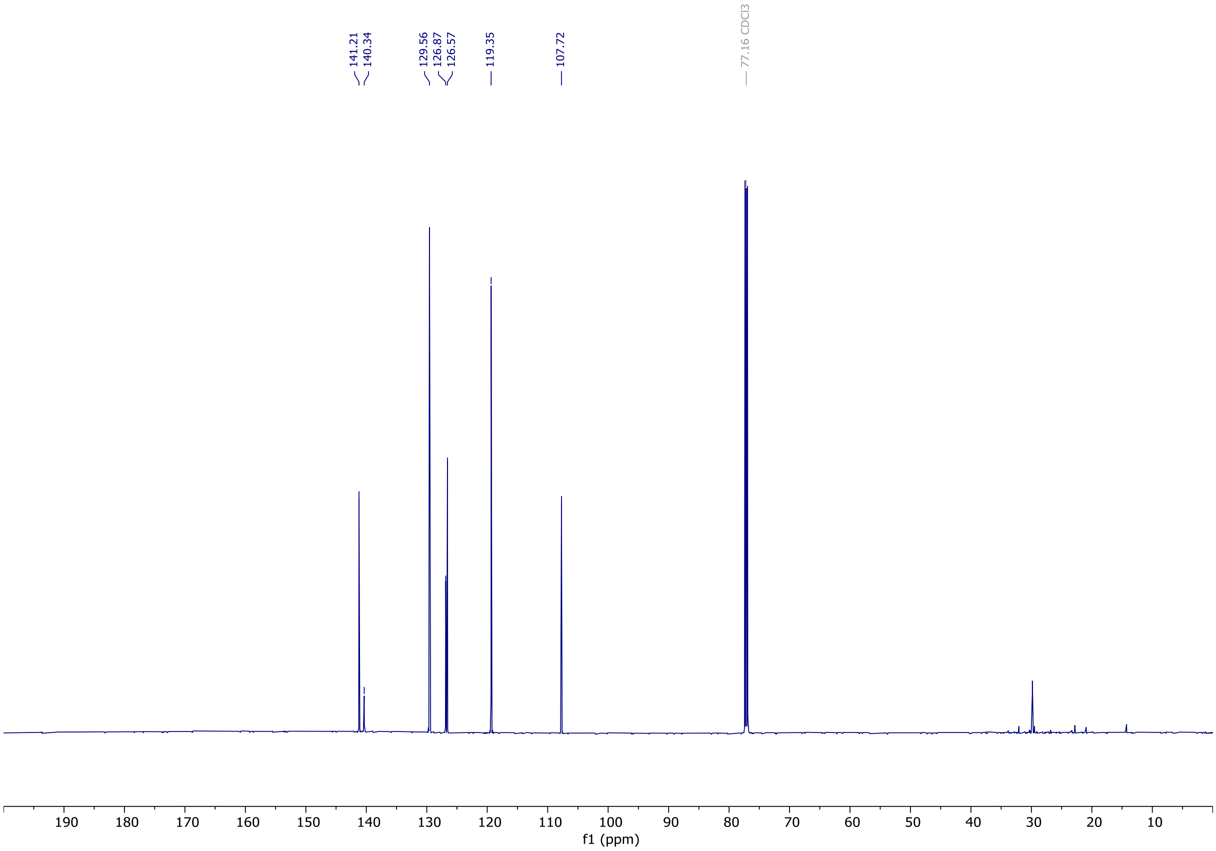

## 3b – $^{1}$H NMR (600 MHz, $CDCl_3$)

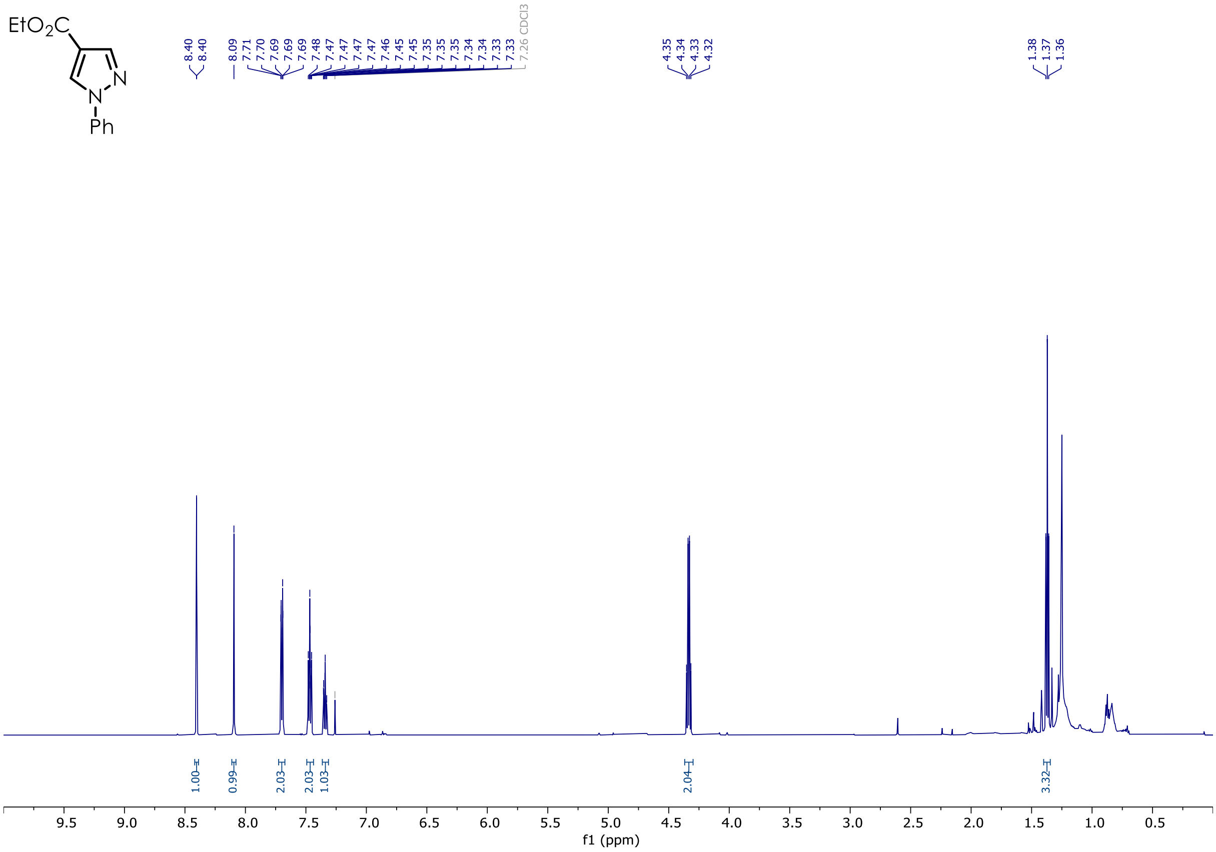


## 3b – $^{13}$C NMR (151 MHz, $CDCl_3$)

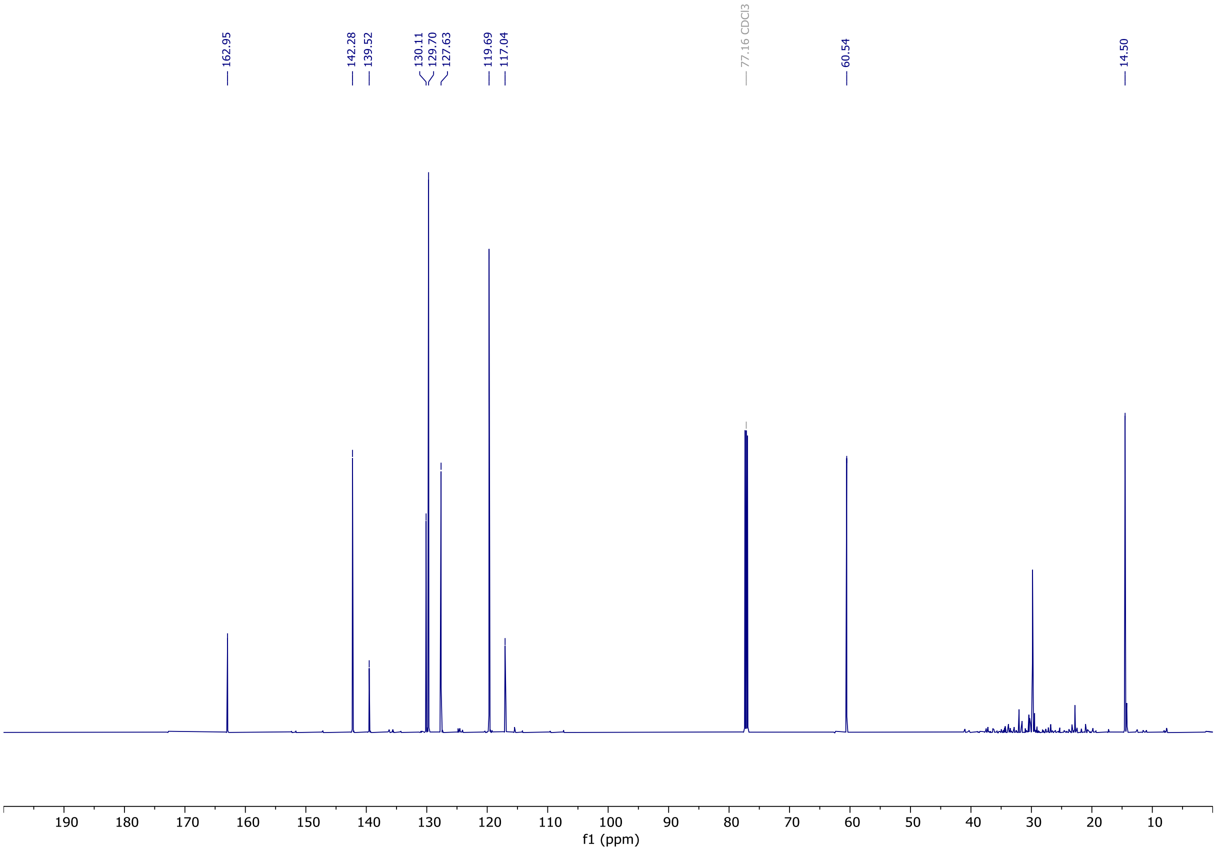

**4b – $^1$H NMR (600 MHz, $CDCl_3$)**

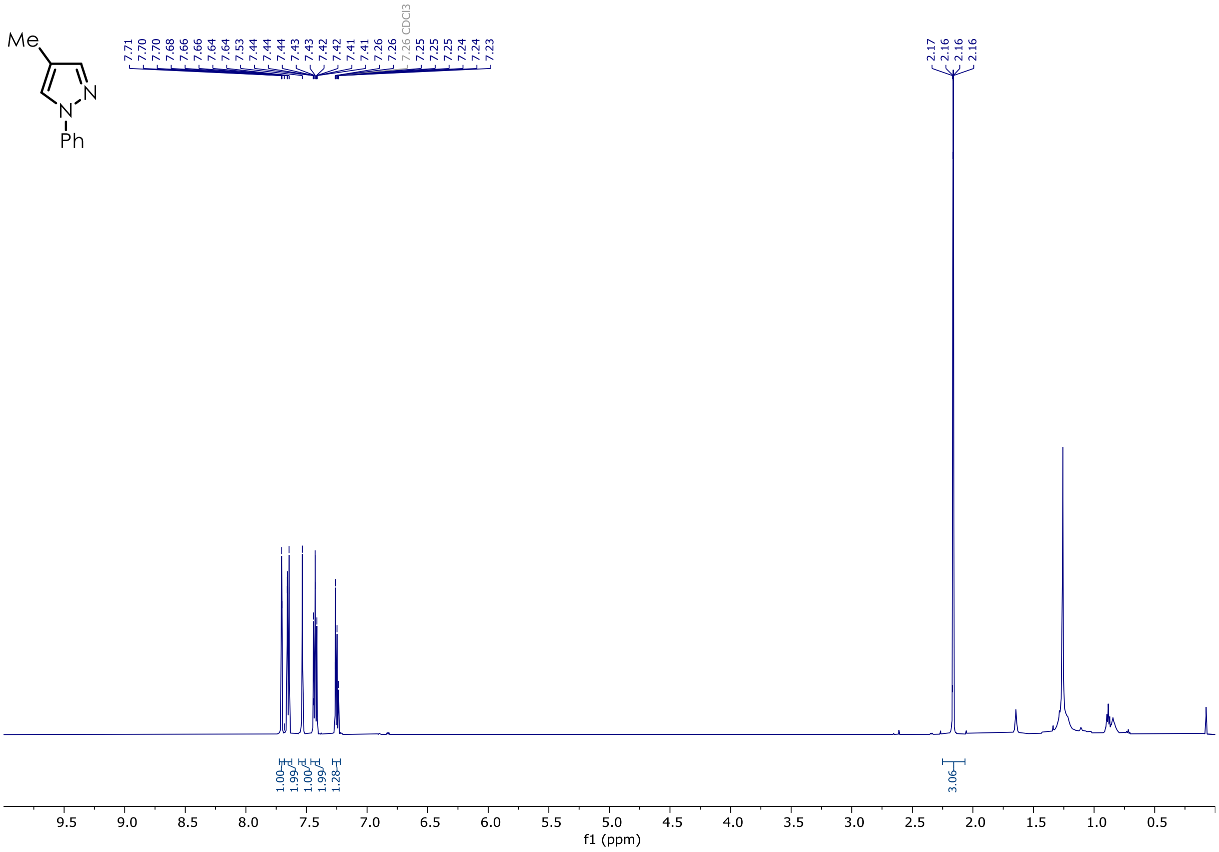

**4b – $^{13}$C NMR (151 MHz, $CDCl_3$)**

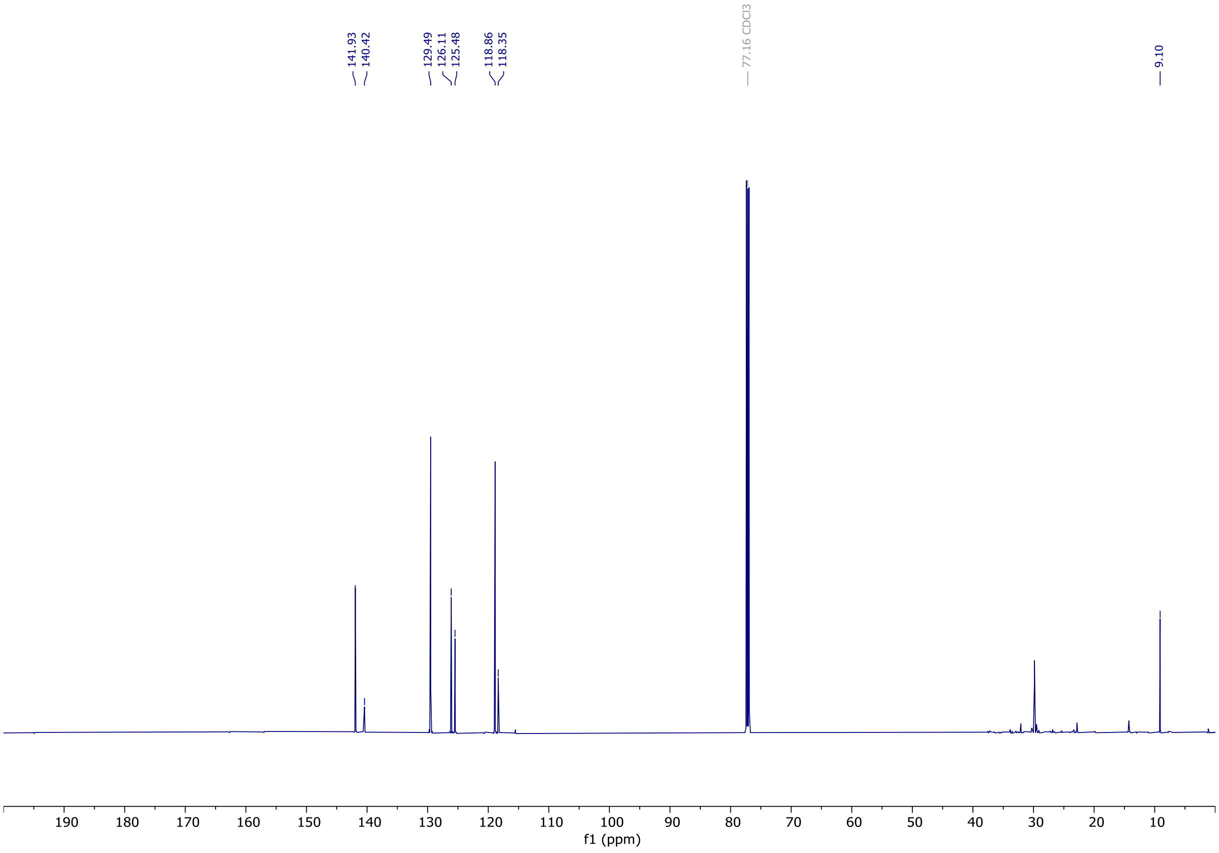

## 5b – $^{1}H$ NMR (600 MHz, $CDCl_3$)

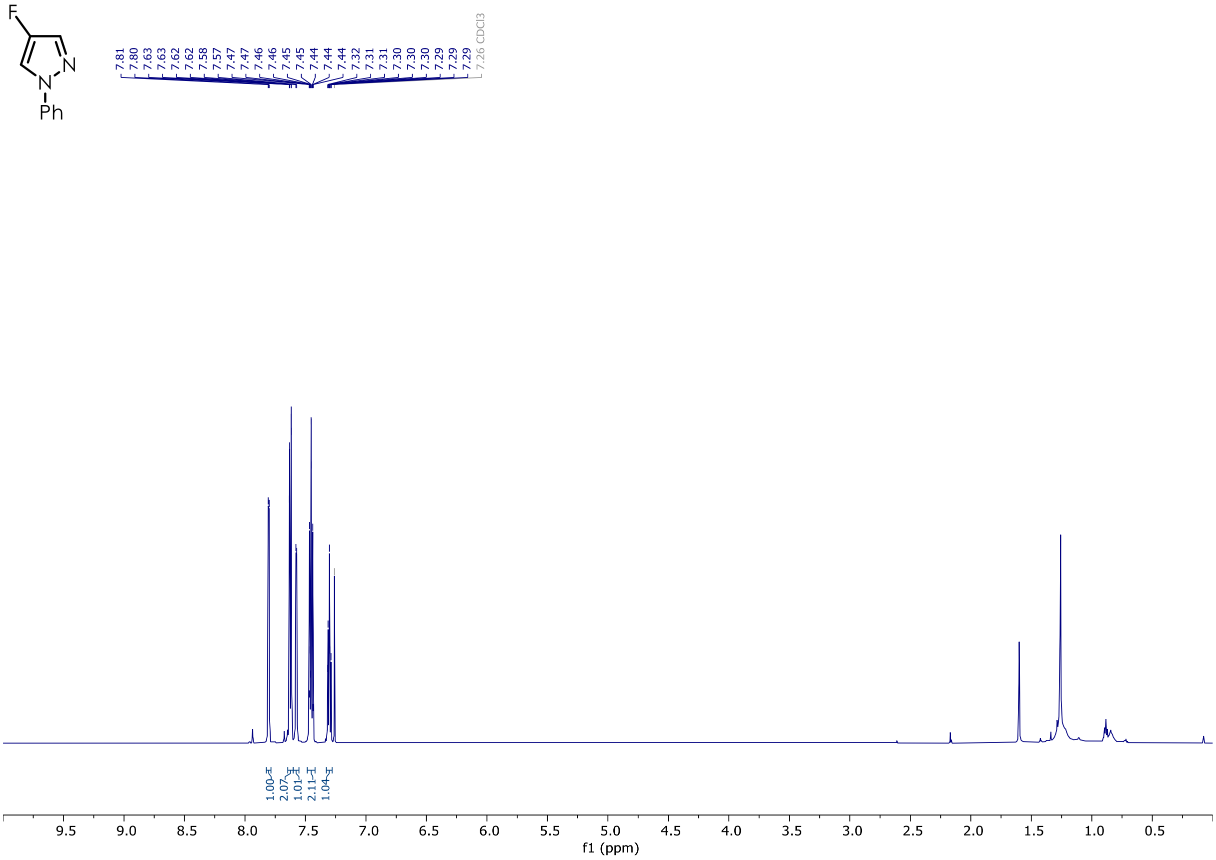


## 5b – $^{13}C$ NMR (151 MHz, $CDCl_3$)

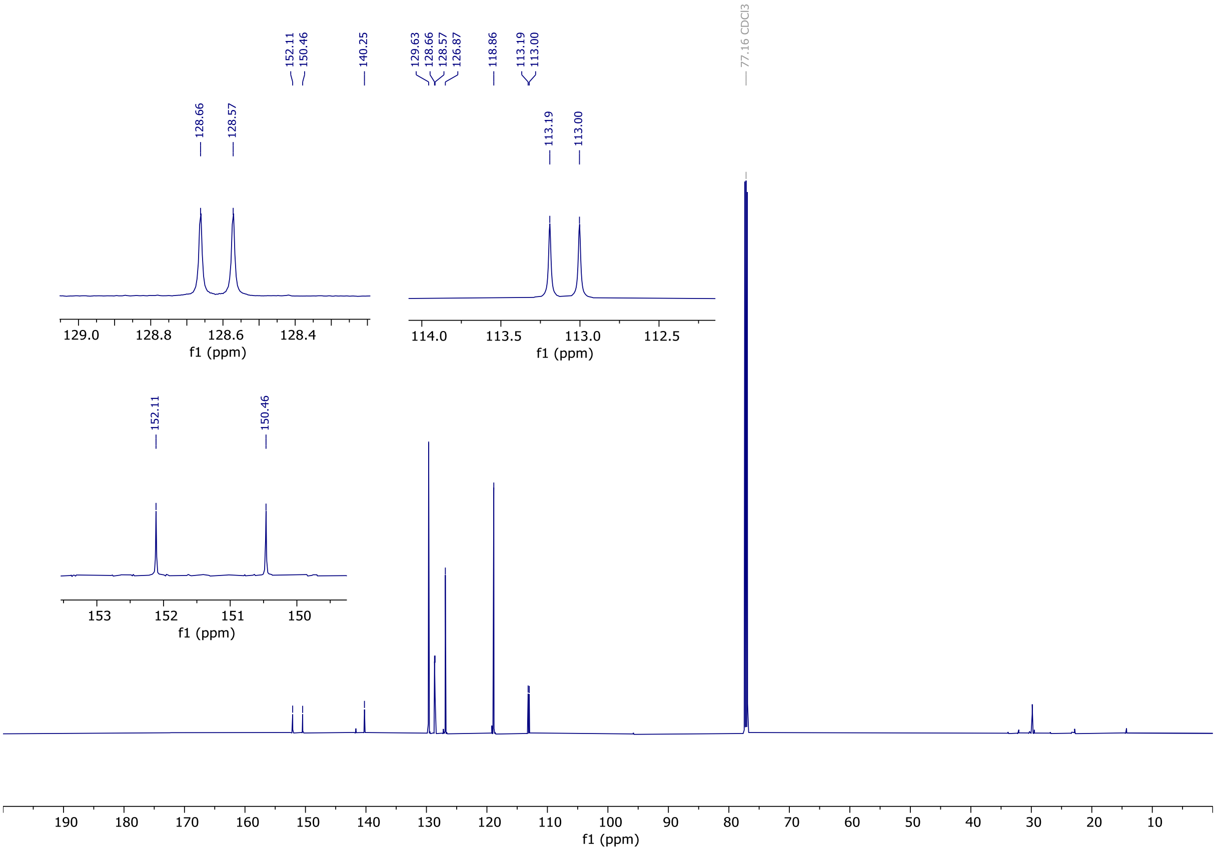

**5b – $^{19}$F NMR (565 MHz, $CDCl_3$)**

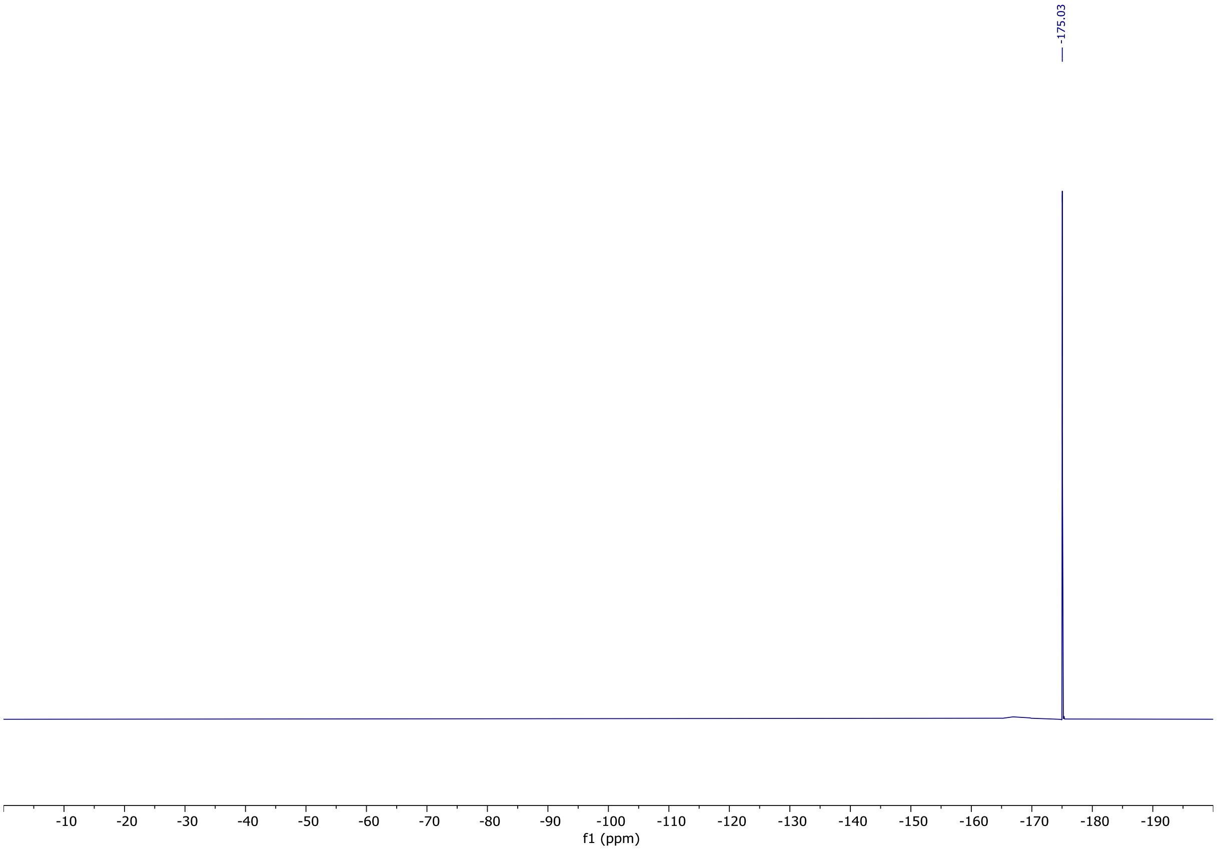

## 6b – $^{1}$H NMR (600 MHz, $CDCl_3$)

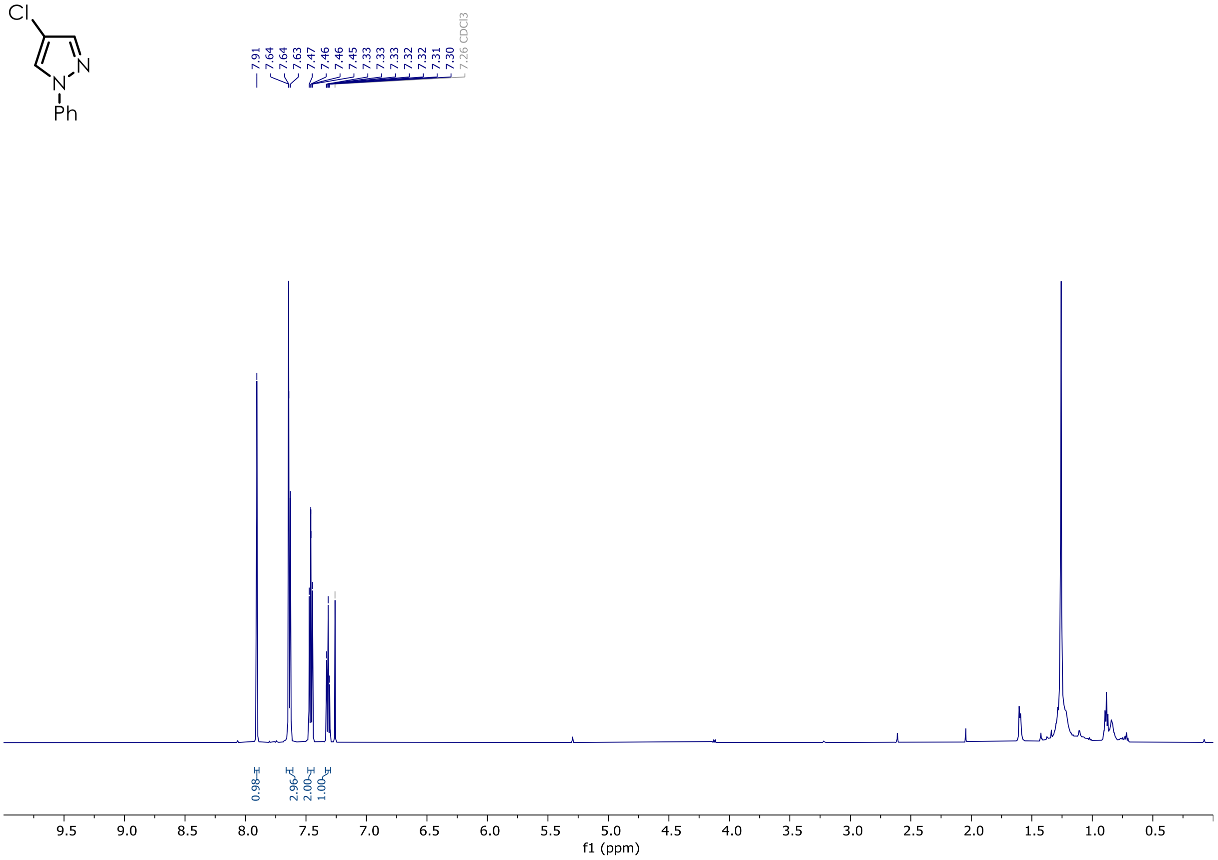


## 6b – $^{13}$C NMR (151 MHz, $CDCl_3$)

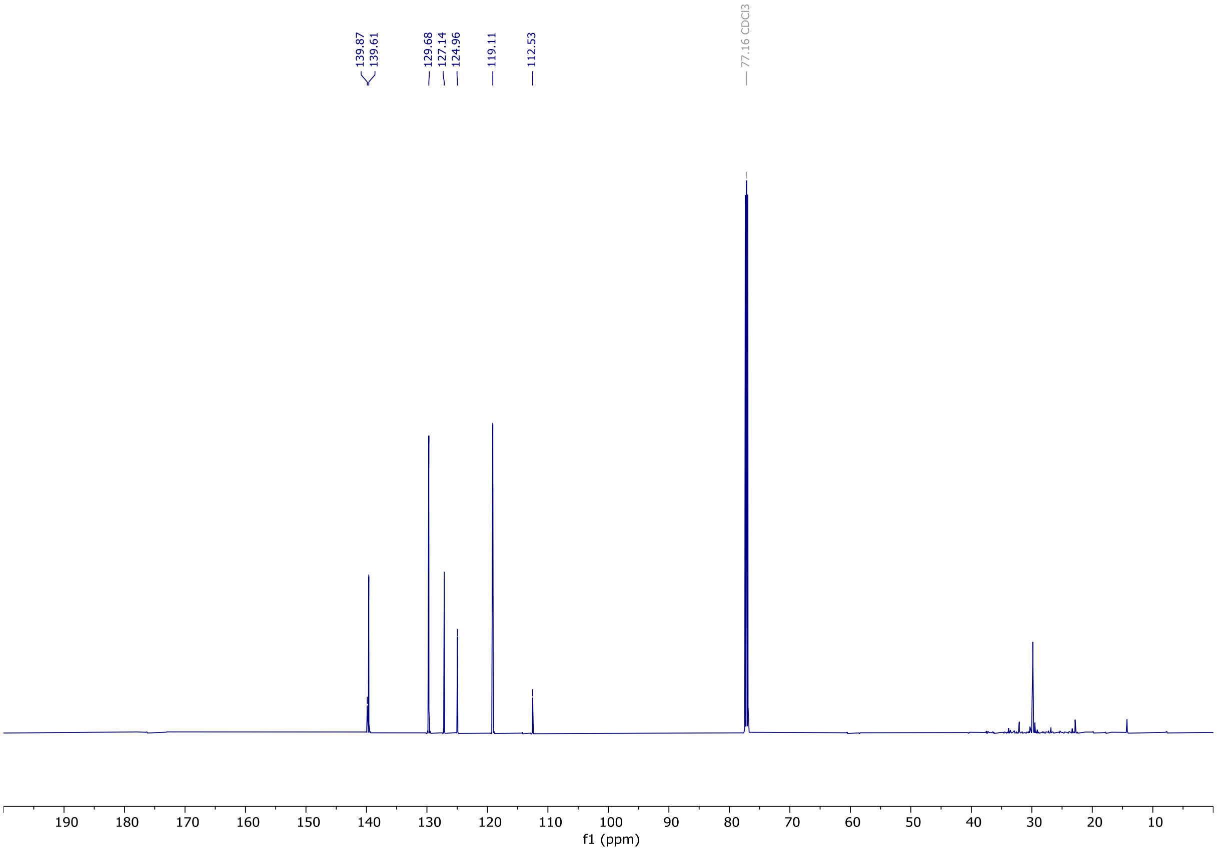

## 7b – $^{1}$H NMR (600 MHz, CDCl$_3$)

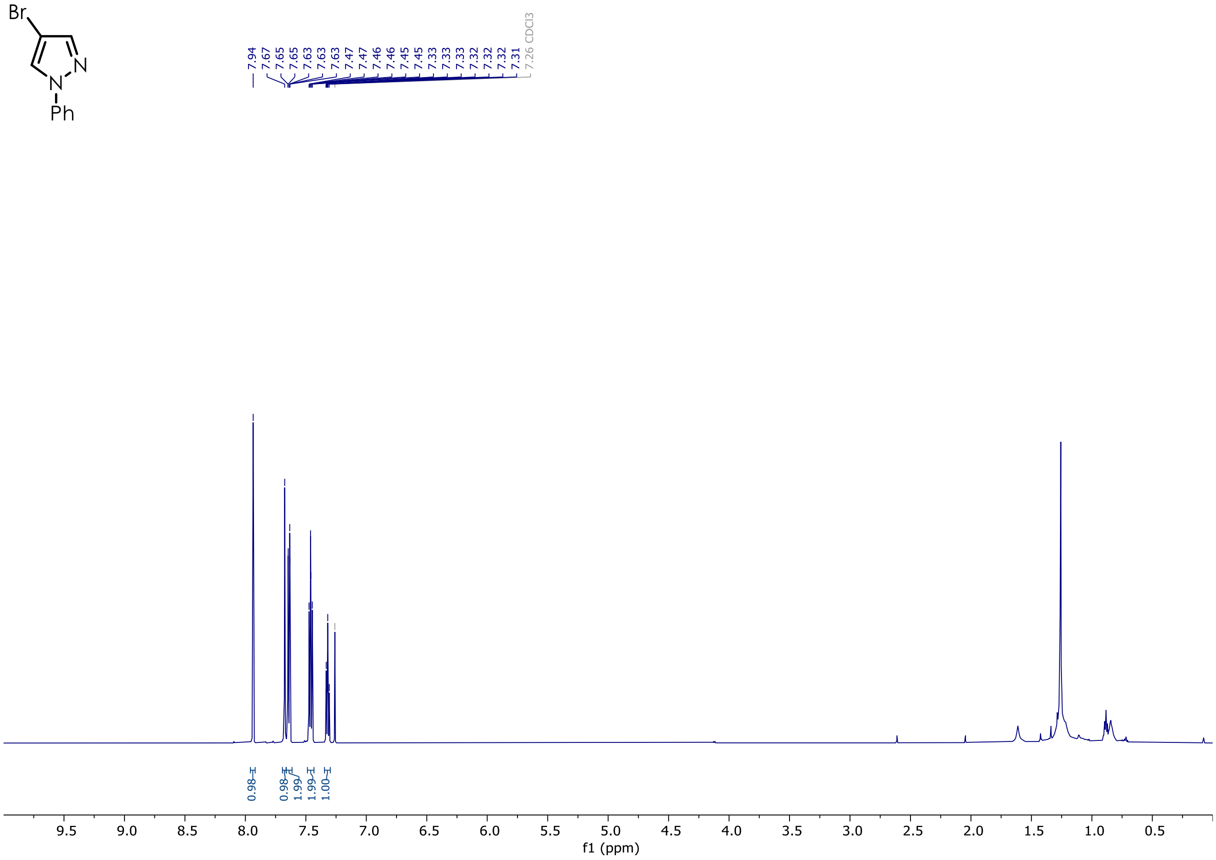


## 7b – $^{13}$C NMR (151 MHz, CDCl$_3$)

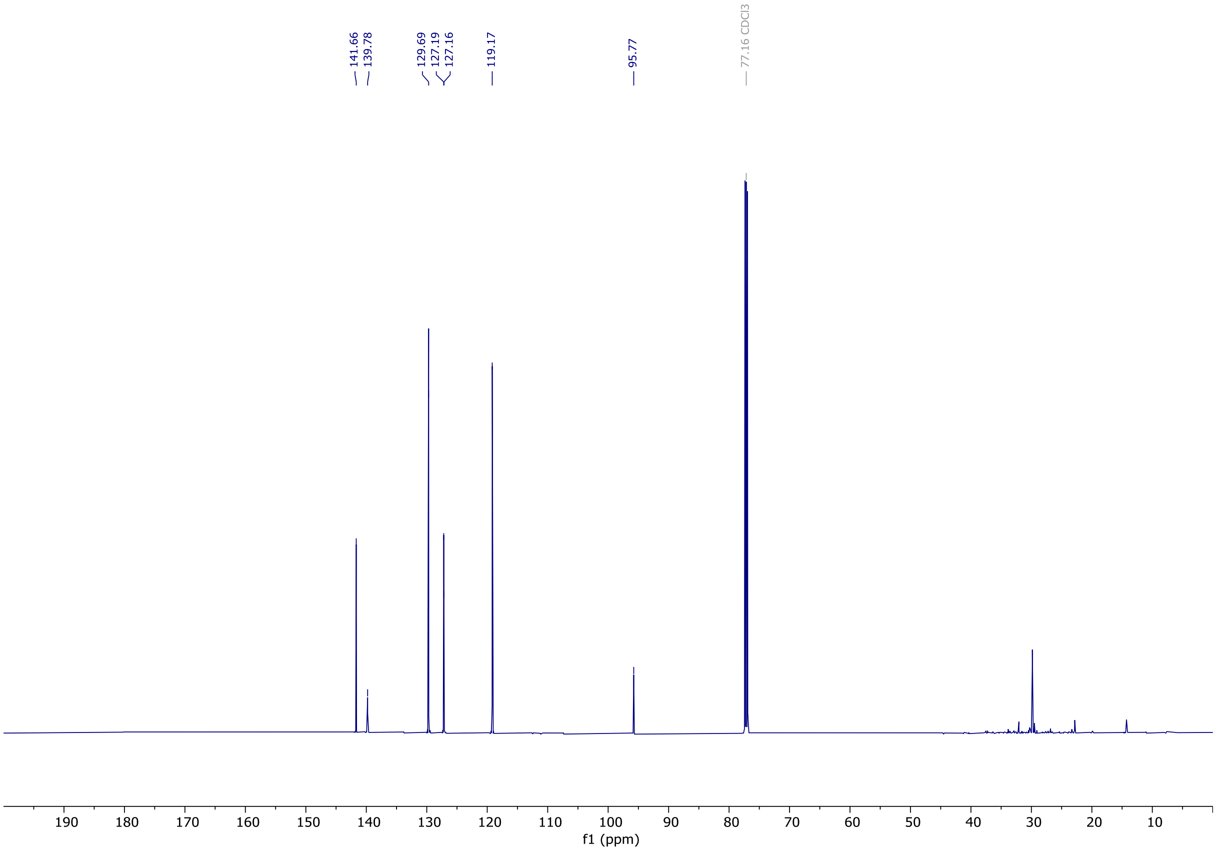

## 8b – $^{1}H$ NMR (600 MHz, $CDCl_3$)

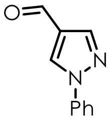

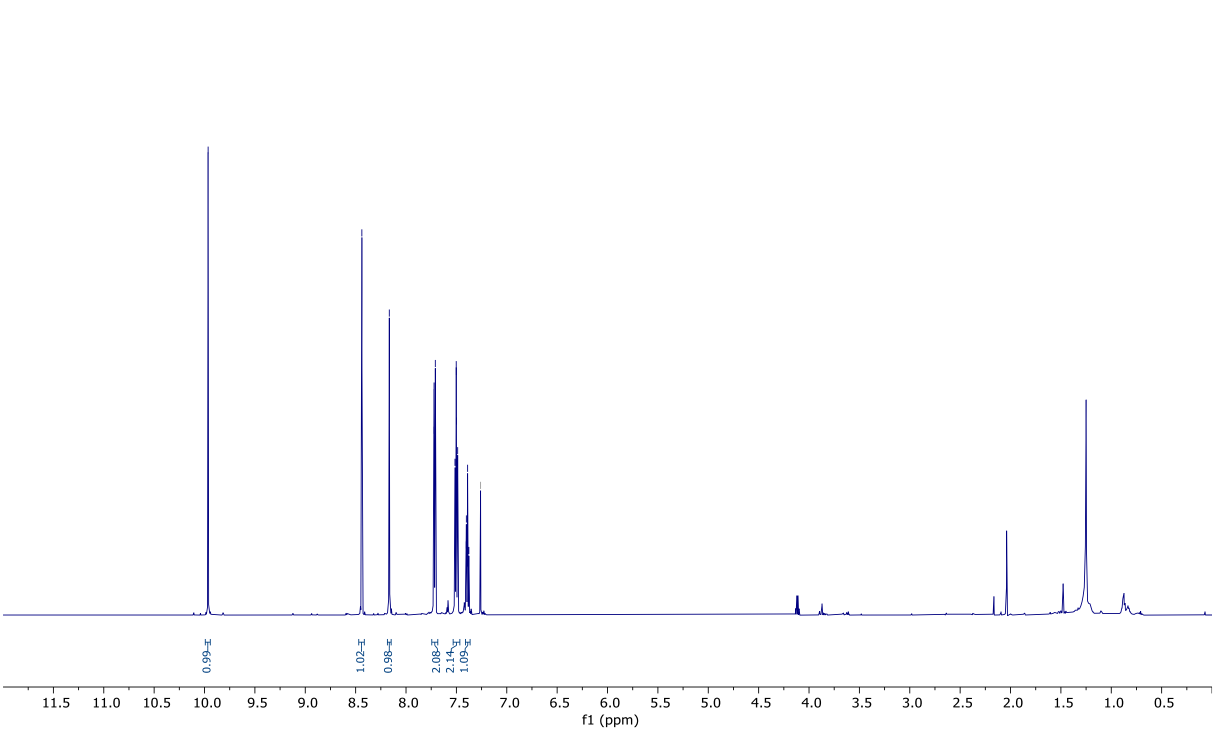


## 8b – $^{13}C$ NMR (151 MHz, $CDCl_3$)

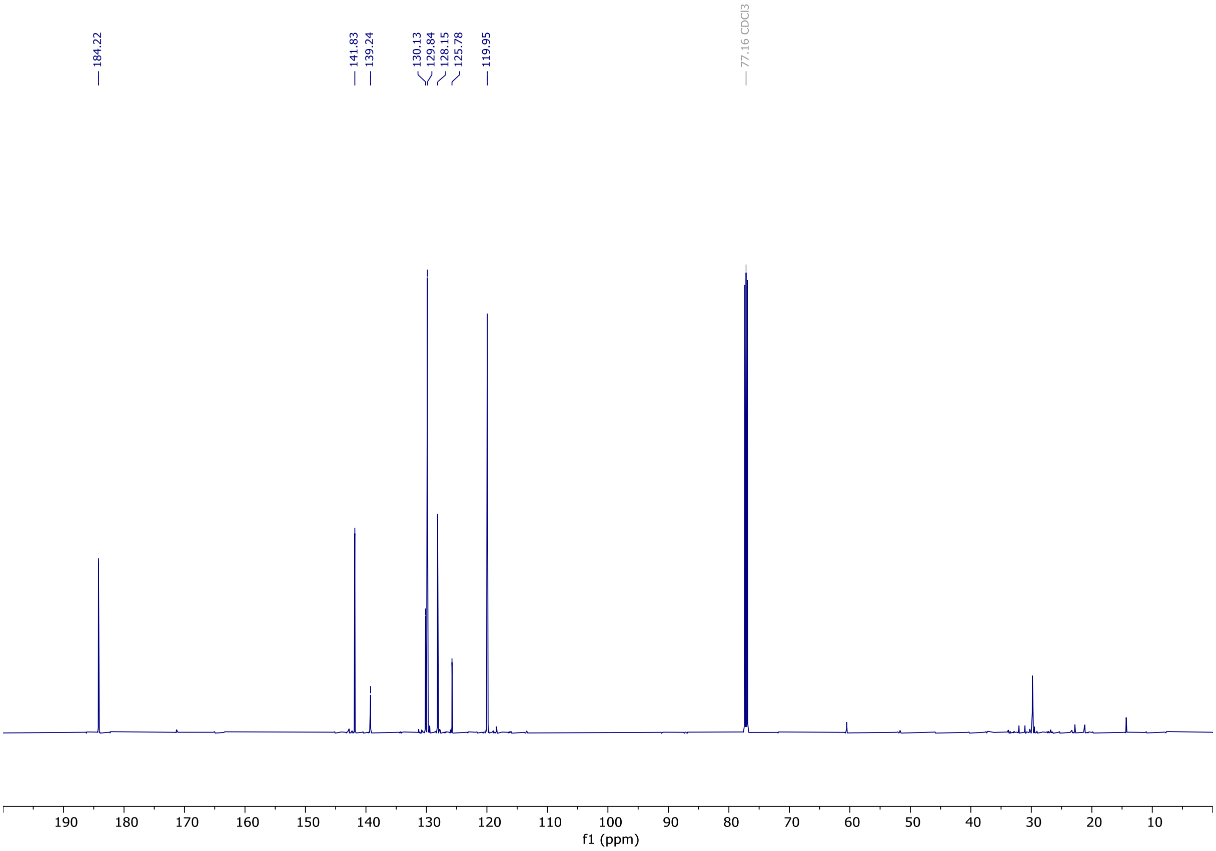

## 9b – $^{1}$H NMR (600 MHz, $CDCl_3$)

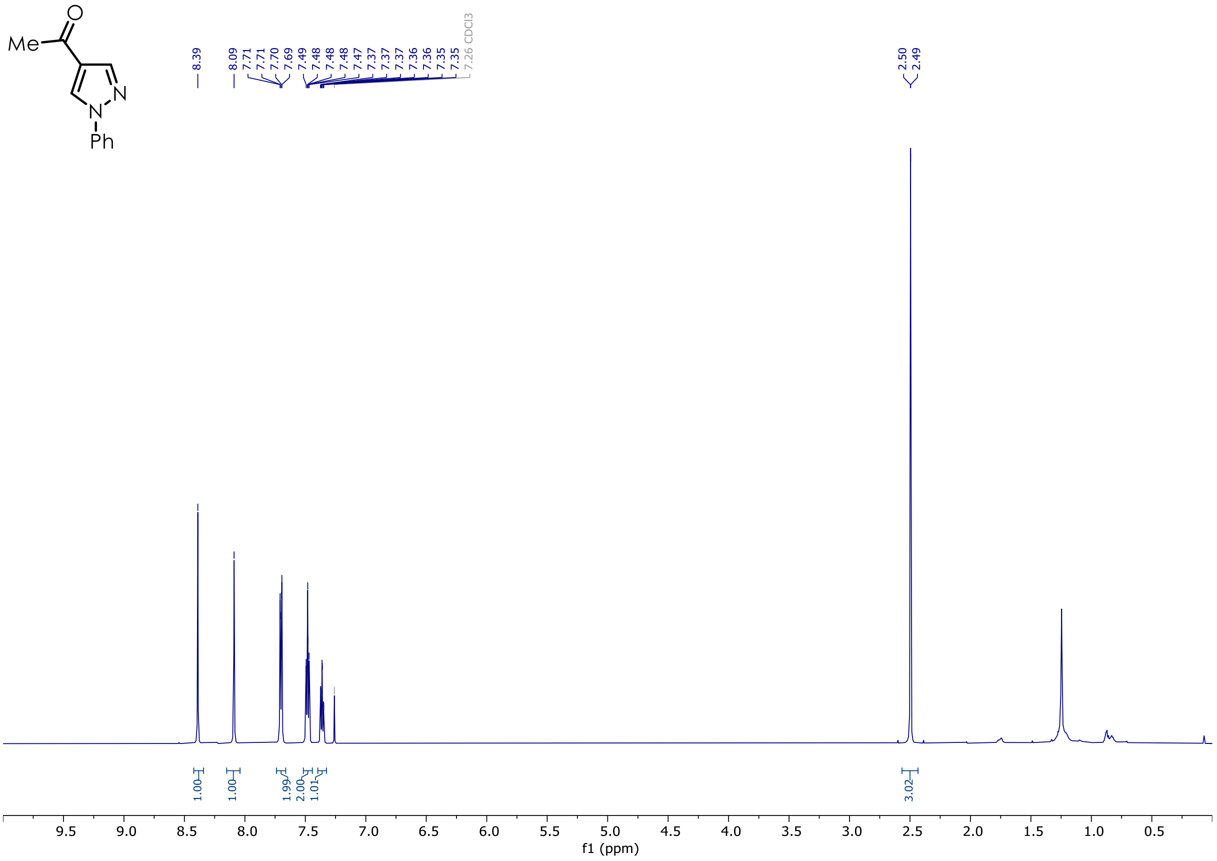


## 9b – $^{13}$C NMR (151 MHz, $CDCl_3$)

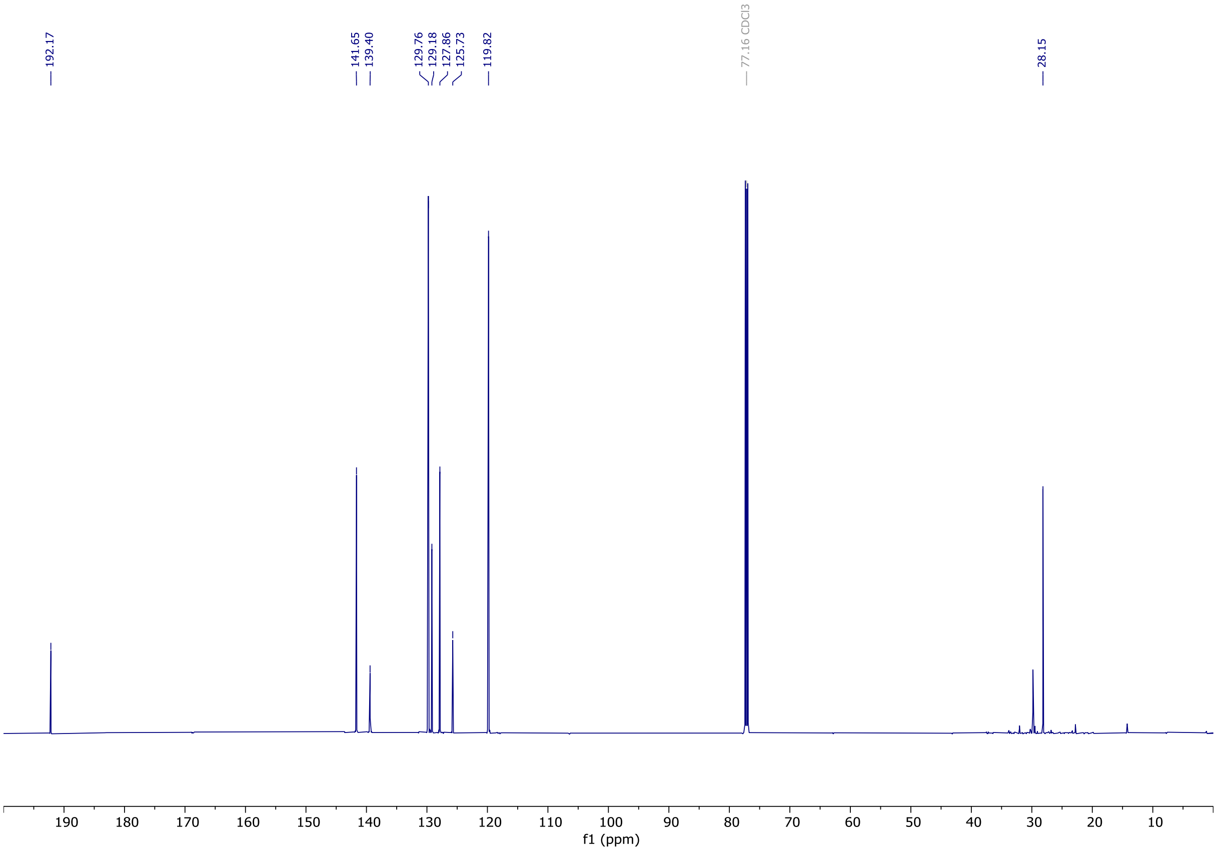

## 10b – $^1$H NMR (600 MHz, $CDCl_3$)

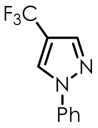


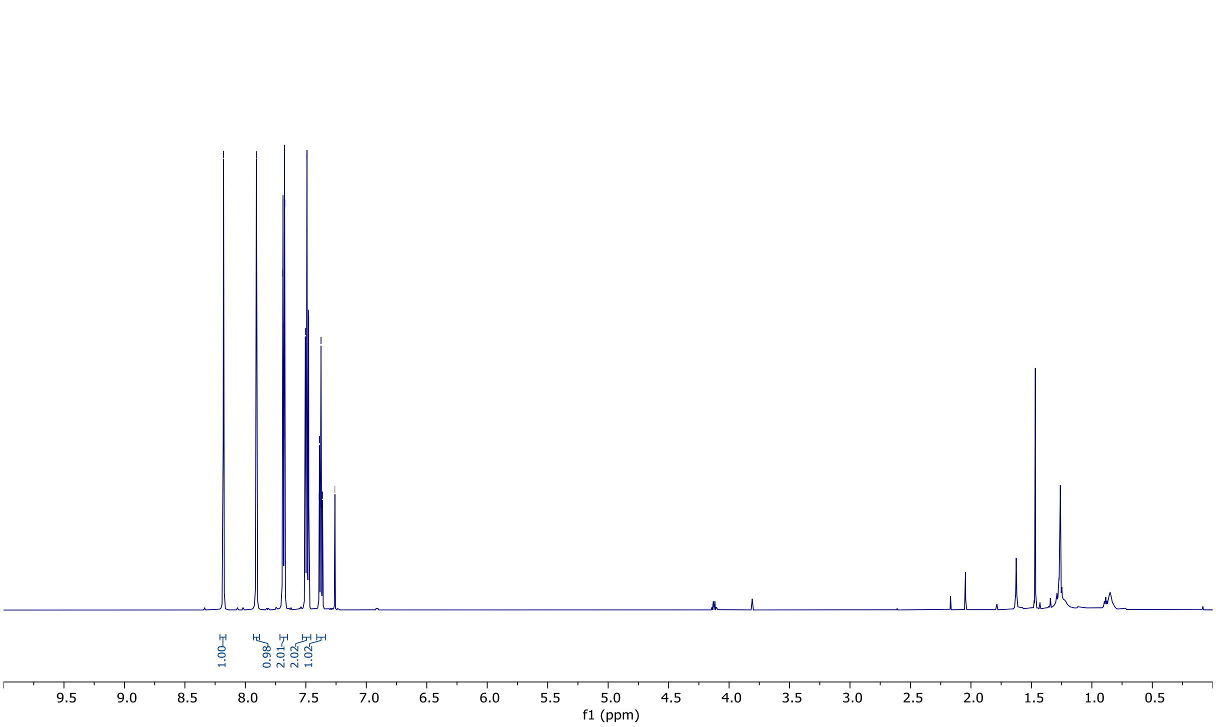


## 10b – $^{13}$C NMR (151 MHz, $CDCl_3$)

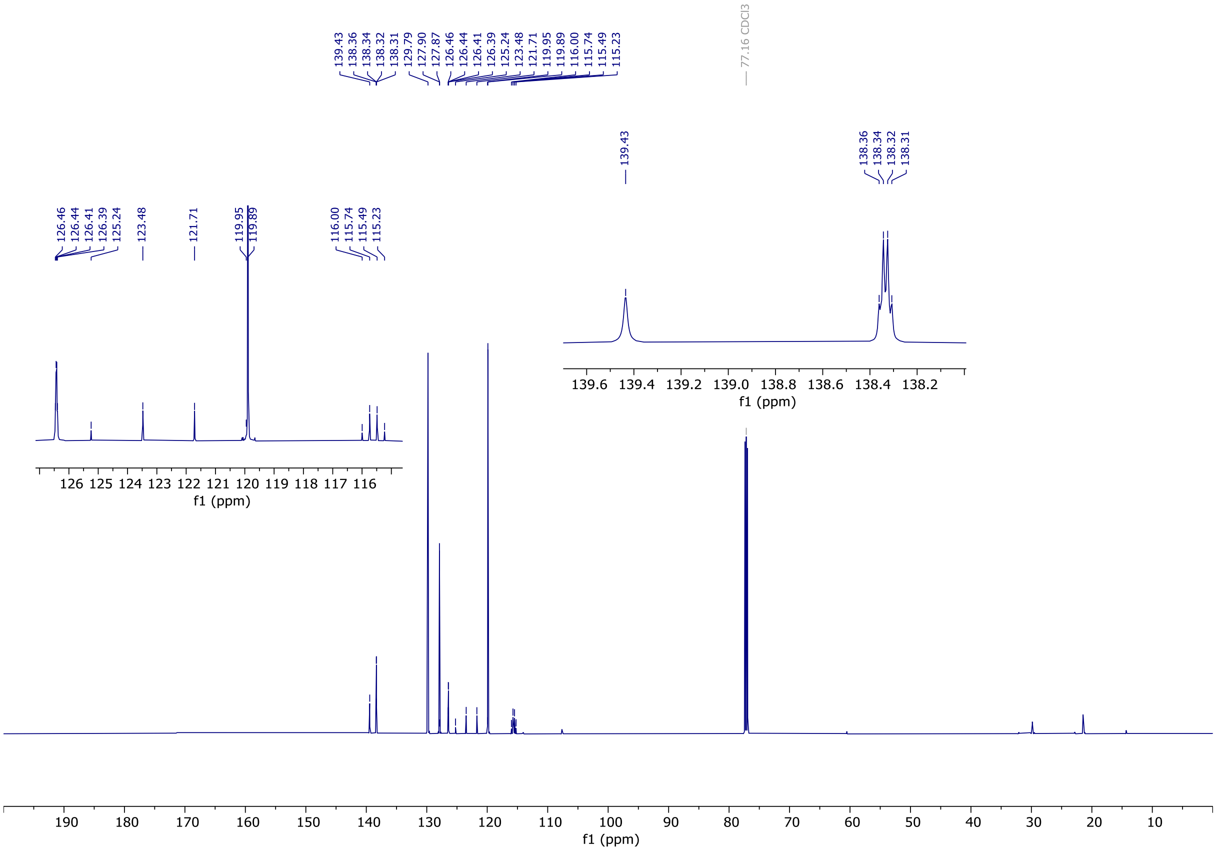

**10b – $^{19}$F NMR (565 MHz, $CDCl_3$)**

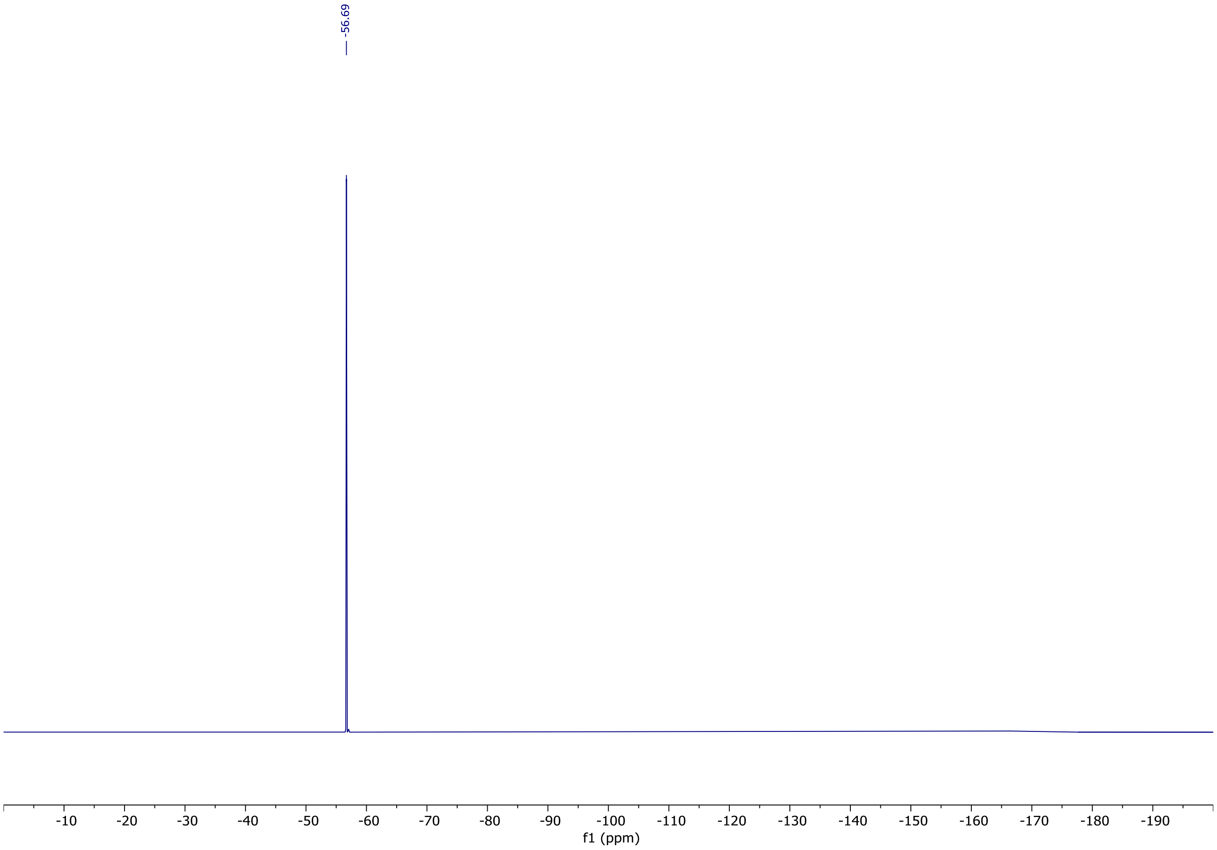

## 11b – $^{1}$H NMR (600 MHz, $CDCl_3$)

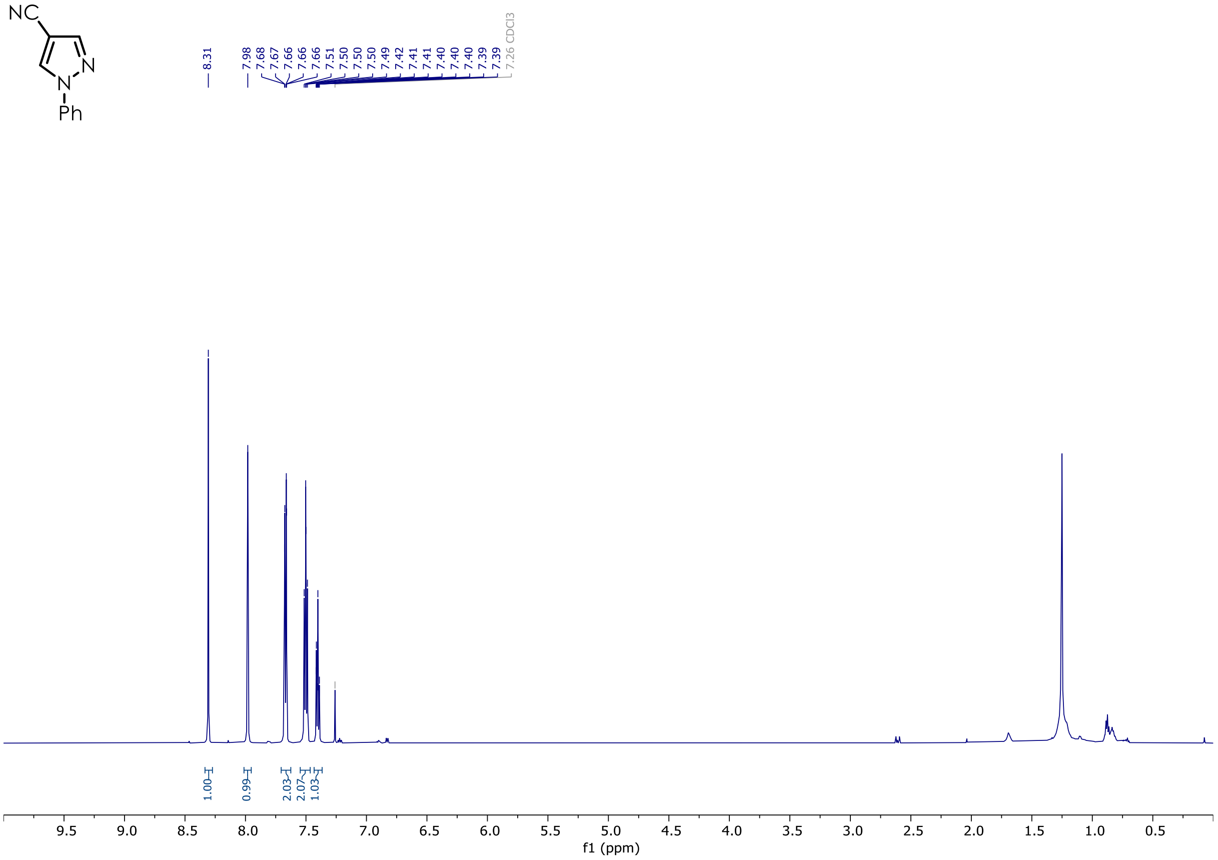


## 11b – $^{13}$C NMR (151 MHz, $CDCl_3$)

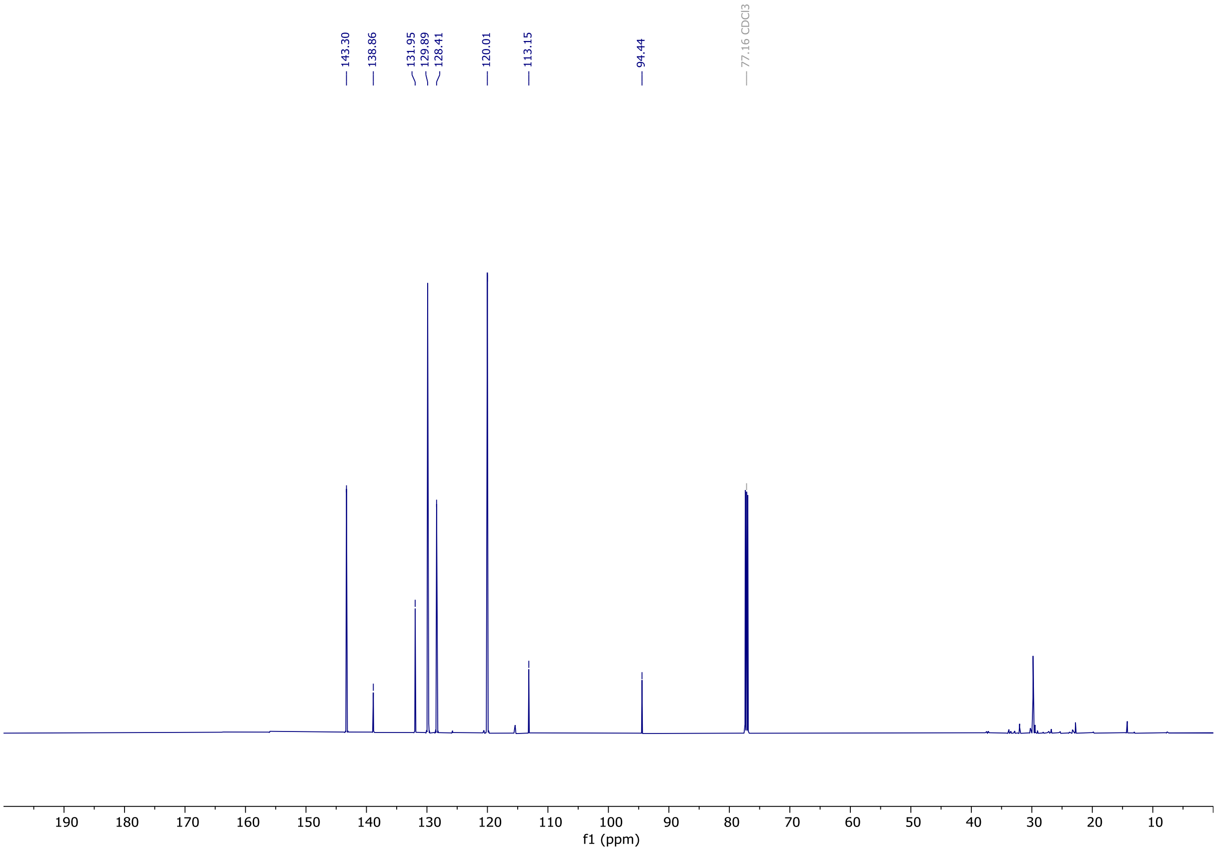

## 12b – $^{1}$H NMR (600 MHz, $CDCl_3$)

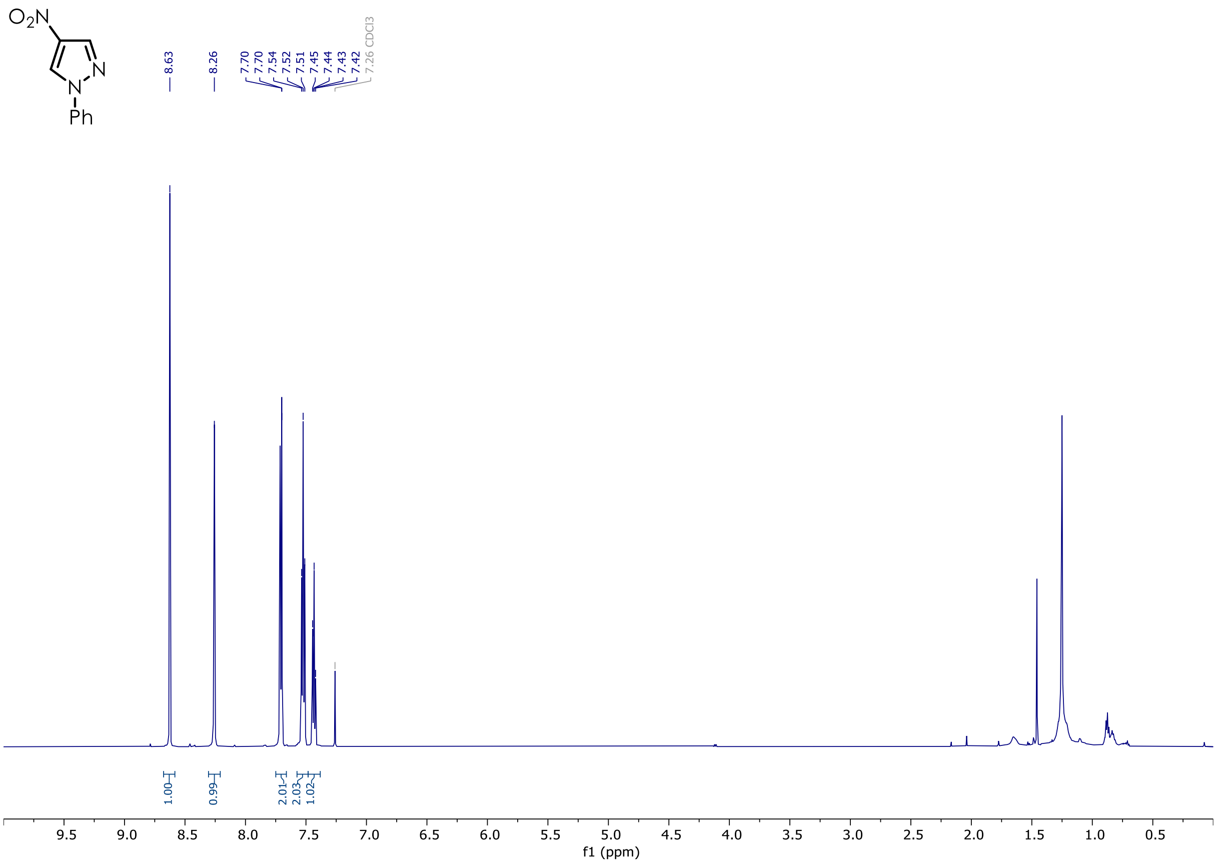


## 12b – $^{13}$C NMR (151 MHz, $CDCl_3$)

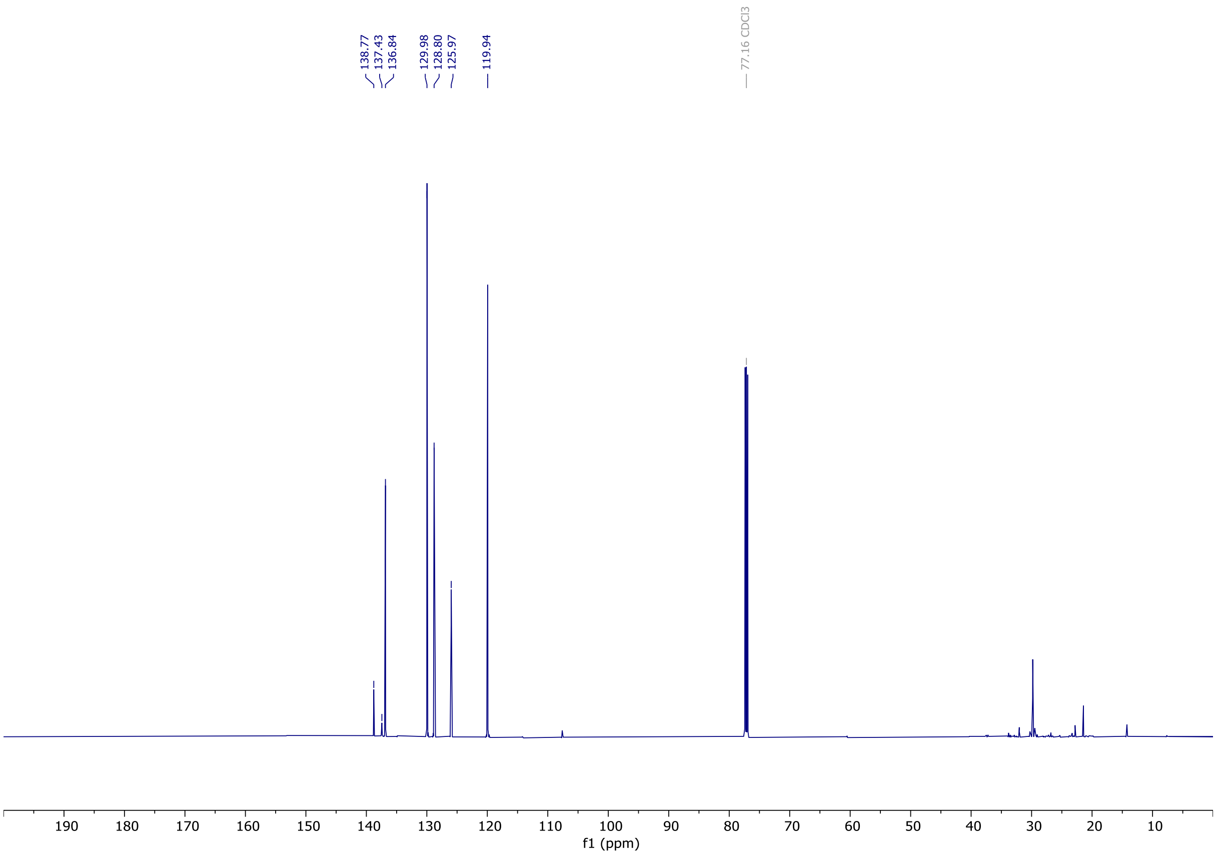

## 13b – $^{1}$H NMR (600 MHz, $CDCl_3$)

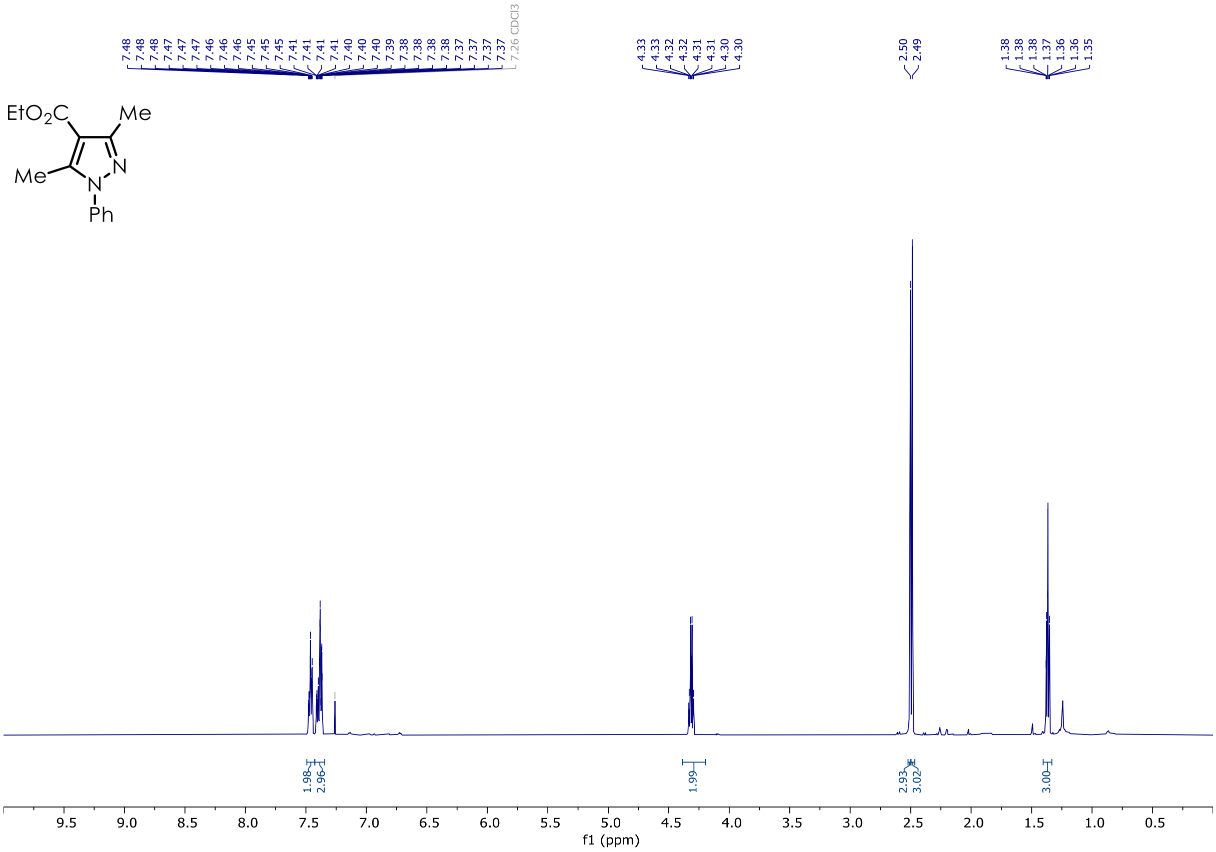


## 13b – $^{13}$C NMR (151 MHz, $CDCl_3$)

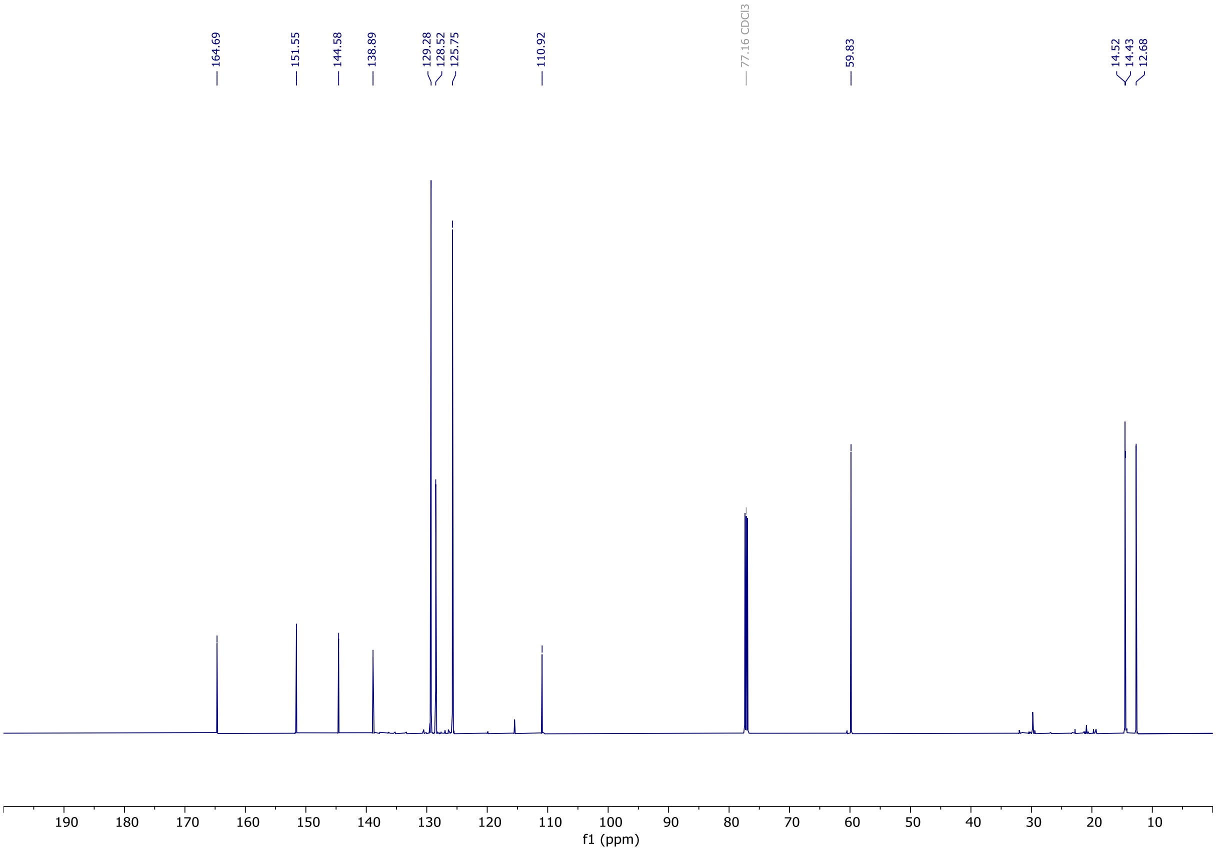

## 14b – $^{1}$H NMR (600 MHz, $CDCl_3$)

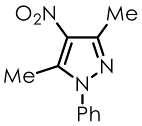

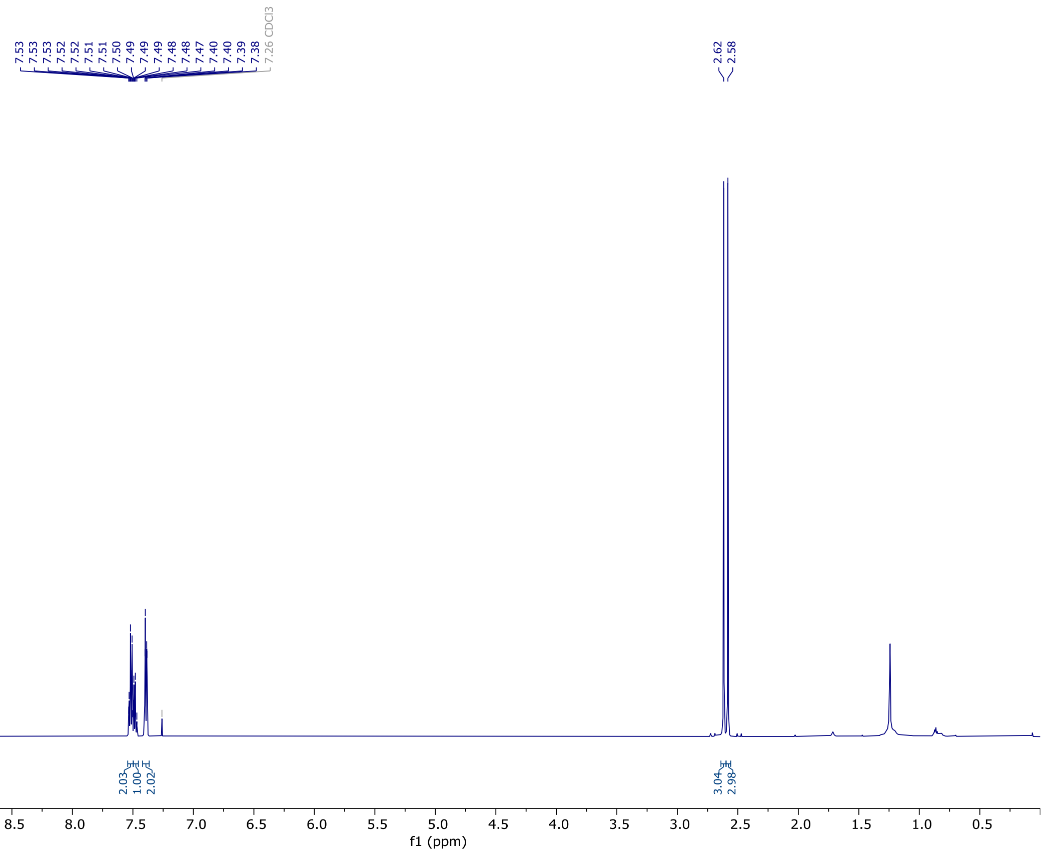

## 14b – $^{13}$C NMR (151 MHz, $CDCl_3$)

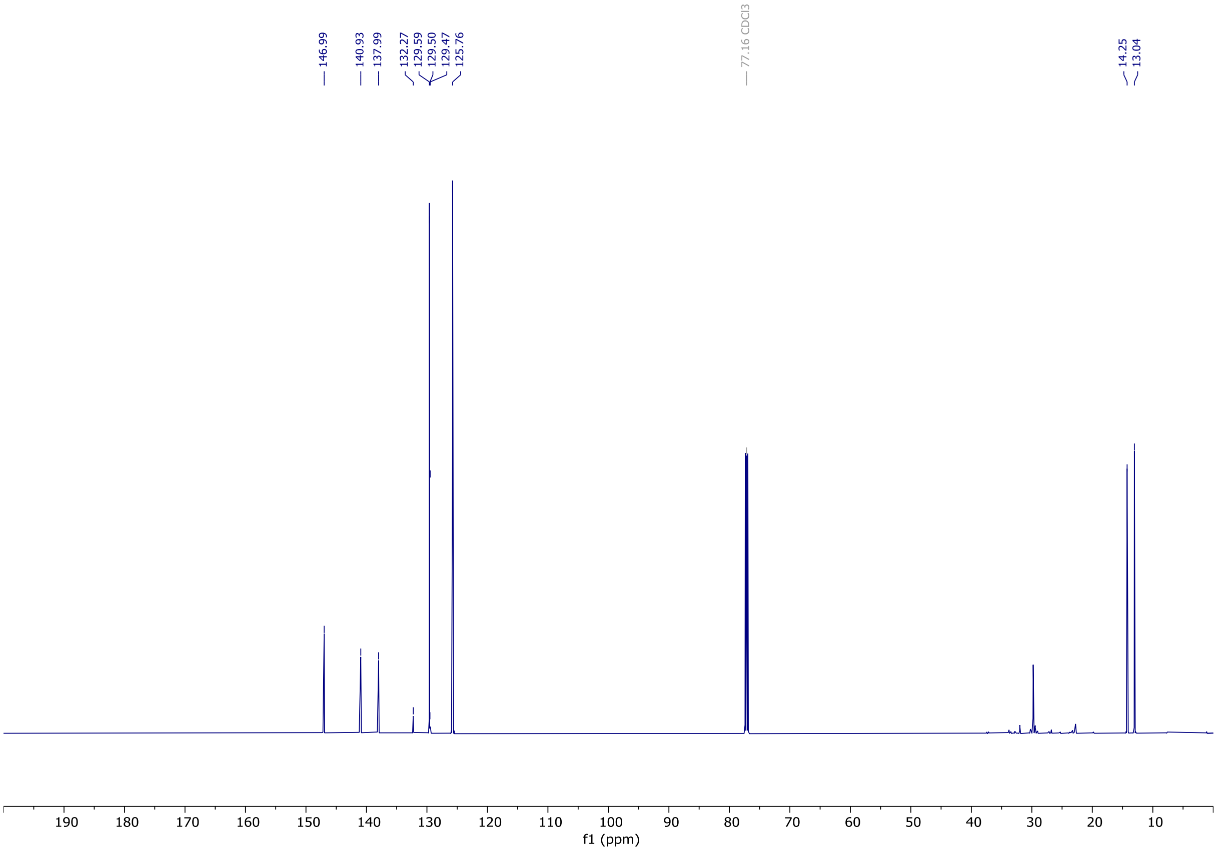

**15b – $^{1}$H NMR (600 MHz, $CDCl_3$)**

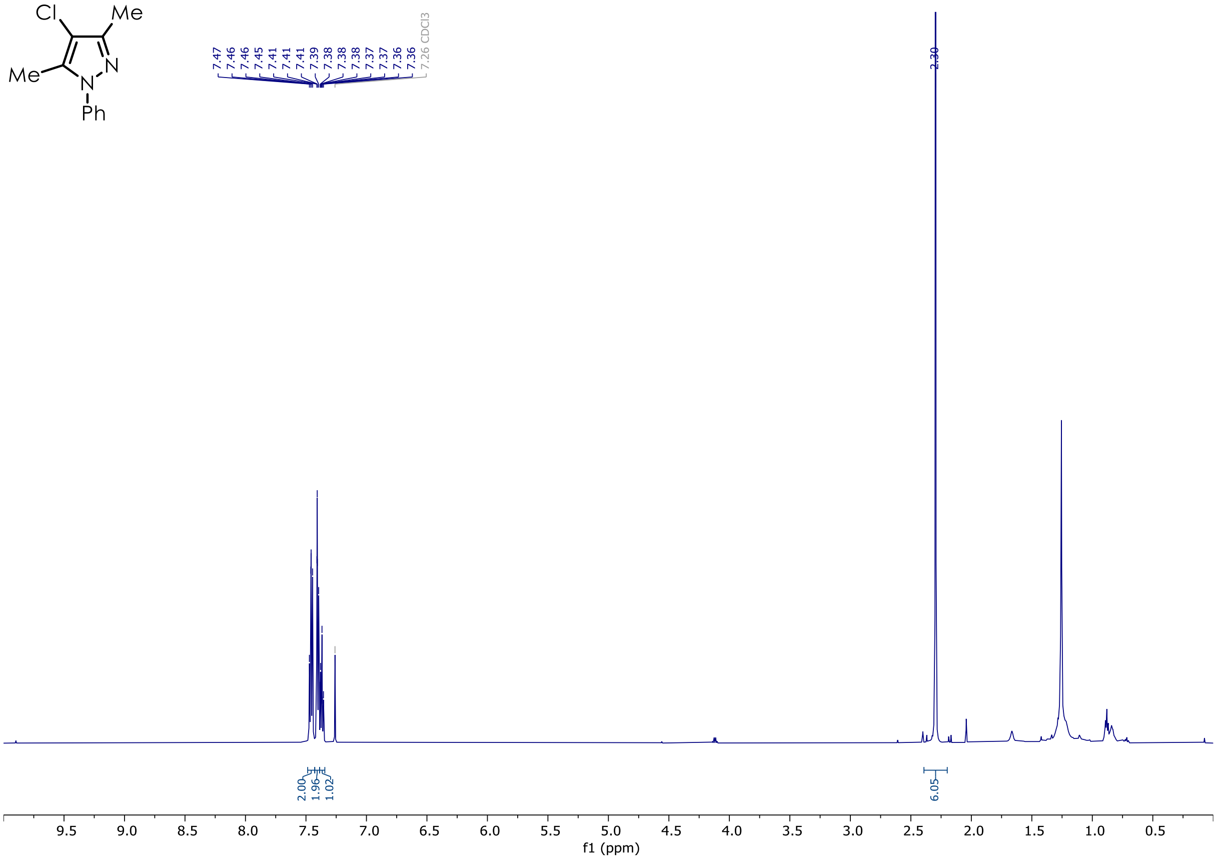


**15b – $^{13}$C NMR (151 MHz, $CDCl_3$)**

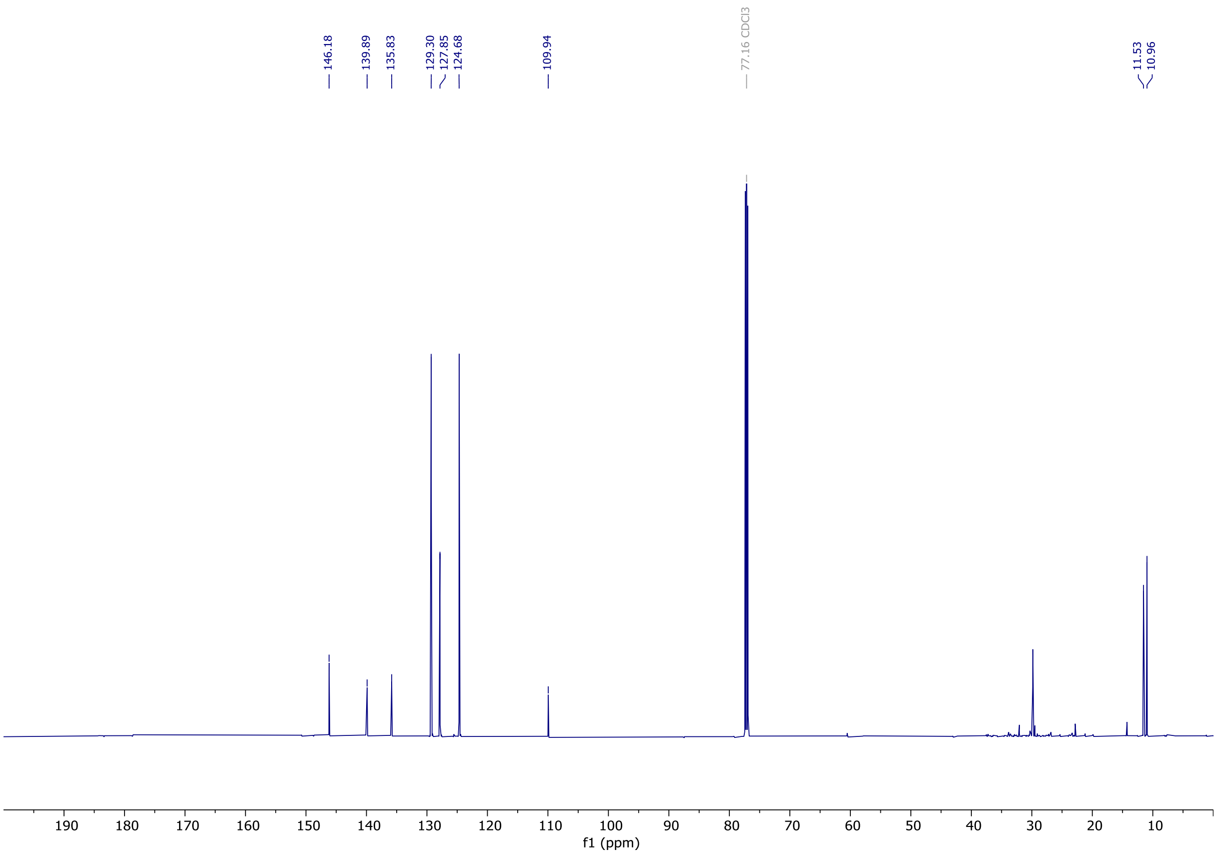

## 16b – $^{1}$H NMR (600 MHz, $CDCl_3$)

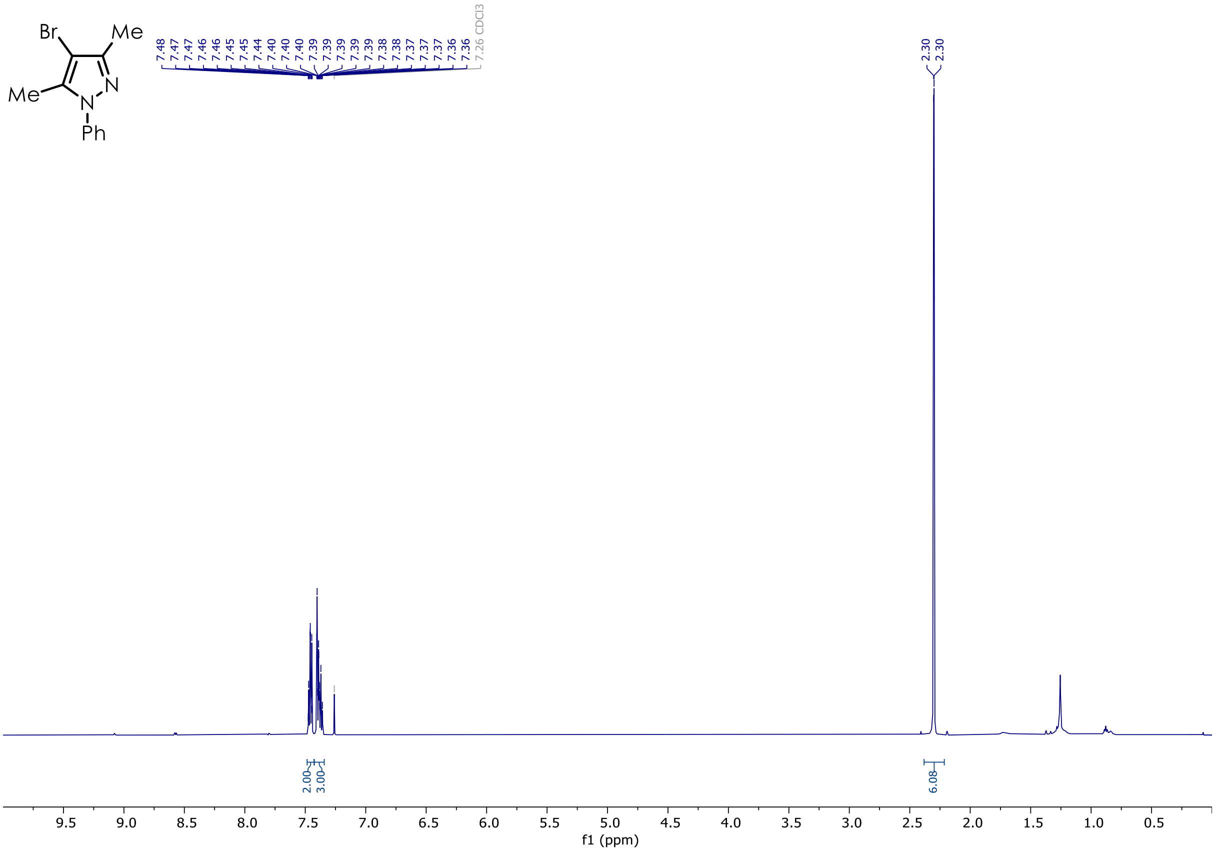


## 16b – $^{13}$C NMR (151 MHz, $CDCl_3$)

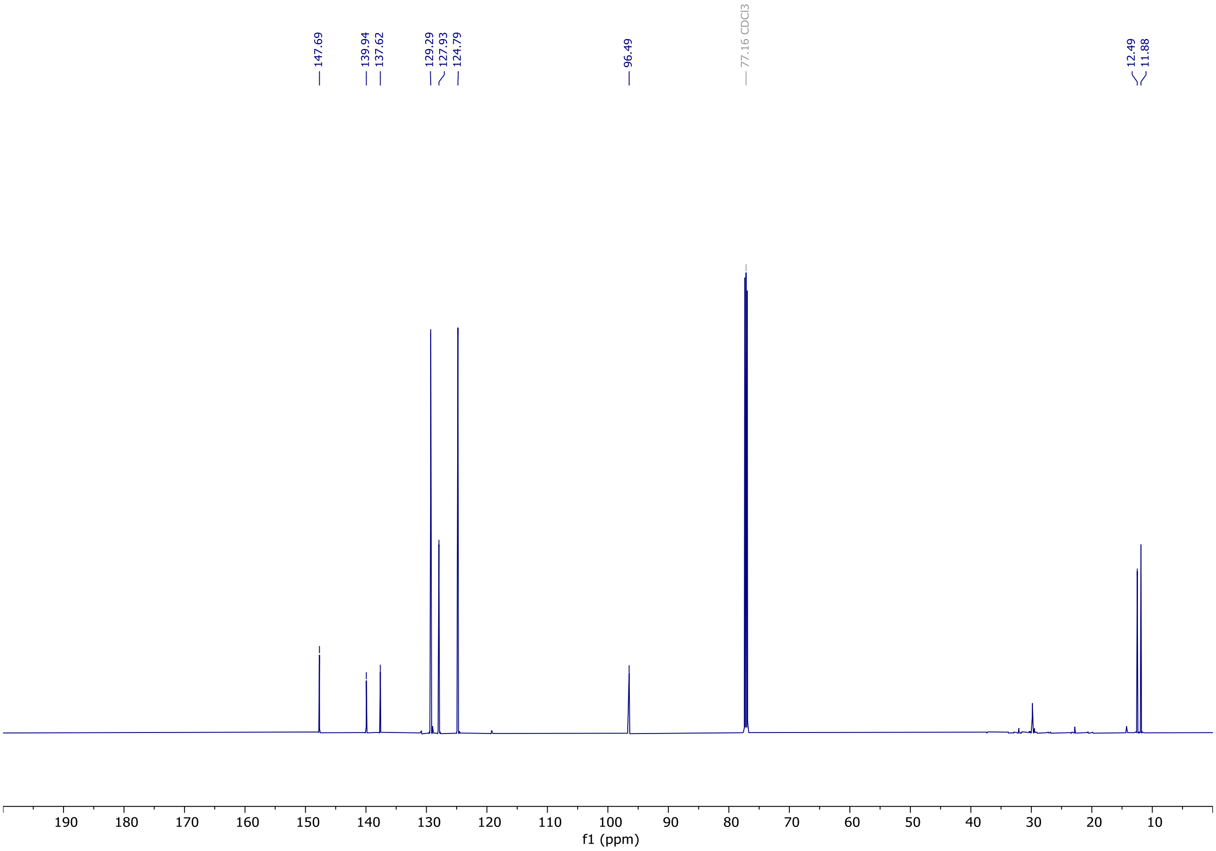

## 17b – $^{1}$H NMR (600 MHz, $CDCl_3$)

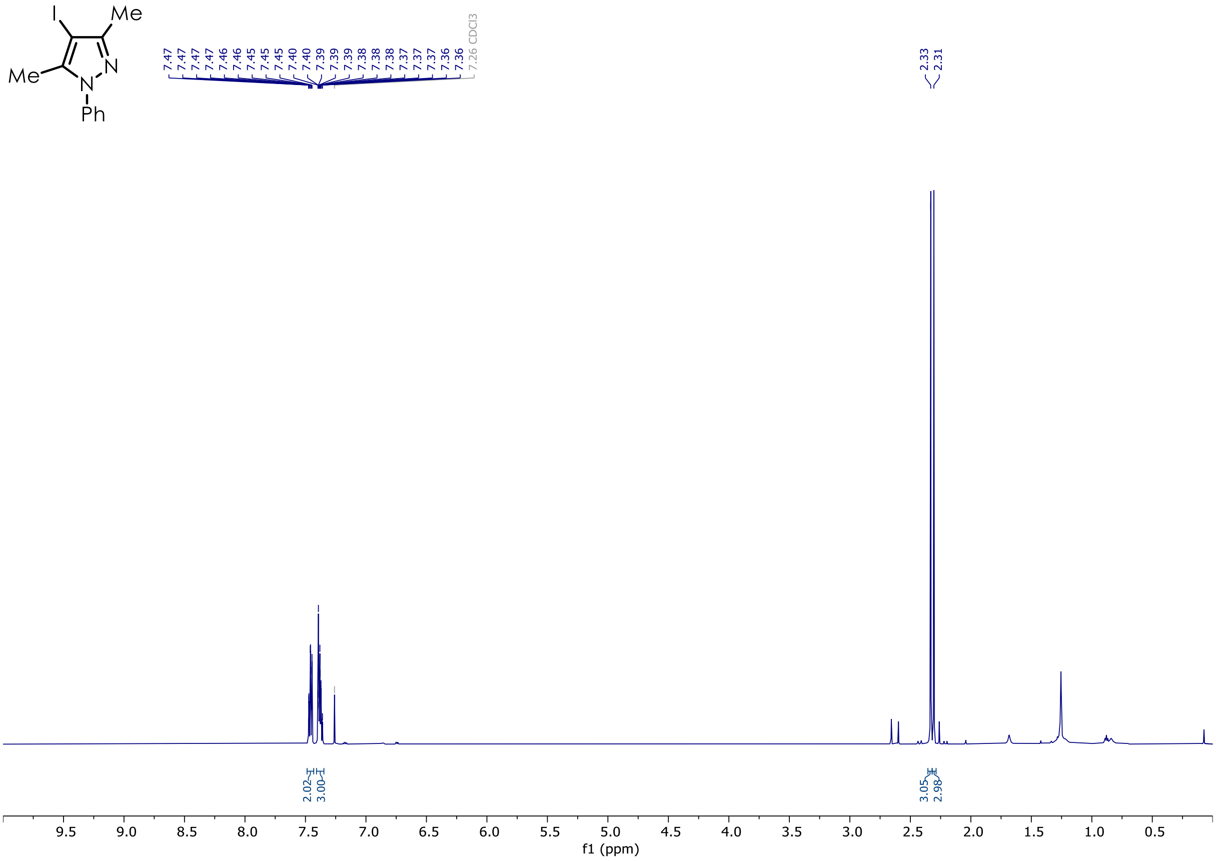


## 17b – $^{13}$C NMR (151 MHz, $CDCl_3$)

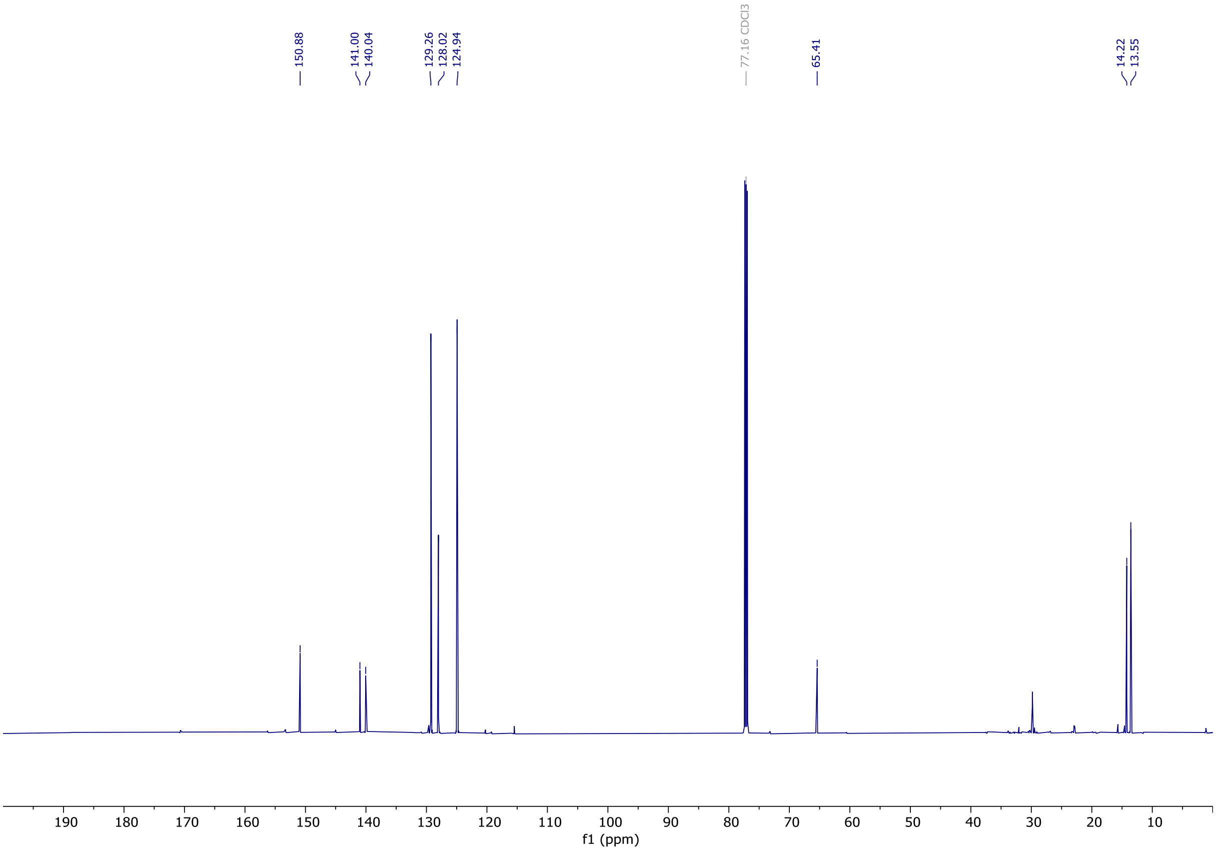

## 18b – $^{1}$H NMR (600 MHz, CDCl$_3$)

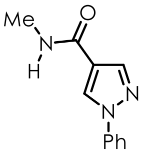


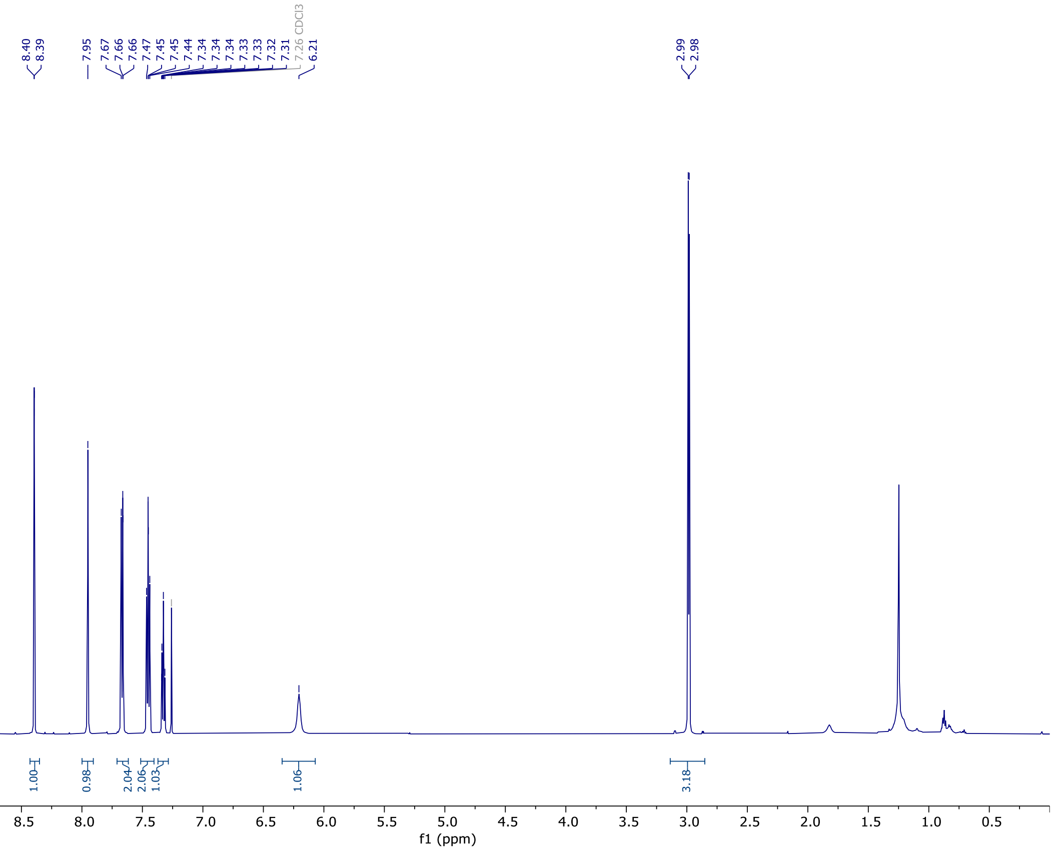


## 18b – $^{13}$C NMR (151 MHz, CDCl$_3$)

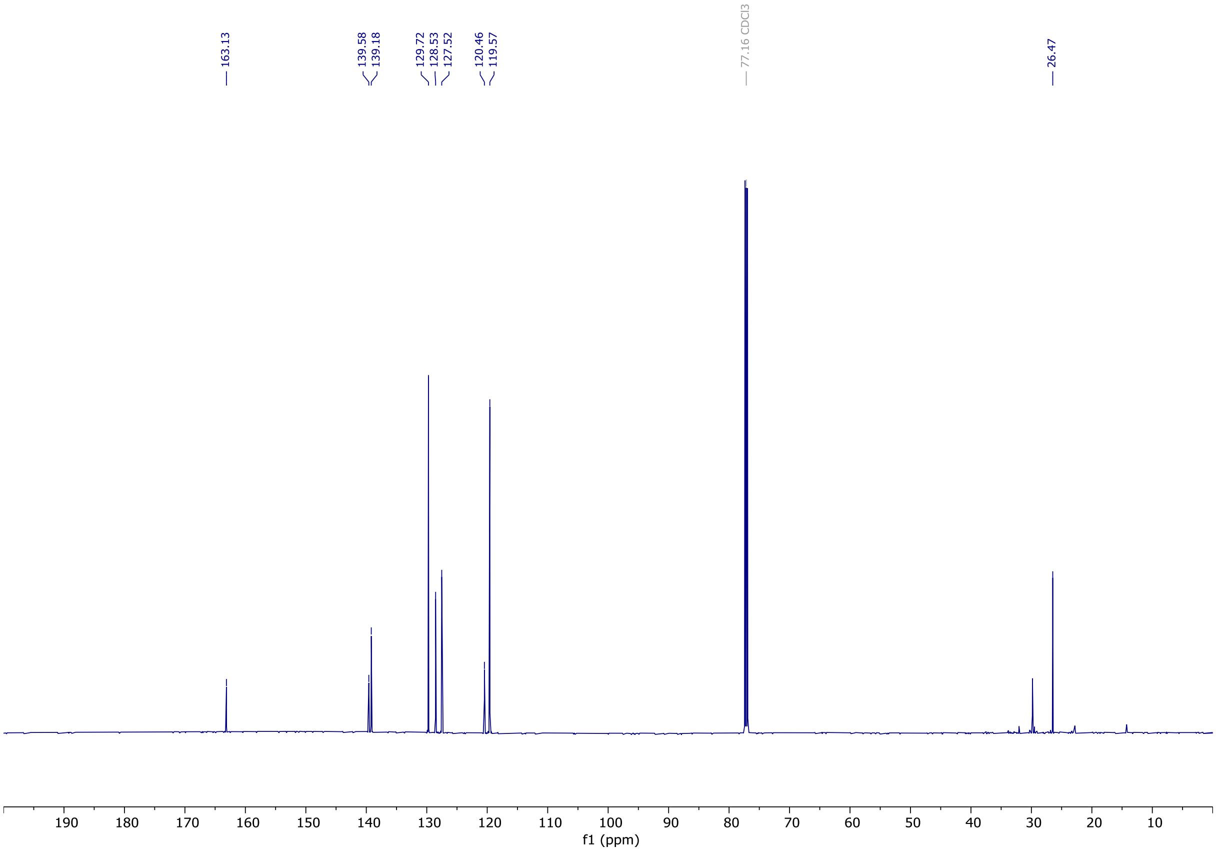

## 19b – $^{1}$H NMR (600 MHz, $CDCl_3$)

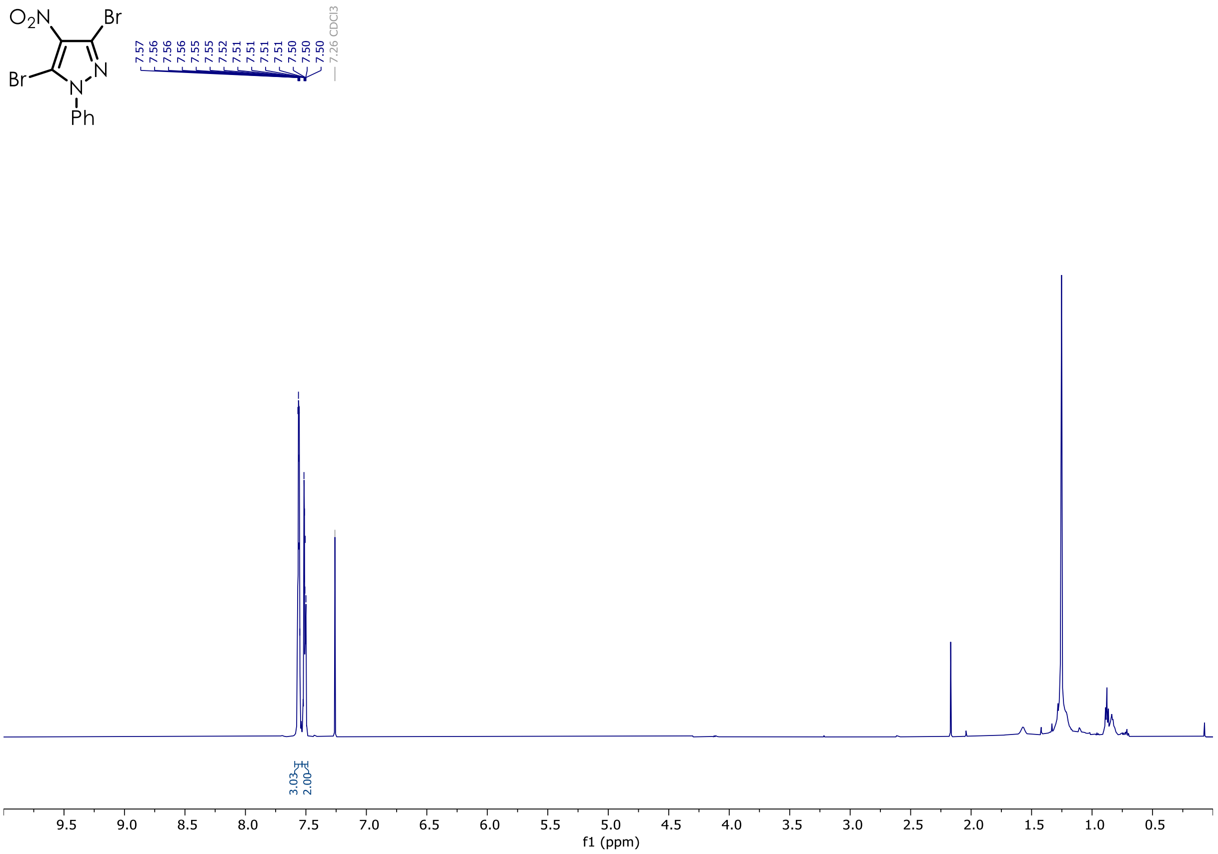


## 19b – $^{13}$C NMR (151 MHz, $CDCl_3$)

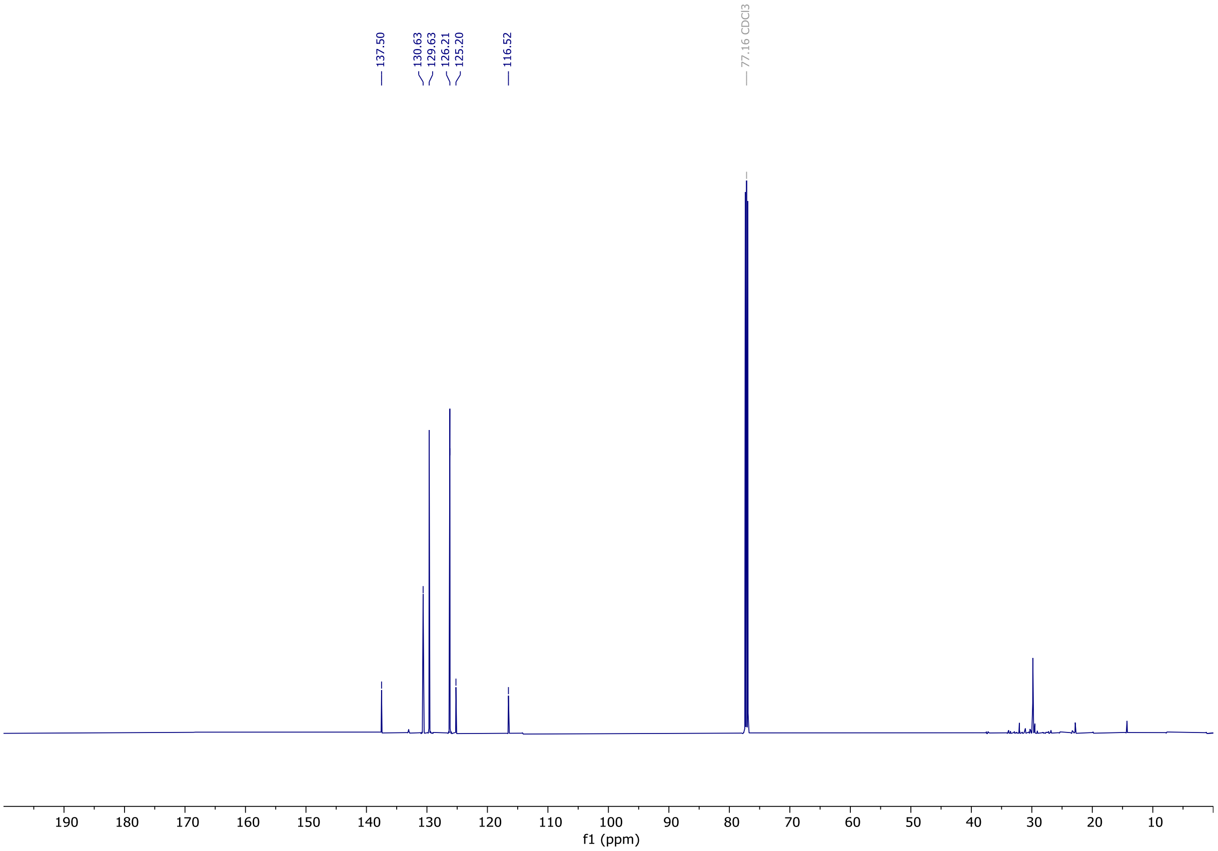

## 20b – $^{1}H$ NMR (600 MHz, $CDCl_3$)

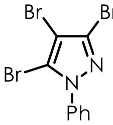


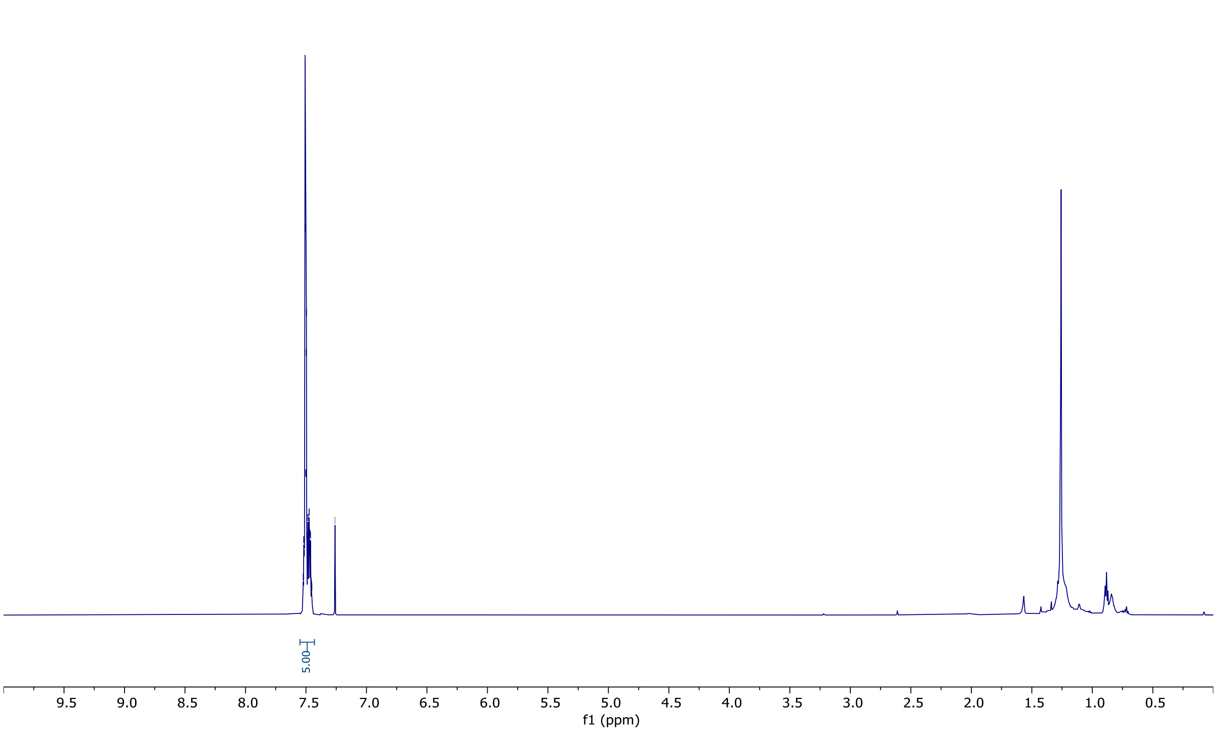


## 20b – $^{13}C$ NMR (151 MHz, $CDCl_3$)

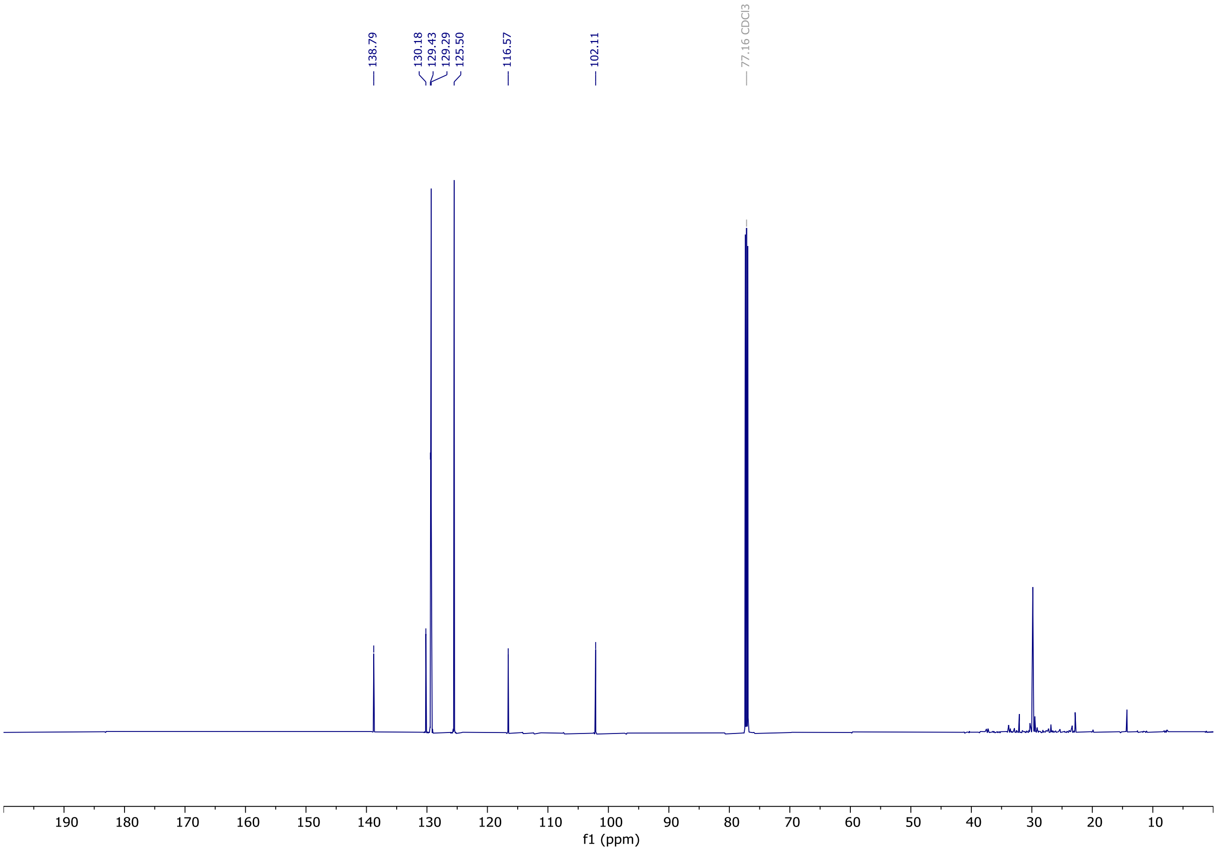

## 21b – $^{1}$H NMR (600 MHz, $CDCl_3$)

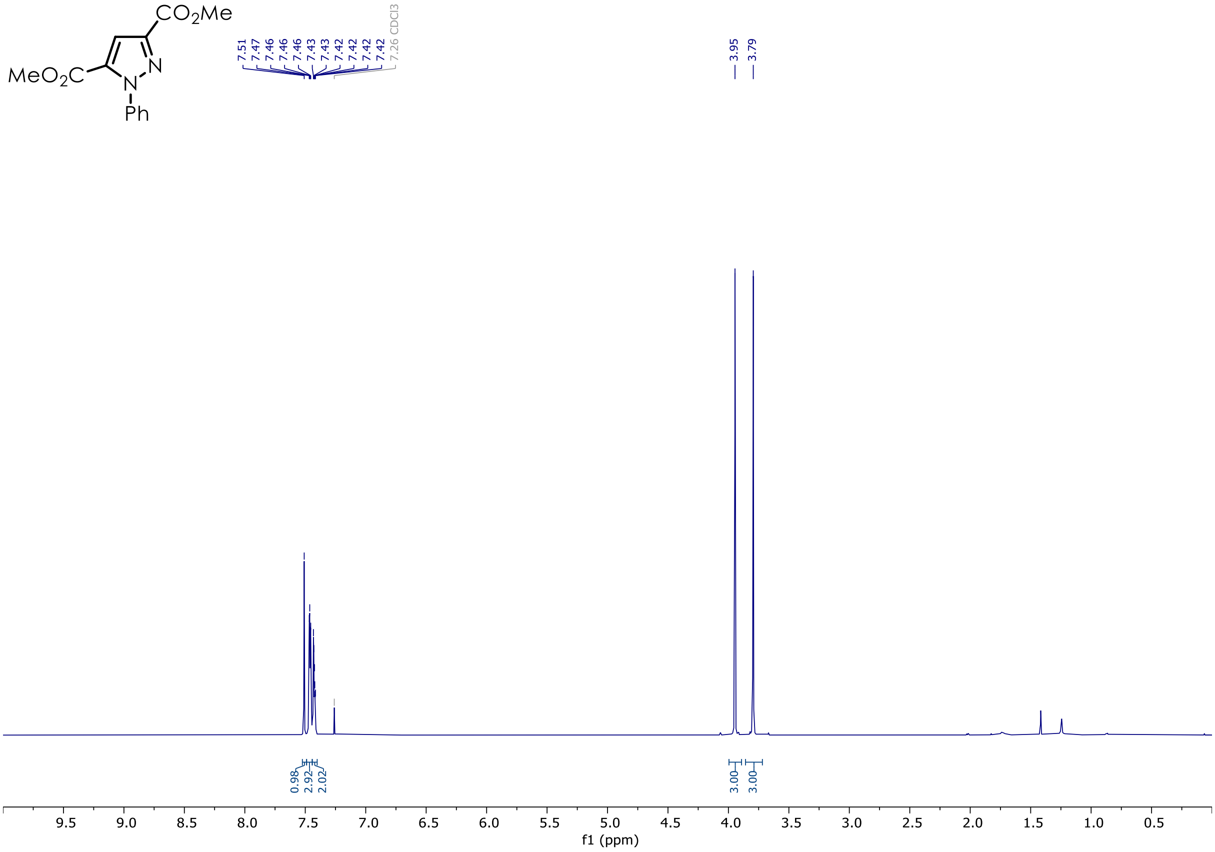


## 21b – $^{13}$C NMR (151 MHz, $CDCl_3$)

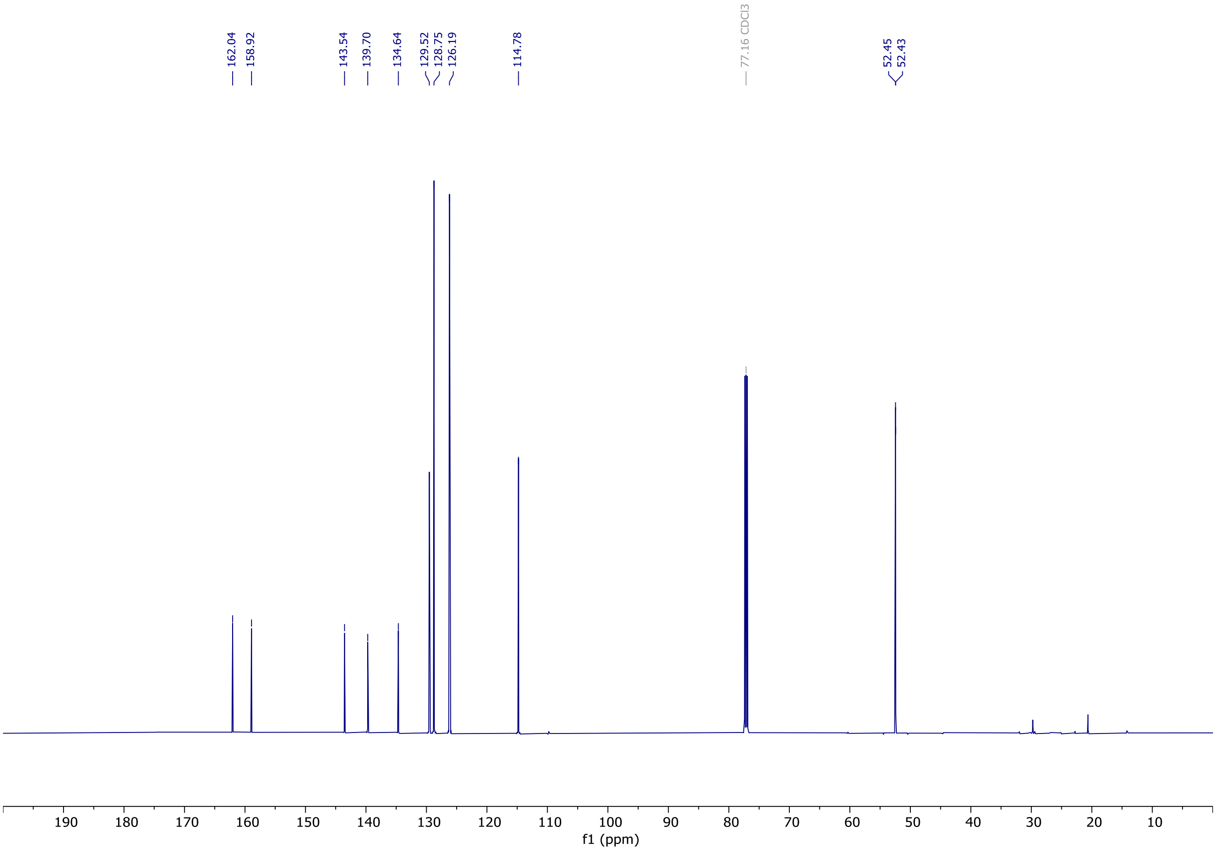

## 22b – $^{1}$H NMR (600 MHz, CDCl$_3$)

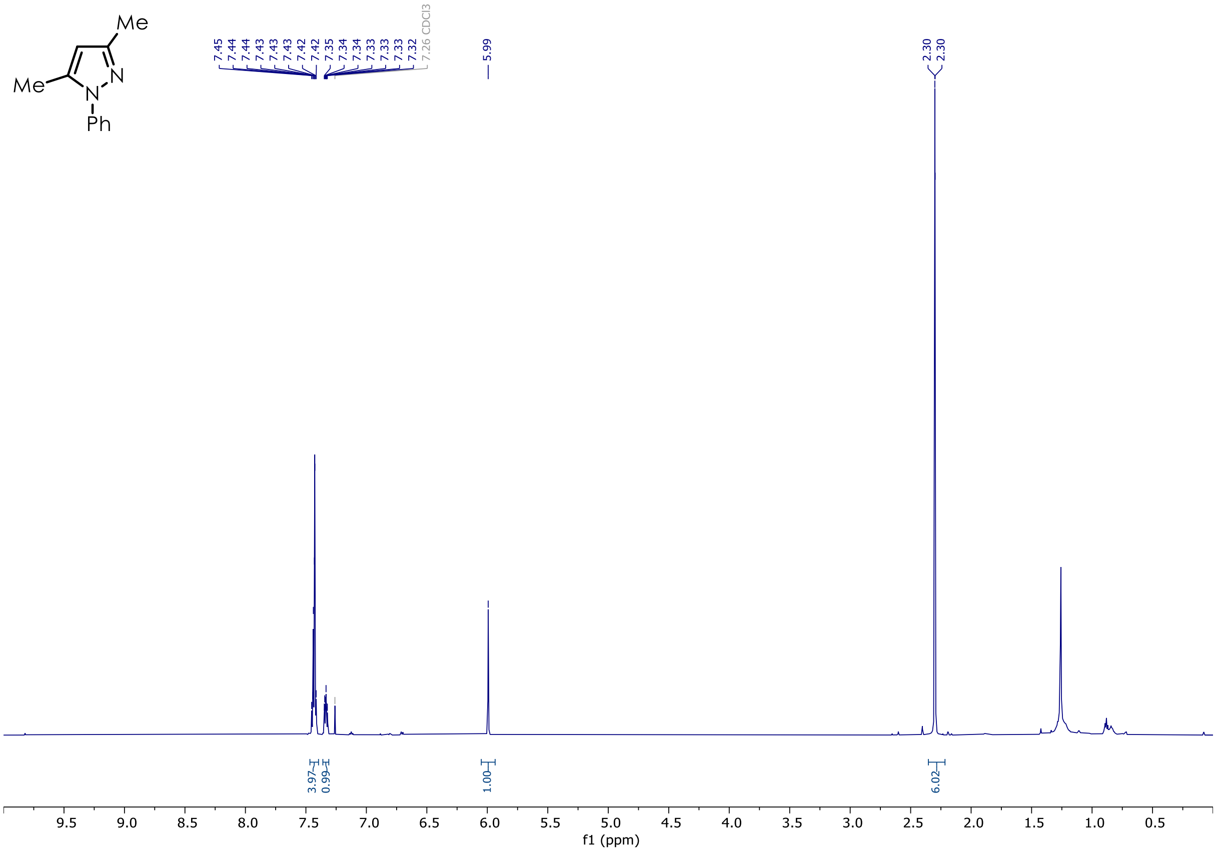


## 22b – $^{13}$C NMR (151 MHz, CDCl$_3$)

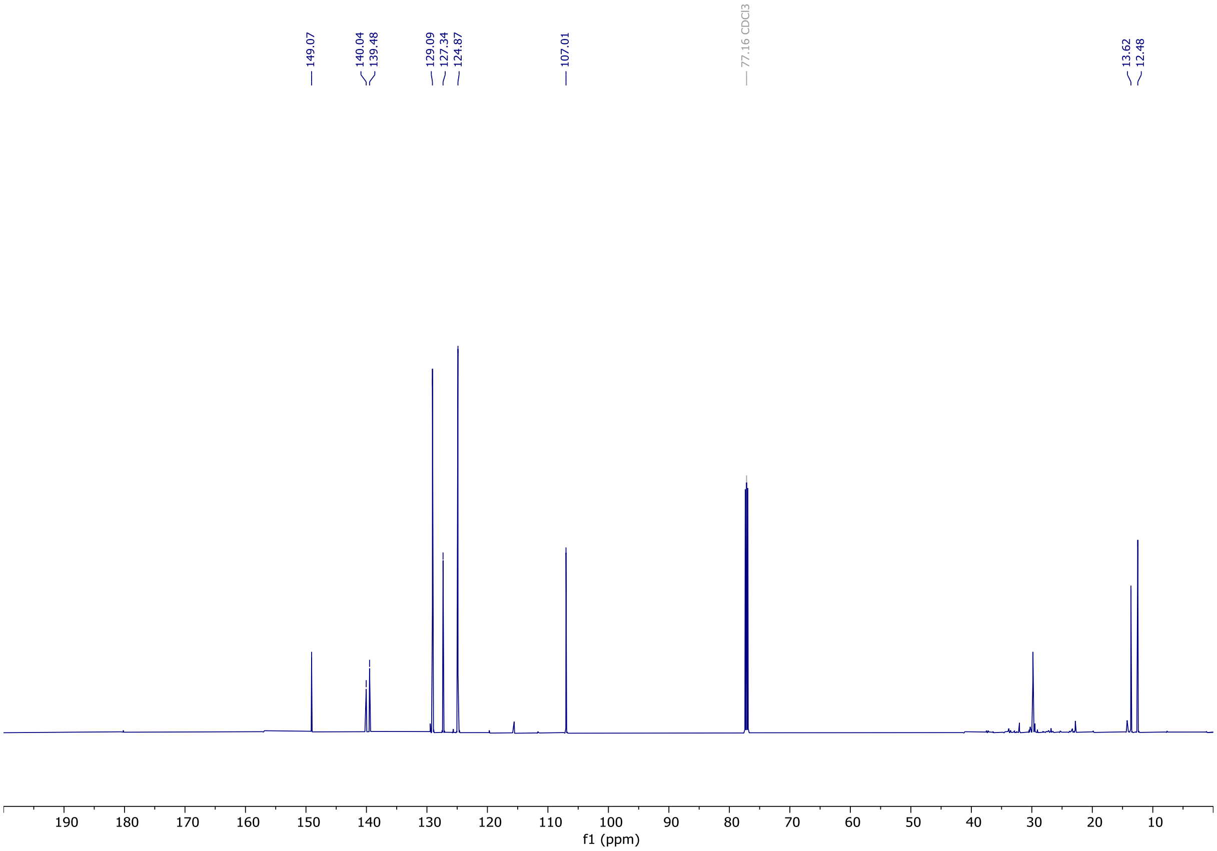

## 23b – $^{1}$H NMR (600 MHz, $CDCl_3$)

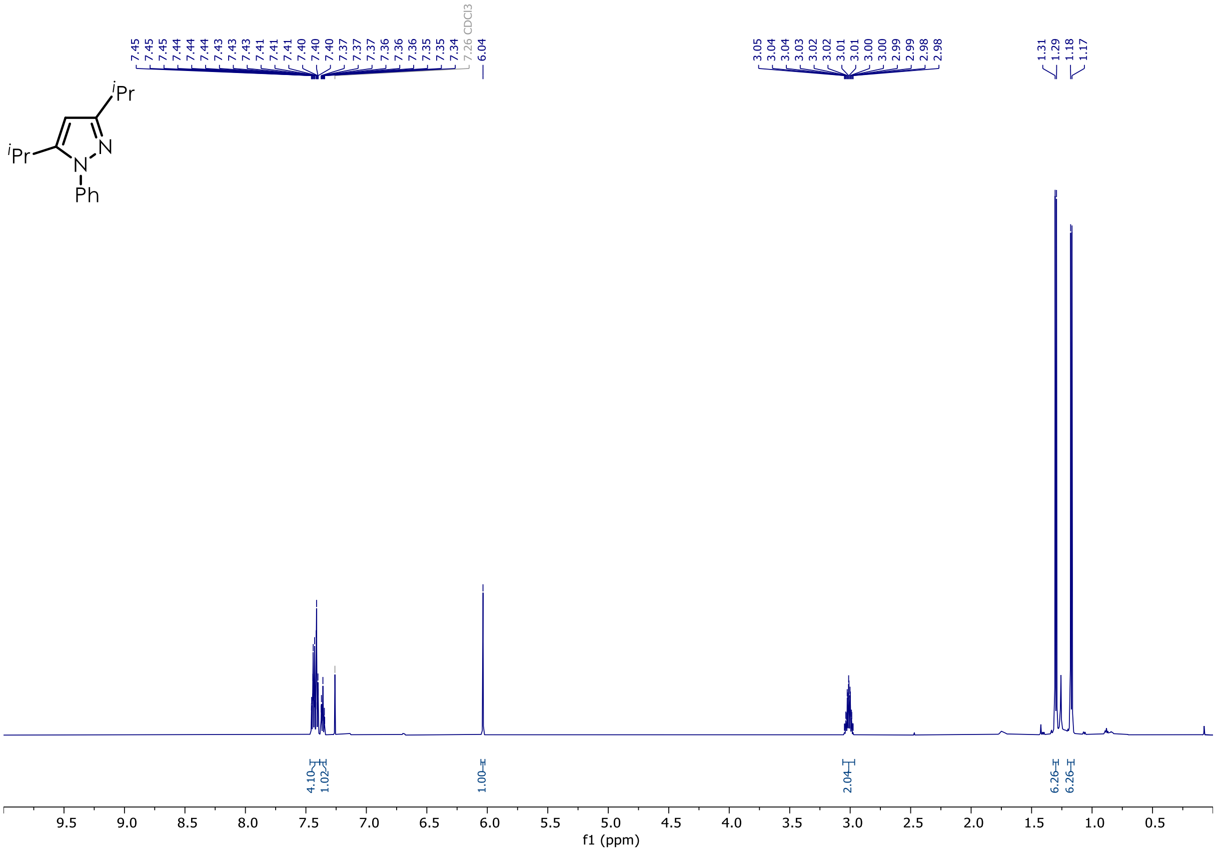


## 23b – $^{13}$C NMR (151 MHz, $CDCl_3$)

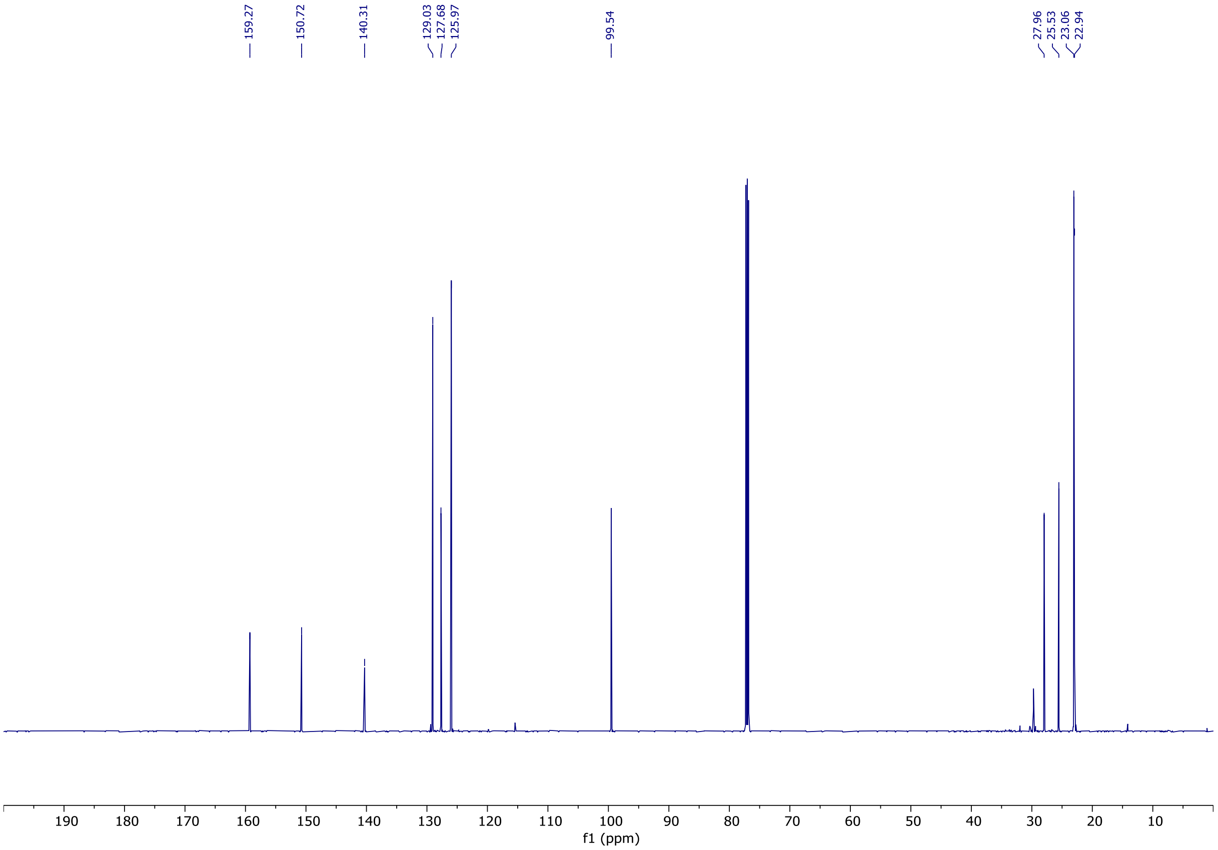

# 24b & 24b' – $^{1}$H NMR (600 MHz, $CDCl_3$)

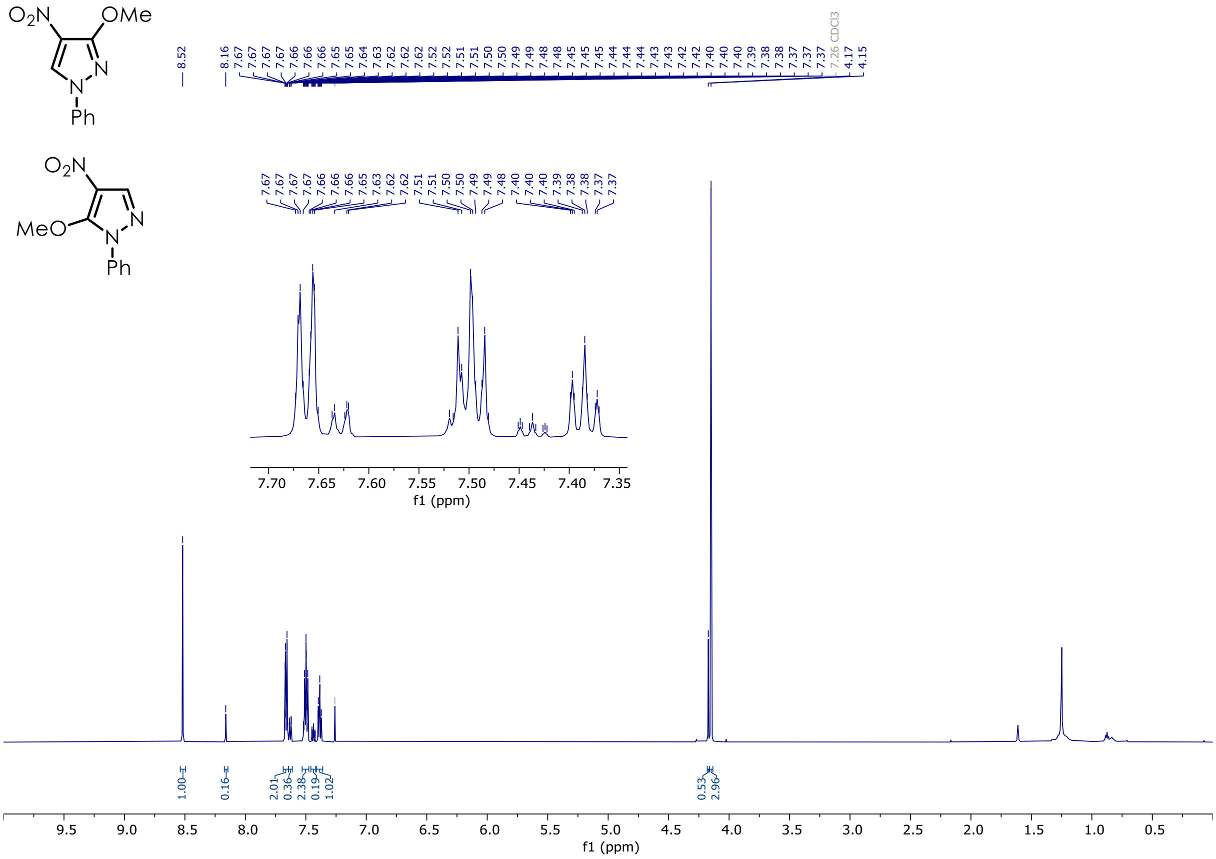


# 24b & 24b' – $^{13}$C NMR (151 MHz, $CDCl_3$)

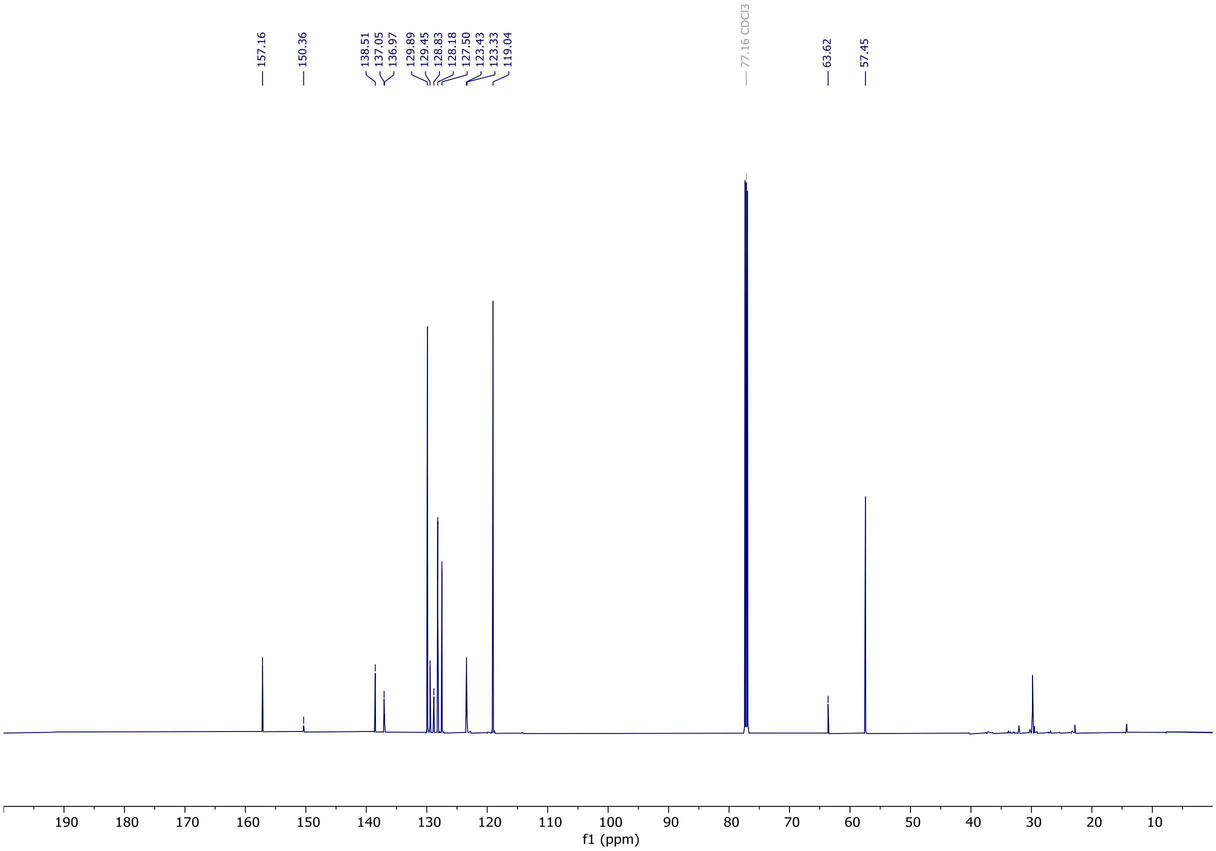

## 25b – $^{1}$H NMR (600 MHz, $CDCl_3$)

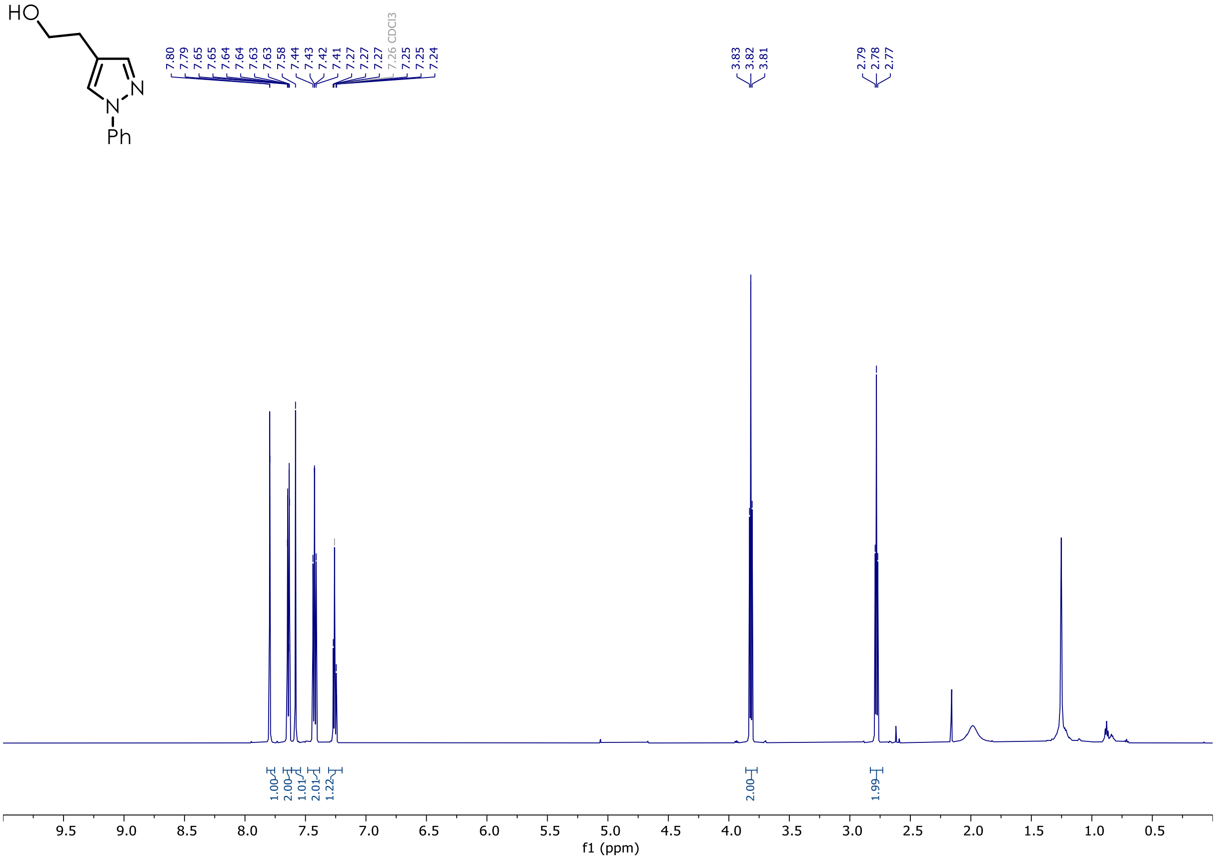

## 25b – $^{13}$C NMR (151 MHz, $CDCl_3$)

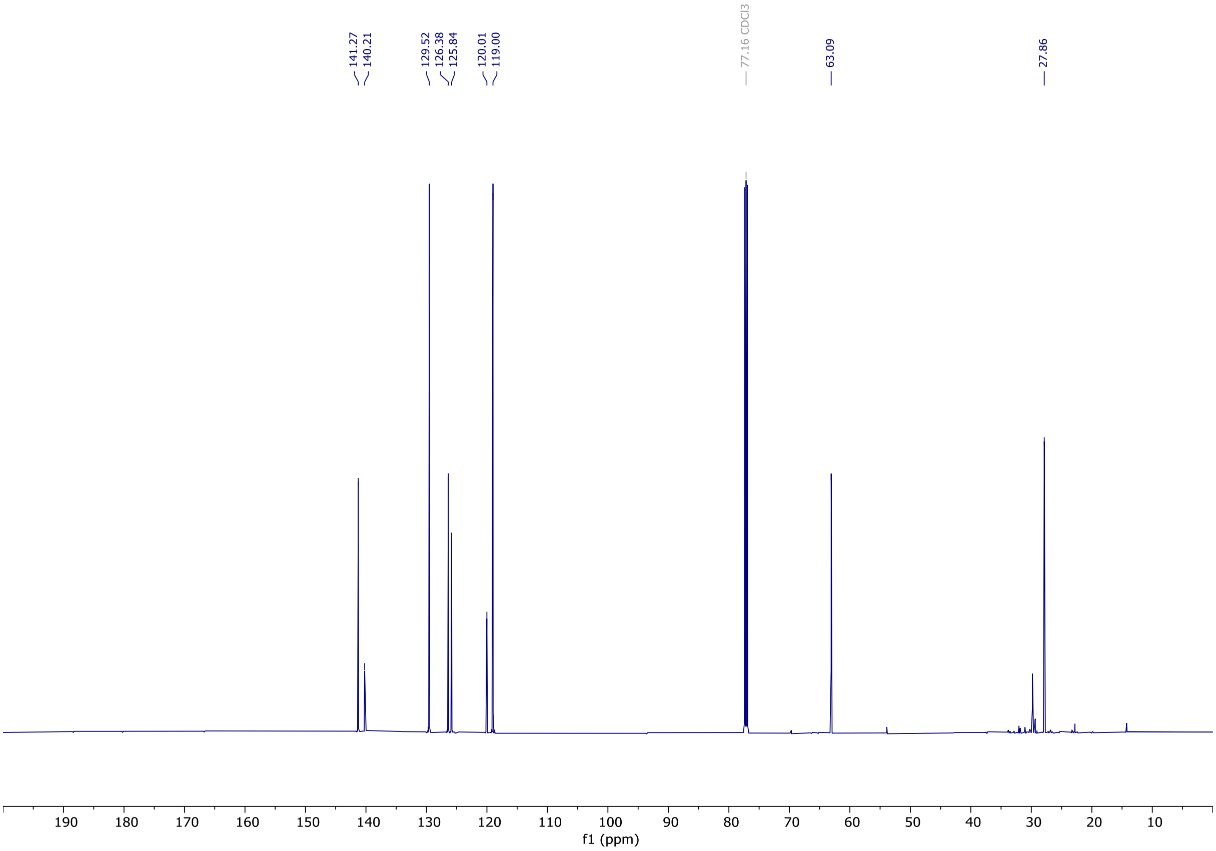

## 26b – $^{1}$H NMR (600 MHz, $CDCl_3$)

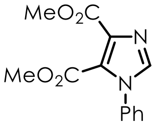

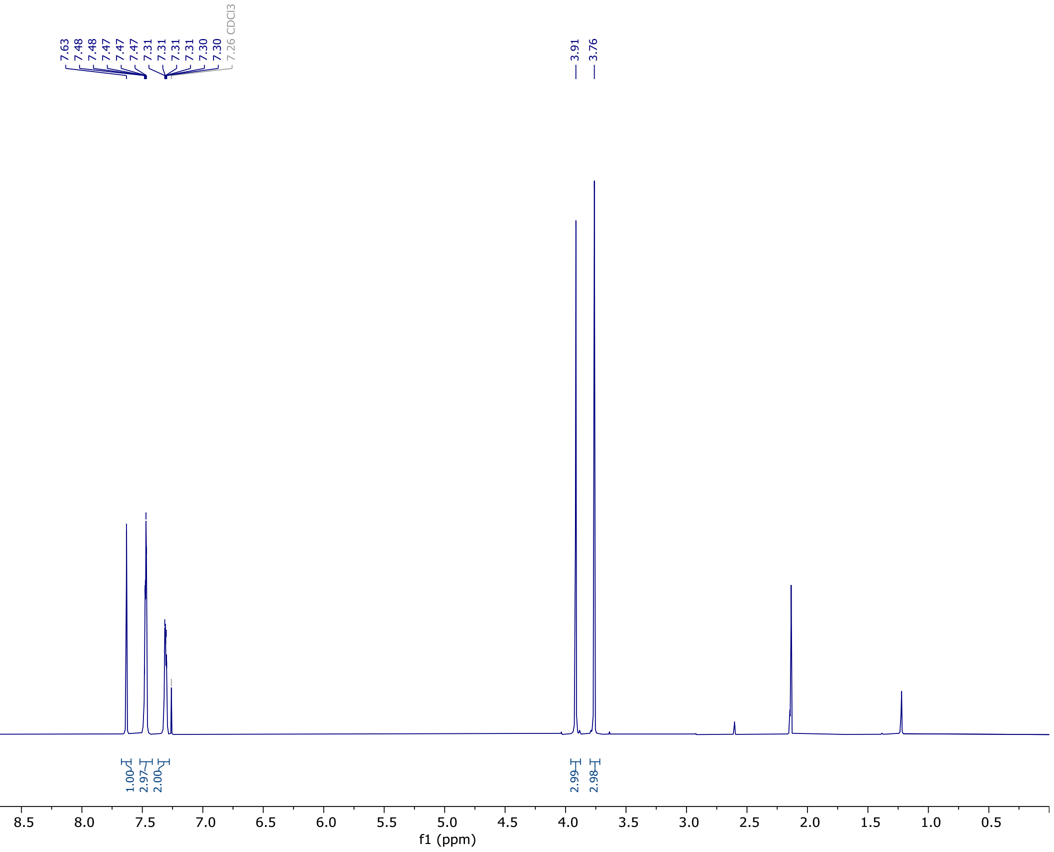

## 26b – $^{13}$C NMR (151 MHz, $CDCl_3$)

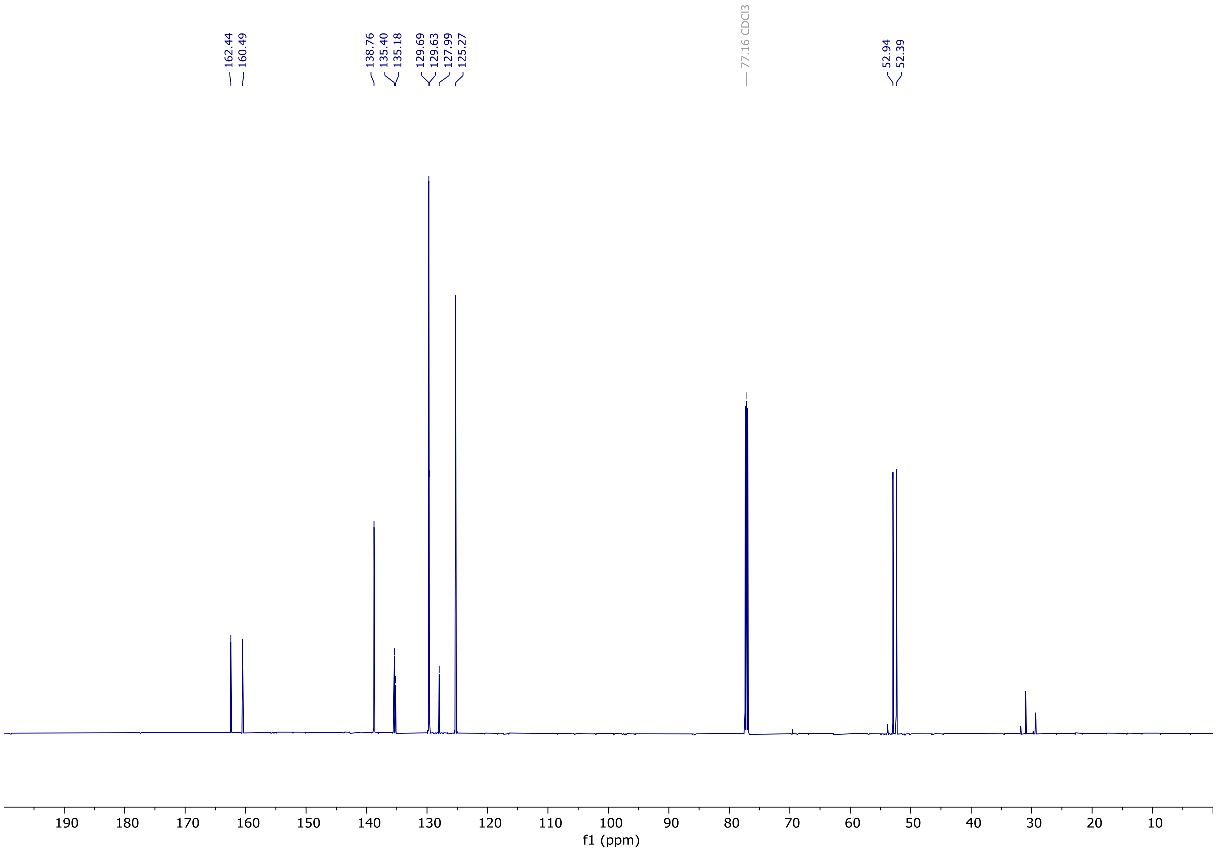

## 27b – $^{1}$H NMR (600 MHz, $CDCl_3$)

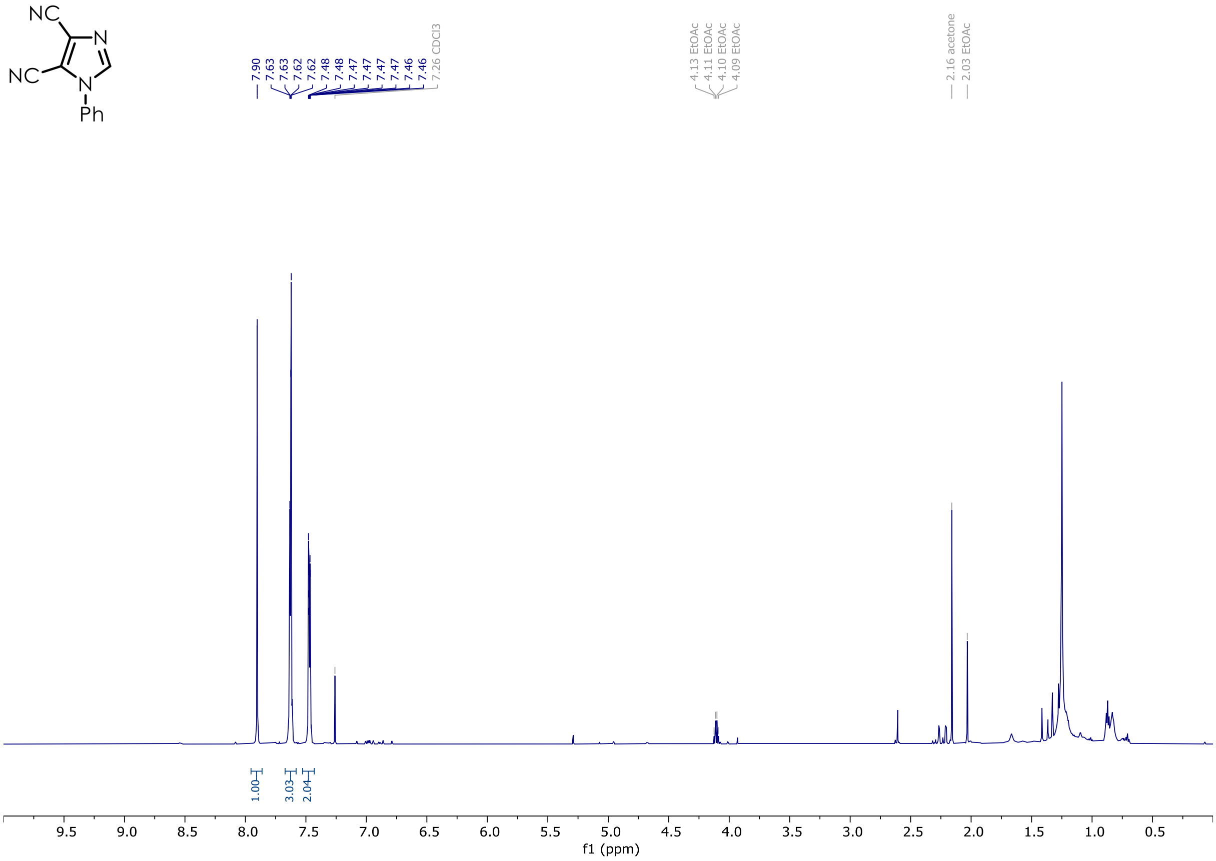


## 27b – $^{13}$C NMR (151 MHz, $CDCl_3$)

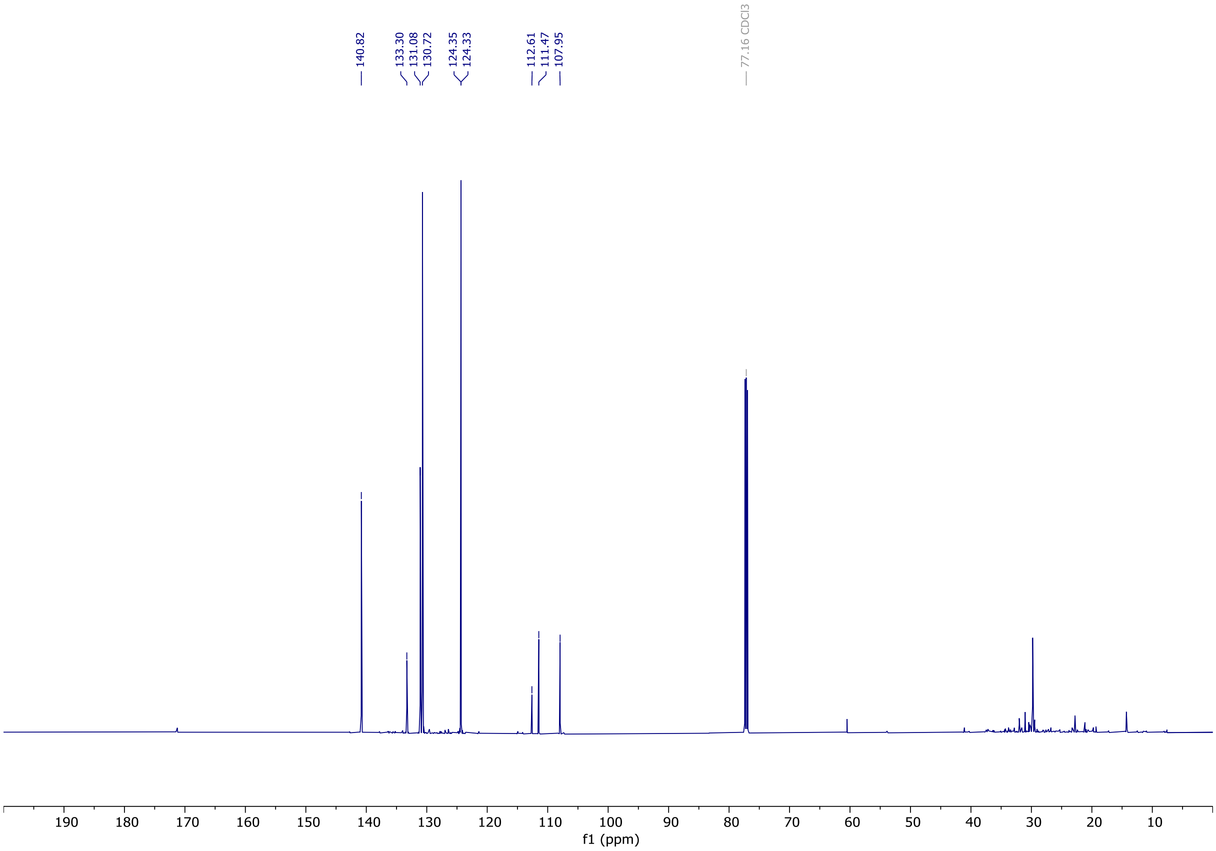

## 28b – $^{1}$H NMR (600 MHz, CDCl$_3$)

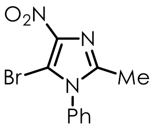

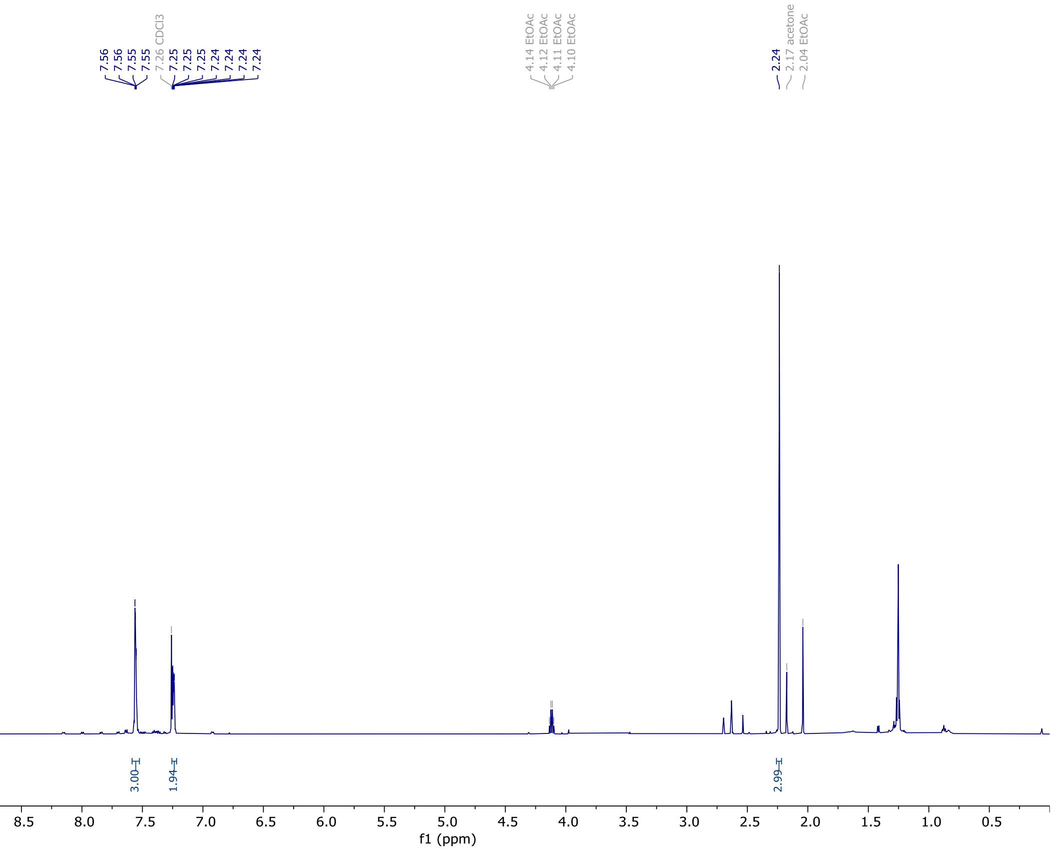


## 28b – $^{13}$C NMR (151 MHz, CDCl$_3$)

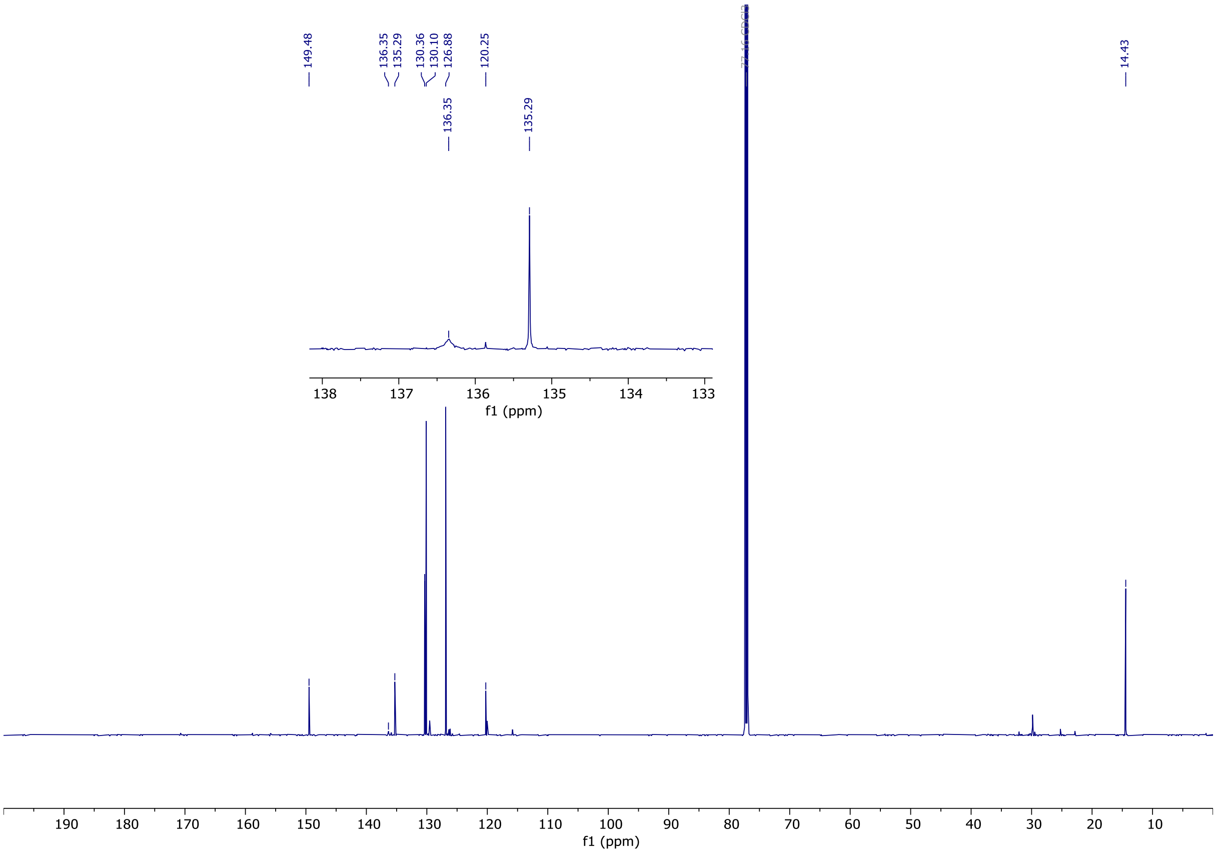

## 28b – $^{1}H$-$^{13}C$ HMBC (600 MHz, $CDCl_3$)

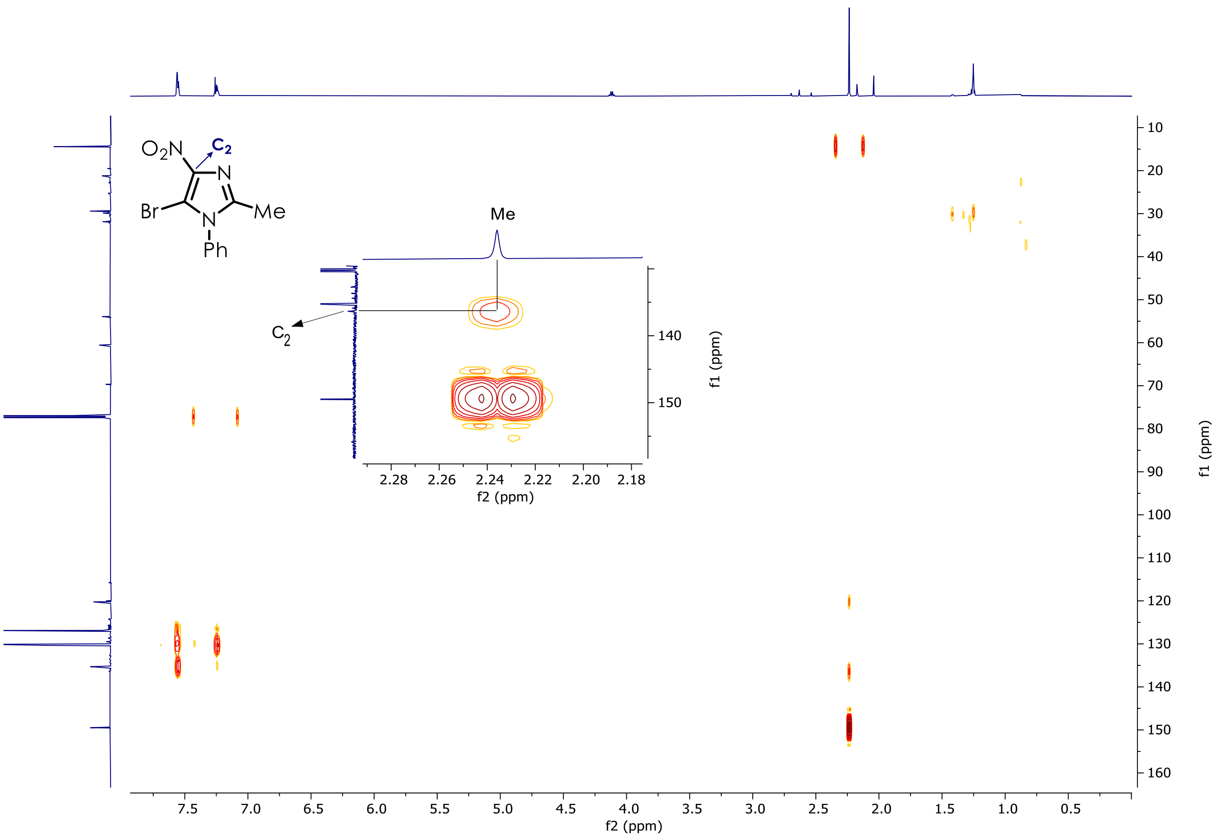

## 29b – $^{1}$H NMR (600 MHz, $CDCl_3$)

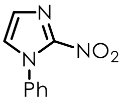


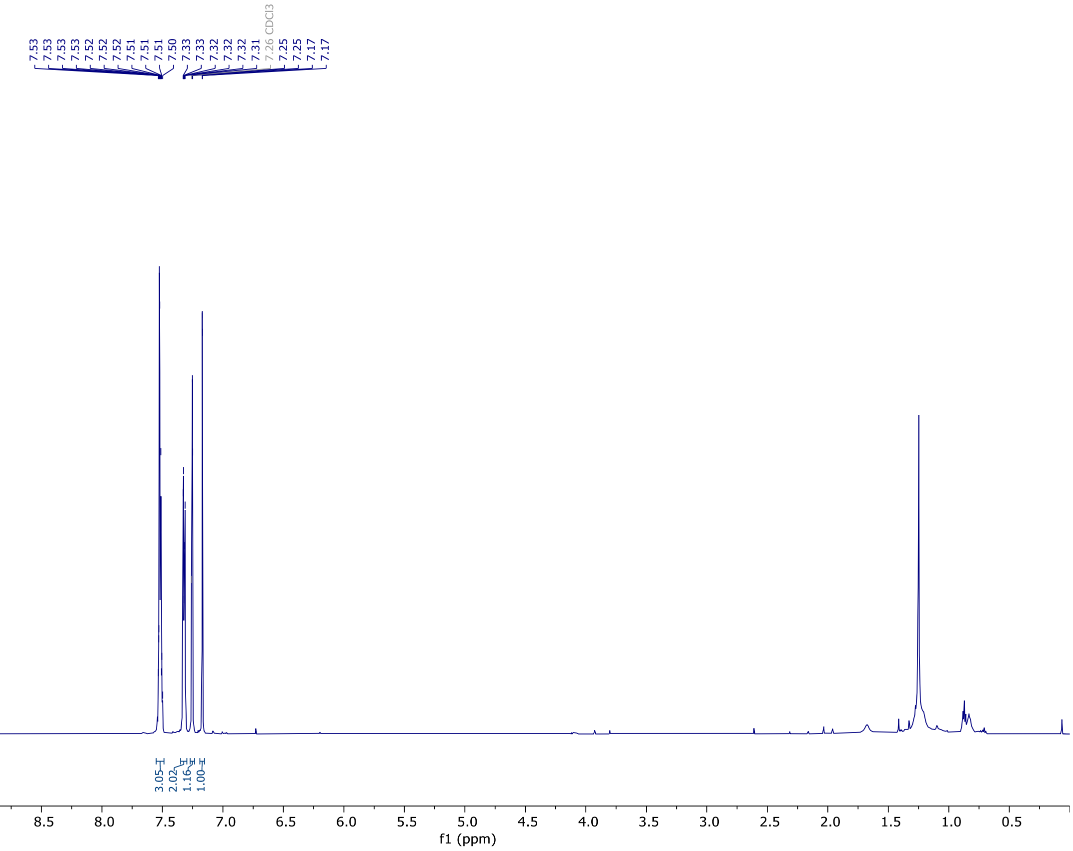


## 29b – $^{13}$C NMR (151 MHz, $CDCl_3$)

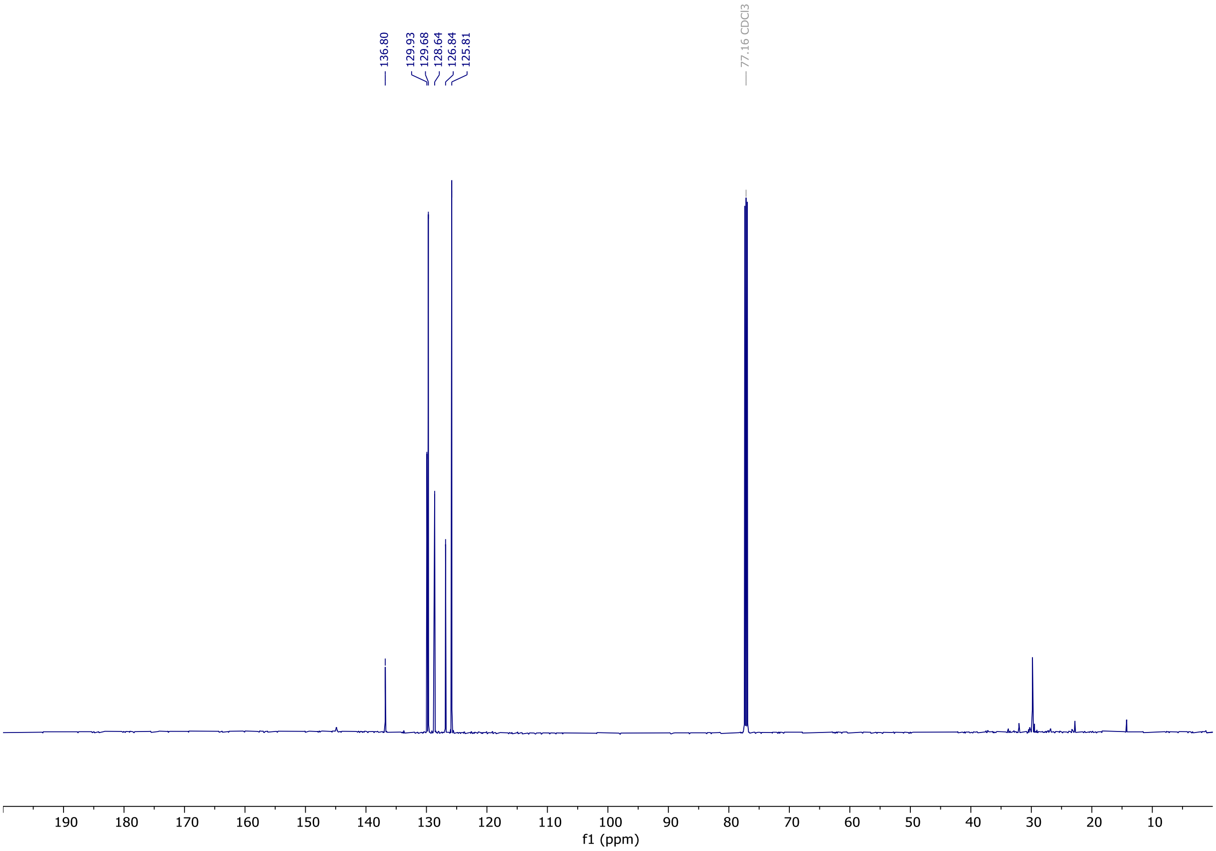

## 30b’ – $^{1}$H NMR (600 MHz, $CDCl_3$)

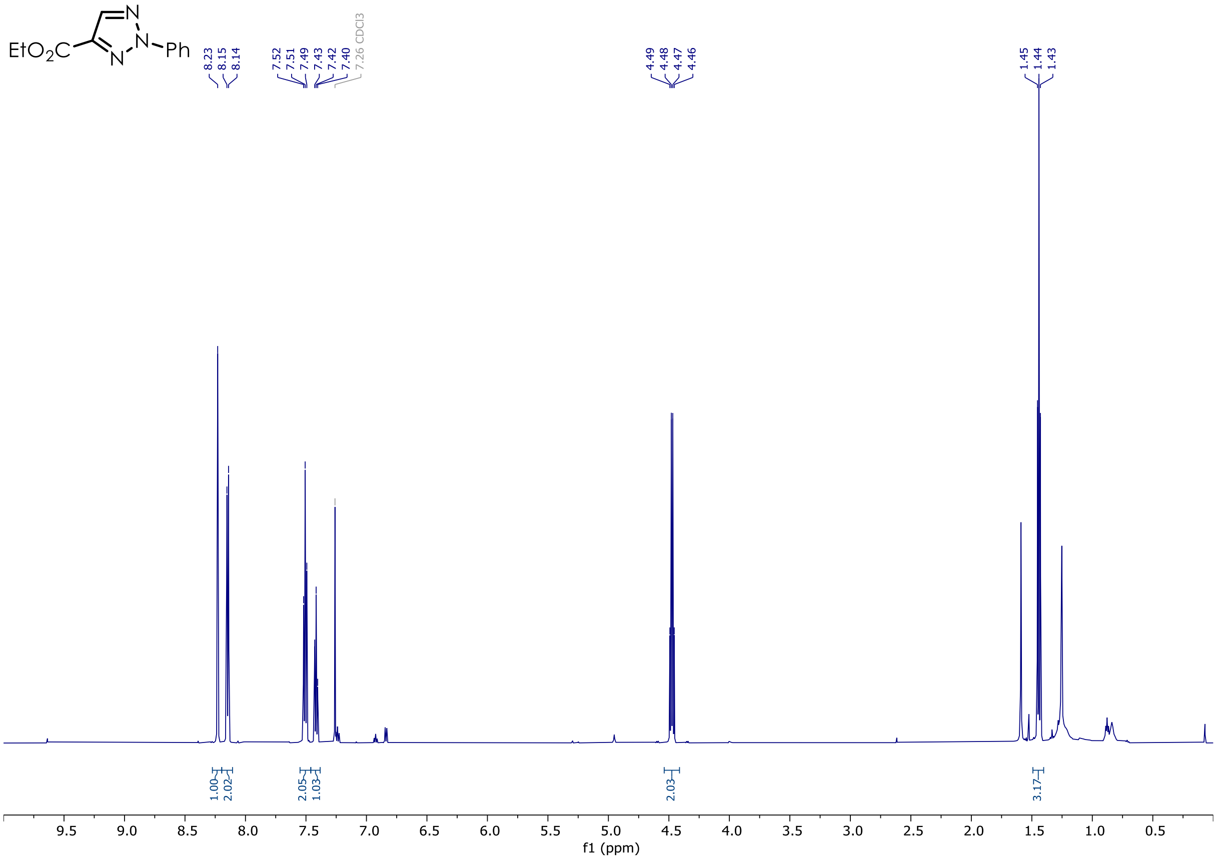


## 30b’ – $^{13}$C NMR (151 MHz, $CDCl_3$)

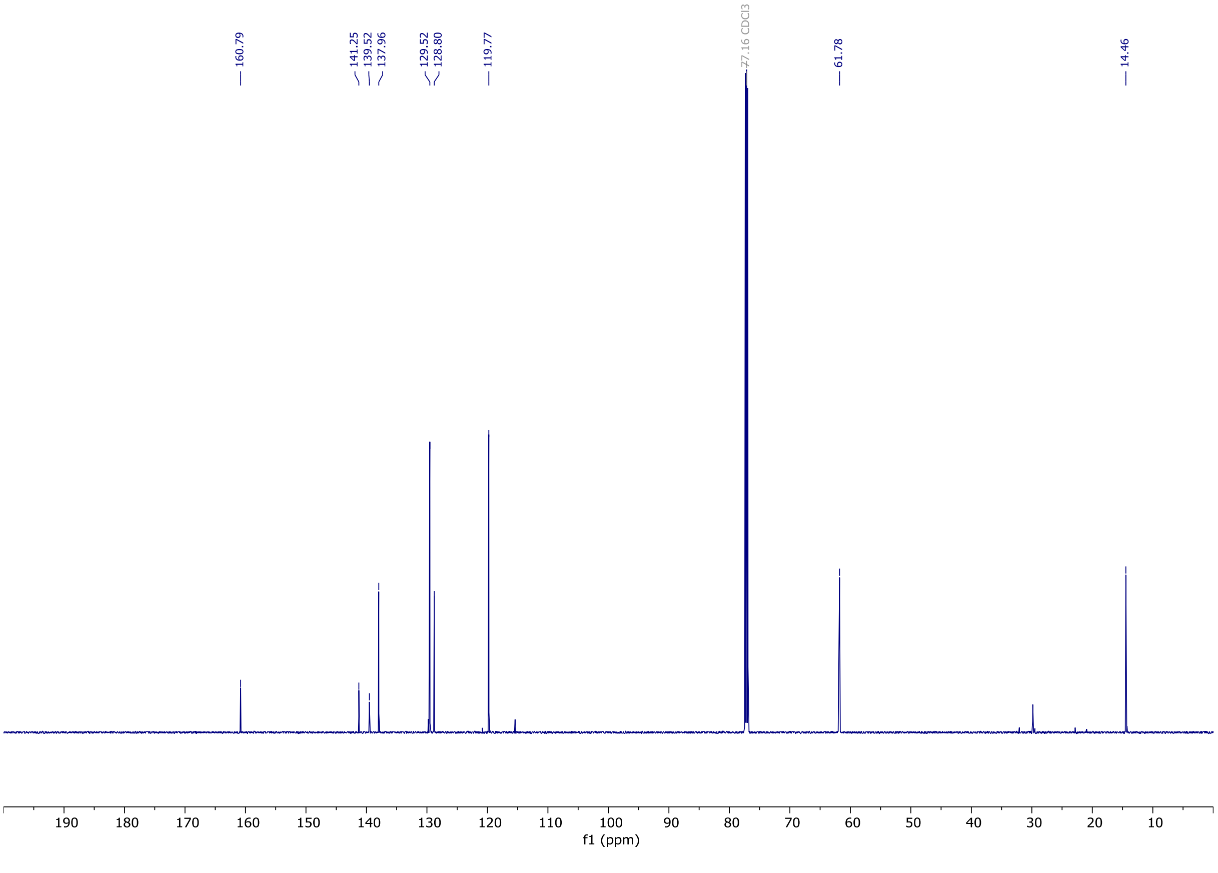

## 30b & 30b'' – ¹H NMR (600 MHz, $CDCl_3$)

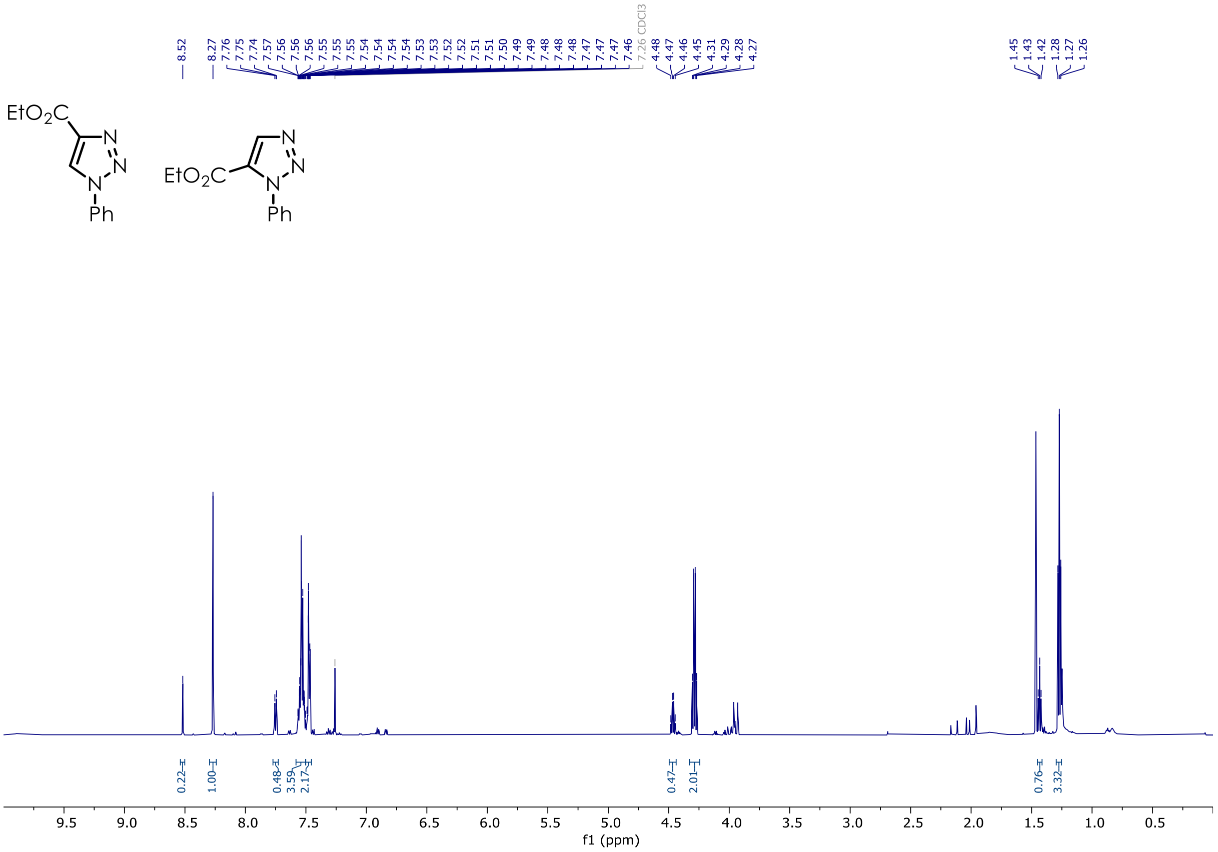


## 30b & 30b'' – ¹³C NMR (151 MHz, $CDCl_3$)

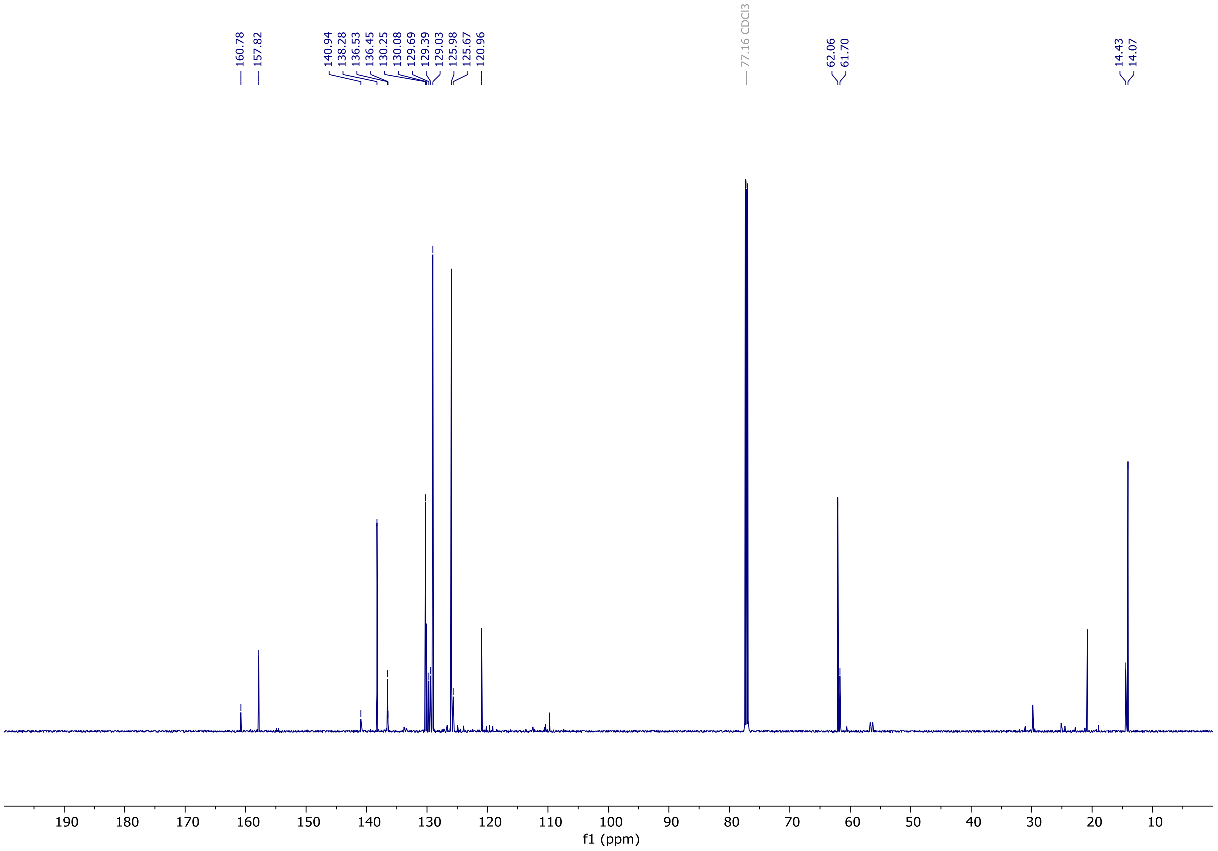

## 31b & 32b – $^{1}$H NMR (600 MHz, $CDCl_3$)

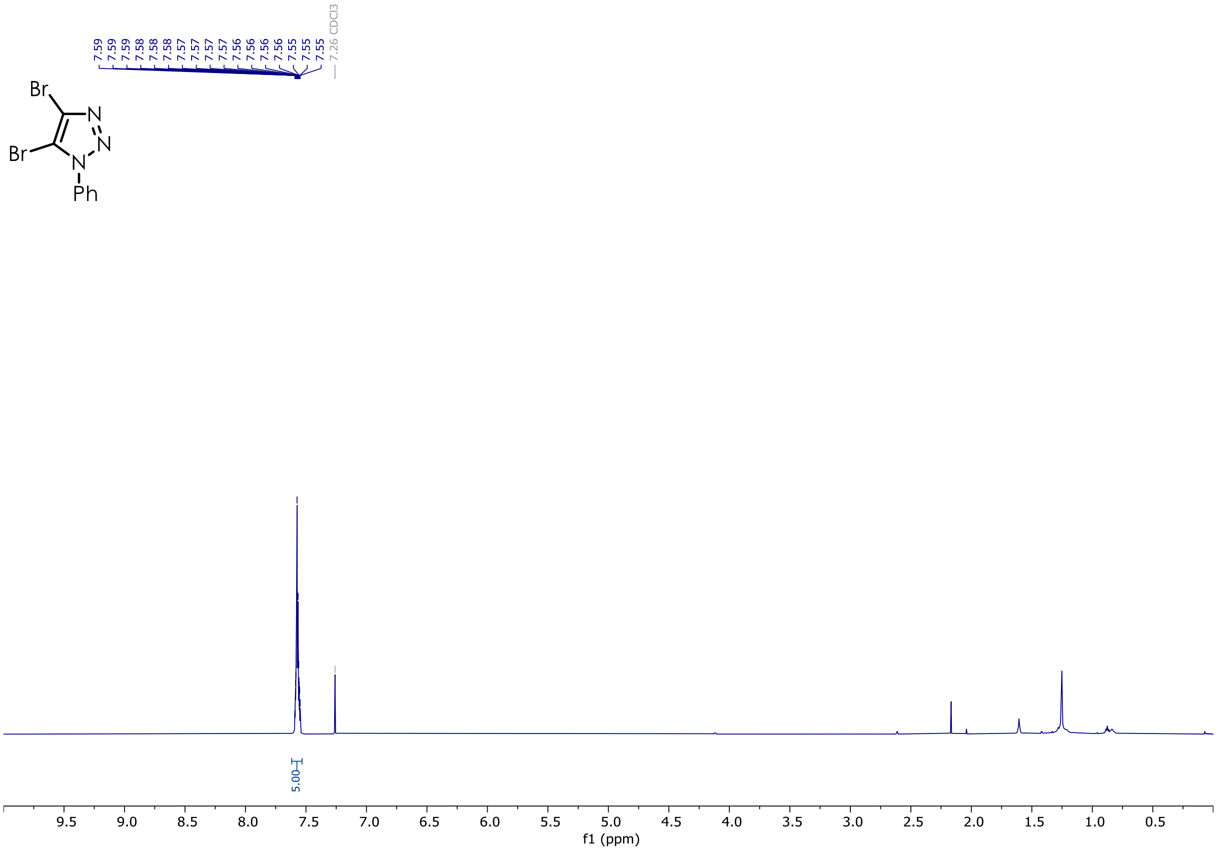


## 31b & 32b – $^{13}$C NMR (151 MHz, $CDCl_3$)

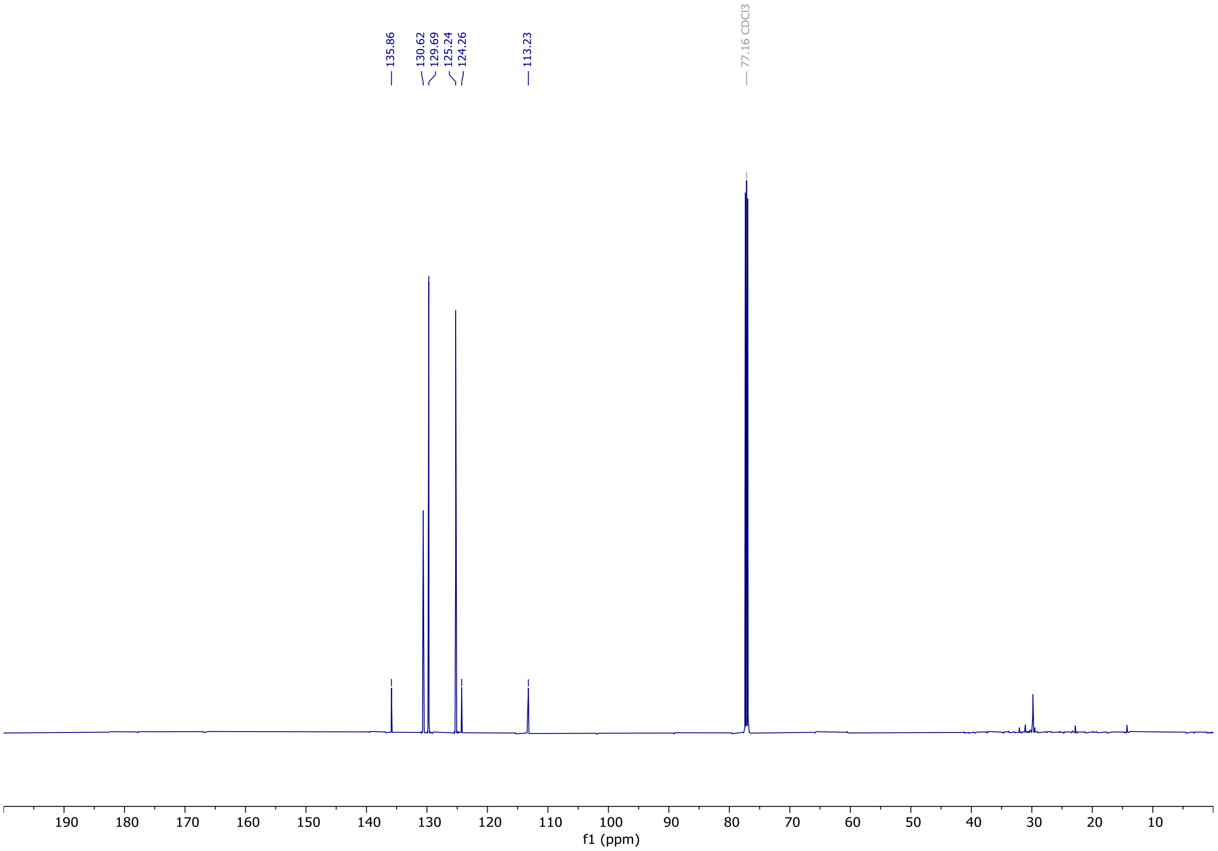

## 33b – ¹H NMR (600 MHz, CDCl₃)

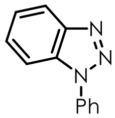

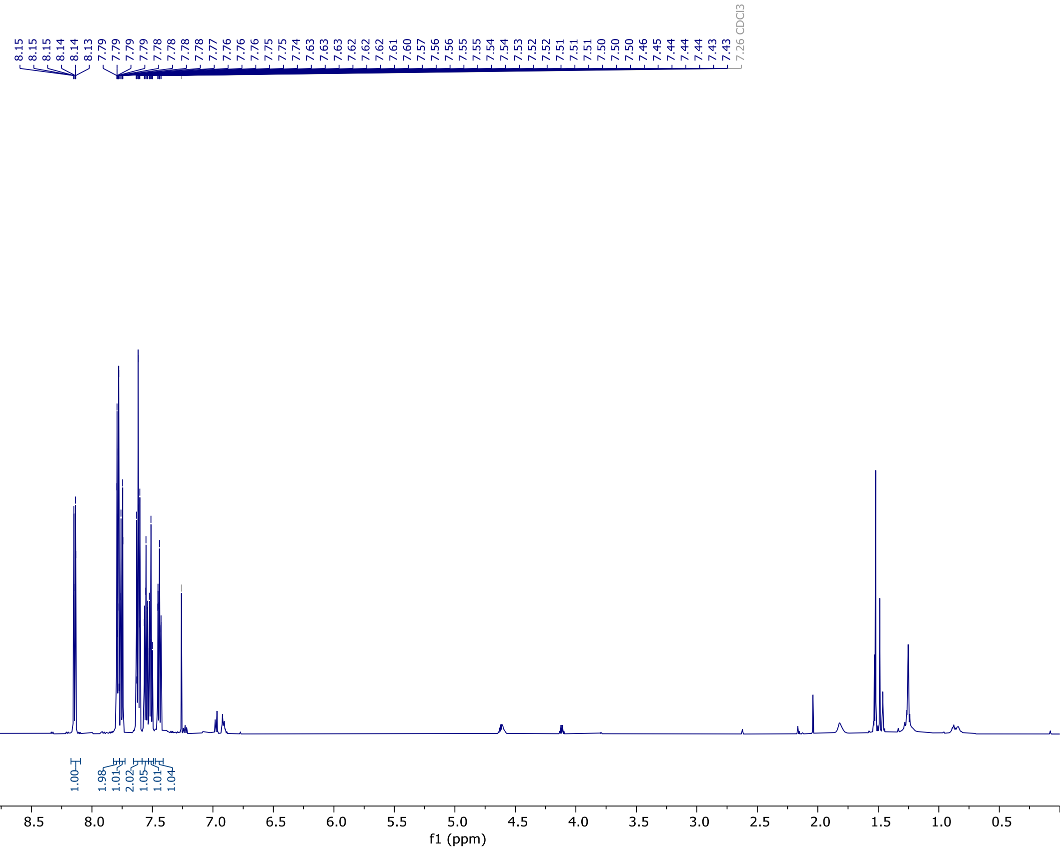

## 33b – ¹³C NMR (151 MHz, CDCl₃)

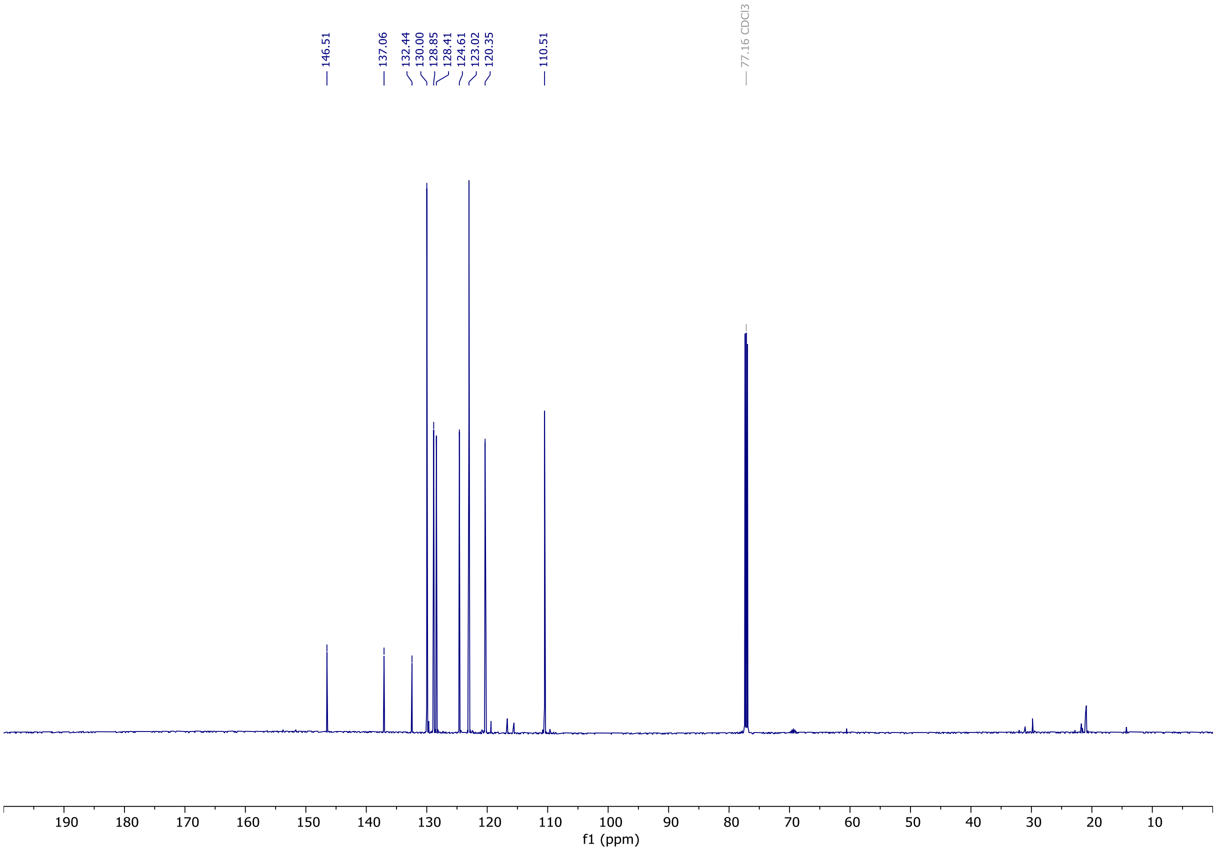

## 34b & 34b' – $^{1}$H NMR (600 MHz, $CDCl_3$)

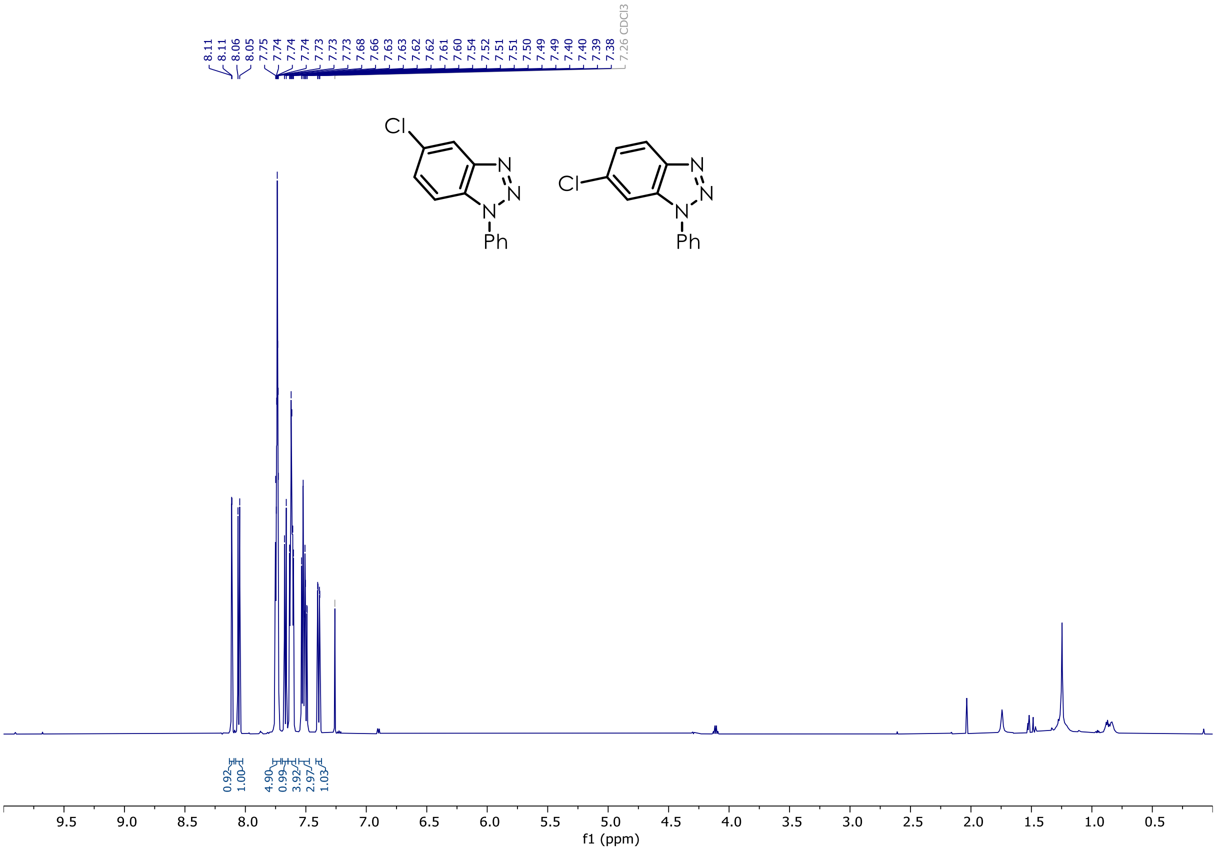

## 34b & 34b' – $^{13}$C NMR (151 MHz, $CDCl_3$)

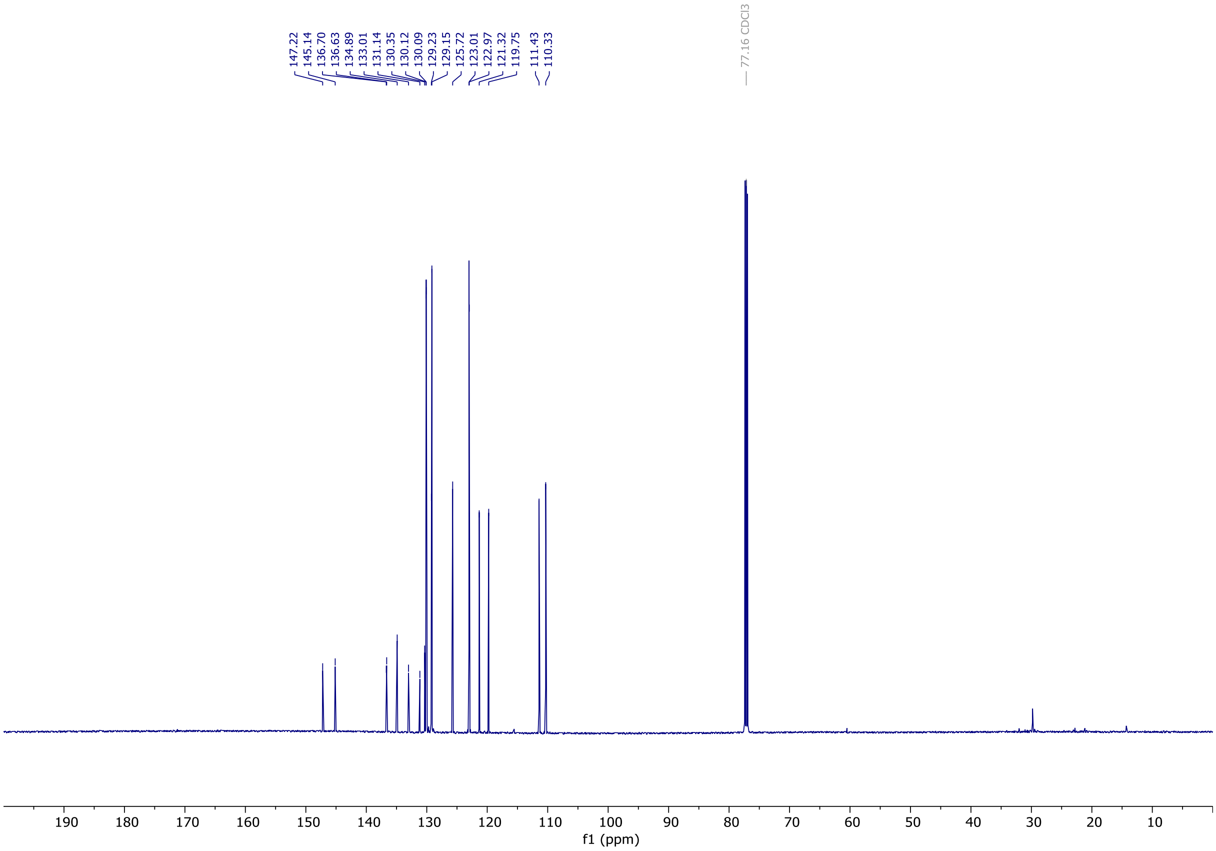

## 35b & 35b' – $^{1}$H NMR (600 MHz, $CDCl_3$)

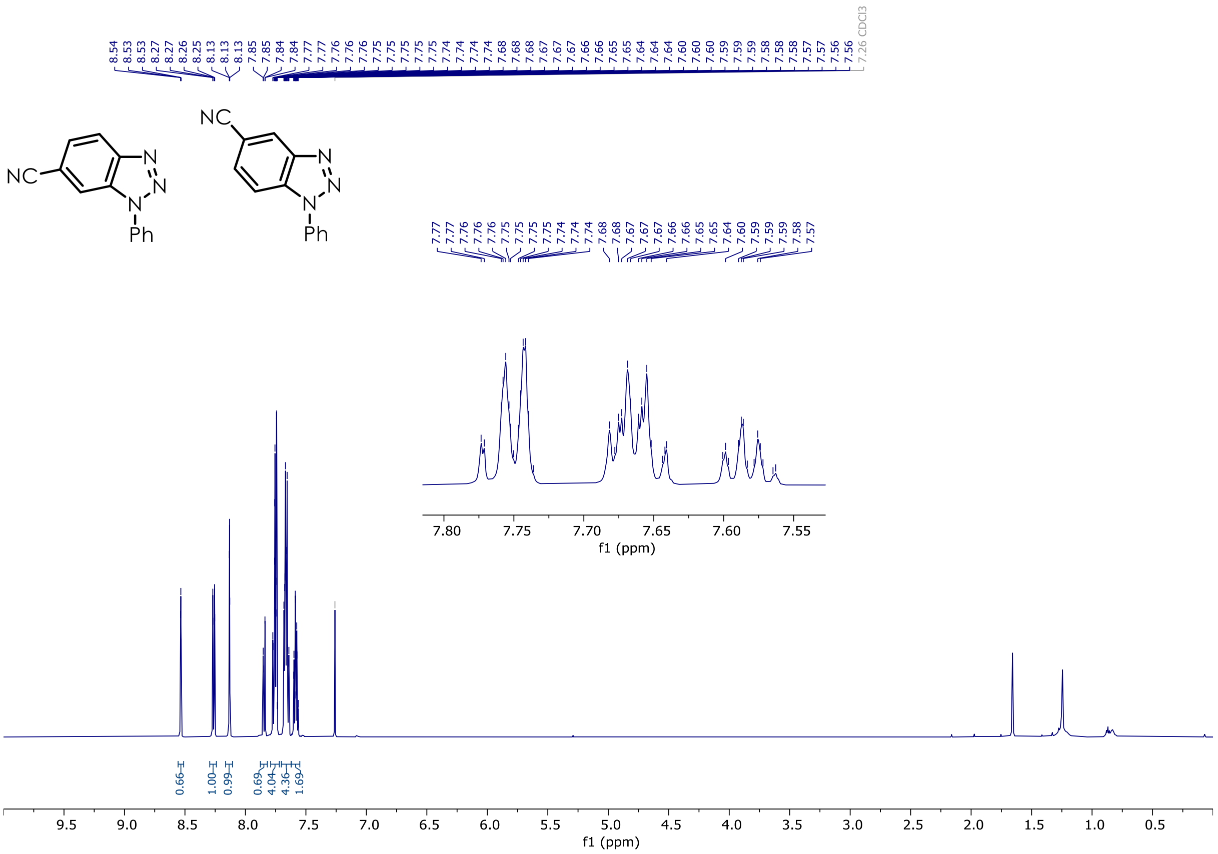


## 35b & 35b' – $^{13}$C NMR (151 MHz, $CDCl_3$)

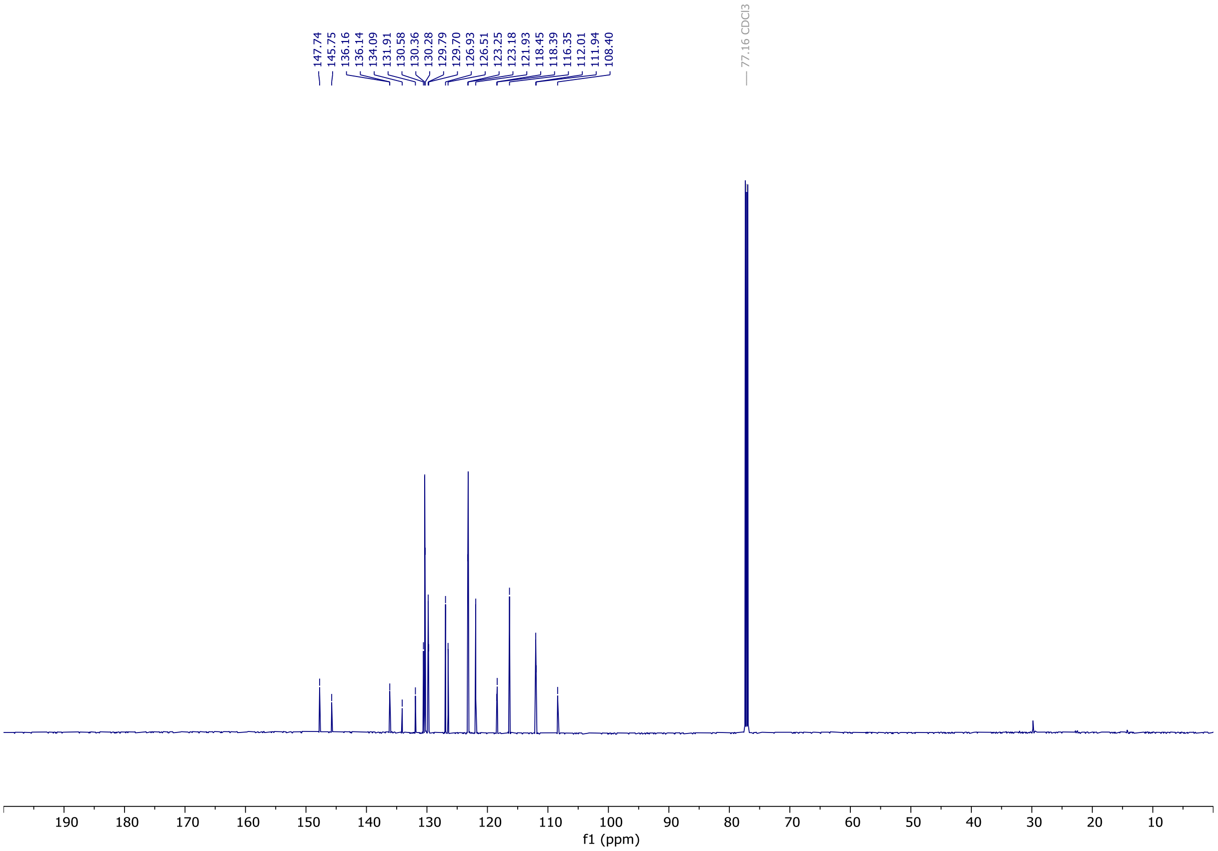

## 36b – $^{1}$H NMR (600 MHz, $CDCl_3$)

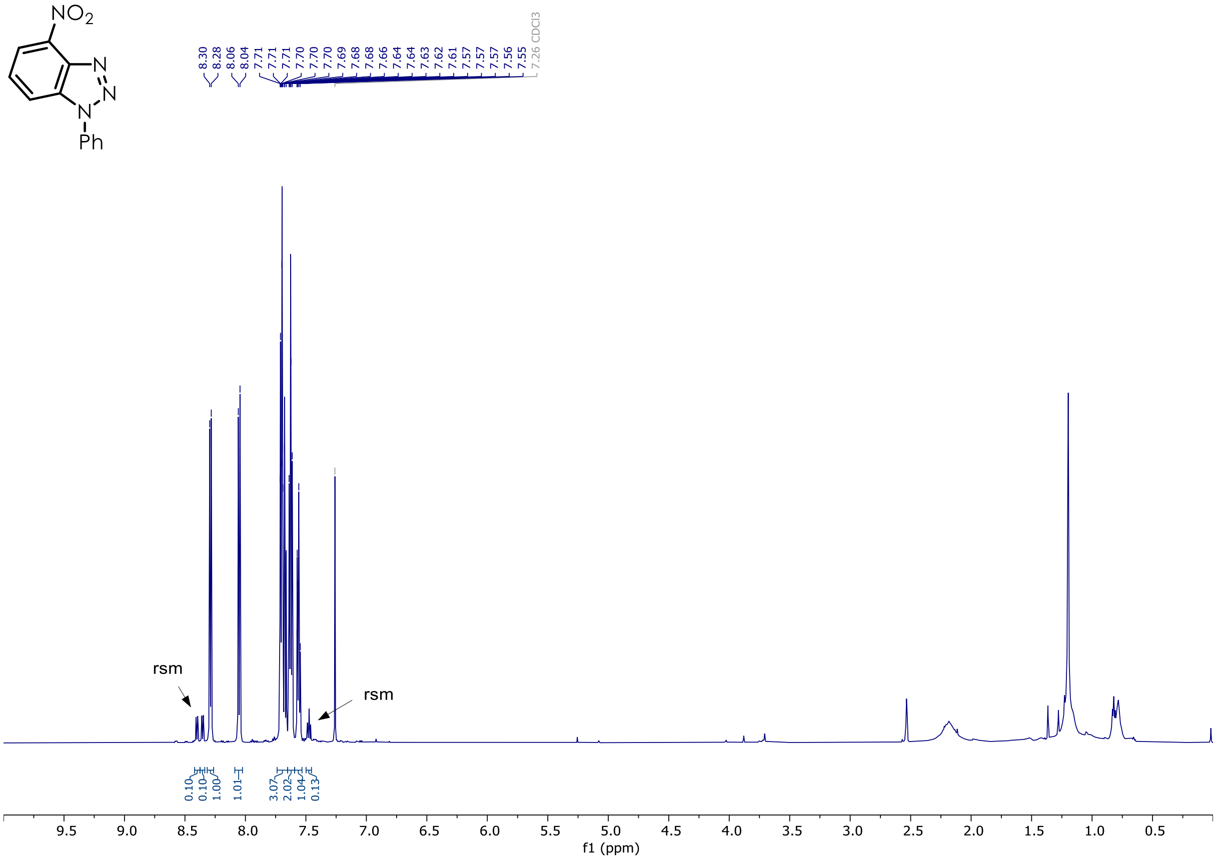


## 36b – $^{13}$C NMR (151 MHz, $CDCl_3$)

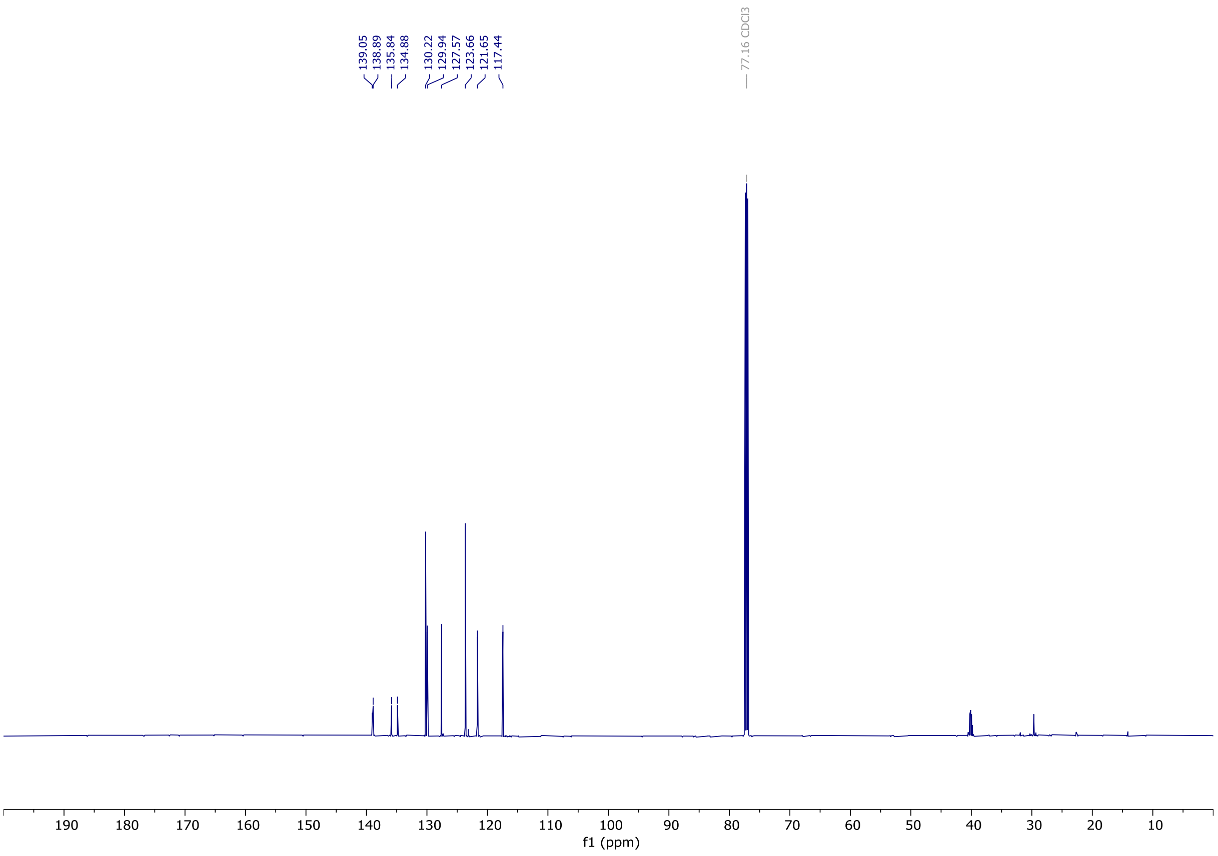

## 37b – ¹H NMR (600 MHz, $CDCl_3$)

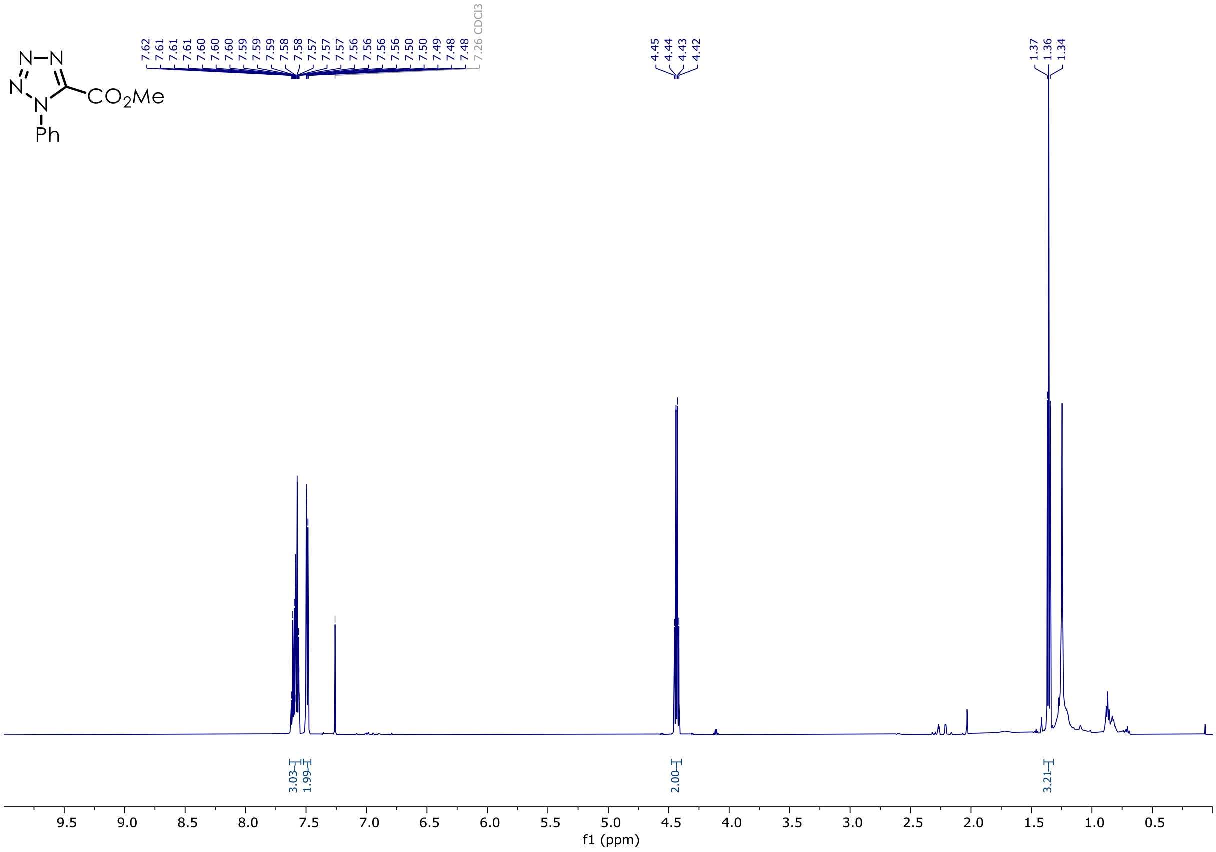


## 37b – ¹³C NMR (151 MHz, $CDCl_3$)

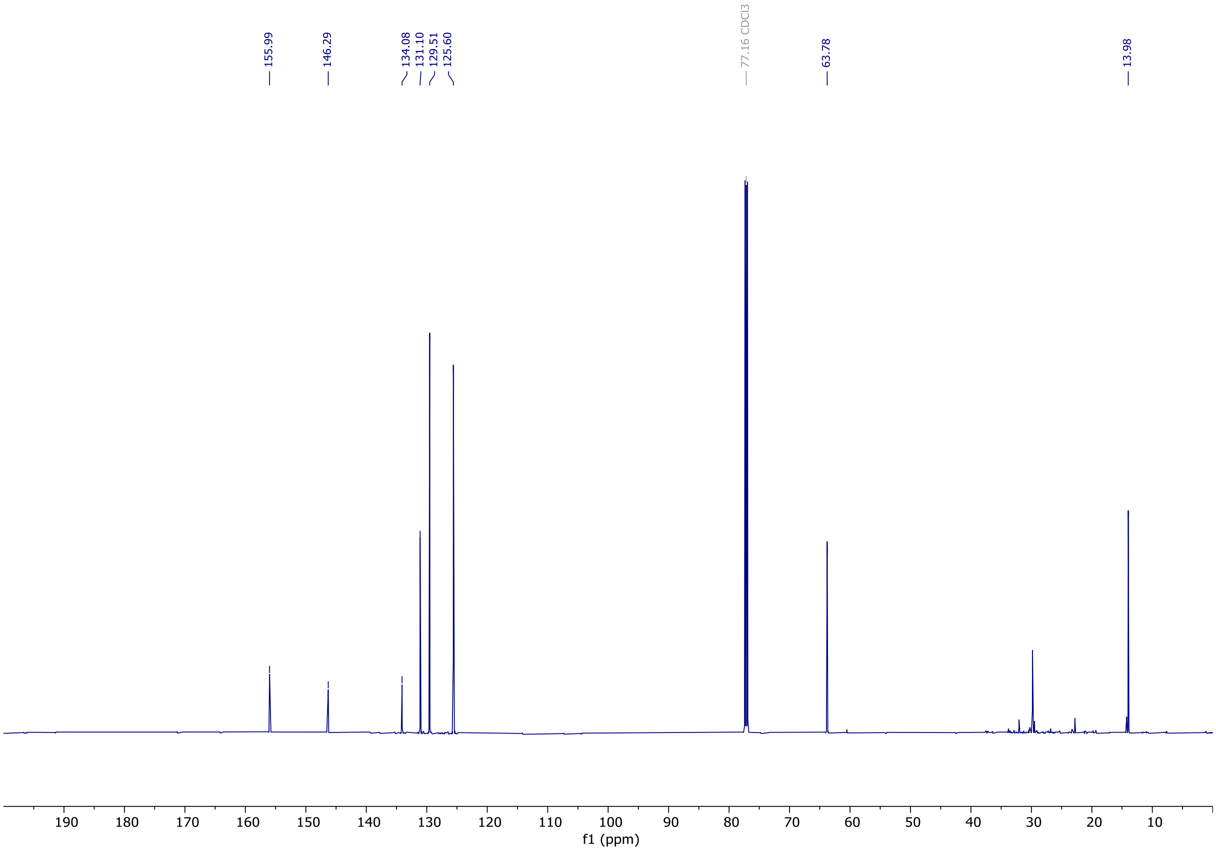

## 37b' – $^{1}$H NMR (600 MHz, $CDCl_3$)

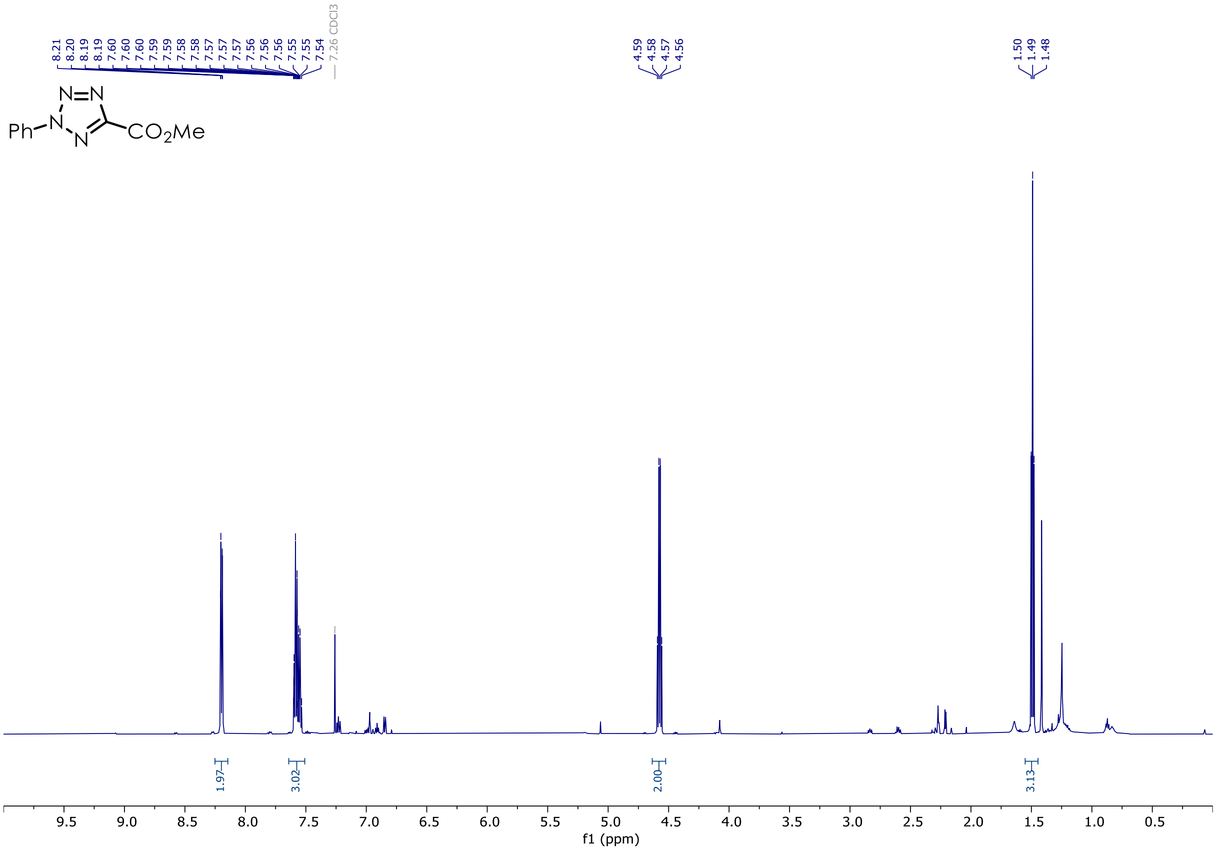

## 37b' – $^{13}$C NMR (151 MHz, $CDCl_3$)

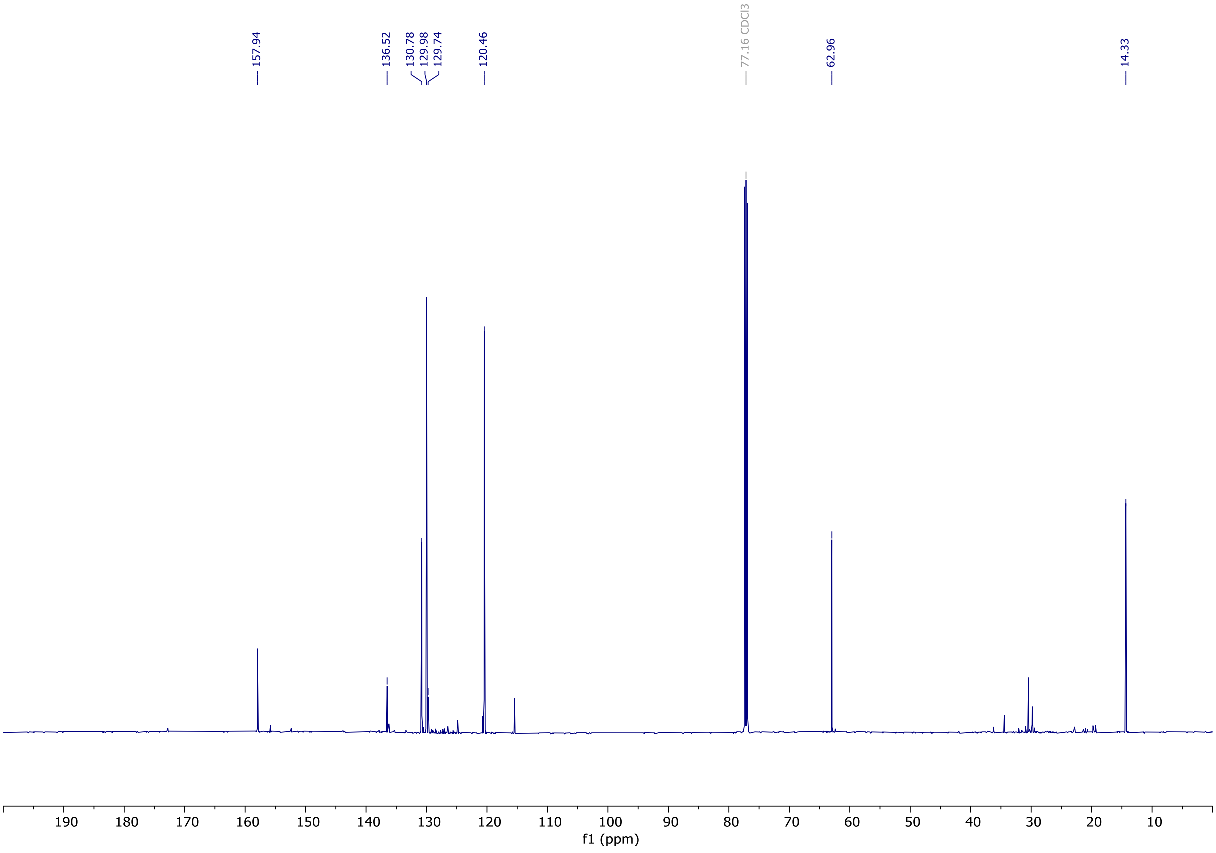

# 38b – $^{1}$H NMR (600 MHz, $CDCl_3$)

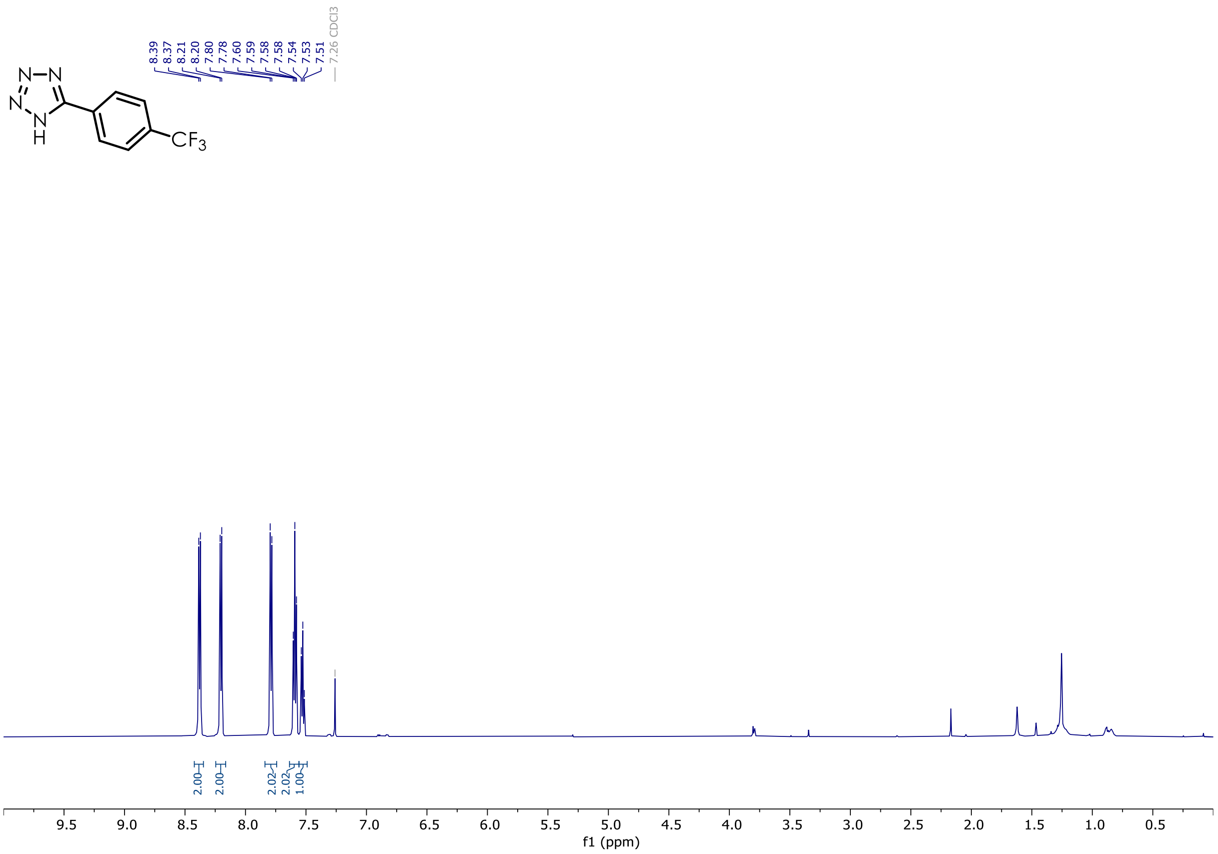


# 38b – $^{13}$C NMR (151 MHz, $CDCl_3$)

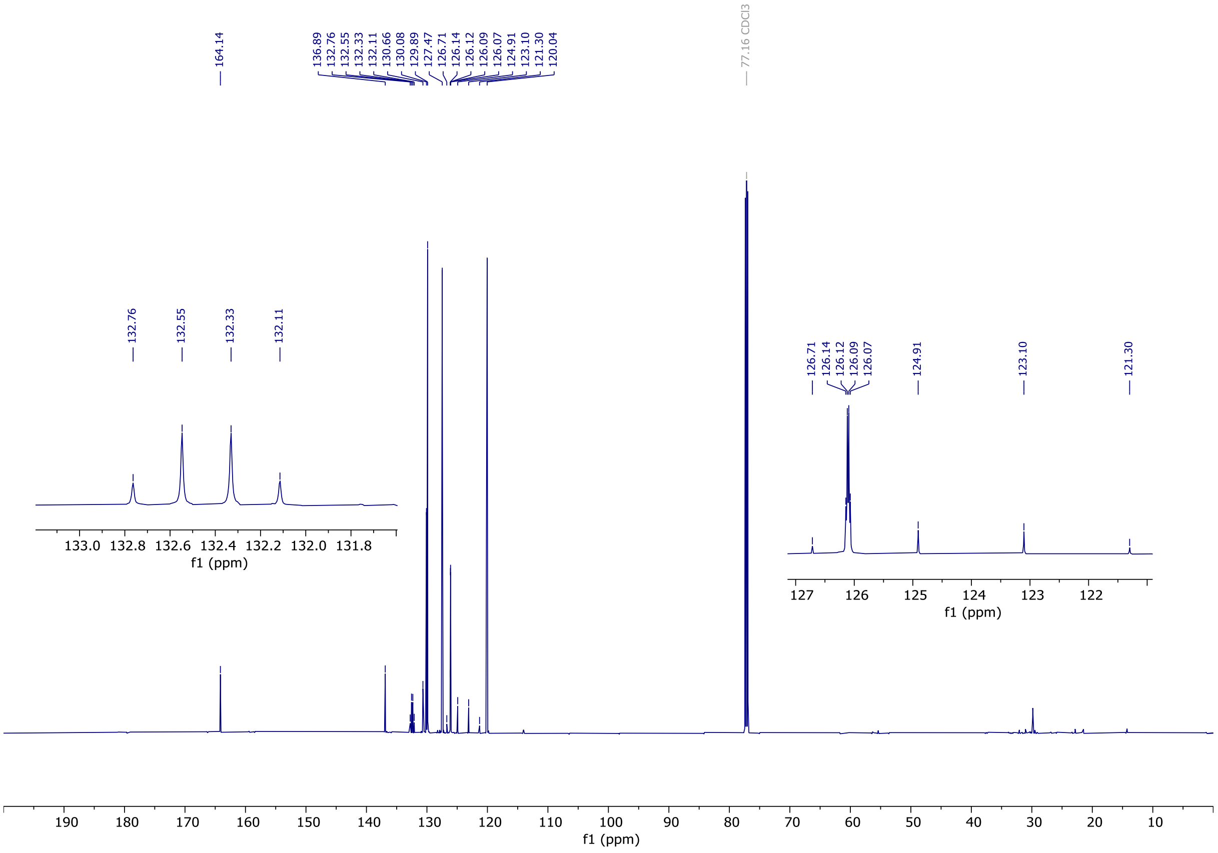

**38b – $^{19}$F NMR (565 MHz, CDCl$_3$)**

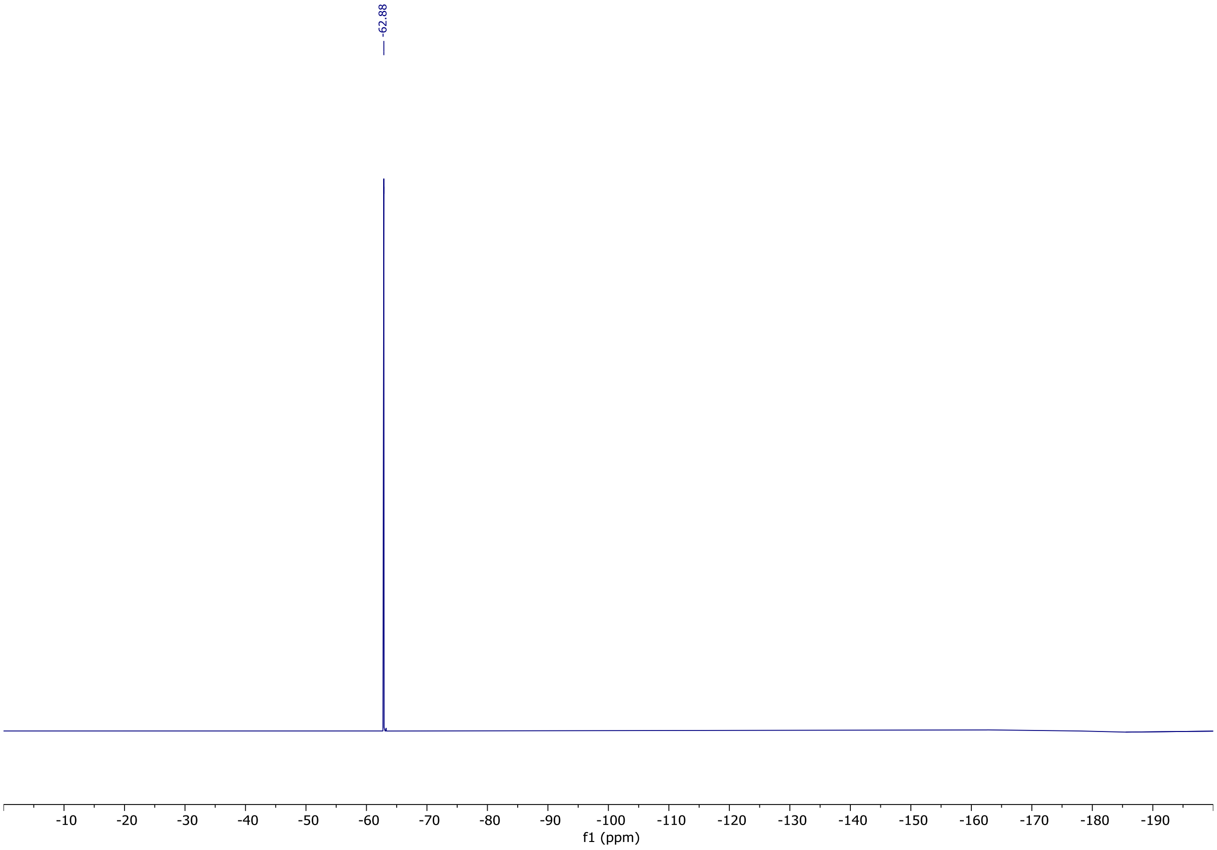

## 39b – $^{1}$H NMR (600 MHz, $CDCl_3$)

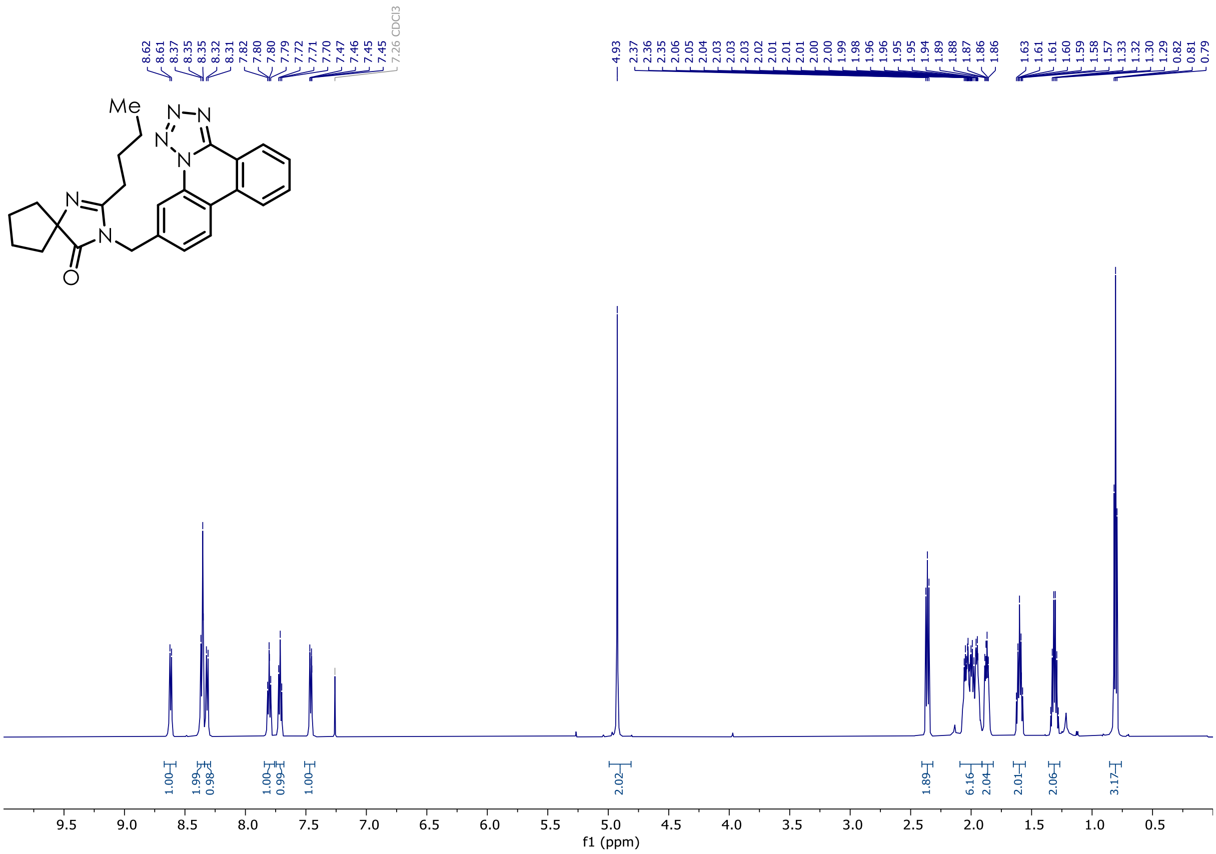


## 39b – $^{13}$C NMR (151 MHz, $CDCl_3$)

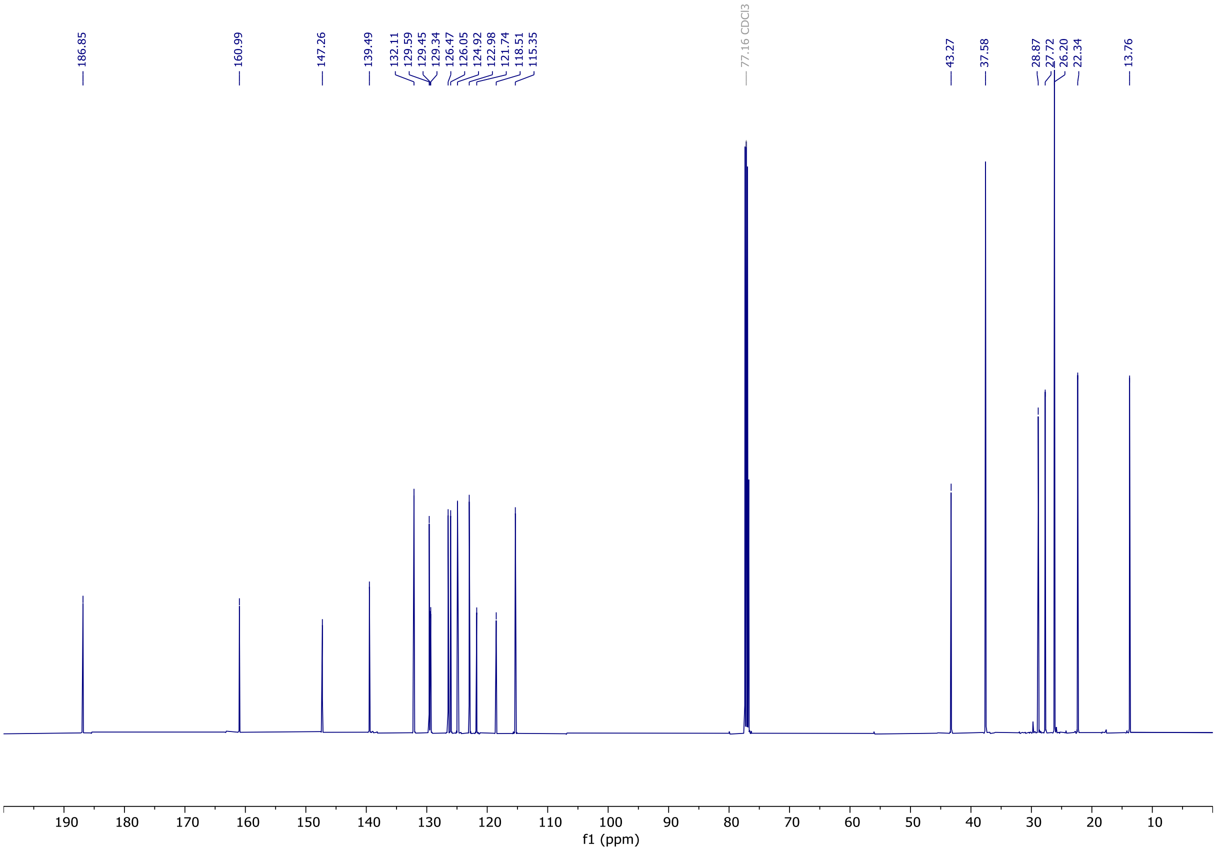

## 39b – DEPT $^{13}C$ NMR (151 MHz, $CDCl_3$)

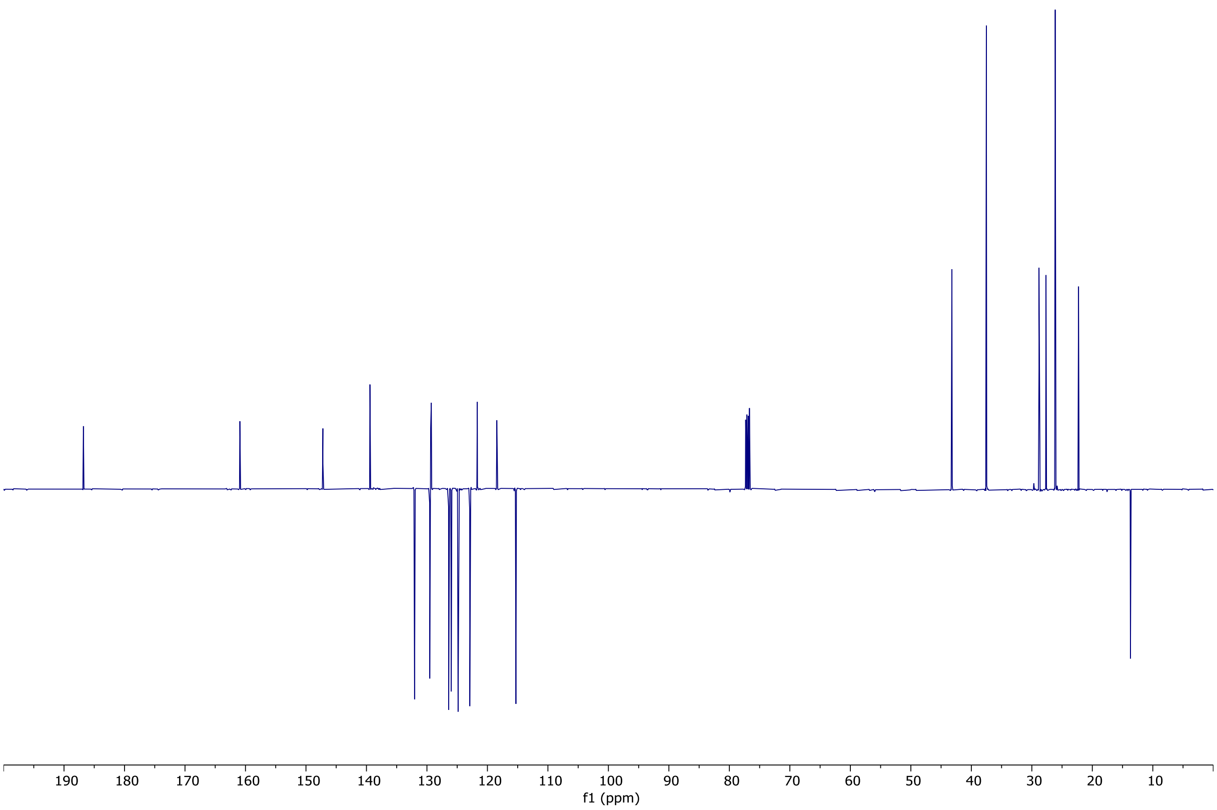


## 39b – $^{1}H$-$^{1}H$ COSY NMR (600 MHz, $CDCl_3$)

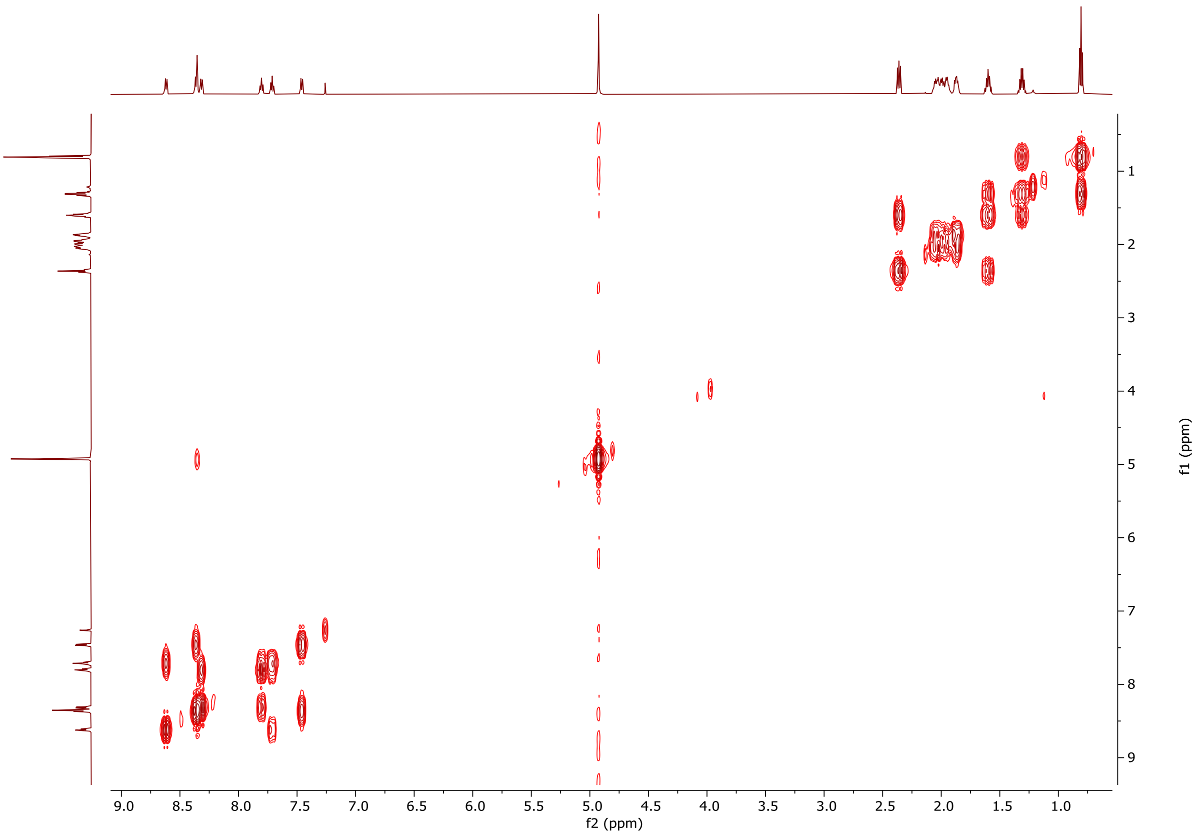

## 39b – $^{1}H$-$^{13}C$ HMBC NMR (151 MHz, $CDCl_3$)

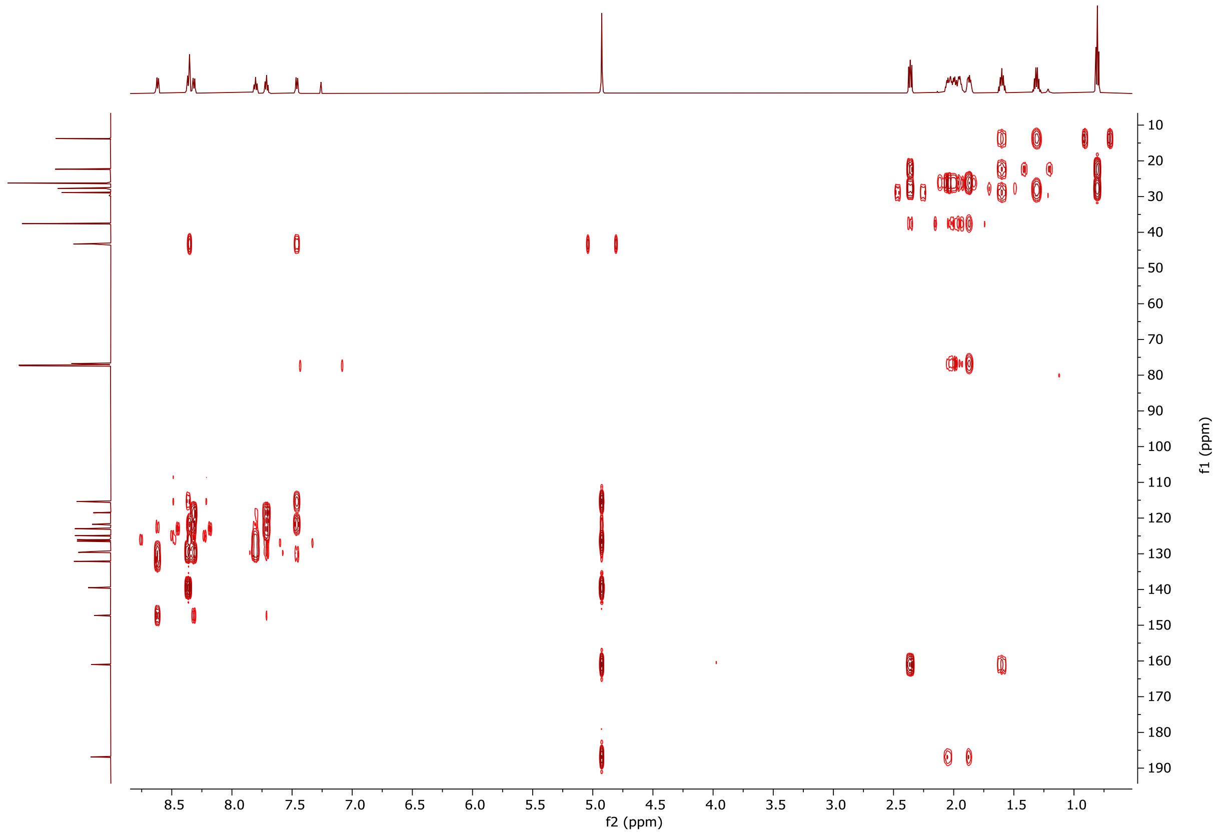


## 39b – $^{1}H$-$^{13}C$ HSQC NMR (151 MHz, $CDCl_3$)

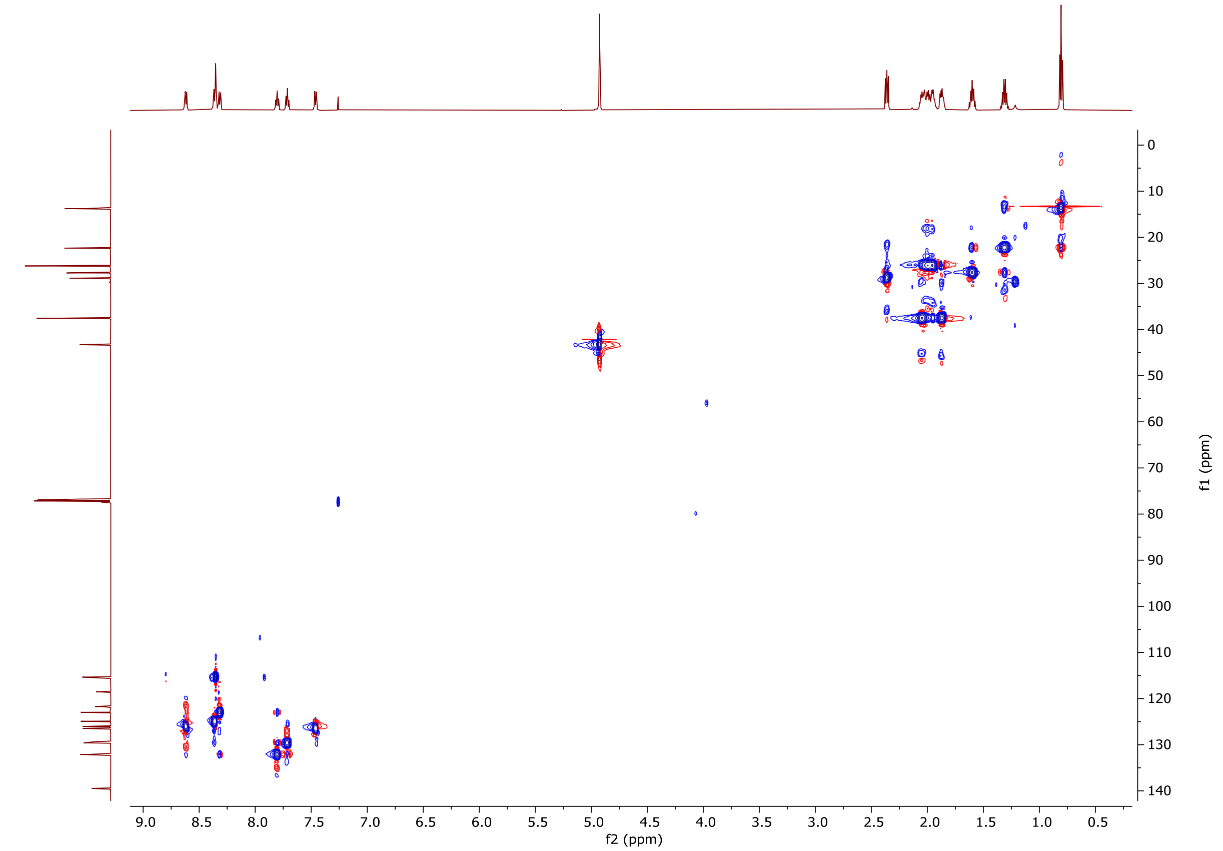

## 40b – $^{1}H$ NMR (600 MHz, $CDCl_3$)

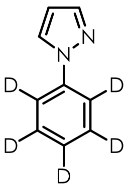

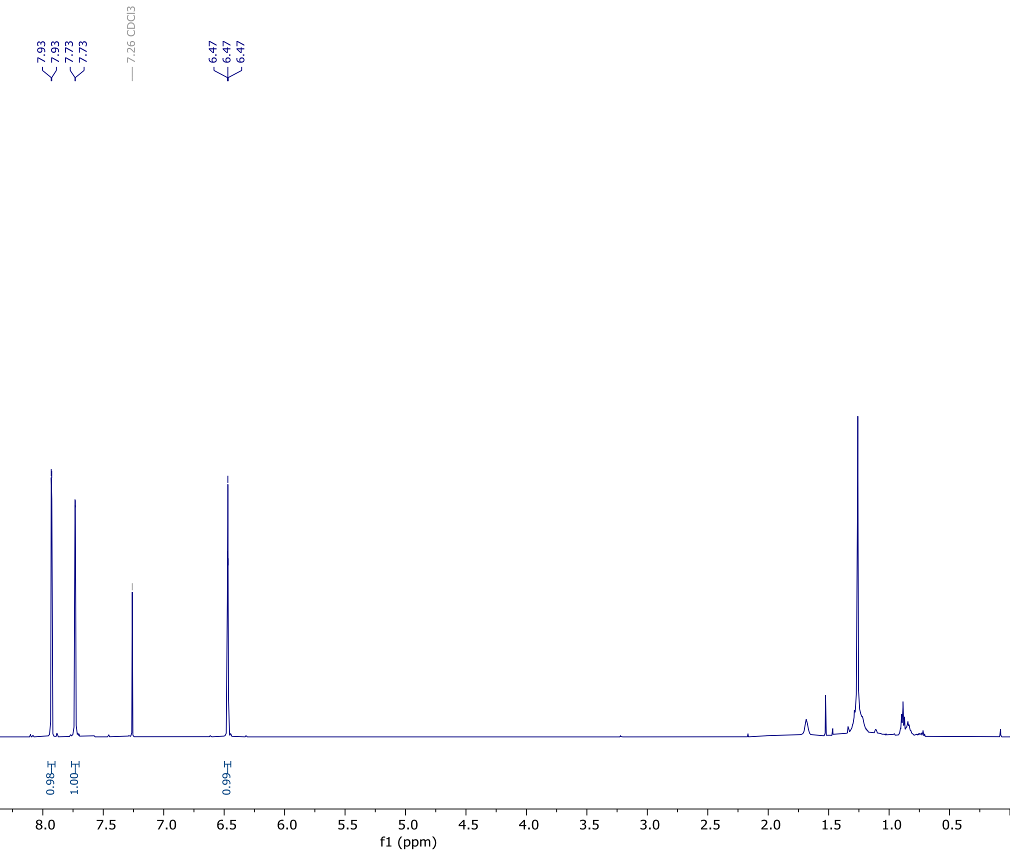


## 40b – $^{2}H$ NMR (92 MHz, $CDCl_3$)

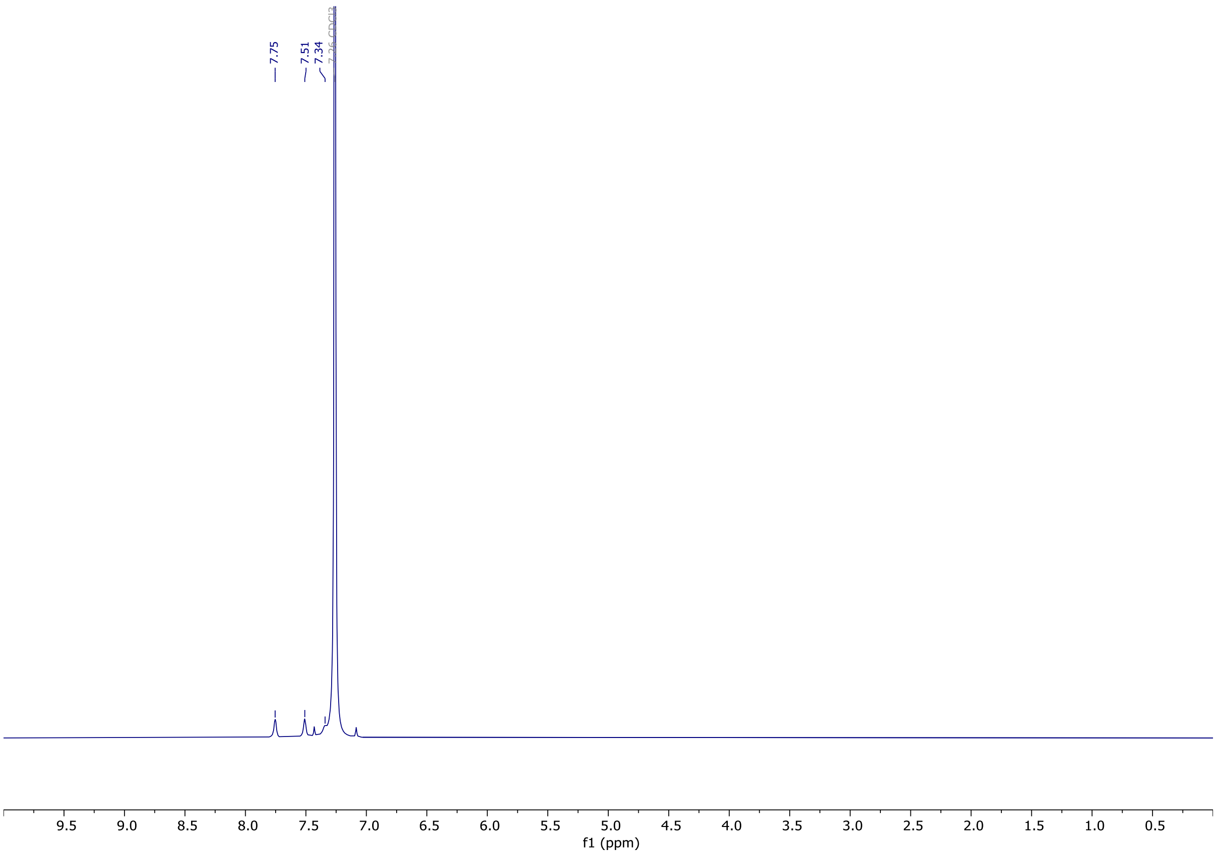

**40b – $^{13}$C NMR (151 MHz, CDCl$_3$)**

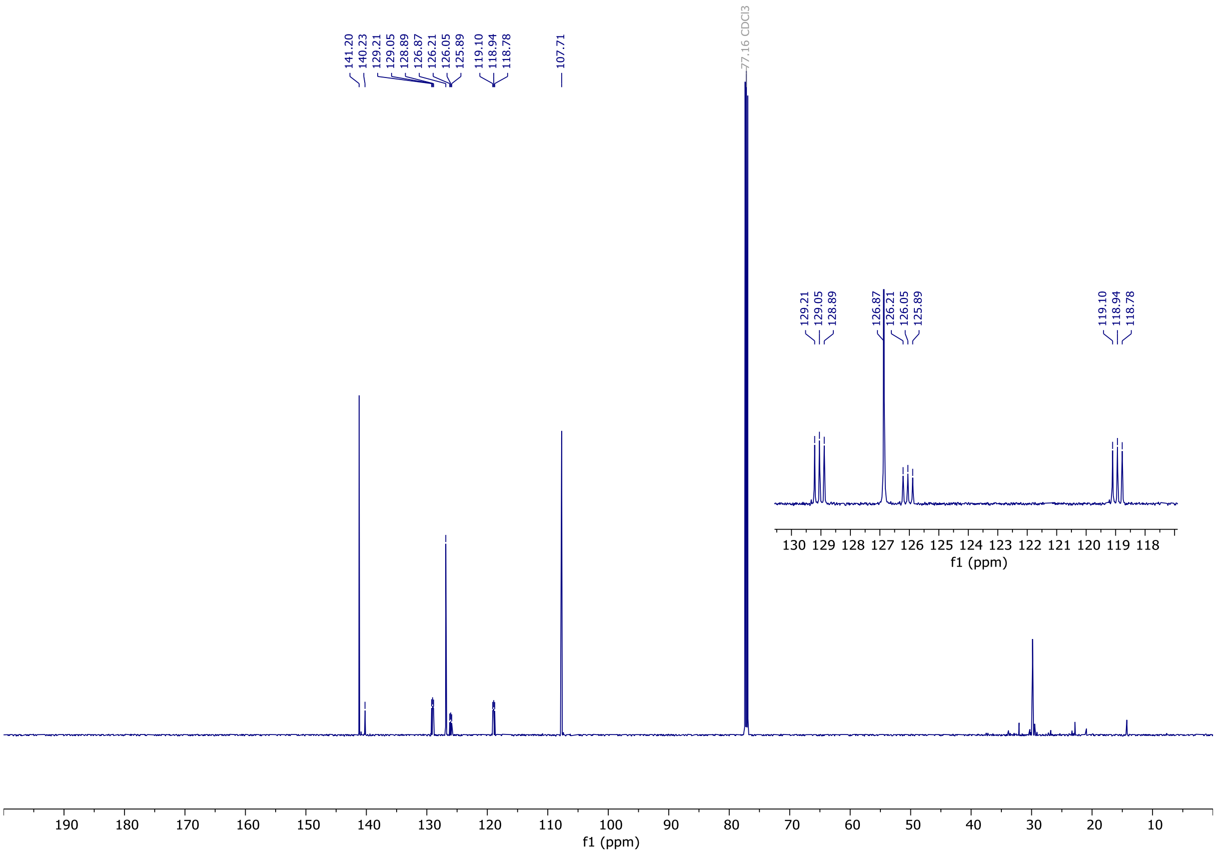

## 41b, 41b’ & 41b’’ – $^{1}$H NMR (600 MHz, $CDCl_3$)

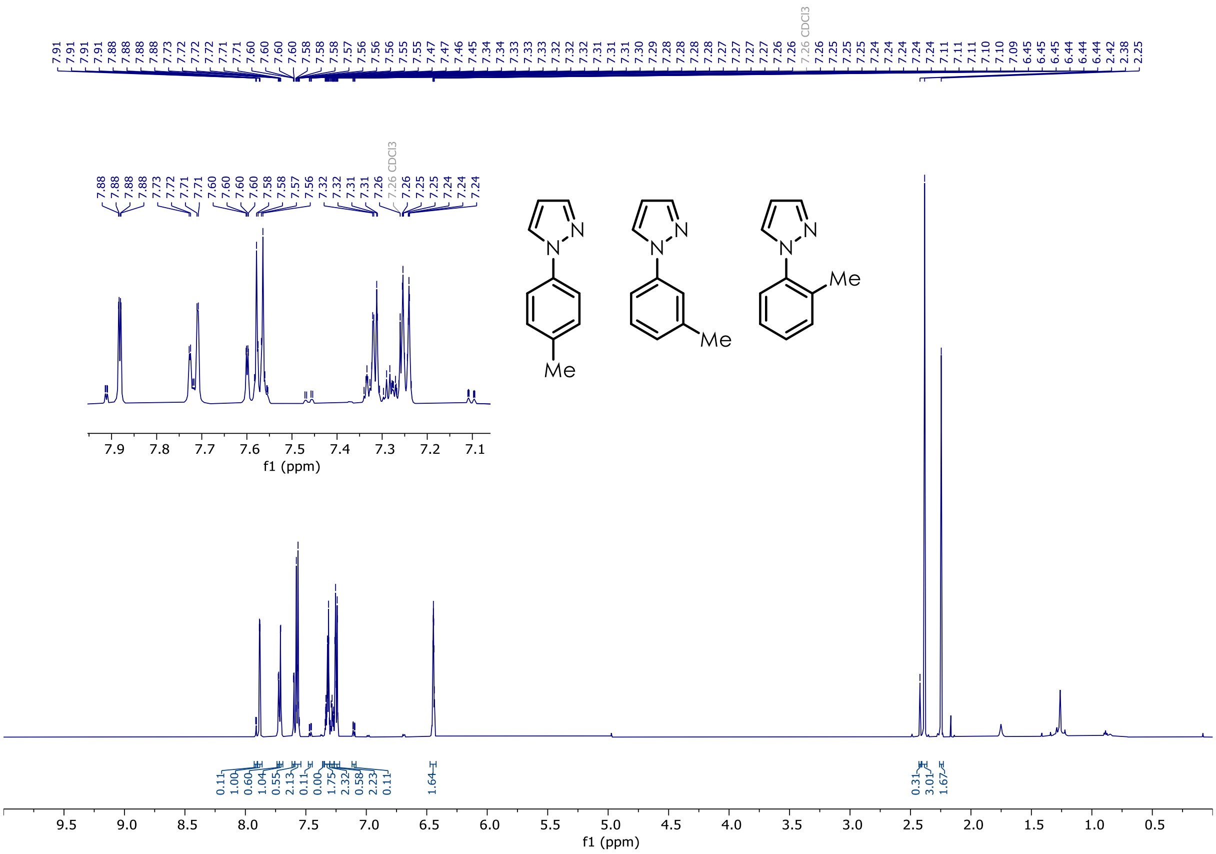


## 41b, 41b’ & 41b’’ – $^{13}$C NMR (151 MHz, $CDCl_3$)

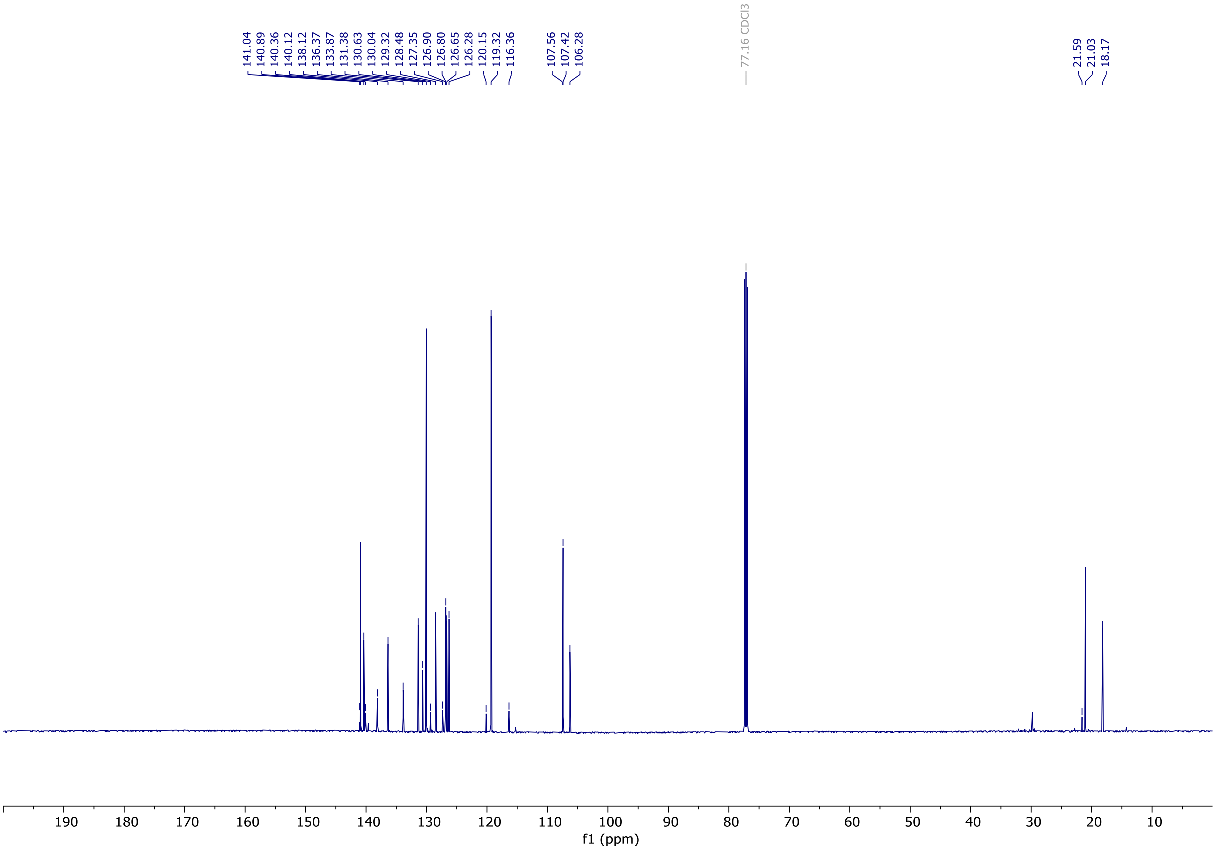

## 42b, 42b', 42b'' & 2b – ¹H NMR (600 MHz, $CDCl_3$)

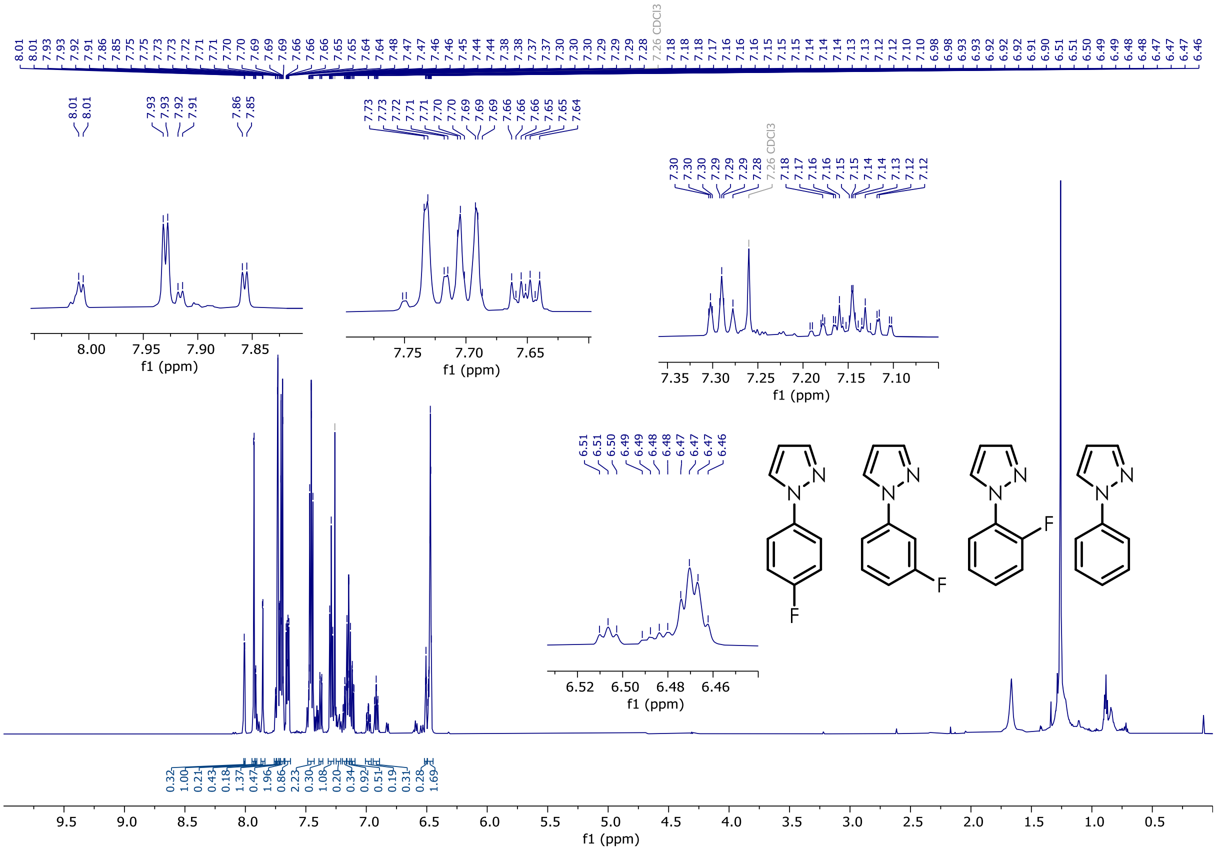

## 42b, 42b', 42b'' & 2b – ¹³C NMR (151 MHz, $CDCl_3$)

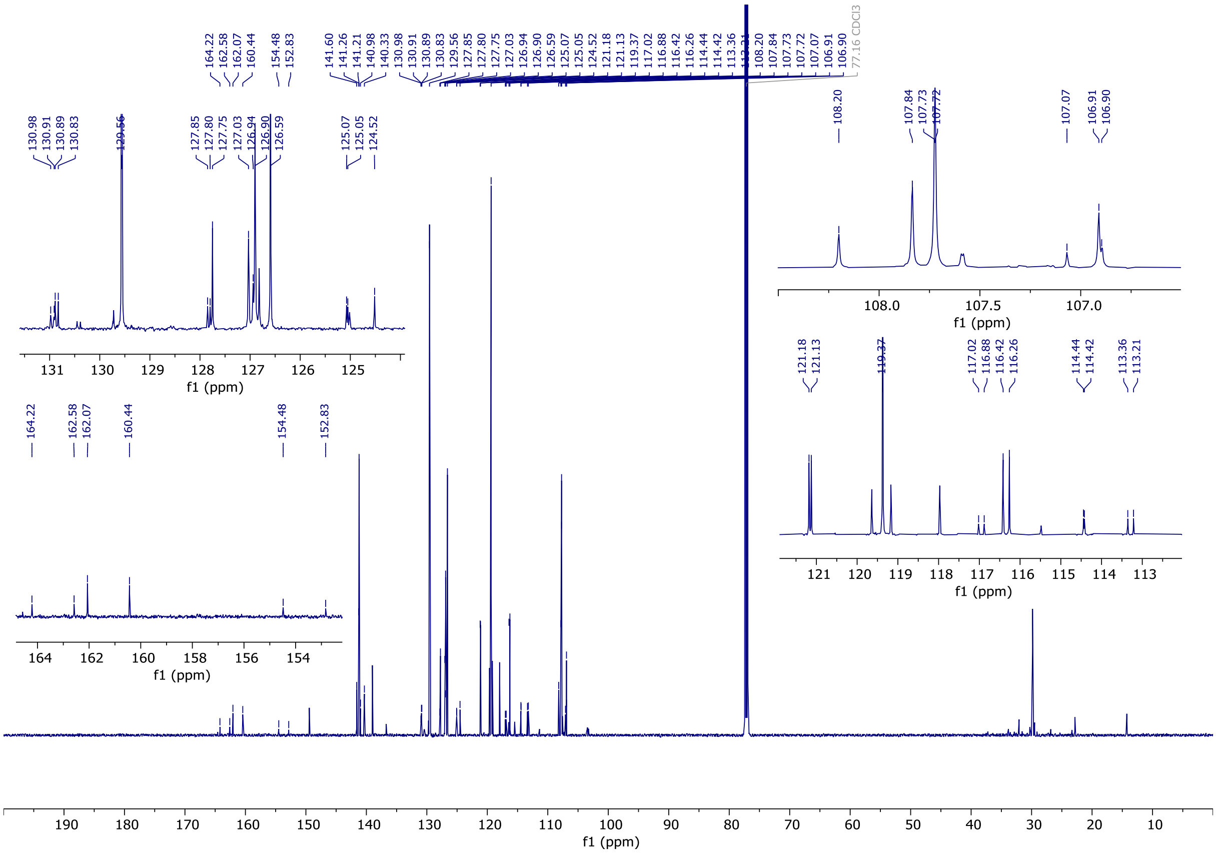

**42b, 42b', 42b'' & 2b – $^{19}$F NMR (565 MHz, CDCl$_3$)**

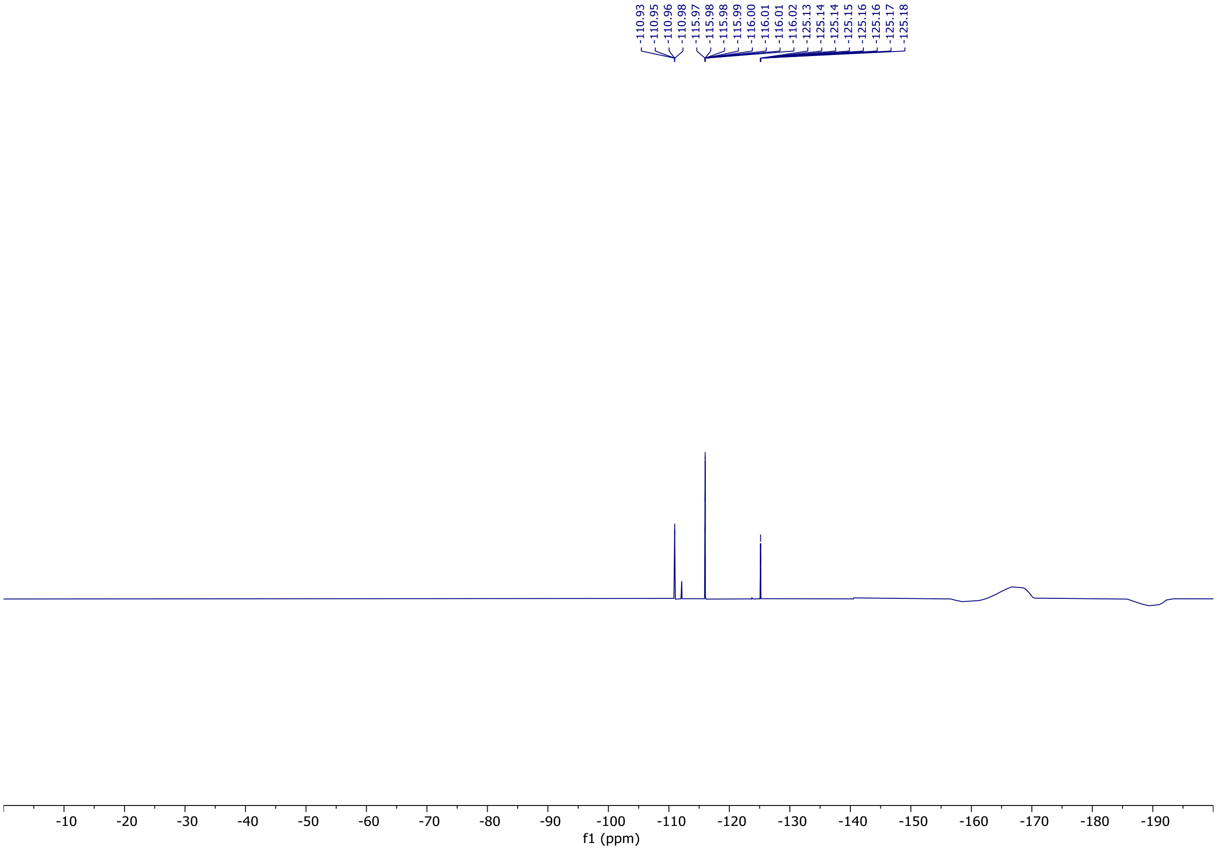

## 42c – ¹H NMR (600 MHz, $CDCl_3$)

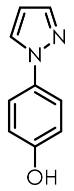

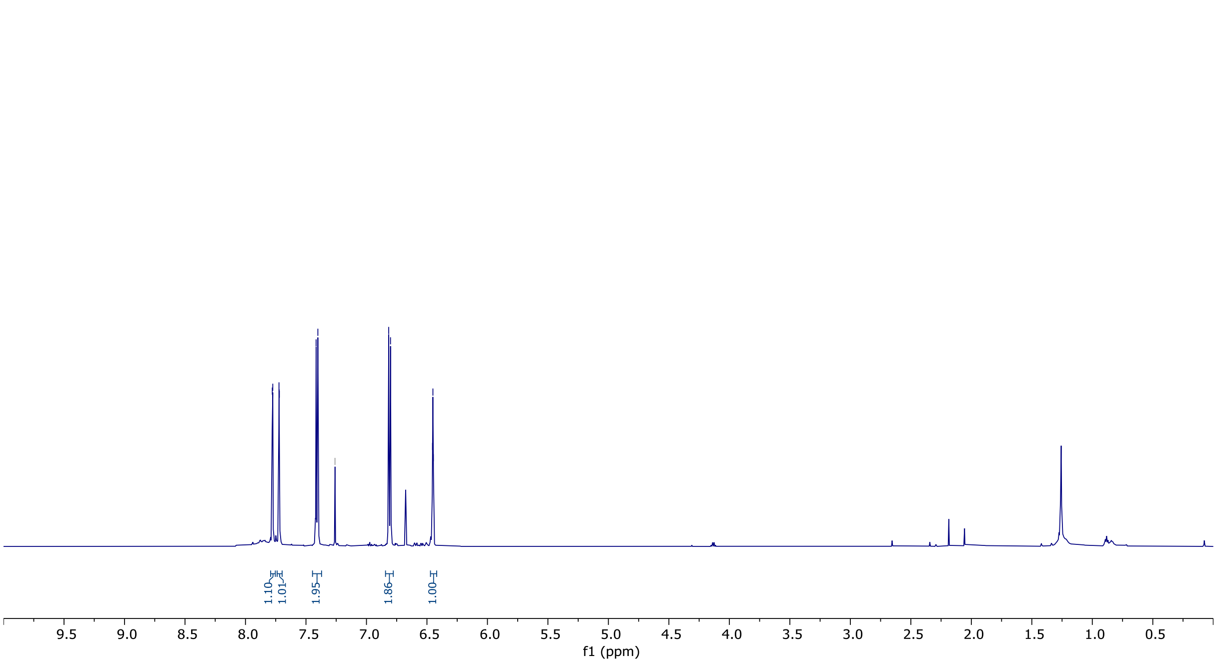

## 42c – ¹³C NMR (151 MHz, $CDCl_3$)

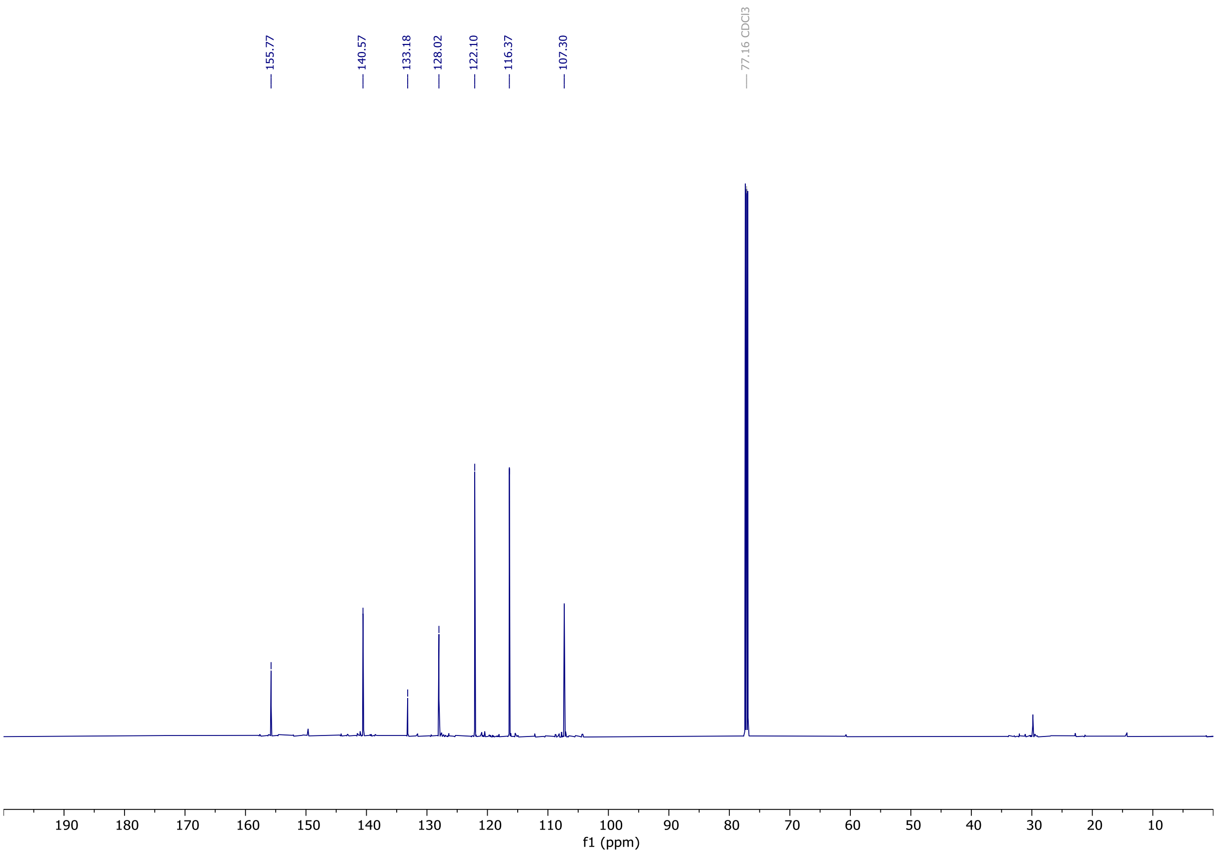

## 43b & 43b' – $^{1}$H NMR (600 MHz, CDCl$_3$)

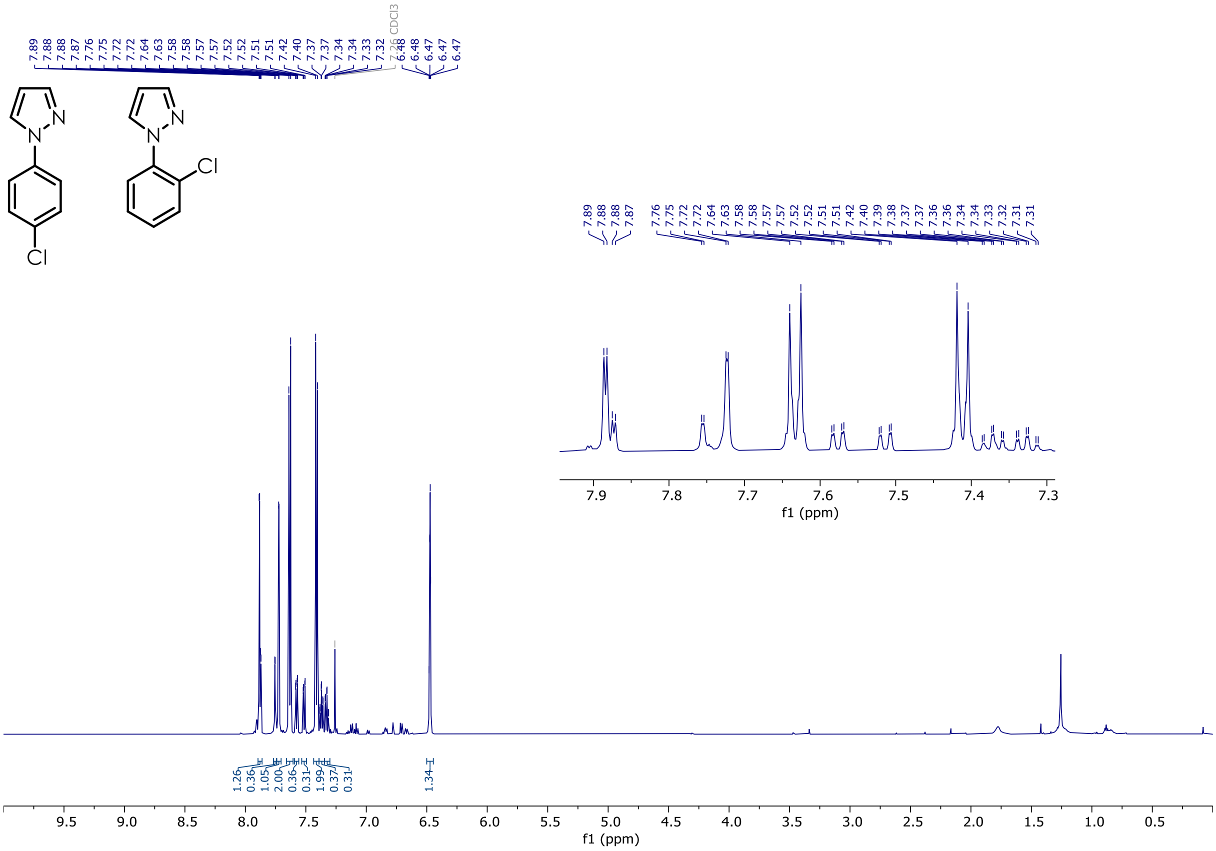


## 43b & 43b' – $^{13}$C NMR (151 MHz, CDCl$_3$)

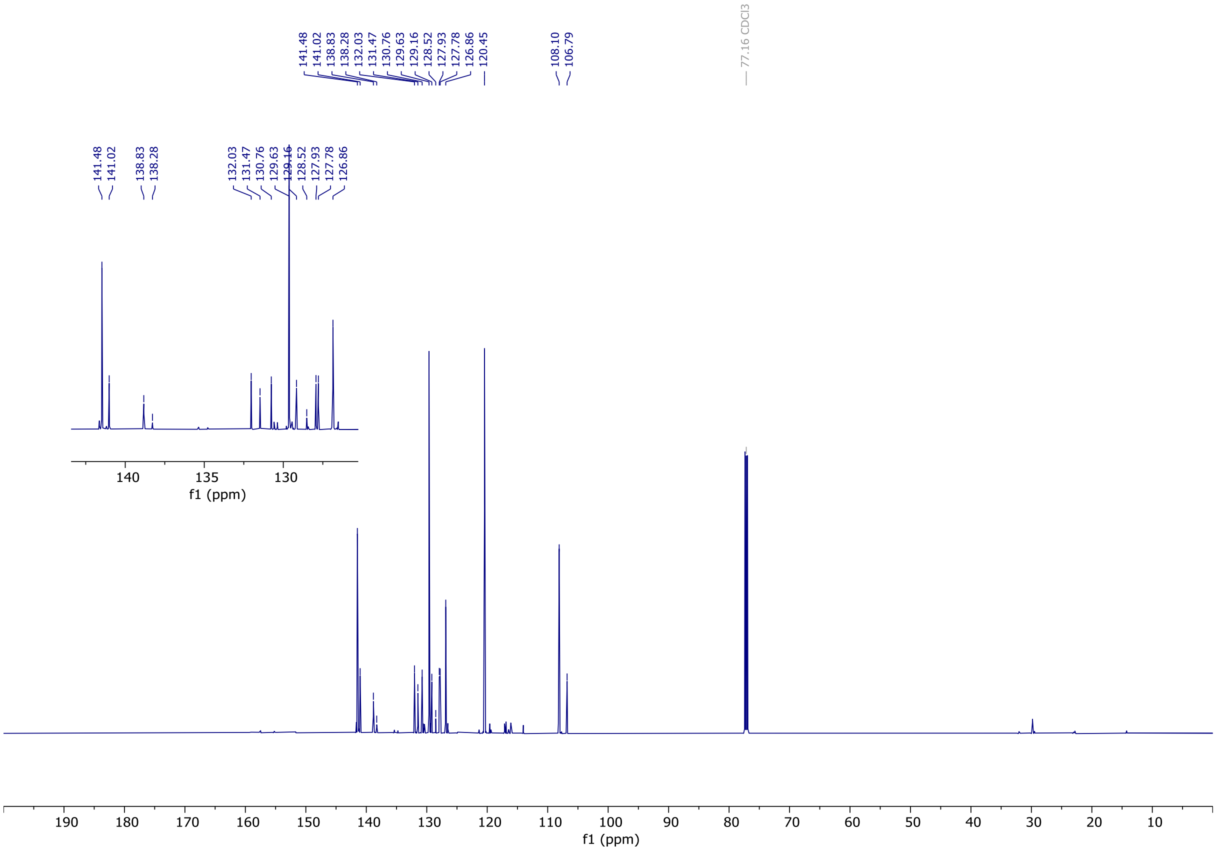

## 44b & 44b' – $^{1}$H NMR (600 MHz, $CDCl_3$)

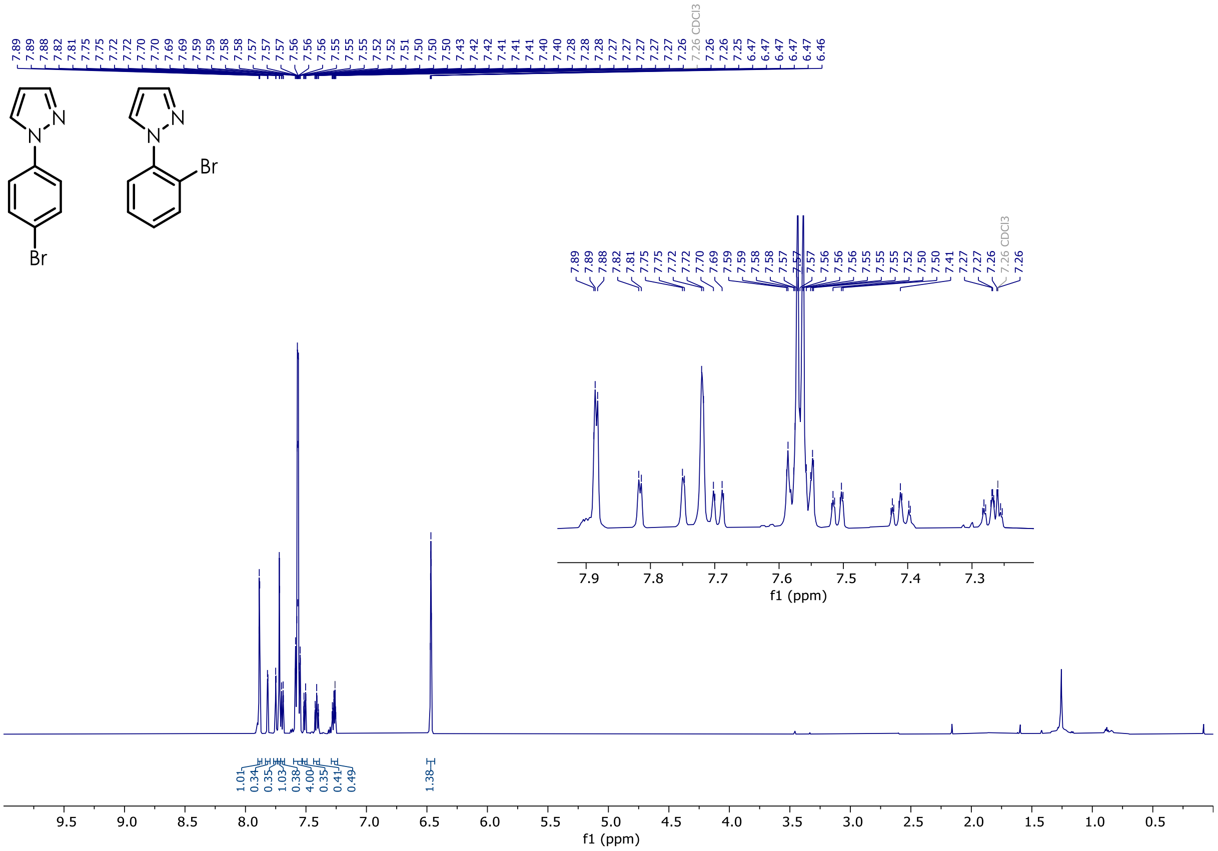

## 44b & 44b' – $^{13}$C NMR (151 MHz, $CDCl_3$)

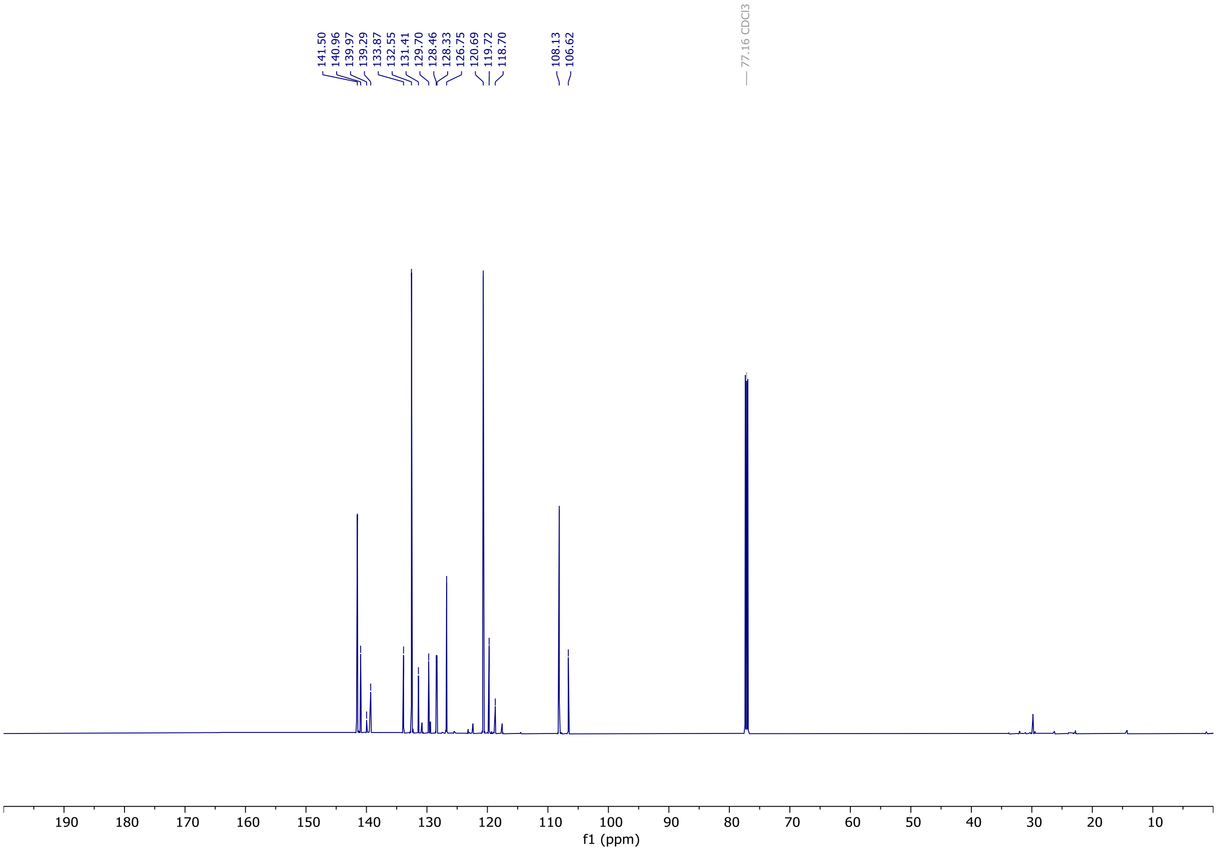

**45b & 45b' – $^{1}$H NMR (600 MHz, CDCl$_3$, *crude*)**

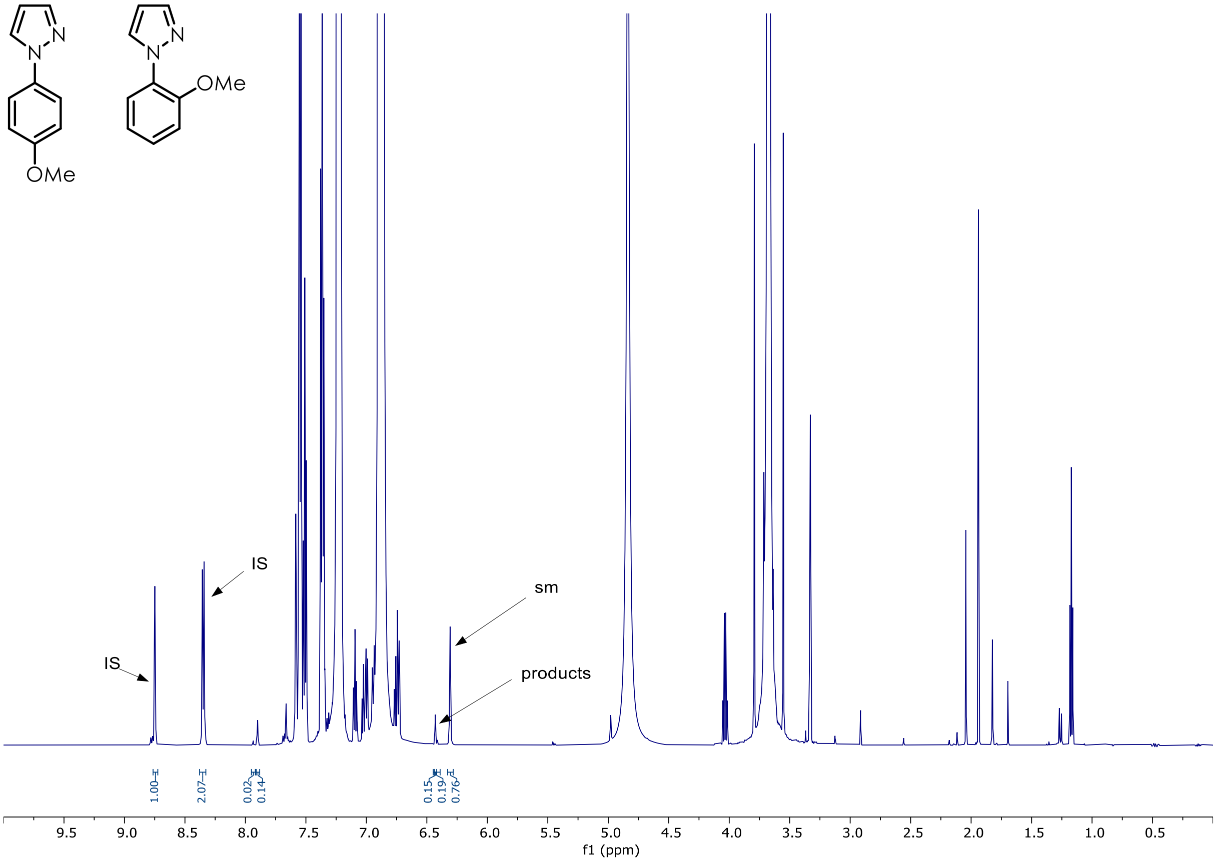

## 48b & 48b' – $^{1}$H NMR (600 MHz, CDCl$_3$)

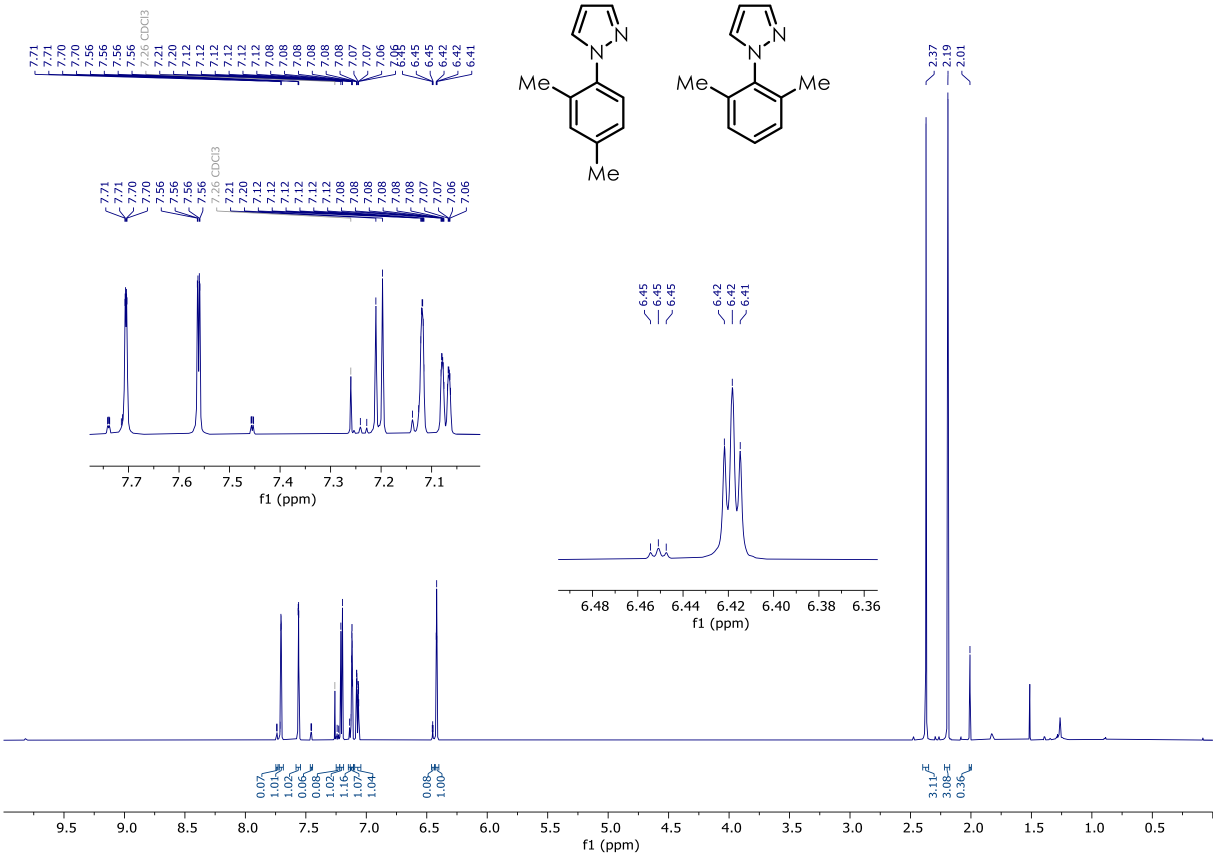


## 48b & 48b' – $^{13}$C NMR (151 MHz, CDCl$_3$)

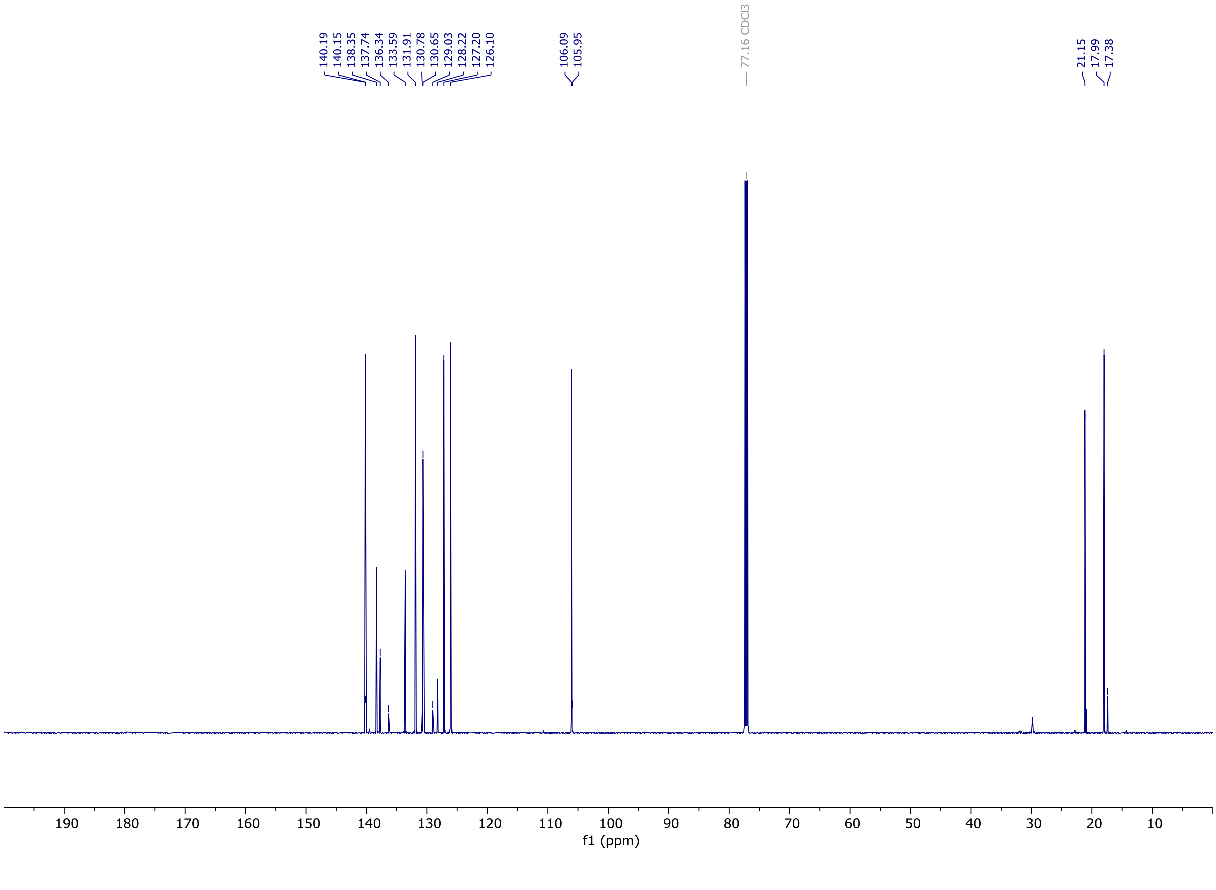

# 49b – $^1$H NMR (600 MHz, $CDCl_3$)

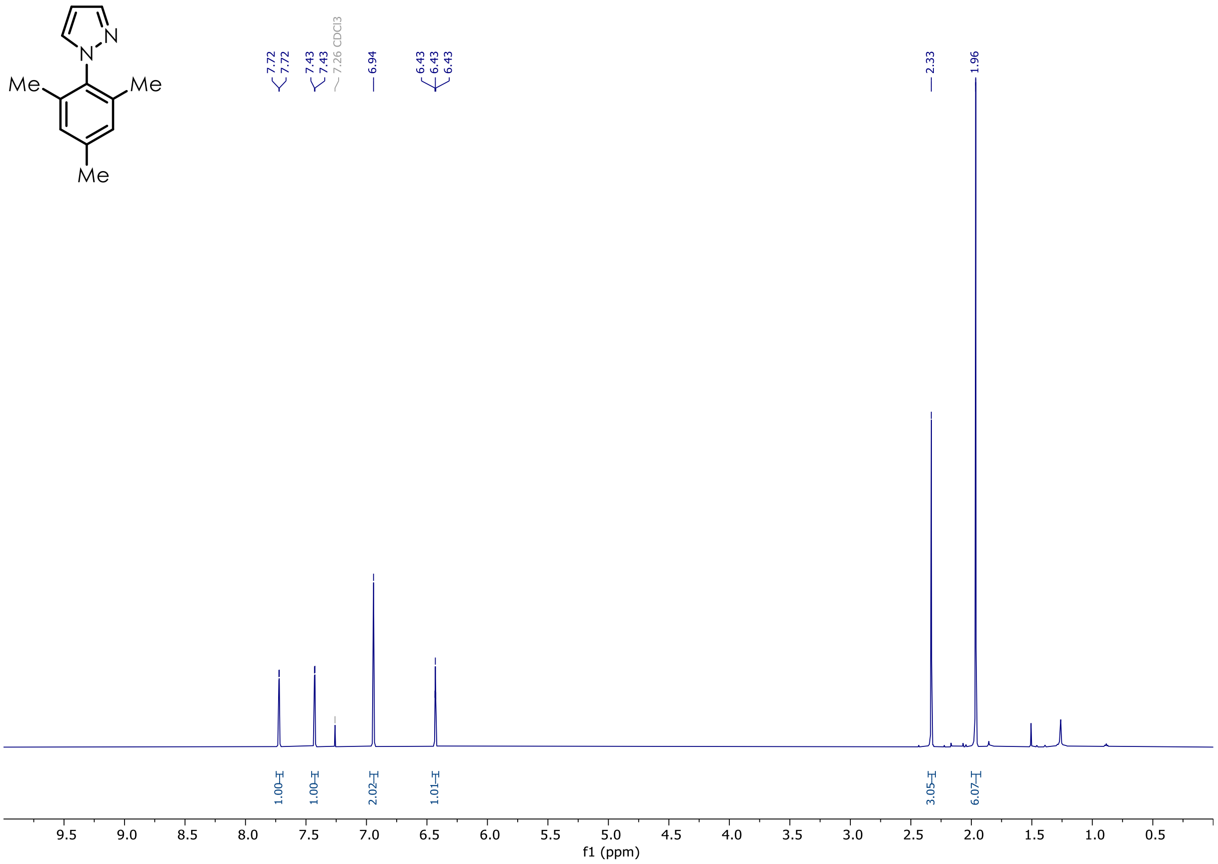


# 49b – $^{13}$C NMR (151 MHz, $CDCl_3$)

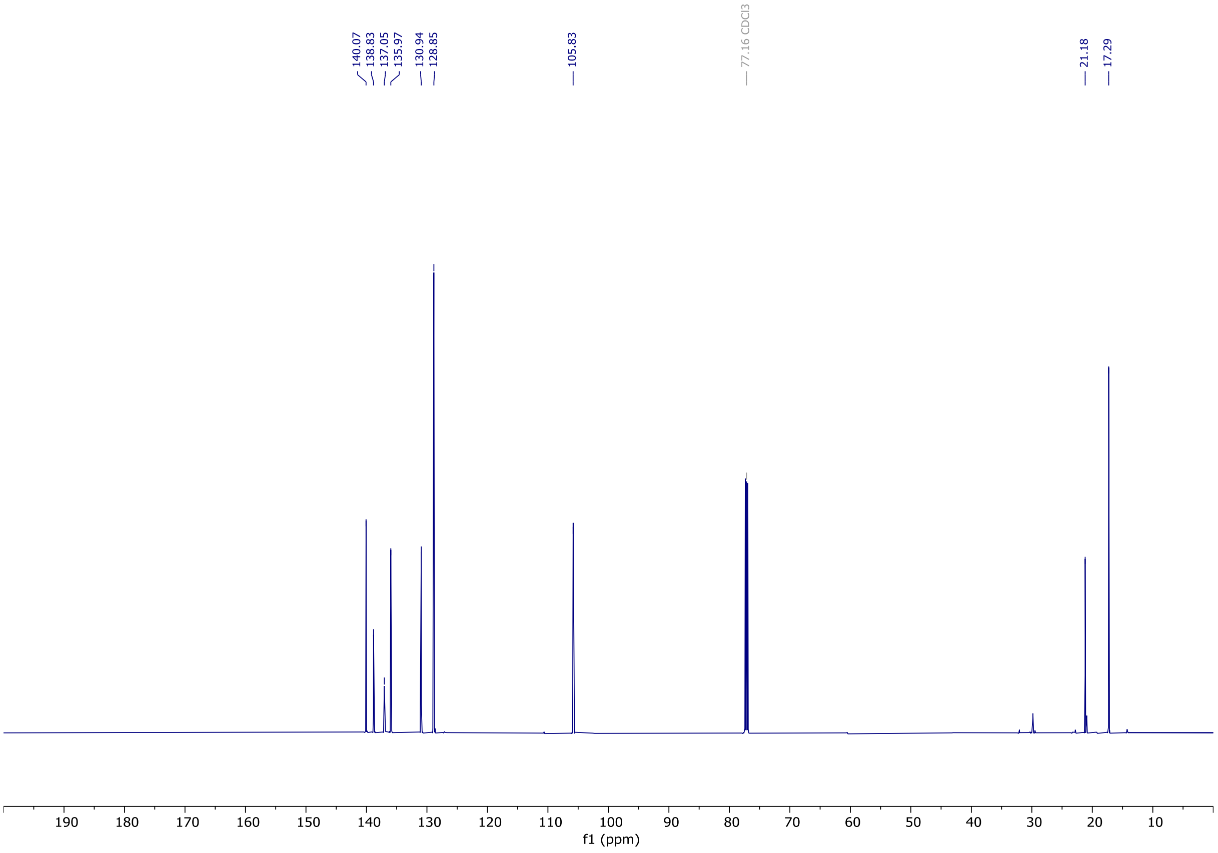

## 50b – $^{1}$H NMR (600 MHz, CDCl$_3$)

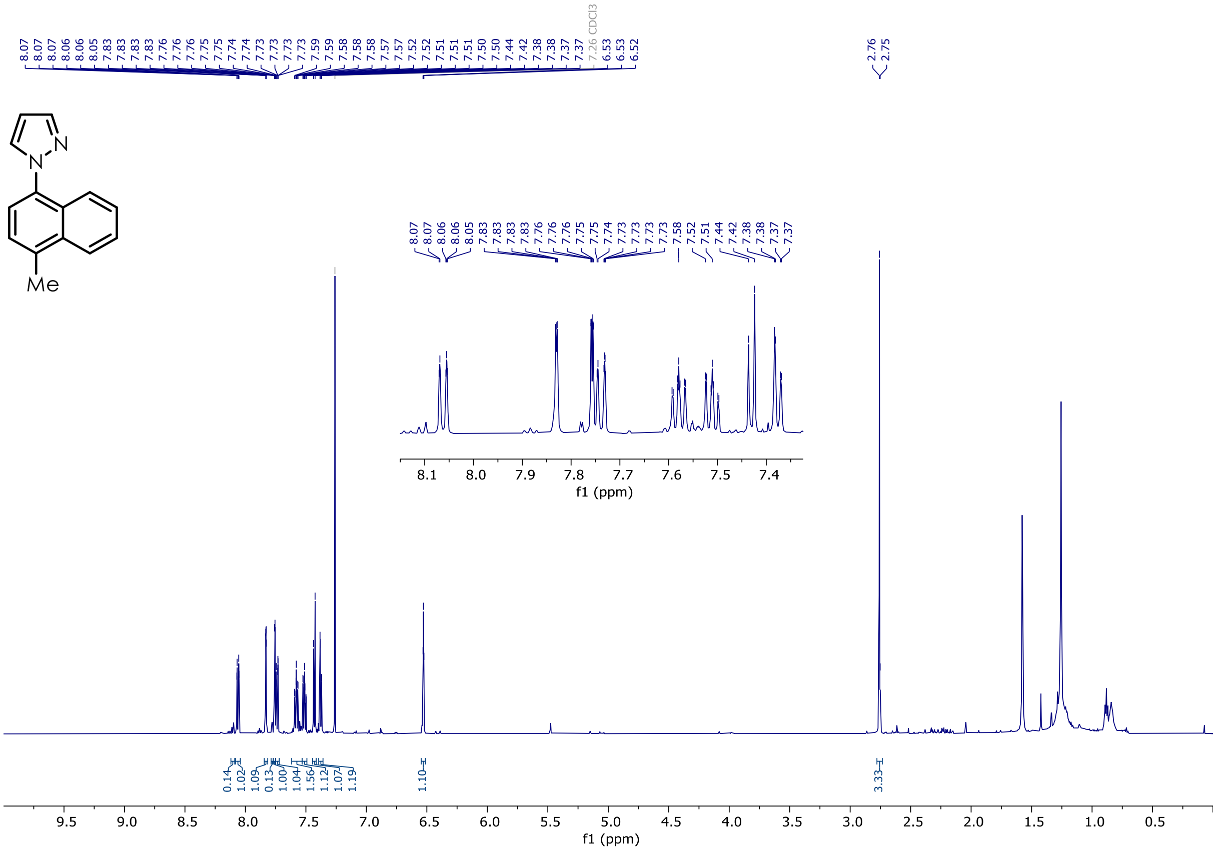

## 50b – $^{13}$C NMR (151 MHz, CDCl$_3$)

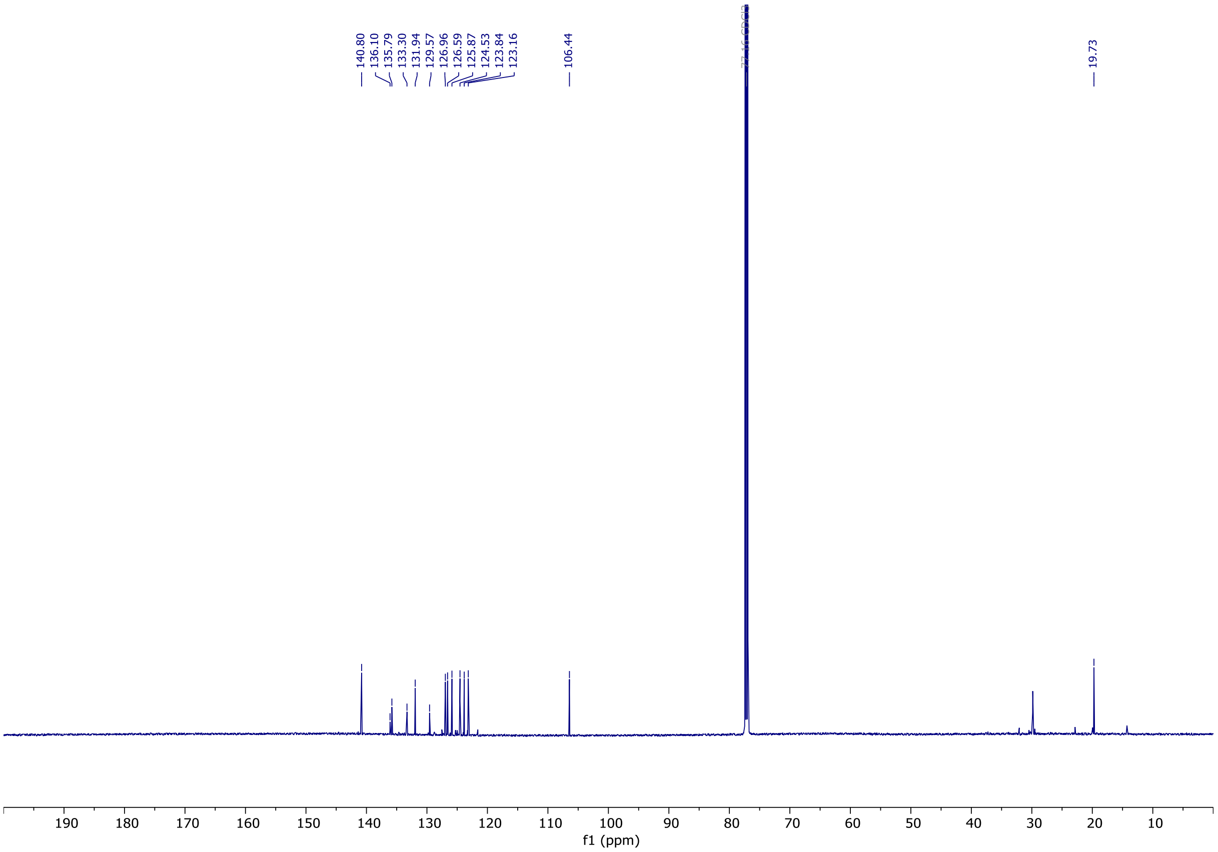

## 51b – $^{1}$H NMR (600 MHz, CDCl$_3$)

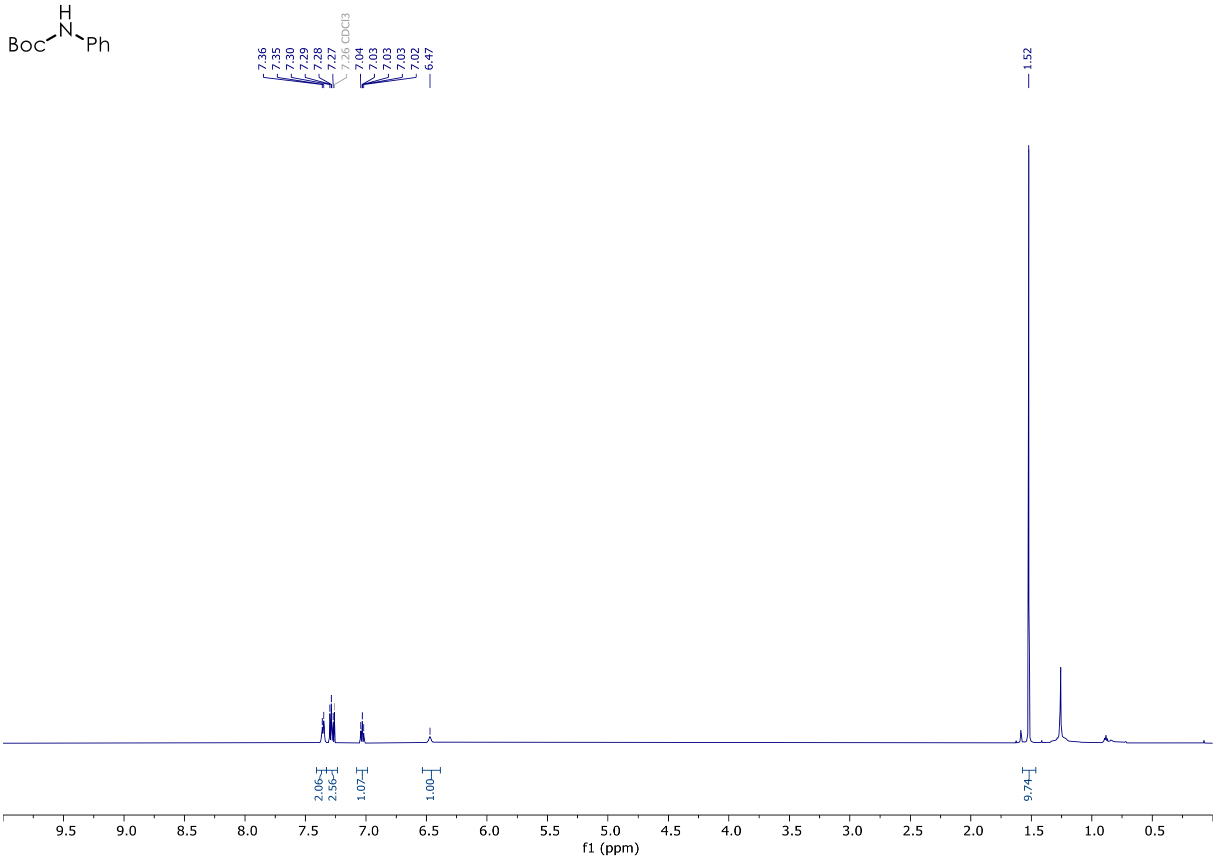


## 51b – $^{13}$C NMR (151 MHz, CDCl$_3$)

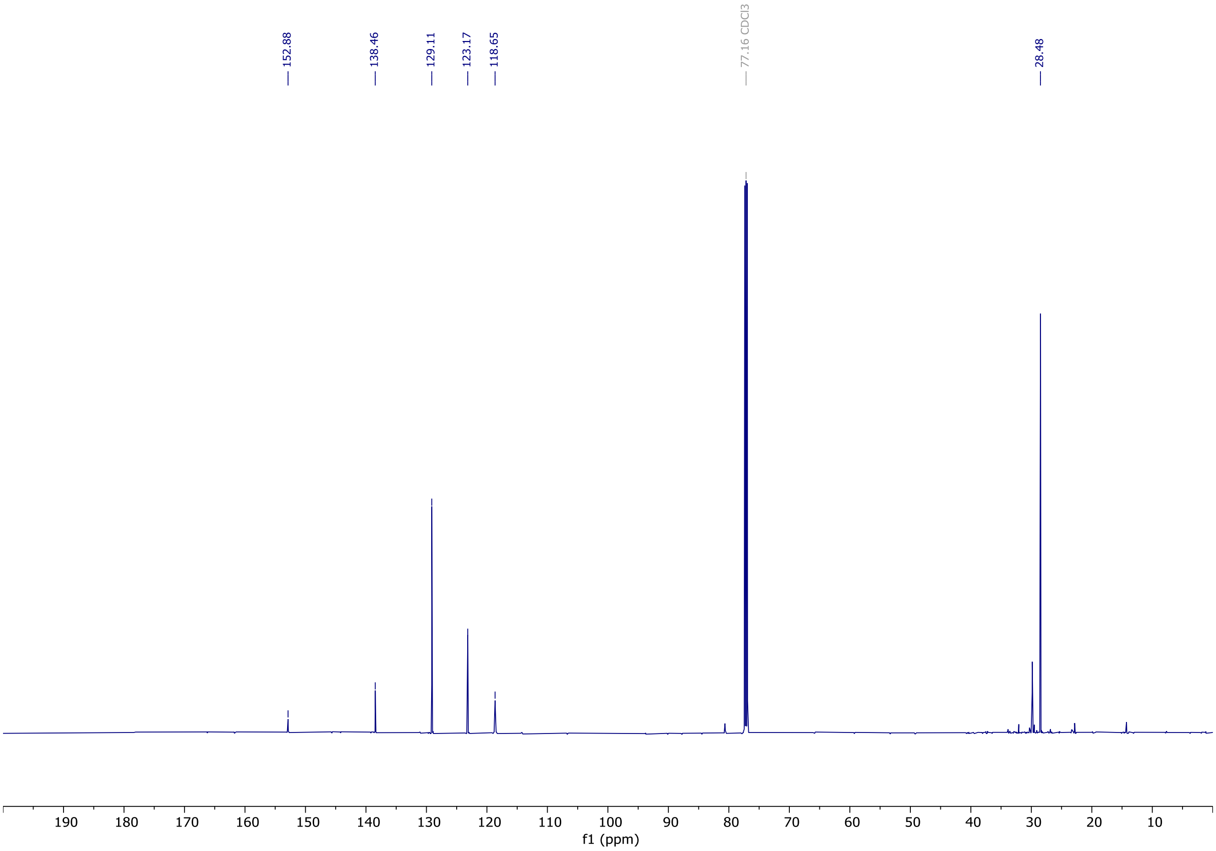

## 52b – ¹H NMR (600 MHz, MeOD, *crude*)

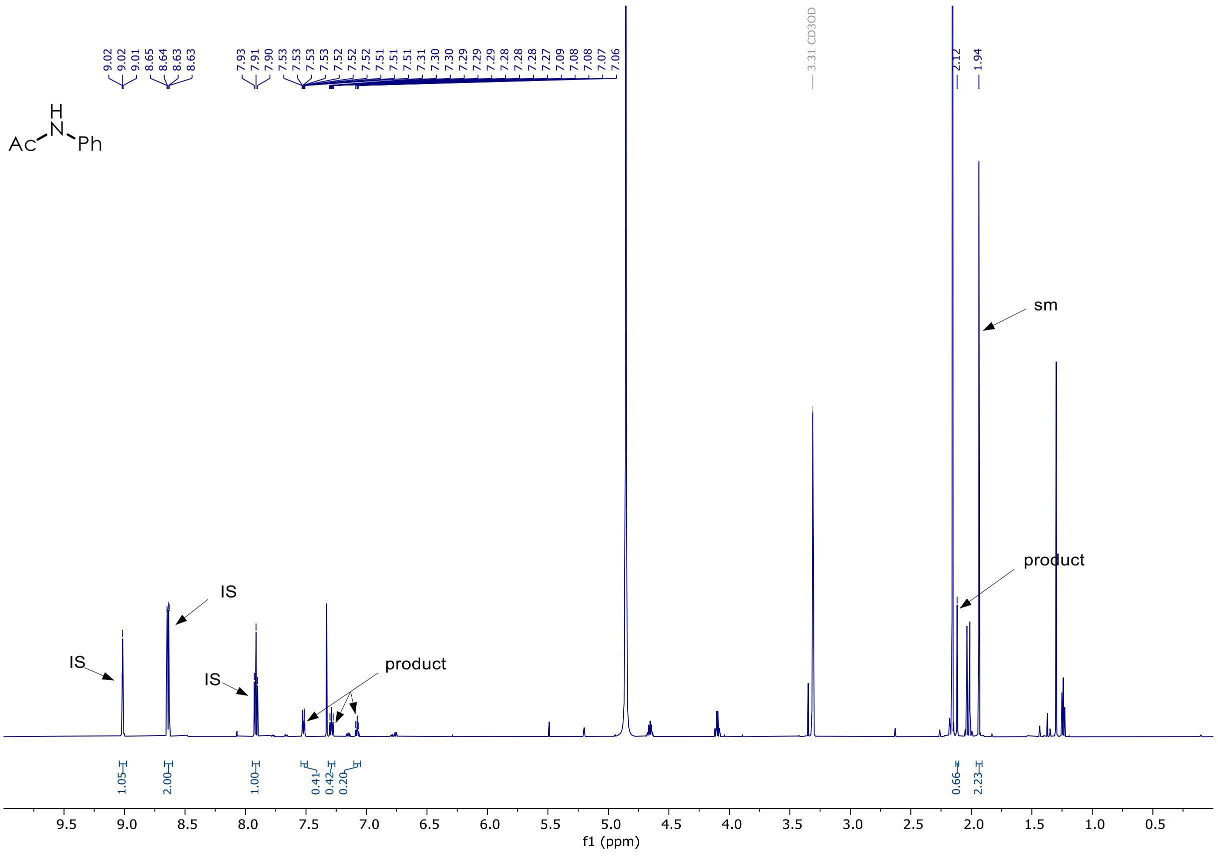

## S1b – $^{1}$H NMR (600 MHz, $CDCl_3$)

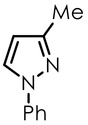


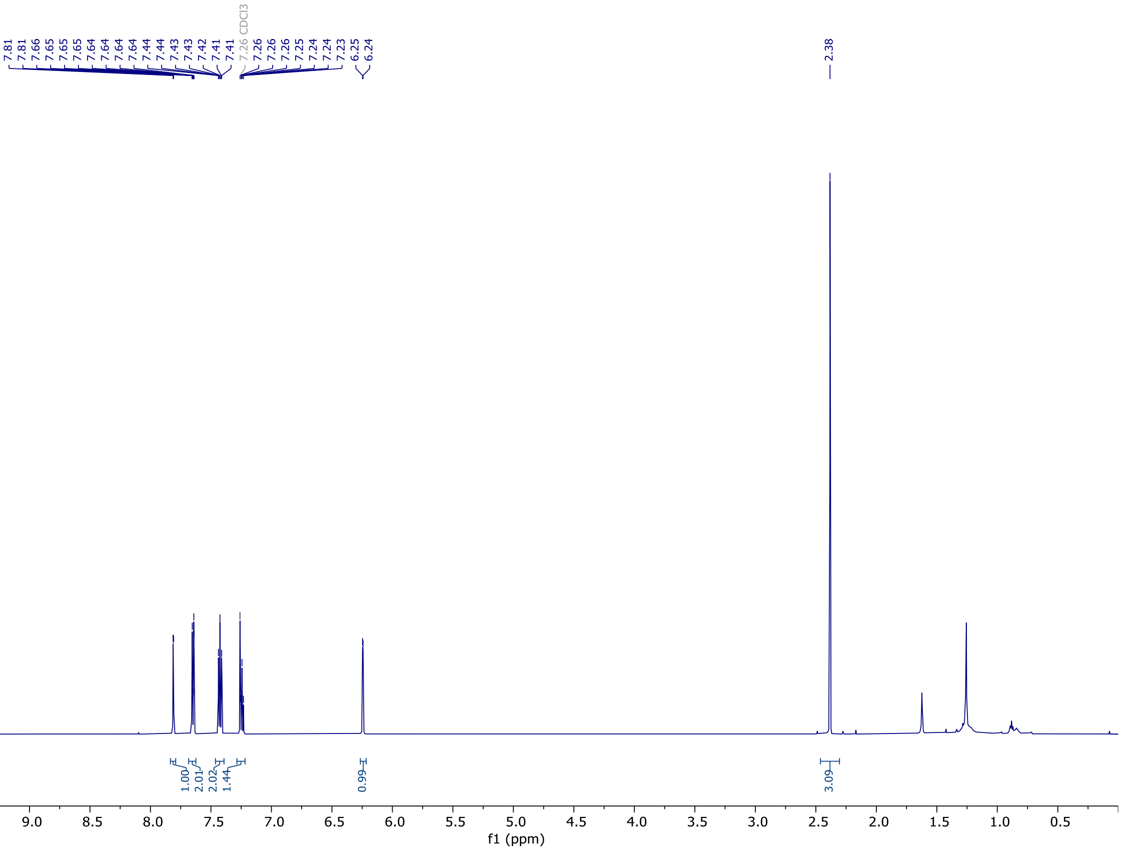


## S1b – $^{13}$C NMR (151 MHz, $CDCl_3$)

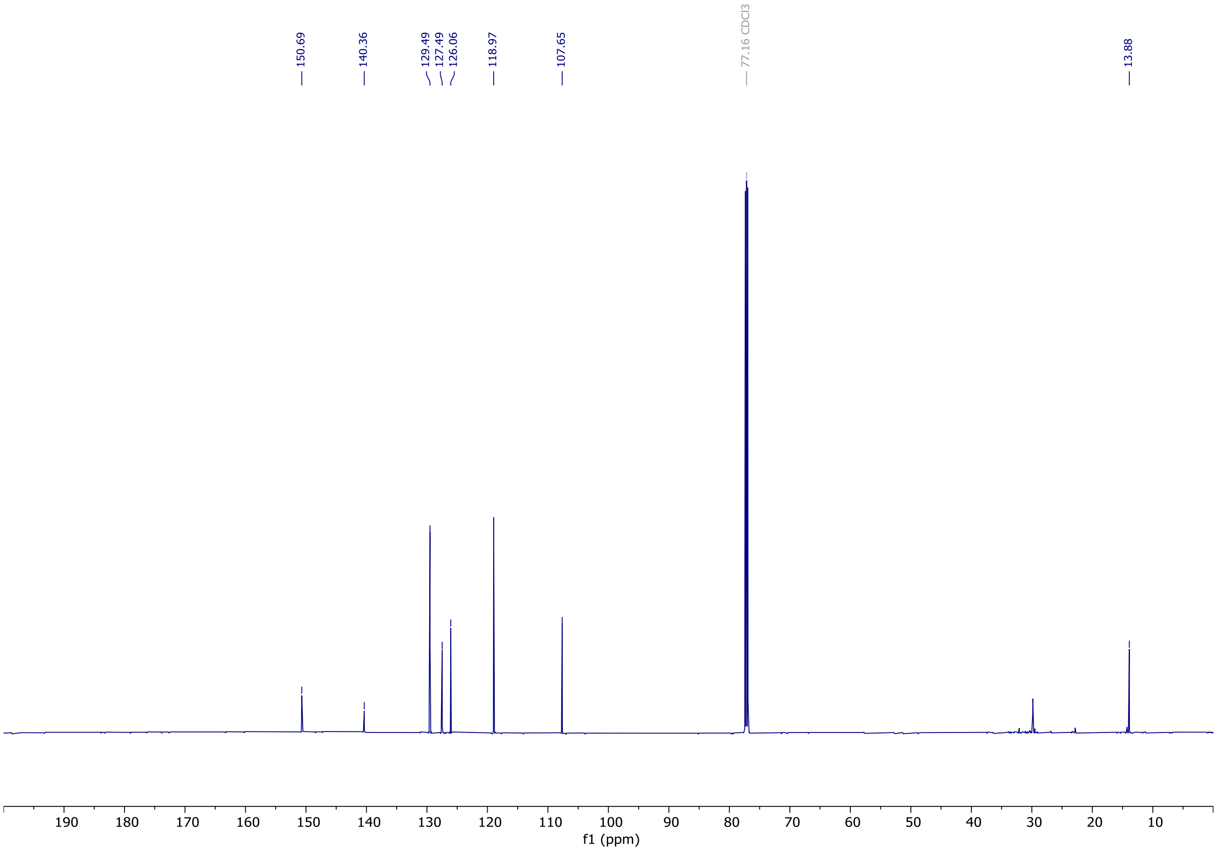

## S1b' – $^{1}$H NMR (600 MHz, $CDCl_3$)

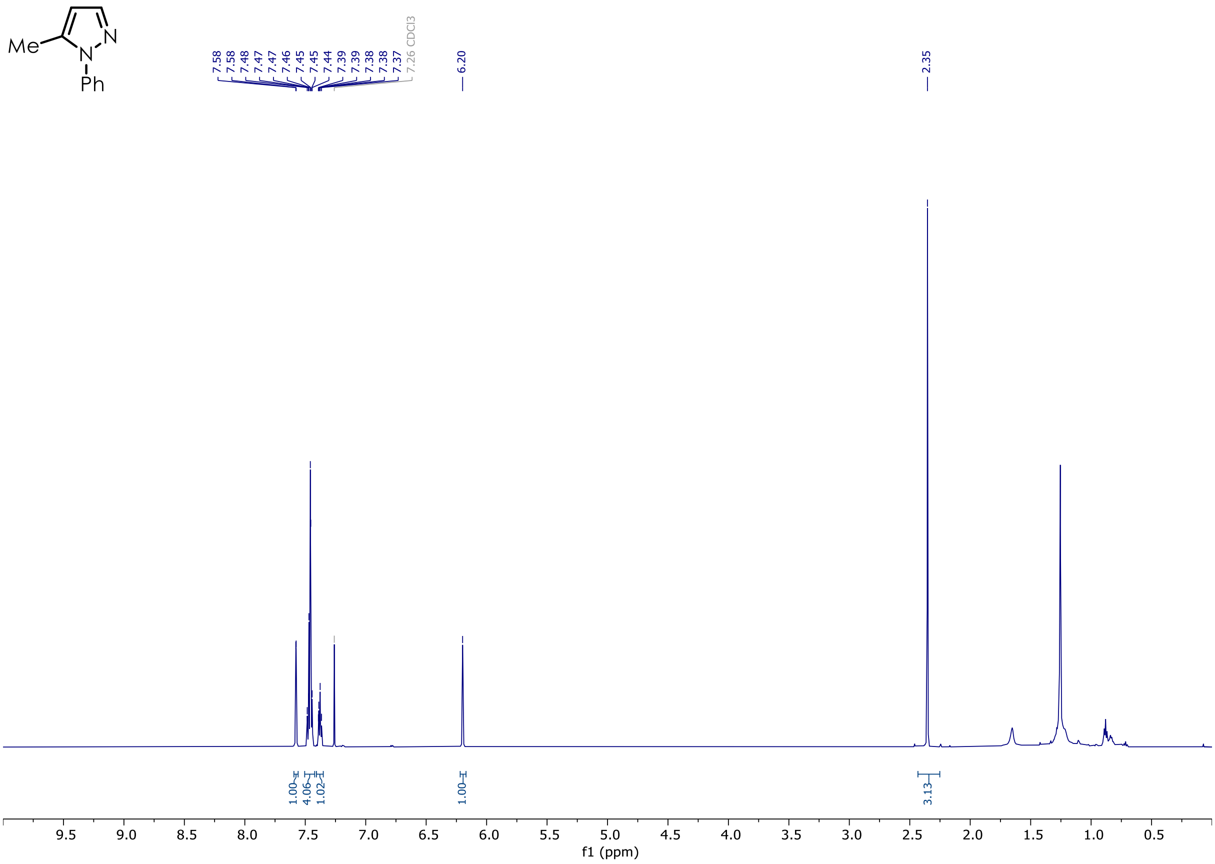


## S1b' – $^{13}$C NMR (151 MHz, $CDCl_3$)

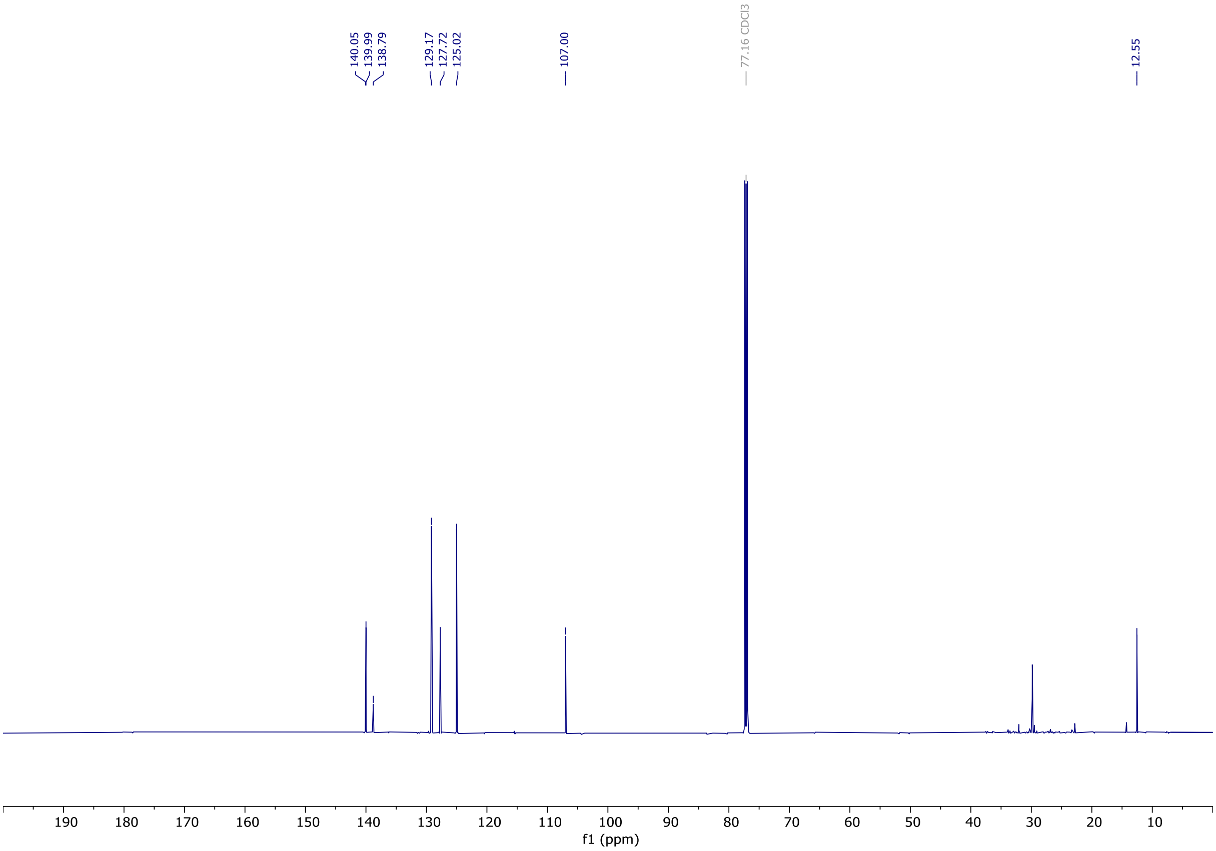

## S2b – $^{1}H$ NMR (600 MHz, $CDCl_3$)

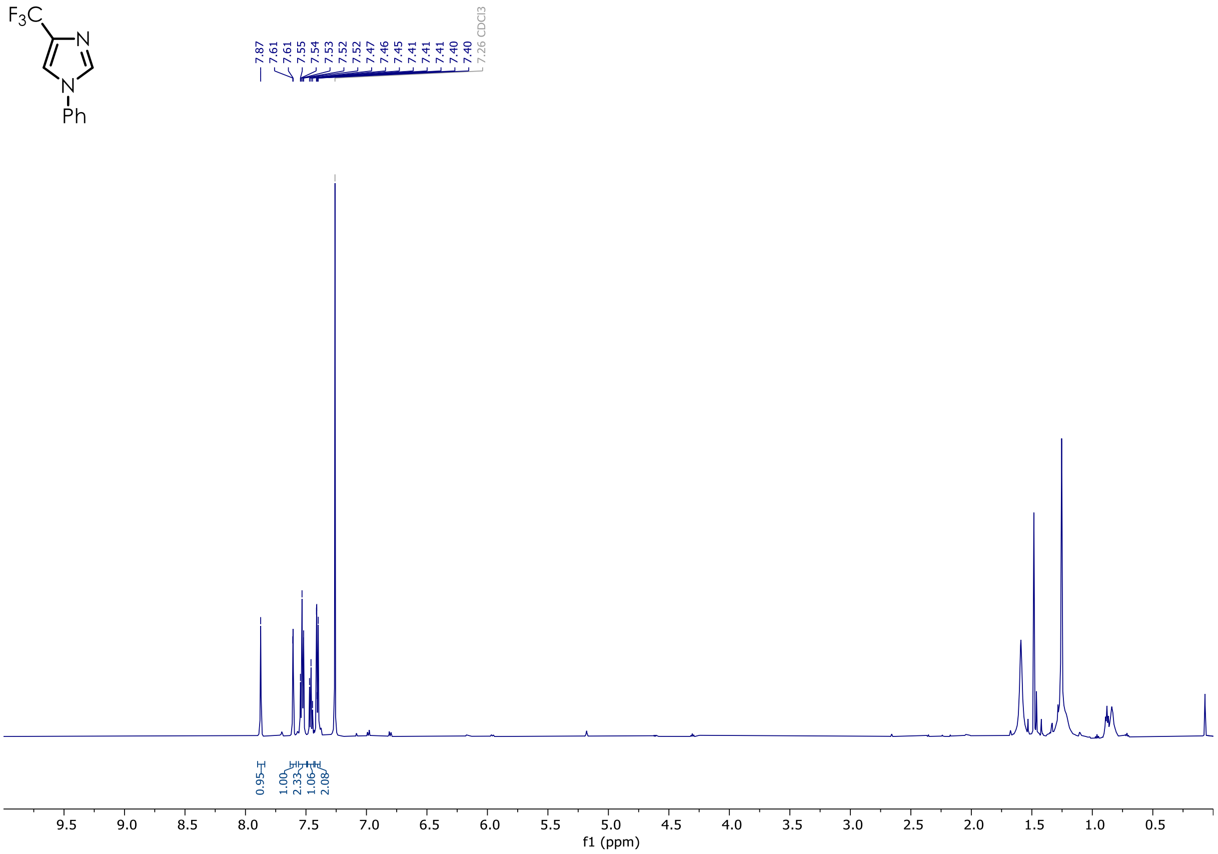


## S2b – $^{13}C$ NMR (151 MHz, $CDCl_3$)

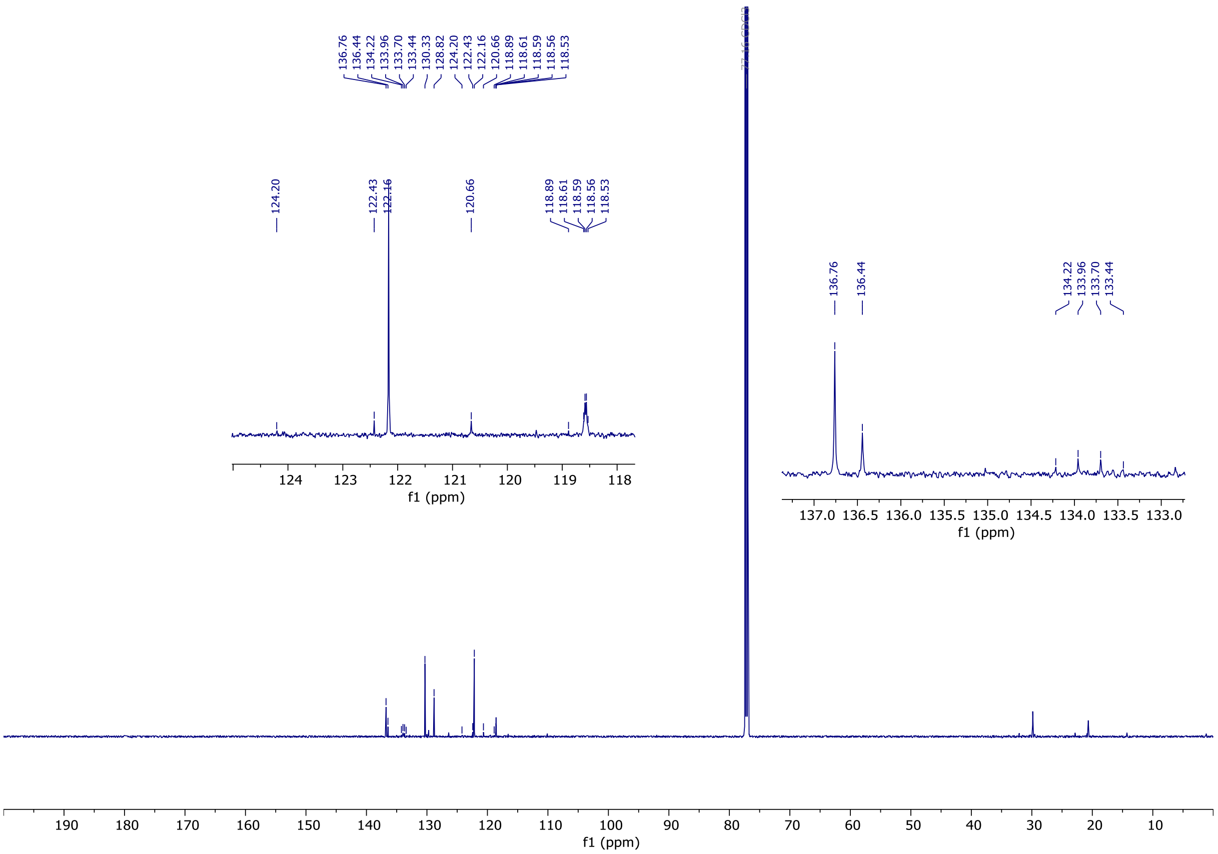

## S3b – $^{1}H$ NMR (600 MHz, $CDCl_3$)

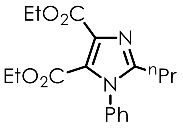


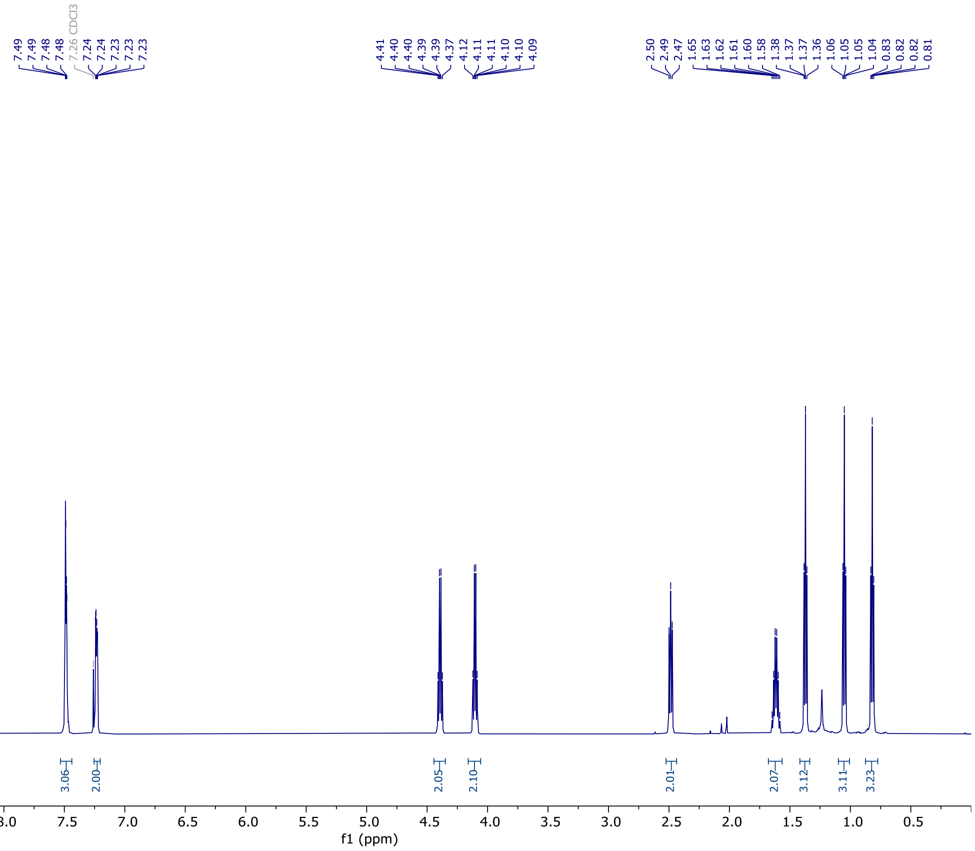


## S3b – $^{13}C$ NMR (151 MHz, $CDCl_3$)

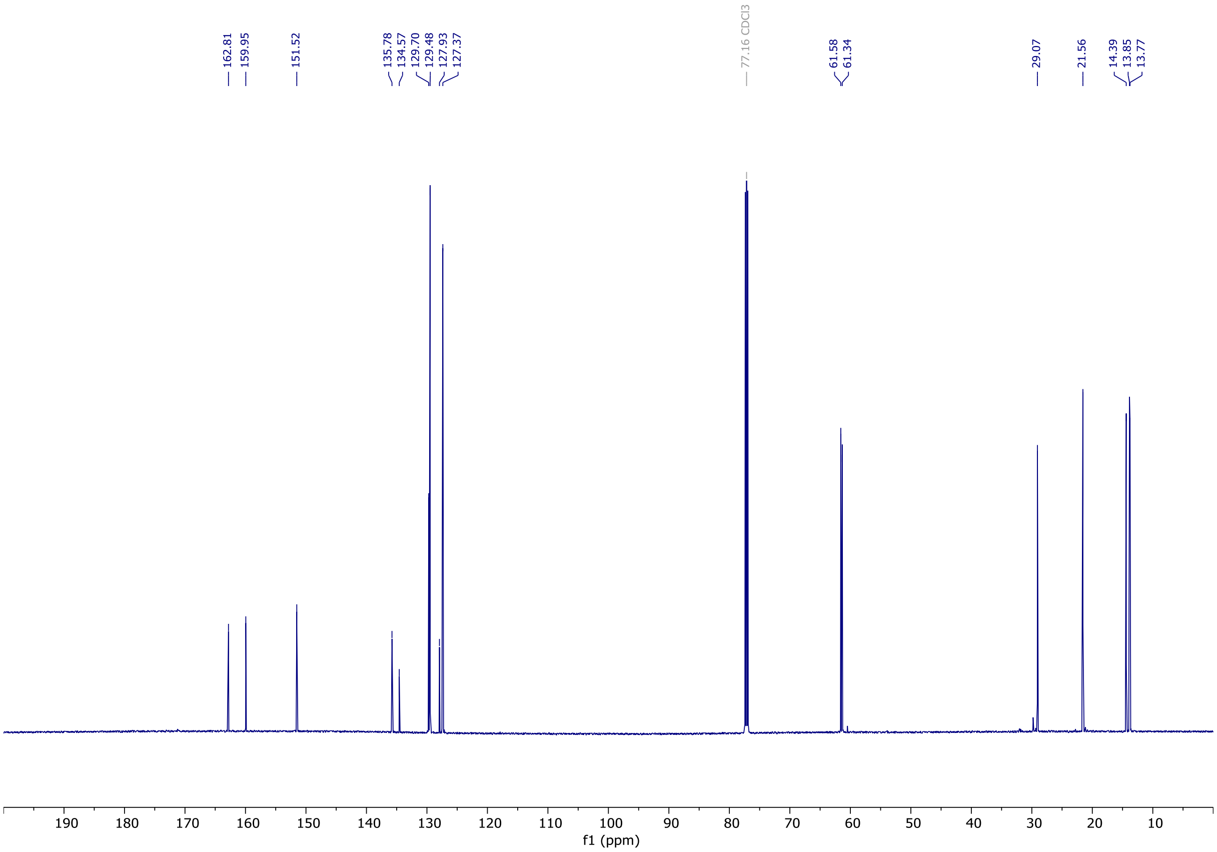

## 2a-$d_1$ – $^1$H NMR (600 MHz, $CDCl_3$)

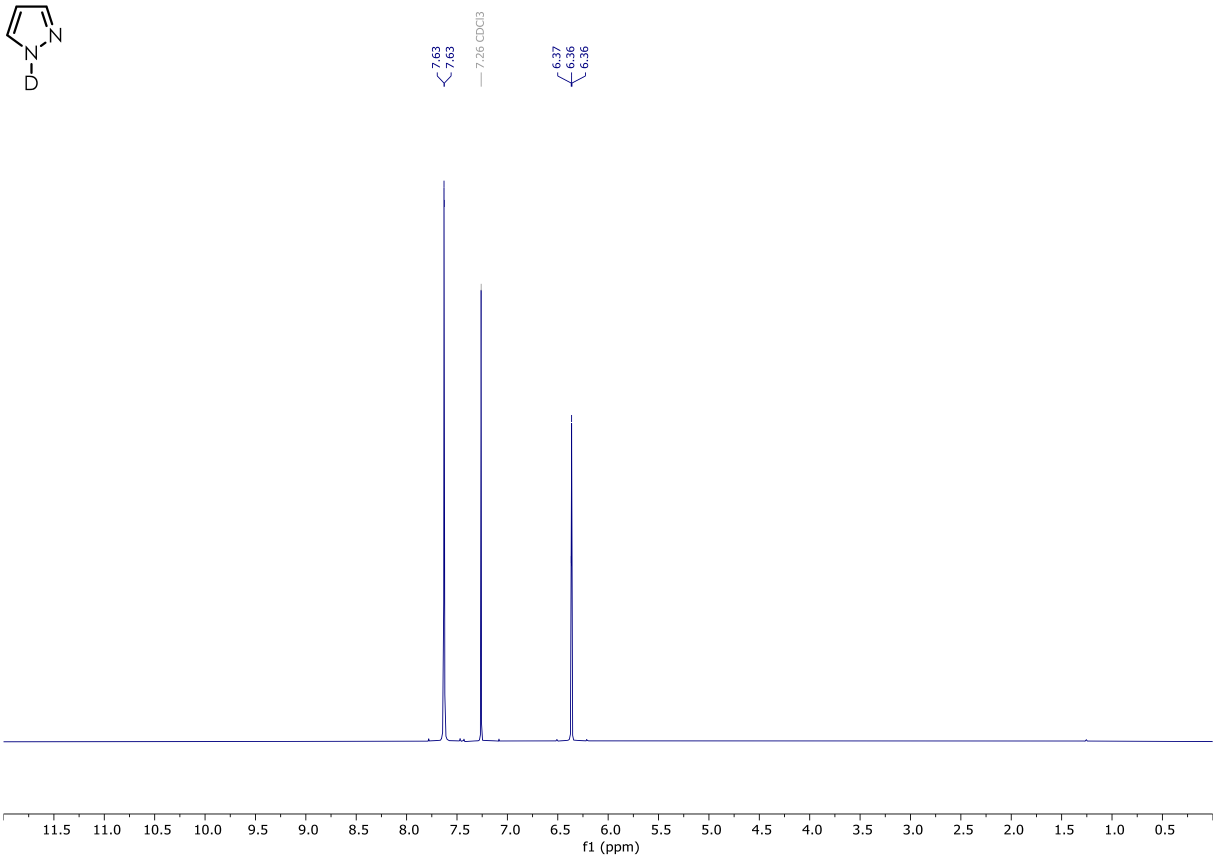


## 2a-$d_1$ – $^2$H NMR (92 MHz, $CHCl_3$)

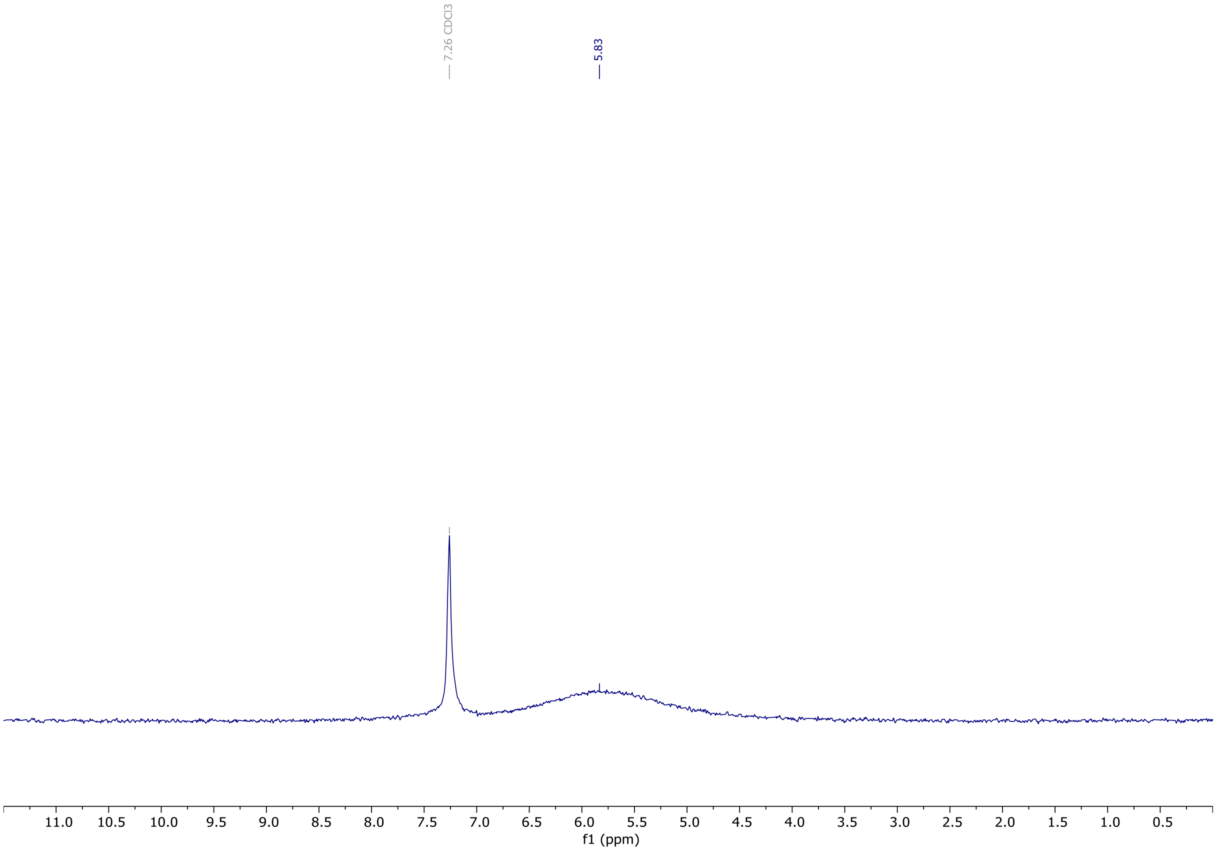

## 16 References


1 T. M. Masson, S. D. A. Zondag, J. H. A. Schuurmans and T. Noël, *React. Chem. Eng.*, 2024, **9**, 2218–2225.

2 R. Hein, C. N. Stindt and B. L. Feringa, *J. Am. Chem. Soc.*, 2024, **146**, 26275–26285.

3 S. C. Sau, M. Schmitz, C. Burdenski, M. Baumert, P. W. Antoni, C. Kerzig and M. M. Hansmann, *J. Am. Chem. Soc.*, 2024, **146**, 3416–3426.

4 S. S. Rana and J. Choudhury, *J. Am. Chem. Soc.*, 2024, **146**, 3603–3608.

5 P. P. Sen and S. R. Roy, *Org. Lett.*, 2023, **25**, 1895–1900.

6 M. R. Lasky, E.-C. Liu, M. S. Remy and M. S. Sanford, *J. Am. Chem. Soc.*, 2024, **146**, 14799–14806.

7 Y. Ai, S. Sakamuru, G. Imler, M. Xia and F. Xue, *Bioorg. Med. Chem.*, 2022, **69**, 116890.

8 J. J. Neumann, M. Suri and F. Glorius, *Angew. Chem. Int. Ed.*, 2010, **49**, 7790–7794.

9 V. A. Chertkov, A. K. Shestakova and D. V. Davydov, *Chem. Heterocycl. Compd.*, 2011, **47**, 45–54.

10 Y. Xie, L. Lin and B. Xu, *Green Chem.*, 2024, **26**, 5173–5177.

11 P. G. Dalai, K. Palit and N. Panda, *Adv. Synth. Catal.*, 2022, **364**, 1031–1038.

12 S. Paul, T. Choudhuri, S. Das, R. Pratap and A. K. Bagdi, *J. Org. Chem.*, 2024, **89**, 1492–1504.

13 A. Ponti and G. Molteni, *J. Org. Chem.*, 2001, **66**, 5252–5255.

14 G. Molteni, *HETEROCYCLES*, 2020, **100**, 1249.

15 Z.-L. Min, Q. Zhang, X. Hong, X.-L. Cao and X.-M. Hu, *Asian J. Chem.*, 2015, **27**, 3205–3207.

16 C. P. Lakeland, D. W. Watson and J. P. A. Harrity, *Chem. – Eur. J.*, 2020, **26**, 155–159.

17 L. Lv, Y. Chen, A. Shatskiy, J. Liu, X. Liu, M. D. Kärkäs and X. Wang, *Eur. J. Org. Chem.*, 2021, **2021**, 964–968.

18 W. Huang, C. Zhu, M. Li, Y. Yu, W. Wu, Z. Tu and H. Jiang, *Adv. Synth. Catal.*, 2018, **360**, 3117–3123.

19 G. Surendra Reddy, A. Suresh Kumar and D. B. Ramachary, *Org. Biomol. Chem.*, 2020, **18**, 4470–4478.

20 M. Kr. Barman, A. K. Sinha and S. Nembenna, *Green Chem.*, 2016, **18**, 2534–2541.

21 P. P. Sen and S. R. Roy, *Org. Lett.*, 2023, **25**, 1895–1900.

22 K. Nakayama and Y. Okada, *J. Org. Chem.*, 2023, **88**, 5913–5922.

23 D. Wang, X. Gan, L. Zhou, H. Shu and J. Zhou, *Tetrahedron Lett.*, 2022, **88**, 153587.

24 J. K. Pathak, O. S. Srivastava, R. Kant and N. Rastogi, *Adv. Synth. Catal.*, 2025, **367**, e202500300.

25 T. Onaka, H. Umemoto, Y. Miki, A. Nakamura and T. Maegawa, *J. Org. Chem.*, 2014, **79**, 6703–6707.

26 L.-H. Li, Z.-J. Niu, Y.-X. Li and Y.-M. Liang, *Chem. Commun.*, 2018, **54**, 11148–11151.

27 Y. Zheng, Y. Long, H. Gong, J. Xu, C. Zhang, H. Fu, X. Zheng, H. Chen and R. Li, *Org. Lett.*, 2022, **24**, 3878–3883.

28 L. Niu, H. Yi, S. Wang, T. Liu, J. Liu and A. Lei, *Nat. Commun.*, 2017, **8**, 14226.

29 D. Toummini, A. Tlili, J. Bergès, F. Ouazzani and M. Taillefer, *Chem. – Eur. J.*, 2014, **20**, 14619–14623.

30 L. S. Sharninghausen, A. F. Brooks, W. P. Winton, K. J. Makaravage, P. J. H. Scott and M.

S. Sanford, *J. Am. Chem. Soc.*, 2020, **142**, 7362–7367.
31 M. Pang, A. Ramazani, Z. Zhang and G. Zhang, *Org. Biomol. Chem.*, 2025, **23**, 2812–2817.
32 R. Sikari, S. Sinha, G. Chakraborty, S. Das, N. P. Van Leest and N. D. Paul, *Adv. Synth. Catal.*, 2019, **361**, 4342–4353.
33 Y. A. Vlasenko, T. J. Kuczmera, N. S. Antonkin, R. R. Valiev, P. S. Postnikov and B. J. Nachtsheim, *Adv. Synth. Catal.*, 2023, **365**, 535–543.
34 A. Saha, M. Roy, S. Maji, G. Rana, D. Maiti and D. Adhikari, *J. Am. Chem. Soc.*, 2025, **147**, 20735–20747.
35 H. Huang, Y. Jiang, W. Yuan and Y. Lin, *Angew. Chem. Int. Ed.*, 2024, e202409653.
36 J. K. Widness, D. G. Enny, K. S. McFarlane-Connelly, M. T. Miedenbauer, T. D. Krauss and D. J. Weix, *J. Am. Chem. Soc.*, 2022, **144**, 12229–12246.
37 Y. Sawama, A. Nakano, T. Matsuda, T. Kawajiri, T. Yamada and H. Sajiki, *Org. Process Res. Dev.*, 2019, **23**, 648–653.
38 F. O. Rodriguez del Rey and P. E. Floreancig, *Org. Lett.*, 2021, **23**, 150–154.
39 D. Toummini, A. Tlili, J. Bergès, F. Ouazzani and M. Taillefer, *Chem. – Eur. J.*, 2014, **20**, 14619–14623.
40 A. Y. Jiu, H. S. Slocumb, C. S. Yeung, X. Yang and V. M. Dong, *Angew. Chem. Int. Ed.*, 2021, **60**, 19660–19664.
41 P. P. Sen and S. R. Roy, *Org. Lett.*, 2023, **25**, 1895–1900.
42 T. Lee and J. F. Hartwig, *Angew. Chem.*, 2016, **128**, 8865–8869.
43 K. J. Hall, T. I. Quickenden and D. W. Watts, *J. Chem. Educ.*, 1976, **53**, 493.
